# Microbial Community Composition Reveals Spatial Variation and Distinctive Core Microbiome of the Weaver Ant *Oecophylla smaragdina* in Malaysia

**DOI:** 10.1038/s41598-018-29159-2

**Published:** 2018-07-17

**Authors:** Kah-Ooi Chua, Sze-Looi Song, Hoi-Sen Yong, Wah-Seng See-Too, Wai-Fong Yin, Kok-Gan Chan

**Affiliations:** 1 Institute of Biological Sciences, Faculty of Science, University of Malaya, 50603 Kuala Lumpur, Malaysia; 2 Institute of Ocean and Earth Sciences, University of Malaya, 50603 Kuala Lumpur, Malaysia; 3 International Genome Centre, Jiangsu University, Zhenjiang, China

## Abstract

The weaver ant *Oecophylla smaragdina* is an aggressive predator of other arthropods and has been employed as a biological control agent against many insect pests in plantations. Despite playing important roles in pest management, information about the microbiota of *O*. *smaragdina* is limited. In this work, a number of *O*. *smaragdina* colonies (n = 12) from Malaysia had been studied on their microbiome profile using Illumina 16S rRNA gene amplicon sequencing. We characterized the core microbiota associated with these *O*. *smaragdina* and investigated variation between colonies from different environments. Across all 12 samples, 97.8% of the sequences were assigned to eight bacterial families and most communities were dominated by families Acetobacteraceae and Lactobacillaceae. Comparison among colonies revealed predominance of Acetobacteraceae in *O*. *smaragdina* from forest areas but reduced abundance was observed in colonies from urban areas. In addition, our findings also revealed distinctive community composition in *O*. *smaragdina* showing little taxonomic overlap with previously reported ant microbiota. In summary, our work provides information regarding microbiome of *O*. *smaragdina* which is essential for establishing healthy colonies. This study also forms the basis for further study on microbiome of *O*. *smaragdina* from other regions.

## Introduction

The ant family Formicidae has 17 subfamilies, 333 genera and 13,263 valid species described^1^. The tropical forests in Malaysia have the greatest species diversity recorded but most of these ant species are poorly known and understudied^2^. Besides being famous for their developed social organization and behaviour of labour division, ants are known for harbouring complex bacterial community in their body^3,4^. Many studies have reported consistent association of individual bacterial taxa or whole communities with certain species of ants^5–7^. It is believed that symbiotic relationships of ants and microorganisms play an integral role in their evolutionary success^8^.

The ant-associated bacterial taxa and whole communities contribute to nutrition, reproduction and other physiological functions of the hosts which in their absence, influence the health and fitness of the insects^7,9–11^. The first insect endosymbiont *Blochmannia* species was discovered in ant genus *Camponotus* (Mayr, 1861)^12^. The intracellular bacteria were shown to give nutritional support to the host by providing essential amino acids which enhance the competitive ability of the host^13^. Apart from endosymbionts, there are also bacterial taxa that interact with ants as ectosymbionts. A famous example is the filamentous bacteria of genus *Pseudonocardia* that are acquired by fungus-growing Attine ants on their cuticle^14,15^. The group of bacteria produce antibiotics to suppress *Escovopsis*, a genus of virulent microfungal parasite that attacks the basidiomycetous fungi cultivated as predominant food source by Attine ants^4,15^. Over the years, studies on the microbiome of ants revealed a plenitude of knowledge from ant-microbe interaction. However, as compared to the diversity of the ant family, the studies on their microbiome are still limited, especially the ant species from tropical areas.

*Oecophylla smaragdina* (Fabricius, 1775), commonly known as weaver ant is an obligate arboreal species well distributed throughout Southeast Asia, Oriental regions of India and northern Australia^16,17^. The species constructs its nest on tree by weaving leaves together with silk from its larvae^2^ and a mature colony is huge, often consists of up to 500,000 ants^18^. *O*. *smaragdina* actively patrols various parts of trees and preys on a wide range of arthropods that enter their territory including insect pests^19^. Due to its aggressive predatory behaviour, *O*. *smaragdina* is therefore recognized as biological control agents for tree crops and has been suggested as an alternative to chemical pesticides^20^. To date, *O*. *smaragdina* has been reported effective in controlling pests in crops such as coconut^21^, cocoa^22^, citrus^23^, cashew^24^ and mango^19^.

In taking up the role in pest management, many challenges are faced by *O*. *smaragdina*. The ant often needs to compete for territory with other dominant ants living close to its nest^25^. In studies that described the ability of *O*. *smaragdina* in protecting coconut *Cocos nucifera* from its pest *Amblypelta cocophaga*, the ant needs to compete for occupancy of palm trees with a codominant but not beneficial ground-nesting ant *Pheidole megacephala* (Fabricius, 1793). In many cases, *O*. *smaragdina* is observed displaced from the site by *P*. *megacephala*^21,25^. Efforts and attempts to increase the distribution and abundance of *O*. *smaragdina*, including to kill competing ants with insecticides has been suggested but not extensively practiced^21^. This is because to ensure *O*. *smaragdina*’s success as biological control agents, the ant’s competitive ability should be enhanced by establishing healthy colonies, and microbiota associated with *O*. *smaragdina* is believed to play a critical role in the host fitness.

Microbiota associated with ants have been described with ability to improve the fitness of the host^4,13,26^, and the examples of insect microbiome with ability to influence the competence of the hosts do not just limit to the ant family. Studies have shown in insect aphids that harbour the endosymbiont Candidatus *Hamiltonella defensa* are protected from attack by parasitoid wasps as the bacterium is able to block the larval formation of the endoparasitoid wasp in the host^27,28^. To date, most microbiome studies focus on insects that are agricultural pests or vectors of diseases, with the aim to identify candidate microorganisms for insect pest control^29–31^. A successful case has been demonstrated by the intracellular bacterium *Wolbachia* that is responsible for expression of cytoplasmic incompatibility in mosquitoes and contributes to population control of the pest^32^. *Wolbachia* infections are widespread in ants^33,34^. The bacterium is highly prevalent in ant species such as *Formica exsecta* (Nylander, 1846)^35^ and genus *Solenopsis* (Westwood, 1840)^36^. However, *O smaragdina* has not been reported with *Wolbachia* infection so far^34^. As *O*. *smaragdina* has been playing an important role as biological control agent, the information about its microbiota would be valuable in the development of pest control and agricultural biotechnology.

From previous studies that explore the influence of geography and environment on microbiota of insects, it was revealed that different host conditions may cause variation in microbiota composition of an insect host^5,37–42^. However, in most cases, the hosts often associate with a particular group of microorganisms, termed as the core microbiota^3,37,43–45^. Characterizing the microbial community of insect hosts is thus essential for elucidating the normal healthy state of insects’ microbiota and to distinguish a disrupted microbiota that might indicate infection or disease in insects^37,46^. In this study, we identified the core microbiota of 12 *O*. *smaragdina* colonies in Malaysia using 16S rRNA gene amplification followed by high throughput Illumina sequencing. In tropical regions where *O*. *smaragdina* is abundant, the species has been observed surviving outside the forest area in unfavourable environments of urban city^47^, despite being an obligate arboreal ant. Hence, we included representative samples from different environments including forest areas, patchy green areas in urban city and urban areas with low abundance of greenery to obtain a holistic microbiome profile of *O*. *smaragdina* in the country. We also assessed the spatial variation in the microbiota of *O*. *smaragdina* by performing comparison among colonies from different environments.

## Results

### Data summary and alpha diversity analyses

A total of 12 16S rRNA gene libraries from *O*. *smaragdina* colonies sampled from different locations were subjected to Illumina paired-end sequencing (Supplementary Table S1). Sequencing of the libraries produced 22,999,352 raw reads. After stringent quality-filtering and chimera removal, we obtained 2,664,077 high quality sequences in our dataset with an average of 222,006 sequences per sample (minimum and maximum of 138,607 and 325,050 sequences per sample) (Supplementary Table S2).

Clustering the data with UCLUST^48^ at 97% sequence similarity produced 1,397 OTUs (Supplementary Table S3). When OTUs accounting for lesser than 0.5% of the total sequences were excluded, 97.8% of the sequences retained with 16 OTUs remained (Supplementary Table S4). From rarefaction analysis, the number of observed OTUs of all samples reached plateau at a sampling depth of 10,000 (Supplementary Fig. S1). The Good’s coverage estimates (Table 1) also indicated that the sequencing coverage was adequate in capturing the microbial diversity associated with *O*. *smaragdina*.

**Table 1:** Alpha diversity analysis after rarefaction to 50,000 sequences per sample of *O*. *smaragdina*.

Sample	Environment	Observed OTUs	Chao1	Shannon	Simpson	Good’s coverage
PGA1	Patchy green area in urban city	13	13.00	2.59	0.80	1.00
PGA2	Patchy green area in urban city	11	11.00	1.07	0.36	1.00
PGA3	Patchy green area in urban city	11	11.00	1.97	0.67	1.00
PGA4	Patchy green area in urban city	13	16.00	1.57	0.57	1.00
Forest1	Forest	9	9.00	1.80	0.62	1.00
Forest3	Forest	11	12.00	1.39	0.46	1.00
Forest4	Forest	12	12.00	1.83	0.63	1.00
Forest2	Forest	14	14.00	1.51	0.54	1.00
Urban1	Urban city	10	11.00	2.14	0.74	1.00
Urban2	Urban city	12	12.00	1.26	0.42	1.00
Urban3	Urban city	12	13.00	1.70	0.57	1.00
Urban4	Urban city	8	8.00	1.83	0.69	1.00

The Illumina sequencing revealed a relatively simple microbiota in the ant species. Only an average of 11 unique 97% OTUs per 50,000 sequences was observed in all the samples. Chao1, Shannon’s and Simpson’s diversity indices also indicated an overall low microbial diversity associated with the ant species (Table 1). Interestingly, lowest diversity was observed in *O*. *smaragdina* sampled from forest (mean Shannon index = 1.63; mean Simpson’s index = 0.56) than urban area (mean Shannon index = 1.73; mean Simpson’s index = 0.6) and patchy green area in urban city (mean Shannon index = 1.8; mean Simpson’s index = 0.6), despite being relatively close in term of richness as indicated by Chao1 analysis.

### Community composition of *O*. *smaragdina*-associated microbiota

**Figure 1:**
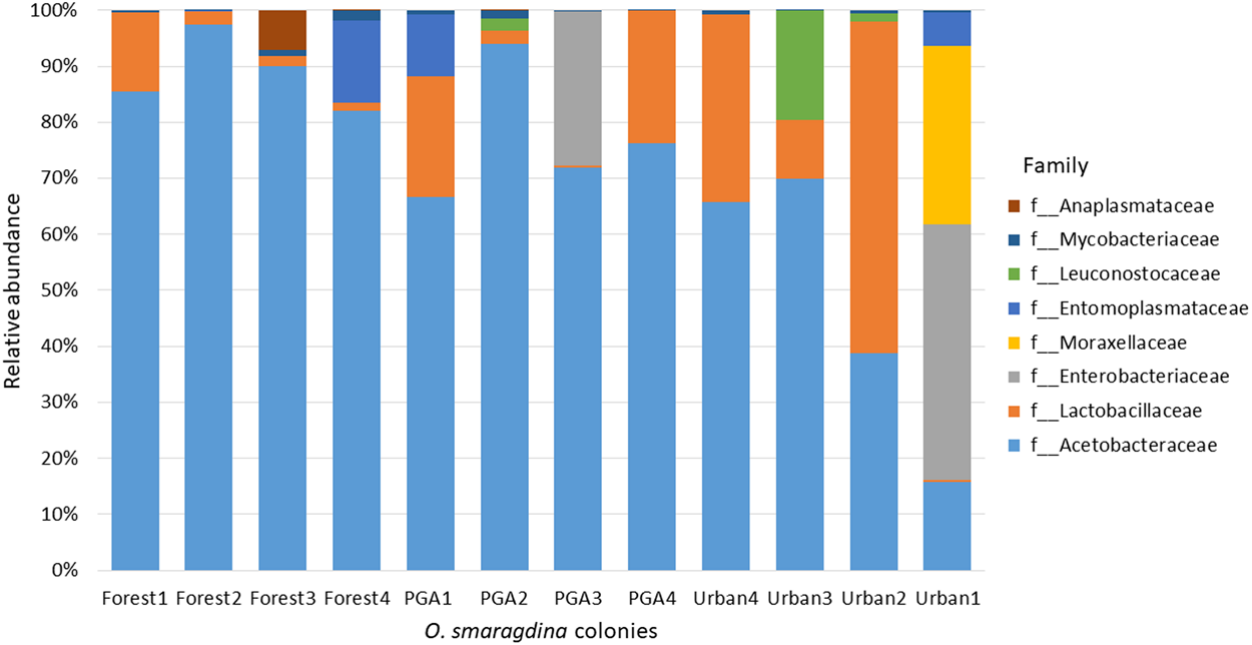
Relative abundance of bacterial families in community of *O*. *smaragdina* colonies. Colonies labelled Forest are from forest areas; colonies labelled PGA are from patchy green areas in urban cities; colonies labelled Urban are from urban areas with low abundance of green areas.

**Figure 2:**
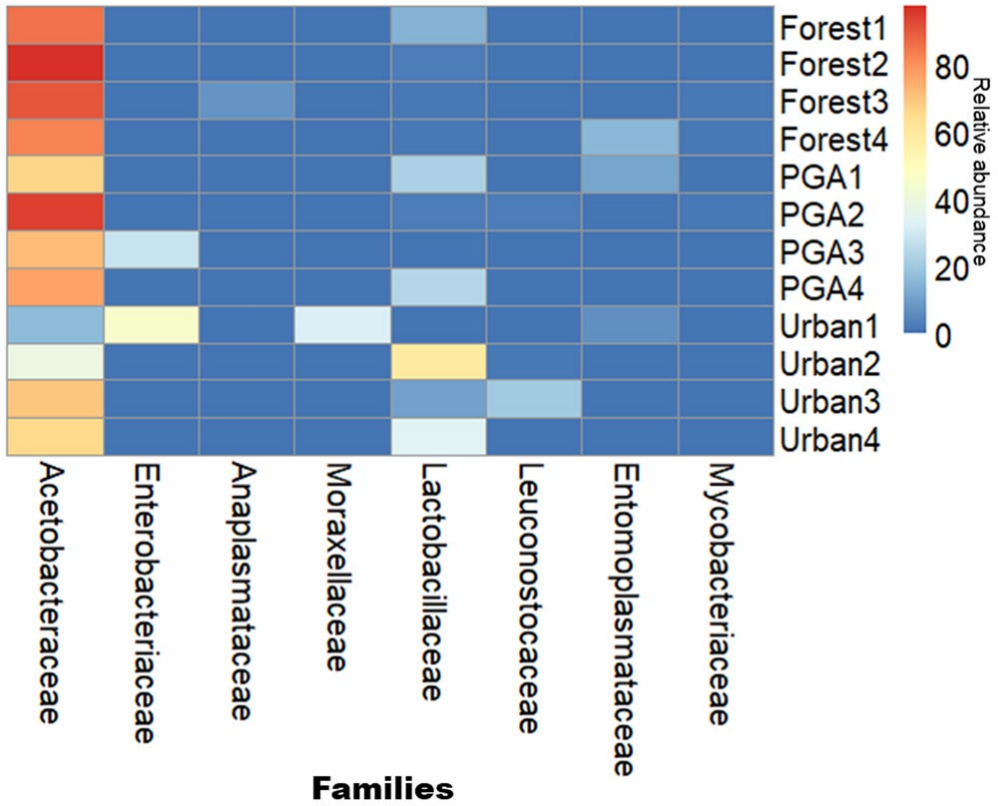
Heat maps showing the relative abundance of dominant bacterial families in microbiome of *O*. *smaragdina* colonies from forest areas (Forest1 to Forest4), patchy green areas in urban city (PGA1 to PGA4) and urban areas with low green abundance (Urban1 to Urban4).

The microbial communities associated with *O*. *smaragdina* appeared to be relatively simple. 97.8% of the sequences clustered into 16 major OTUs (all 0.5% or higher in total abundance) that were assigned to eight bacterial families, Acetobacteraceae (71.2%), Lactobacillaceae (14.3%), Enterobacteriaceae (6.1%), Moraxellaceae (2.7%), Entomoplasmataceae (2.6%), Leuconostocaceae (1.9%), Mycobacteriaceae (0.6%) and Anaplasmataceae (0.6%) (Fig. 1, Table 2). From heat map analysis on the relative abundance of each bacterial family across colonies from different environment, most *O*. *smaragdina* were dominated by family Acetobacteraceae, with the highest abundance observed in forest group samples (Fig. 2). High abundance of family Lactobacillaceae occurred in three urban group samples while the remaining families were generally in low abundance and occasionally occurred abundantly in few samples (Fig. 2).

**Table 2:** Relative abundance of family and genus level taxa from each *O*. *smaragdina* colony. Only OTUs of 0.5% or higher in total abundance are included.

Phylum	Order	Family	Genus	Forest1	Forest2	Forest3	Forest4	PGA1	PGA2	PGA3	PGA4	Urban1	Urban2	Urban3	Urban4
Proteo-bacteria	Rhodo-spirillales	Acetobacteraceae	*Acetobacter*	0.06	6.05	0	0	33.12	12.71	3.09	0.04	2.04	2.93	5	0.09
			*Asaia*	0.01	0.18	0	0	0.35	1.6	0	0.15	0	10.29	0	0
			*Gluconobacter*	4.35	1.42	13.94	8.6	1.03	0	2.23	2.54	0.07	0	0	0.32
			*Neokomagataea*	81.03	89.75	76.00	73.51	32.14	79.77	66.54	73.54	13.66	25.59	64.95	65.31
	Entero-bacteriales	Enterobacteriaceae	*Arsenophonus*	0	0	0	0	0	0	27.46	0	0	0	0	0
			*Enterobacter*	0.01	0.02	0	0	0.07	0.02	0.03	0	45.69	0.01	0	0
	Rickettsi-ales	Anaplasmataceae	*Wolbachia*	0	0	7.05	0	0	0	0	0	0	0	0	0
	Pseudo-monadales	Moraxellaceae	*Acinetobacter*	0	0.01	0	0.03	0	0.01	0.01	0	31.94	0	0	0
Firmi-cutes	Lacto-bacillales	Lactobacillaceae	*Lactobacillus*	14.17	2.47	1.86	1.42	21.57	2.21	0.41	23.65	0.30	59.15	10.44	33.62
		Leuconostocaceae	*Weissella*	0	0	0	0	0	2.26	0	0	0	1.50	19.53	0
Tene-ricutes	Entomo-plasma-tales	Entomoplasma-taceae	*Entomoplasma*	0	0.07	0.08	14.61	11.03	0.01	0.01	0	5.85	0.01	0	0
Actino-bacteria	Actinomy-cetales	Mycobacteriaceae	*Mycobacterium*	0.37	0.06	1.07	1.85	0.7	1.42	0.23	0.09	0.44	0.53	0.07	0.66

At the OTUs level, we compared the results of taxonomy assignment to green genes 13_8-release database and SILVA128 SSU Ref NR database and most of the identity of OTUs from both databases were in consensus (Supplementary Tables S3 and S4). Further BLASTn analysis revealed the nearest identity of the 16 major OTUs at genus and species levels with varying range of similarity (91.32% to 100%) (Supplementary Table S4). Network analysis on the major OTUs showed a high degree of shared OTUs between the *O*. *smaragdina* from different environments, especially OTUs of family Acetobacteraceae with 4 OTUs found in every sample (except OTU1012 that was not found in two colonies from urban area) (Supplementary Fig. S3).

Most microbial communities of *O*. *smaragdina* were dominated by family Acetobacteraceae (order Rhodospirillales) that harboured two core OTUs (OTU424 and OTU636, both were identified as genus *Neokomagataea*) with several OTUs that had lower relative abundance (Fig. 1, Table 2, Supplementary Table S4). Further BLASTn search to NCBI 16S microbial database revealed that the sequences had ≥ 92.17% similarity with bacteria from the genera in family Acetobacteraceae. We observed that the relative abundance of family Acetobacteraceae in *O*. *smaragdina* differed by environment the colonies thrived in (F_2, 9 = _6.560, P < 0.05, Supplementary Table S5). Highest abundance of Acetobacteraceae occurred in colonies from forest area (mean = 88.72% ± 6.61), with a gradual decrease in colonies from patchy green area in urban city (mean = 77.21% ± 11.91) to colonies from urban area with low abundance of green area (mean = 47.56% ± 25.28) (Fig. 2, Supplementary Fig. S2).

On the other hand, the abundance of family Lactobacillaceae (order Lactobacillales) was variable in microbial communities of *O*. *smaragdina*. Relative abundance of Lactobacillaceae was low in most *O*. *smaragdina* from forest area (range from 1.42% to 14.17%). A second BLASTn analysis revealed that all OTUs under family Lactobacillaceae shared ≥93.67% similarity with bacteria from genus *Lactobacillus*. In two out of four colonies from patchy green area of urban city, the family constituted 21.57% and 23.65% of the sequences but the remaining two colonies had less than 5% (Fig. 1, Table 2). Highest abundance of Lactobacillaceae occurred in three colonies from urban areas, and became the most dominant family in colony Urban2, although the observation was not consistent in colony Urban1 from urban area (Table 2, Fig. 2).

Other than the two dominant families, the family Mycobacteriaceae (order Actinomycetales) represented by OTU1052 and family Moraxellaceae (order Pseudomonadales) represented by OTU1193 were detected at high prevalence. Family Mycobacteriaceae was consistently low in relative abundance in all the samples. Family Moraxellaceae was only observed at high abundance in colony Urban1 although it had been observed in 100% of the samples (Fig. 1, Table 2).

In addition to the highly prevalent OTUs, five families distributed sporadically but occasionally high prevalence in the community of *O*. *smaragdina*. Of these, family Enterobacteriaceae (represented by OTU1277 and OTU633) had relative abundance as high as 45.69% in colony Urban1 and 27.46% in colony PGA3. Besides, family Leuconostocaceae (OTU965) was absent in most of the colonies but had a relative abundance of 1.50% in colony Urban2, 2.26% in colony PGA2, and 19.53% in colony Urban3. The family Anaplasmataceae (represented by OTU1176) was almost unique to colony Forest3 with relative abundance of 7.05%, although its presence was detected in other samples with lesser than 0.01% (Fig. 1, Table 2).

### Beta diversity analysis

**Figure 3:**
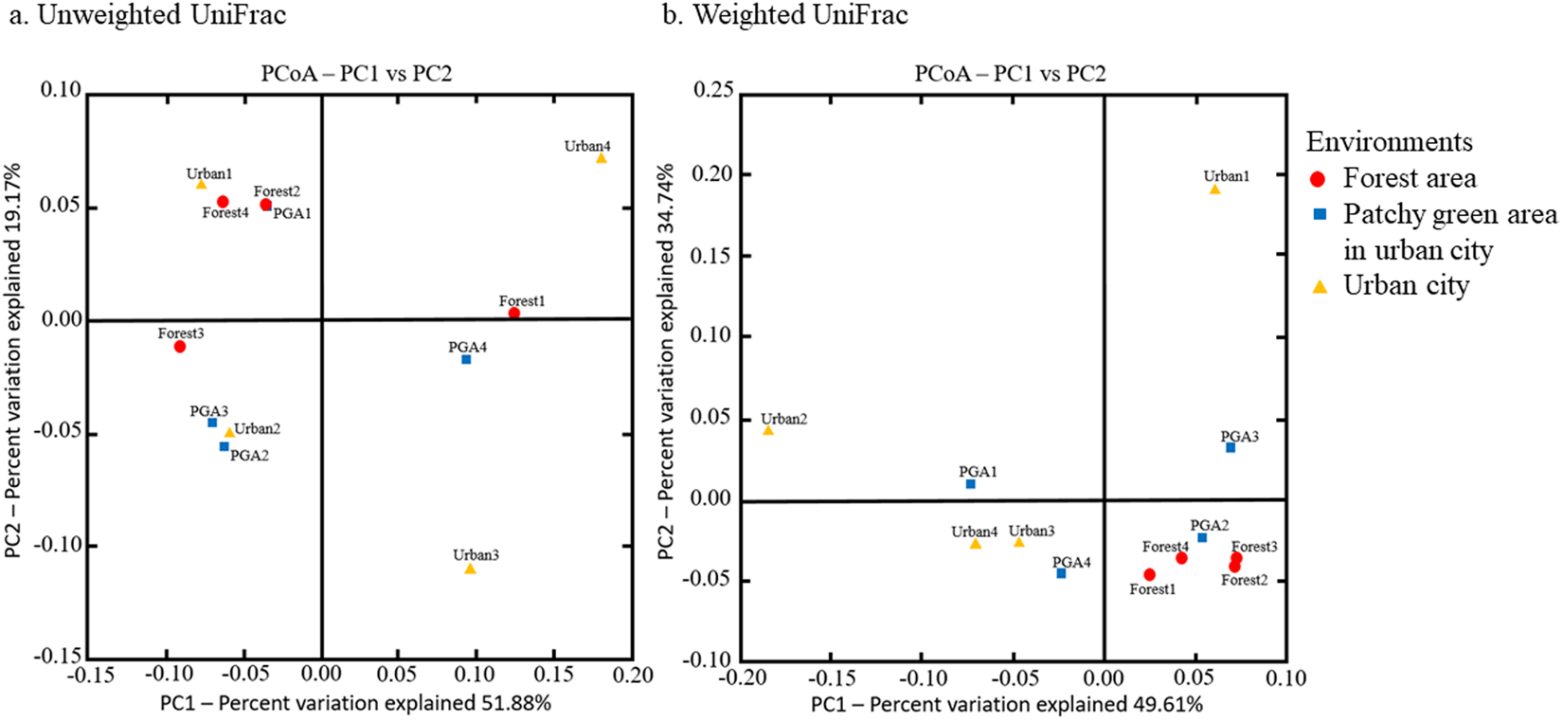
Principal coordinate analysis (PCoA) of (**a**) Unweighted UniFrac distances and (**b**) Weighted UniFrac distances for all OTUs associated with *O*. *smaragdina* colonies in forest areas, patchy green areas in urban city and urban areas with low green abundance.

Abundance-weighted and unweighted UniFrac are beta diversity metrics that incorporate phylogenetic relationship of taxa in measuring community similarity^49^. From PCoA plot for unweighted UniFrac that accounts only for presence and absence of taxa, no pattern of correlation was observed among the communities (Fig. 3a). We related the result to presence of taxa that were sporadically distributed among the communities.

As for abundance-weighted UniFrac analysis, clear separation of forest group colonies from the urban area colonies was observed. The microbial communities of forest *O*. *smaragdina* colonies were closely related and clustered together (Fig. 3b). Samples from patchy green area of urban city and urban area were distantly related from each other, with samples from urban area group showing the largest distance within group. The first two coordinates explained in total 84.35% of the variation in the data. We related the result to the variation in abundance of the most dominant taxa. Colonies from forest areas had characteristically highest abundance of family Acetobacteraceae and least abundance of family Lactobacillaceae. In contrast, high abundance of family Lactobacillaceae only occurred in colonies from urban areas and was predominant in Urban2 (Fig. 2).

## Discussion

Our findings revealed the common bacteria taxa associated with *O*. *smaragdina* workers and showed that its community composition is generally stable as 97.8% of over 2 million quality-filtered reads were clustered into only 16 major OTUs which were assigned into 8 bacterial families (Supplementary Tables S2 and S4). We found two dominant taxa of acidophilic bacteria, families Acetobacteraceae and Lactobacillaceae to make up 87.2% of the *O*. *smaragdina* microbiome and are likely gut inhabitants. Genera from both families are known to tolerate various sugar-rich and acidic environments and constitute gut community in many insects^50,51^. We also relate the predominance of Acetobacteraceae and Lactobacillaceae to the high formic acid content in the body of *O*. *smaragdina* worker ants^52^. When a prey is encountered, the aggressive worker ants attack by biting and spraying formic acid on the area of bite from the tips of their abdomen^53^. Both dominant families Acetobacteraceae and Lactobacillaceae were represented by a few OTUs of varying relative abundance and assigned to different species in the family (Table 2, Supplementary Table S4). These findings suggest that the microbiota of *O*. *smaragdina* is dominated by a few bacterial strains.

OTUs of the most abundant family Acetobacteraceae were closely related to acetic acid bacteria from plant-associated bacteria such as genera *Neokomagataea*^54^, *Asaia*^55^, *Acetobacter*^56^, as well as genus *Gluconobacter* previously found in insect^57^ (Supplementary Table S4). Bacteria from this family had also been reported in microbiome of Camponotini ants^6^ and Argentine ants^58^. More interestingly, several species of family Acetobacteraceae that were identified as bacterial symbiont in other insects are actually cultivable bacteria. For instance, *Acetobacter tropicalis* that is the major symbiont of the fruit fly *Bactrocera oleae*^59^, *Asaia bogorensis* and *A*. *siamensis* that are constantly associated with mosquito *Anopheles stephensi*^60^ had all been successfully cultivated in laboratory following microbiome studies on the insect hosts. Most of these symbiotic acetic acid bacteria have been shown able to colonize the gut of insects and survive under acidic pH with the availability of diet-derived carbohydrates and oxygen^50^.

On the other hand, BLASTn analysis revealed that all OTUs under family Lactobacillaceae shared ≥93.67% similarity with bacteria from genus *Lactobacillus* and most of them were closely related to *Lactobacillus* found in fermented food^61–63^ and plant^64^ (Supplementary Table S4). Besides being a well-known commensal microorganism in human and other animals, *Lactobacillus* has also been detected in microbiome study of insects including bee^37^, fruit fly^30^ as well as ants^65–67^. Although the role of *Lactobacillus* in *O*. *smaragdina* is unknown, *Lactobacillus* isolated from the crop of honeybees shown ability to produce potent antimicrobial and defends the host from microbial infections^68^. Among the remaining OTUs, families Enterobacteriaceae and Entomoplasmataceae were detected in microbiome of many ants as symbionts^38,65,69^, although Entomoplasmataceae that forms a unique lineage in army ants has no essential contribution to the growth or development of the host^69^.

This study assessed the spatial variation in microbiome of *O*. *smaragdina* from different environments. High sequencing depth had been allocated for each sample to obtain a holistic microbiome profile from all samples. This has been indicated by rarefaction analysis where individual curves reached plateau and saturation (Supplementary Fig. S1). Despite the overall similarity in community composition, differences in term of abundance of the dominant families exist between *O*. *smaragdina* colonies from different environments, especially for the family Acetobacteraceae (Supplementary Fig. S2). As shown in PCoA plot for abundance-weighted UniFrac distance, the forest group samples formed a cluster that is separated from the samples from other environments, while the urban area samples were distantly related to each other (Fig. 3b). We related the observation to the possible differences in feeding habits between *O*. *smaragdina* from forest area and urban area.

In Southeast Asia where *O. smaragdina* is dominant, the arboreal ant distributes across wide range of habitats from forests^70^, plantations^71,72^, urban areas^47^ to mangrove swamp^73^. Food resources play important roles in supporting *O. smaragdina* colonies, which often comprise up to hundreds of thousands of workers at mature stage^18^. In addition to preying on other arthropods, *O. smaragdina* also consumes plant-derived extrafloral nectar and honeydew and tends homopterans in return for the honeydew produced^74,75^. As compared to arthropod preys, plant sap is generally more and in sufficient supply to compensate the high energy requirement in huge colonies of *O. smaragdina*^16^. However, nectar and honeydew are food sources that are rich in carbohydrate but scarce in protein (nitrogen). To obtain optimal colony growth both carbohydrate from honeydew and nectar and protein from arthropod preys are required^76^.

Unfortunately, urbanization causes fragmentation of forests into patchy green areas and destroys natural habitats of living species, leading to decline of biodiversity^77,78^. Clearing of large area of vegetation alters species composition as certain species become locally extinct^79^. Even if a species survives the process of urbanization, its functional roles are often affected in the urban environment. Among the affected species, ants have been recognized with remarkable ability to adapt to urban habitats^80^. However, the changes of vegetation and plant growth may affect the food choice and feeding habits of ants living in urban areas^81^.

Host diet plays an important role in shaping the microbiota of insects^82^. In a microbiome study that involved the ant *Azteca trigona* (Emerly, 1893), it was postulated that differences in diet caused significant microbiota variation across colonies^40^. Besides, *Cephalotes varians* (Smith, 1876) worker ants experienced significant changes in their microbiota when they were fed with pollen for one to two months^44^. In a recent diet manipulation study on Argentine workers ants (*Linepithema humile*, (Mayr, 1868)), a carbohydrate-rich and protein-poor diet resulted in dominance of bacteria of family Acetobacteraceae (order Rhodospirillales) in the gut communities. By comparison, gut communities of the ants experienced a reduction in the same group of bacteria when treated with low carbohydrate and high protein diet^58^.

In this study, similar bacteria taxon (family Acetobacteraceae, order Rhodospirillales) was observed in highest abundance within community composition of forest group *O*. *smaragdina* (Fig. 2). Colonies from forest areas are believed to have more access to plant-derived extrafloral nectar and honeydew and encounter higher density of homopterans that produce honeydew in their living environment. High consumption of carbohydrate-rich resources may therefore result in predominance of family Acetobacteraceae in their community. In contrast, *O*. *smaragdina* colonies from urban areas have the least abundance of family Acetobacteraceae. They are affected as mature trees that serve as its nesting site are removed for urban development. When diversity of plant trees and insects decreases in urban city, food resources of *O*. *smaragdina* colony are affected as plant-derived resources and honeydew-producing homopterans are scarce. In times of carbohydrate food scarcity, an omnivorous ant colony increases foraging activity for insect preys and thus relies more on protein-rich resources^83^.

Even though *O*. *smaragdina* has a wide distribution, the species is confined to tropical regions of Asia and having similar ecological niches. As samples were not collected for biogeographic comparisons, this study focused primarily on *O*. *smaragdina* colonies in Malaysia. Nonetheless, our findings obtained are in concordance to other reported ant species, whose microbiome comprised of dominant members consistently present across all samples and a core microbiome had been identified. Notably, a substantial amount of studies had demonstrated that geographical location of ant species had no correlation with overall microbiome diversity^5,6,58,65^.

To date, herbivorous species have been the focus of study on microbiota of ants and only a few reported for omnivorous species^13,40,41,58^. Our findings revealed distinctive community composition in *O*. *smaragdina* from other herbivorous arboreal ant species. For examples, the turtle ants (genus *Cephalotes*) stably associate with bacteria of phylum Verrucomicrobia (order Opitutales)^3,5,44,45^ and nitrogen-fixing bacteria of order Rhizobiales have been detected in ant genus *Tetraponera* (Smith, 1852)^26,84^. The community associated with omnivorous *O*. *smaragdina* was mainly dominated by bacteria from families Acetobacteraceae and Lactobacillaceae (Fig. 1). Unlike the herbivorous ants that rely solely on plant exudates, omnivorous *O*. *smaragdina* obtains nitrogen and protein from preying on other arthropods and possesses a distinctive community composition^2^.

Distinctive from herbivorous ants, greater taxonomic overlap is observed among community compositions of *O*. *smaragdina* and some omnivorous ants. Among omnivorous ants, the community composition of carpenter ants (genus *Camponotus*) is predominated by obligate bacterial endosymbiont *Blochmannia* species that provide essential amino acids for growth of the hosts^7,11,13,85^. Additionally, in a recent study that investigated the microbial composition across ants of genus *Polyrhachis* (Smith, 1857) that were omnivorous, multiple novel *Blochmannia* strains were recovered and some of the strains displayed host fidelity with different subgenera of the ant genus. In contrast, we observed no *Blochmannia* or any closely related taxa in community of *O*. *smaragdina*.

However, in addition to *Blochmannia*, family Acetobacteraceae have been reported in high prevalence in *Camponotus chromaiodes* (Bolton, 1995) and other members of the genus^6^. Furthermore, phylogenetic analysis on Acetobacteraceae across the genus *Camponotus* ants revealed that the bacteria formed a monophyletic clade with other ant-associated Acetobacteraceae with accelerated substitution rates in their 16S rRNA gene^6^. Similar to the Camponotini ants, bacteria Acetobacteraceae were among the dominant and persistent taxa of the omnivorous *O*. *smaragdina* in this study. From the BLASTn analysis, it was revealed that Acetobacteraceae in *O*. *smaragdina* were showing high evolutionary rates as these OTUs shared only 92.17% to 96.77% identity in 16S rRNA gene with described taxa of Acetobacteraceae in database (Supplementary Table S4).

Among the ant species with reported microbiota, the bacteria community composition of *O*. *smaragdina* has higher similarity with Argentine ants (*Linepithema humile*)^58^. Although both the omnivorous species were dominated by families Acetobacteraceae and Lactobacillaceae, Lactobacillaceae is the predominant taxon in Argentine ants. Furthermore, dominance of Lactobacillaceae in community of Argentine ants remains stable in both native populations and colonies in introduced regions despite a shift from typical carnivorous diet towards sugar-rich, nitrogen-poor foods in its introduced range^58^. In contrast, the predominant family Acetobacteraceae in *O*. *smaragdina* colonies varied between environments and was most abundant in the forest areas but reduced in the patchy green areas in urban city and urban areas.

*O*. *smaragdina* is known to be effective in controlling over 50 species of insect pests that infest many tropical tree crops^19,86^. Our findings in this study had shown that colonies of *O*. *smaragdina* from similar environments in Malaysia possess highly similar microbiome, especially among the group of colonies from forest with high abundance of Acetobacteraceae (Fig. 1, Table 2). The maintenance of a stable microbiome is vital to the development of *O*. *smaragdina* colonies in plantations of different crops. Observations from a study on mango orchards revealed that only trees with abundant level of *O*. *smaragdina* had significantly lesser shoots damage by the red-banded thrips, as compared to trees with fewer *O*. *smaragdina* ants and trees without^19^. The consistent association *O*. *smaragdina* with its microbiota might be the driving factor for its success as biological control agent against insect pests in different crop plantations. In addition to pest control, insecticides were sprayed less frequently in citrus orchards and expenditures on insecticides reduced by half when *O*. *smaragdina* is abundant^87^. This avoids the use of highly hazardous insecticides which could be harmful to the health of consumers and environments^87^.

## Conclusion

The data presented in this study provide insight into the microbial community composition associated with the weaver ant *O*. *smaragdina* in Malaysia across different environments. The bacteria families Acetobacteraceae and Lactobacillaceae occurred in high prevalence and abundance in all colonies and constituted the core microbiota of the ant species. Despite community similarity in terms of bacterial taxa, abundance of the dominant families among ant colonies varied between environments, as observed with family Acetobacteraceae that has highest abundance in forest group colonies but lowest in colonies from urban area. Such differences may indicate change of functional roles from forest areas to fragmented forests in urban city and urban city with low abundance of green area, enhanced by the change in food choice and feeding habits in different living environments. This study formed the basis for continued research efforts which will be beneficial for elucidating the microbiota of *O*. *smaragdina* from other regions.

## Methods

### Specimen collection

*O*. *smaragdina* major workers were obtained from 12 distinct colonies from locations in Malaysia (Supplementary Table S1). All colonies are distantly separated and no interaction was observed among the colonies, including Forest3 and Forest4 (located 1 km from each other). While most samples were obtained from Selangor and Kuala Lumpur, we also included sample Forest1 from Perak state and sample PGA1 from Borneo Malaysia. These locations represent three different environments including forest, patchy green areas in urban city and urban areas with low abundance of greenery. Samples were collected from different environments to explore the influence of environments on the microbiota composition.

Sampling was performed within the period August to October 2016. *O*. *smaragdina* lives in the leaf nest of mature tropical trees which are often difficult to reach^19^. As the species exhibits worker polymorphism and the smaller minor worker ants generally stay inside the nest and tend to queen and brood^2,88^, we thus sampled only major worker ants that were foraging outside the nest. Multiple (10 to 15) major workers were captured from colony of every site and the specimens were immediately preserved in 95% molecular grade ethanol upon collection. The taxonomic identification were determined morphologically^47^ and specimens were deposited in the collection of Microbiome Lab in University of Malaya. The remaining specimens were stored at −20 °C until DNA extraction.

### DNA extraction

Whole ants were removed from ethanol and rinsed several times with nuclease-free water. Three worker ants randomly picked from collection of each site were pooled into one sample and homogenized with sterile micropestle in microcentrifuge tube. Prior to DNA extraction, a two hours lysozyme (20 mg/mL, Sigma-Aldrich) pre-treatment at 37 °C was performed on homogenized samples. Total genomic DNA was extracted with DNeasy blood and tissue kit (Qiagen, Valencia, CA). Purified DNA was eluted in elution buffer (EB) (Qiagen) and stored in −20 **°**C.

### 16S rRNA gene amplification and sequencing

The hypervariable V3-V4 region of the 16S rRNA gene was PCR amplified using the following primer pair: forward 5′-TCGTCGGCAGCGTCAGATGTGTATAAGAGACAGCCTACGGGNGGCWGCAG-3′and reverse 5′-GTCTCGTGGGCTCGGAGATGTGTATAAGAGACAGGACTACHVGGGTATCTAATCC-3′ (underlined letters denote the Illumina overhang adapter sequences)^89^. The PCR mixture consisted of KAPA HiFi HotStart ReadyMix (2X, KAPA Biosystems), 1 µM of each primers and 25 ng of DNA template in a final volume of 25 µl. The PCR parameters (T100 Thermal Cycler, Bio-rad, USA) were 95 **°**C for 3 min, followed by 25 cycles of 95 **°**C for 30 s, 55 **°**C for 30 s, 72 **°**C for 30 s. A final extension step at 72 **°**C for 5 min was added. An aliquot of 2 µl of each PCR product was checked for correct size (~550 bp) on a 1% agarose gel. The remaining PCR product was purified with Agencourt Ampure beads (Agencourt Bioscience Coorporation, MA, USA) and used for sequencing library preparation. The libraries were quantified using KAPA library quantification kit, normalized, pooled and sequenced on the Illumina MiSeq (2 × 250 bp paired-end read).

### Sequence analysis

Demultiplexed raw fastq files of each 16S rRNA gene library were generated by MiSeq Reporter Software. The paired-end reads were imported and quality-filtered in CLC Genomic Workbench v.7.5.1 (https://www.qiagenbioinformatics.com/). Ambiguous bases, primer sequences and low quality reads (below phred score of 20) were trimmed^90^. The paired-end reads were merged at overlapping regions and sequences shorter than 200 bp were discarded. Chimera removal was performed by implementing UCHIME through the USEARCH (v8.1) program with reference to the Gold database (http://www.drive5.com/usearch/manual/otupipe.html, downloaded June 8, 2016)^48,91^. Further analyses was carried out using QIIME v.1.9.0^92^.

The quality-filtered sequences were clustered into Operational taxonomic units (OTUs) using UCLUST^48^ in QIIME based on sequence similarity at 97%. A *de novo* approach was employed in OTUs picking to ensure all reads are clustered. A representative sequence was selected for each OTU and assigned for taxonomy with reference to the green genes 13_8-release database^93^ and SILVA128 SSU Ref NR database^94^. The representative sequences were aligned using PyNAST^95^ and a phylogenetic tree was built using FastTree^96^. An OTU table in biom format was created. OTUs accounting for less than 0.5% of the total number of sequences were excluded from further analysis as many of them consisted of singletons and were actually sequencing errors^90^. The major OTUs were blasted against the NCBI 16S microbial database for taxonomic assignment into genus and species level. A heatmap was constructed with OTUs that accounted for more than 0.5% of the total sequences using pheatmap R package^97^. To examine the interaction between OTUs and samples, an edge table was generated in QIIME and visualized using Cytoscape 3.6.1^98^.

Alpha diversity indices (including Chao1, Shannon, Simpson and observed OTUs) and Good’s coverage^99^ were calculated in QIIME on sample sizes normalized to 50,000 sequences per sample. To compare community from different colonies, samples were rarefied to the smallest dataset and beta diversity was analysed by abundance-weighted and unweighted UniFrac^49^. Weighted and unweighted UniFrac distances were calculated to examine the variation between microbial communities of *O*. *smaragdina* colonies. The resulting distance matrices were used for principal coordinates analyses (PCoA) and visualized in PCoA plots.

### Data availability

The raw datasets for 16S rRNA gene amplicon sequencing generated for this paper have been deposited in the GenBank Sequence Read Archive (accession number SUB2165087).

## Electronic supplementary material

Supplementary Table S1, Supplementary Table S2, Supplementary Table S3, Supplementary Table S4, Supplementary Table S5, Supplementary Fig. S1, Supplementary Fig. S2, Supplementary Fig. S3
