# Supplementary material for: Microbial Community Composition Reveals Spatial Variation and Distinctive Core Microbiome of the Weaver Ant Oecophylla smaragdina in Malaysia
Source: Sci Rep. 2018 Jul 17;8:10777. doi: 10.1038/s41598-018-29159-2 (PMC6050294; doi:10.1038/s41598-018-29159-2)
Supplement: Supplementary file 1 — Supplementary Table S1, Supplementary Table S2, Supplementary Table S3, Supplementary Table S4, Supplementary Table S5, Supplementary Fig. S1, Supplementary Fig. S2, Supplementary Fig. S3 [file 41598_2018_29159_MOESM1_ESM.pdf]

Microbial Community Composition Reveals Spatial Variation and Distinctive Core Microbiome of the Weaver Ant *Oecophylla smaragdina* in Malaysia

Kah-Ooi Chua[1], Sze-Looi Song[2], Hoi-Sen Yong[1], Wah-Seng See-Too[1], Wai-Fong Yin[1], Kok-Gan Chan[1,3*]

[1] Institute of Biological Sciences, University of Malaya, 50603 Kuala Lumpur, Malaysia
[2] Institute of Ocean and Earth Sciences, University of Malaya, 50603 Kuala Lumpur, Malaysia
[3] International Genome Centre, Jiangsu University, Zhenjiang, China

[*] Corresponding author @ kokgan@um.edu.my

Sample ID	Location	Coordinates	Environment	Collection date
Forest1	Perak, Gopeng	4°29’14.62”N 101°10’21.24”E	Forest	5 August 2016
Forest2	Selangor, Serendah	3°22'55.5"N 101°37'27.8"E	Forest	8 October 2016
Forest3	Selangor, Bukit Lagong	3°13'31.0"N 101°36'58.7"E	Forest	26 September 2016
Forest4	Selangor, Bukit Lagong	3°13'36.4"N 101°37'27.7"E	Forest	26 September 2016
PGA1	Sarawak, Miri	4°22'03.4"N 113°58'45.3"E	Patchy green area in urban city	30 September 2016
PGA2	Kuala Lumpur, University of Malaya	3°07'21.9"N 101°39'22.7"E	Patchy green area in urban city	23 September 2016
PGA3	Selangor, Petaling Jaya	3°08'50.8"N 101°37'55.3"E	Patchy green area in urban city	13 October 2016
PGA4	Selangor, Rawang	3°17'12.2"N 101°38'20.2"E	Patchy green area in urban city	8 October 2016
Urban1	Kuala Lumpur, Kepong	3°12'12.7"N 101°37'30.8"E	Urban city	22 September 2016
Urban2	Kuala Lumpur, Kepong	3°11'17.1"N 101°39'18.7"E	Urban city	22 September 2016
Urban3	Selangor, Petaling Jaya	3°06'14.4"N 101°38'56.6"E	Urban city	13 October 2016
Urban4	Selangor, Petaling Jaya	3°07'30.7"N 101°38'19.7"E	Urban city	10 October 2016

Supplementary Table S1 List of *O. smaragdina* samples included in this study.

Sample ID	No. of sequence (raw reads, pre-QC)	No. of sequence (post-QC)	No. of sequence (after chimera removal)
PGA1	1680876	138794	138607
Forest1	1892764	325199	325050
Urban1	1967466	181927	181763
Urban2	1827554	164805	164752
Forest3	1884022	219370	219361
Forest4	1648604	226497	226395
PGA2	2460414	223770	223719
PGA3	1863158	238111	237945
Urban3	2279486	250037	249966
Forest2	1756780	223865	223814
PGA4	1485422	202428	202278
Urban4	2252806	270509	270427

Supplementary Table S2 Summary of 16S rRNA gene sequencing data.

OTUs	Length of OTUs (bp)	Green_gene_13_88_taxonomy	SILVA128 taxonomy	BLASTn top hit	Percentage identity (%) (BLASTn)
OTU1052	438	k__Bacteria; p__Actinobacteria; c__Actinobacteria; o__Actinomycetales; f__Mycobacteriaceae; g__*Mycobacterium*	k__Bacteria; p__Actinobacteria; c__Actinobacteria; o__Corynebacteriales; f__nbr16a11; g__uncultured bacterium; s__uncultured bacterium	*Mycobacterium intermedium* strain 1669/91 16S ribosomal RNA gene, partial sequence	91.32
OTU1316	458	k__Bacteria; p__Firmicutes; c__Bacilli; o__Lactobacillales; f__Lactobacillaceae; g__*Lactobacillus*	k__Bacteria; p__Firmicutes; c__Bacilli; o__Lactobacillales; f__Lactobacillaceae; g__*Lactobacillus*	*Lactobacillus hokkaidonensis* strain LOOC260 16S ribosomal RNA gene, partial sequence	96.94
OTU411	458	k__Bacteria; p__Firmicutes; c__Bacilli; o__Lactobacillales; f__Lactobacillaceae; g__*Lactobacillus*	k__Bacteria; p__Firmicutes; c__Bacilli; o__Lactobacillales; f__Lactobacillaceae; g__Lactobacillus; s__uncultured *Lactobacillus* sp.	*Lactobacillus sanfranciscensis* strain JCM 5668 16S ribosomal RNA gene, partial sequence	99.13
OTU474	458	k__Bacteria; p__Firmicutes; c__Bacilli; o__Lactobacillales; f__Lactobacillaceae; g__*Lactobacillus*	k__Bacteria; p__Firmicutes; c__Bacilli; o__Lactobacillales; f__Lactobacillaceae; g__*Lactobacillus*	*Lactobacillus lindneri* strain KPA 16S ribosomal RNA gene, complete sequence	96.07
OTU55	458	k__Bacteria; p__Firmicutes; c__Bacilli; o__Lactobacillales; f__Lactobacillaceae; g__*Lactobacillus*	k__Bacteria; p__Firmicutes; c__Bacilli; o__Lactobacillales; f__Lactobacillaceae; g__*Lactobacillus*	*Lactobacillus malefermentans* strain NBRC 15905 16S ribosomal RNA gene, partial sequence	93.67
OTU965	458	k__Bacteria; p__Firmicutes; c__Bacilli; o__Lactobacillales; f__Leuconostocaceae; g__*Weissella*	k__Bacteria; p__Firmicutes; c__Bacilli; o__Lactobacillales; f__Leuconostocaceae; g__*Weissella*	*Weissella halotolerans* strain NRIC 1627 16S ribosomal RNA gene, complete sequence	100
OTU1012	434	k__Bacteria; p__Proteobacteria; c__Alphaproteobacteria; o__Rhodospirillales; f__Acetobacteraceae	k__Bacteria; p__Proteobacteria; c__Alphaproteobacteria; o__Rhodospirillales; f__Acetobacteraceae	*Asaia krungthepensis* strain NBRC 100057 16S ribosomal RNA gene, partial sequence	93.81
OTU1024	434	k__Bacteria; p__Proteobacteria; c__Alphaproteobacteria; o__Rhodospirillales; f__Acetobacteraceae	k__Bacteria; p__Proteobacteria; c__Alphaproteobacteria; o__Rhodospirillales; f__Acetobacteraceae; g__uncultured; s__uncultured bacterium	*Acetobacter orleanensis* strain JCM 7639 16S ribosomal RNA gene, partial sequence	92.17
OTU424	440	k__Bacteria; p__Proteobacteria; c__Alphaproteobacteria;	k__Bacteria; p__Proteobacteria; c__Alphaproteobacteria;	*Neokomagataea tanensis* strain BCC 25711 16S	94.33

		o__Rhodospirillales; f__Acetobacteraceae	o__Rhodospirillales; f__Acetobacteraceae	ribosomal RNA gene, partial sequence	
OTU636	433	k__Bacteria; p__Proteobacteria; c__Alphaproteobacteria; o__Rhodospirillales; f__Acetobacteraceae	k__Bacteria; p__Proteobacteria; c__Alphaproteobacteria; o__Rhodospirillales; f__Acetobacteraceae	*Neokomagataea tanensis* strain BCC 25711 16S ribosomal RNA gene, partial sequence	96.77
OTU637	433	k__Bacteria; p__Proteobacteria; c__Alphaproteobacteria; o__Rhodospirillales; f__Acetobacteraceae	k__Bacteria; p__Proteobacteria; c__Alphaproteobacteria; o__Rhodospirillales; f__Acetobacteraceae; g__*Gluconobacter*	*Gluconobacter morbifer* strain G707 16S ribosomal RNA gene, partial sequence	93.3
OTU633	458	k__Bacteria; p__Proteobacteria; c__Gammaproteobacteria; o__Enterobacteriales; f__Enterobacteriaceae	k__Bacteria; p__Proteobacteria; c__Gammaproteobacteria; o__Enterobacteriales; f__Enterobacteriaceae; g__*Arsenophonus*	*Arsenophonus nasoniae* strain ATCC 49151 16S ribosomal RNA gene, partial sequence	98.91
OTU1277	458	k__Bacteria; p__Proteobacteria; c__Gammaproteobacteria; o__Enterobacteriales; f__Enterobacteriaceae	k__Bacteria; p__Proteobacteria; c__Gammaproteobacteria; o__Enterobacteriales; f__Enterobacteriaceae; g__*Enterobacter*	*Enterobacter kobei* strain JCM 8580 16S ribosomal RNA gene, partial sequence	99.78
OTU1193	459	k__Bacteria; p__Proteobacteria; c__Gammaproteobacteria; o__Pseudomonadales; f__Moraxellaceae; g__*Acinetobacter*;	k__Bacteria; p__Proteobacteria; c__Gammaproteobacteria; o__Pseudomonadales; f__Moraxellaceae; g__*Acinetobacter*	*Acinetobacter baumannii* ATCC 17978 strain ATCC 17978 16S ribosomal RNA, complete sequence	100
OTU826	457	k__Bacteria; p__Tenericutes; c__Mollicutes; o__Entomoplasmatales; f__Entomoplasmataceae	k__Bacteria; p__Tenericutes; c__Mollicutes; o__Entomoplasmatales; f__Entomoplasmataceae; g__*Mesoplasma*	*Entomoplasma luminosum* strain PIMN-1 16S ribosomal RNA gene, partial sequence	93.87
OTU1176	322	Unassigned	Unassigned	*Wolbachia* sp. wRi strain wRi 16S ribosomal RNA, complete sequence	91.55

Supplementary Table S4 Taxonomic identification of OTUs higher than 0.5% of total abundance with reference to Greengene 13_8, SILVA 128 databases and their top BLASTn hit (k, kingdom; p, phylum; c, class; o, order; f, family; g, genus).

Anova: Single Factor

SUMMARY

Groups	Count	Sum	Average	Variance
Forest	4	354.8945	88.72363499	43.65966
Green area in urban city	4	308.8312	77.20780666	141.9646
Urban city	4	190.2451	47.56128151	639.2791

ANOVA

Source of Variation	SS	df	MS	F	P-value	F crit
Between Groups	3607.827	2	1803.9134	6.560453	0.017477	4.256495
Within Groups	2474.71	9	274.967792			
Total	6082.537	11				

F > F crit, reject null hypothesis

Supplementary Table S5 ANOVA analysis on abundance level of family Acetobacteraceae in *O. smaragdina* colonies from different environments.

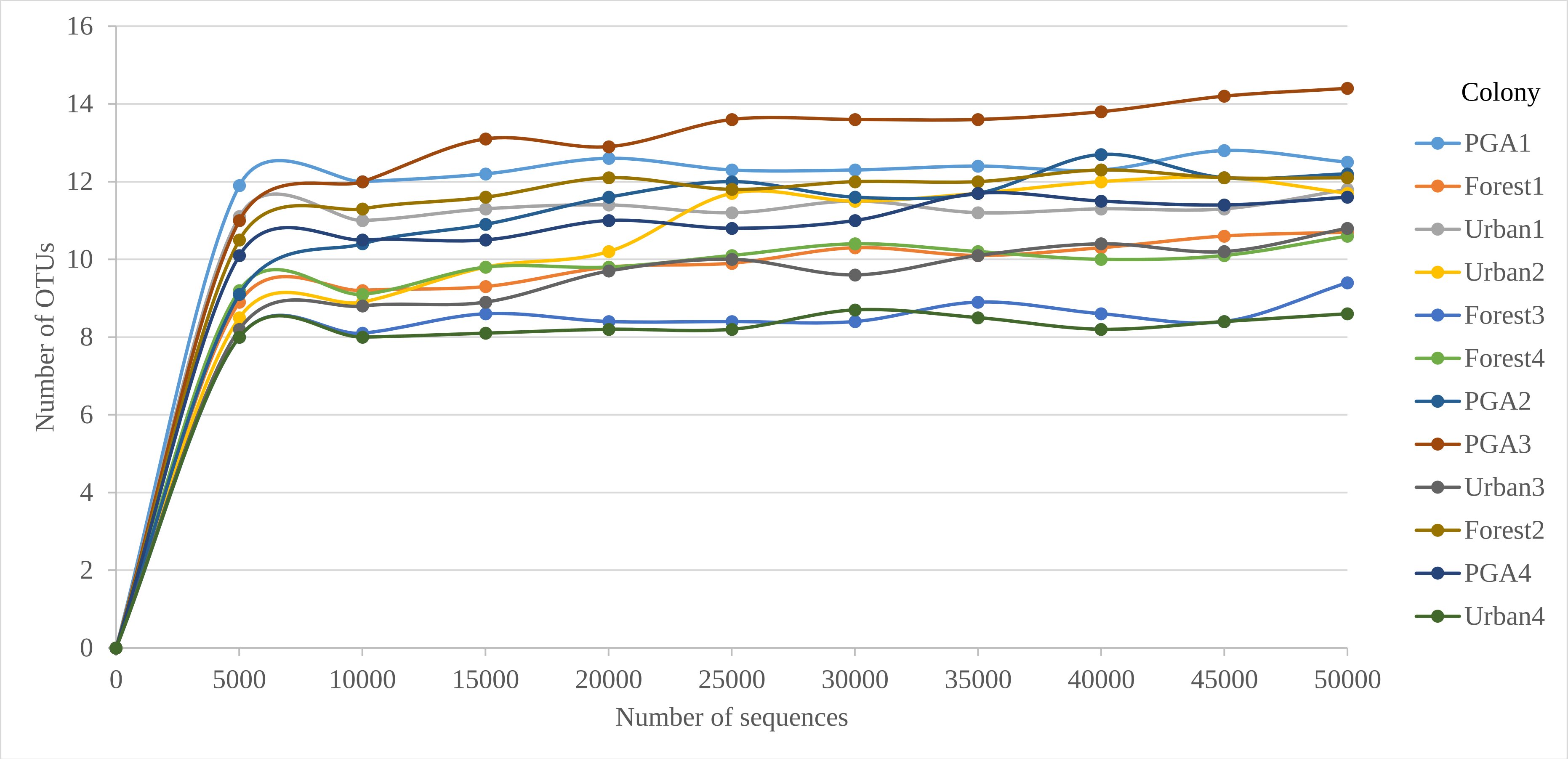

Supplementary Fig. S1 Rarefaction curves of OTUs clustered at 97% similarity for each *O. smaragdina* sample.

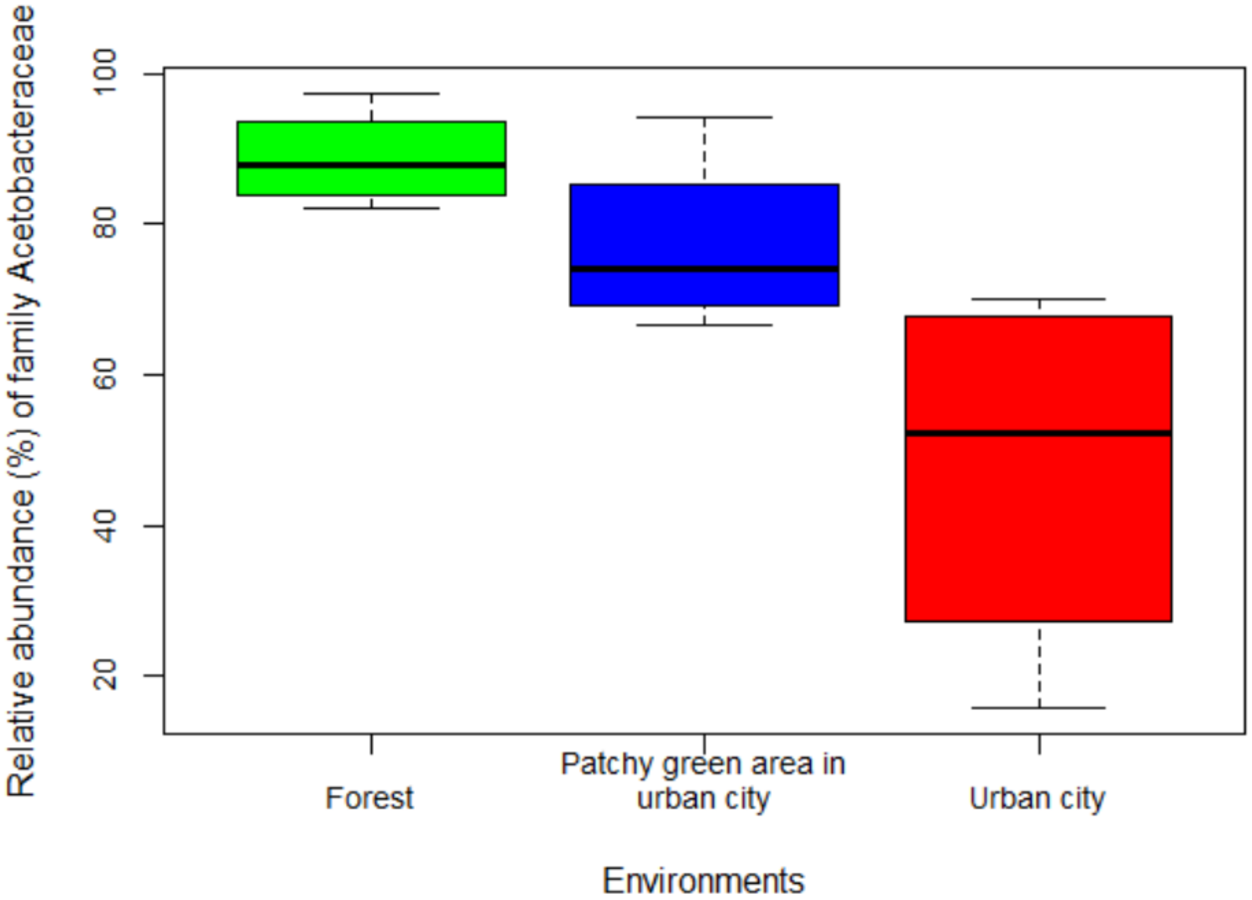

Supplementary Fig. S2 Box-whisker plots show the relative abundance (%) of family Acetobacteraceae in *O. smaragdina* samples from different environments.

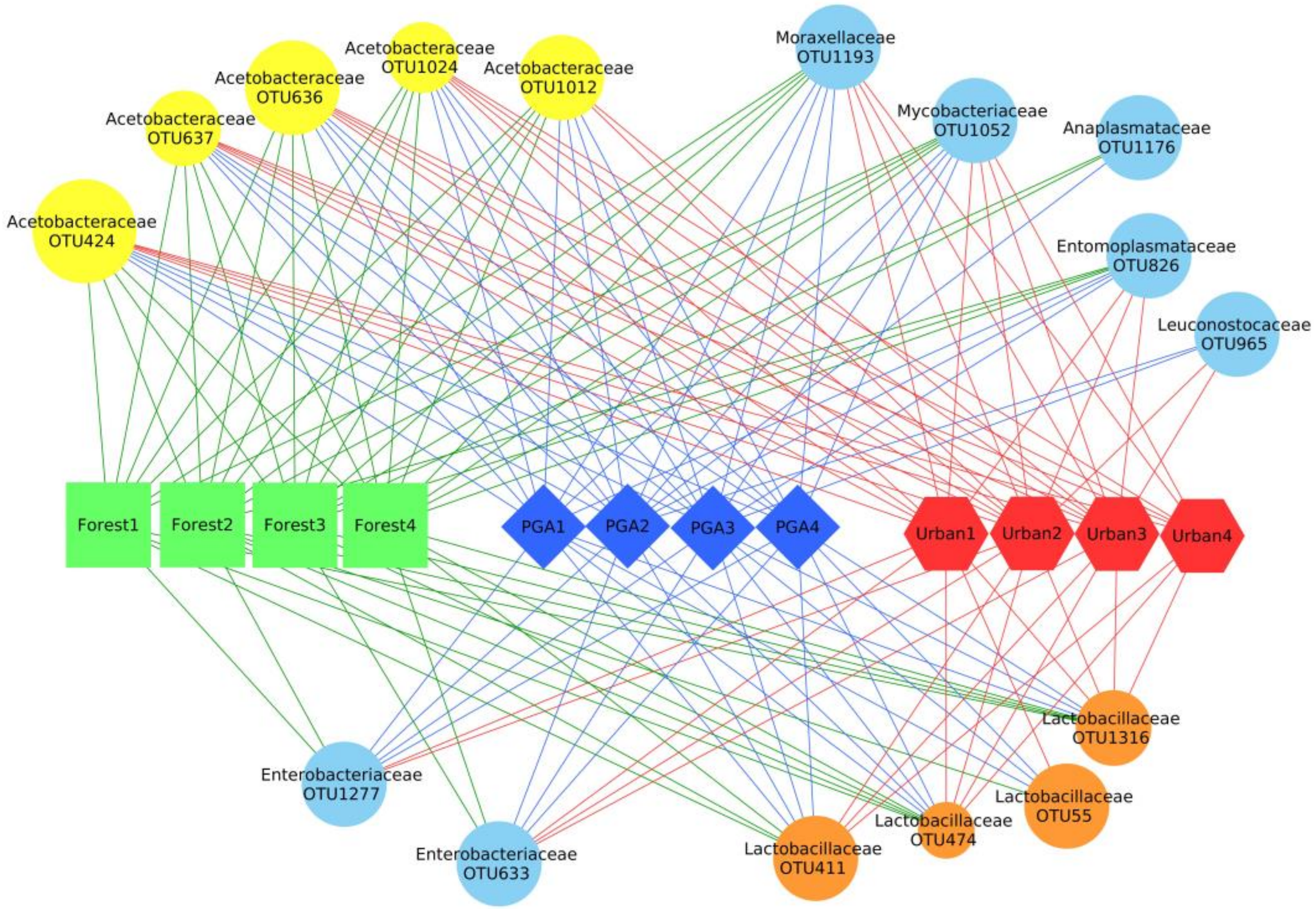

Supplementary Fig. S3 OTU network map showing interactions between OTUs and all *O. smaragdina* colonies from different environments. The OTUs of dominant families Acetobacteraceae and Lactobacillaceae were coloured in yellow and orange respectively, while the rest of the OTUs in light blue. The samples from forest (Forest1 to Forest4) were labelled in green squares, samples from patchy green area of urban city (PGA1 to PGA4) were labelled in blue diamonds while samples from urban area (Urban1 to Urban4) were labelled in red hexagons. The lines connecting OTUs to samples link the OTUs to their source microbiome (green lines for forest group samples, the blue lines for samples from patchy green areas of urban city and red lines for samples from urban areas).

Supplementary Table S3 Lists of all OTUs and their UCLUST-assigned taxonomy with references to Green genes 13_8 database and SILVA128 database (k, kingdom; p, phylum; c, class; o, order; f, family; g, genus).

OTUs	Taxonomy assigned with reference to Green gene 13_8 database using UCLUST	Taxonomy assigned with reference to SILVA128 database using UCLUST
OTU0	k__Bacteria; p__Proteobacteria; c__Alphaproteobacteria; o__Rhizobiales	k__Bacteria;p__Proteobacteria;c__Alphaproteobacteria;o__Rhizobiales;f__Methylobacteriaceae
OTU1	k__Bacteria; p__Cyanobacteria; c__; o__; f__; g__; s__	k__Bacteria;p__Cyanobacteria;c__Cyanobacteria;o__SubsectionIII;f__FamilyI;g__Phormidium;Ambiguous_taxa
OTU10	k__Bacteria; p__Proteobacteria; c__Alphaproteobacteria; o__Sphingomonadales; f__Sphingomonadaceae	k__Bacteria;p__Proteobacteria;c__Alphaproteobacteria;o__Sphingomonadales;f__Sphingomonadaceae;g__Novosphingobium
OTU100	k__Bacteria; p__Proteobacteria; c__Alphaproteobacteria; o__Rhodospirillales; f__Acetobacteraceae; g__; s__	k__Bacteria;p__Proteobacteria;c__Alphaproteobacteria;o__Rhodospirillales;f__Acetobacteraceae;g__uncultured;s__uncultured bacterium
OTU1000	k__Bacteria; p__Proteobacteria; c__Alphaproteobacteria; o__Rickettsiales; f__mitochondria	k__Bacteria;p__Proteobacteria;c__Alphaproteobacteria;o__Rickettsiales;f__Mitochondria;Ambiguous_taxa;Ambiguous_taxa
OTU1001	k__Bacteria; p__Proteobacteria; c__Deltaproteobacteria; o__Myxococcales; f__; g__; s__	k__Bacteria;p__Proteobacteria;c__Deltaproteobacteria;o__Myxococcales;f__BIrii41;g__uncultured bacterium;s__uncultured bacterium
OTU1002	k__Bacteria; p__Proteobacteria; c__Deltaproteobacteria; o__Syntrophobacterales; f__Syntrophobacteraceae; g__; s__	k__Bacteria;p__Proteobacteria;c__Deltaproteobacteria;o__Desulfurellales;f__Desulfurellaceae;g__H16;s__uncultured bacterium
OTU1003	k__Bacteria; p__Actinobacteria; c__Acidimicrobiia; o__Acidimicrobiales; f__; g__; s__	k__Bacteria;p__Actinobacteria;c__Acidimicrobiia;o__Acidimicrobiales;f__uncultured;g__uncultured bacterium;s__uncultured bacterium
OTU1004	k__Bacteria; p__Proteobacteria; c__Alphaproteobacteria; o__Rhodospirillales; f__Acetobacteraceae	k__Bacteria;p__Proteobacteria;c__Alphaproteobacteria;o__Rhodospirillales;f__Acetobacteraceae
OTU1005	Unassigned	Unassigned
OTU1006	k__Bacteria; p__Actinobacteria; c__Actinobacteria; o__Actinomycetales; f__Dietziaceae; g__Dietzia; s__	k__Bacteria;p__Actinobacteria;c__Actinobacteria;o__Corynebacteriales;f__Dietziaceae;g__Dietzia;Ambiguous_taxa
OTU1007	k__Bacteria; p__Cyanobacteria; c__Oscillatoriophycideae; o__Chroococcales; f__Xenococcaceae; g__; s__	k__Bacteria;p__Cyanobacteria;c__Cyanobacteria;o__SubsectionII;f__FamilyII;g__Chroococcidiopsis
OTU1008	k__Bacteria; p__Proteobacteria; c__Betaproteobacteria; o__Burkholderiales; f__Oxalobacteraceae; g__; s__	k__Bacteria;p__Proteobacteria;c__Betaproteobacteria;o__Burkholderiales;f__Oxalobacteraceae
OTU1009	k__Bacteria; p__Planctomycetes; c__Phycisphaerae; o__WD2101; f__; g__; s__	k__Bacteria;p__Planctomycetes;c__Phycisphaerae;o__Tepidisphaerales;f__Tepidisphaeraceae;g__uncultured bacterium;s__uncultured bacterium
OTU101	k__Bacteria; p__Proteobacteria; c__Alphaproteobacteria; o__Sphingomonadales; f__Sphingomonadaceae; g__; s__	k__Bacteria;p__Proteobacteria;c__Alphaproteobacteria;o__Sphingomonadales;f__Sphingomonadaceae;g__Novosphingobium
OTU1010	k__Bacteria; p__Proteobacteria; c__Alphaproteobacteria; o__Rhodospirillales; f__Acetobacteraceae	k__Bacteria;p__Proteobacteria;c__Alphaproteobacteria;o__Rhodospirillales;f__Acetobacteraceae
OTU1011	k__Bacteria; p__Acidobacteria; c__Solibacteres; o__Solibacterales; f__Solibacteraceae; g__; s__	k__Bacteria;p__Acidobacteria;c__Solibacteres;o__Solibacterales;f__Solibacteraceae (Subgroup 3);g__Bryobacter;Ambiguous_taxa
OTU1012	k__Bacteria; p__Proteobacteria; c__Alphaproteobacteria; o__Rhodospirillales; f__Acetobacteraceae	k__Bacteria;p__Proteobacteria;c__Alphaproteobacteria;o__Rhodospirillales;f__Acetobacteraceae
OTU1013	k__Bacteria; p__Proteobacteria; c__Alphaproteobacteria; o__Rhizobiales; f__Bradyrhizobiaceae; g__; s__	k__Bacteria;p__Proteobacteria;c__Alphaproteobacteria;o__Rhizobiales;f__Bradyrhizobiaceae;g__Bosea

OTU1014	k__Bacteria; p__Proteobacteria; c__Alphaproteobacteria; o__Rhizobiales; f__Aurantimonadaceae; g__; s__	k__Bacteria;p__Proteobacteria;c__Alphaproteobacteria;o__Rhizobiales;f__Aurantimonadaceae;g__Aureimonas;s__uncultured bacterium
OTU1015	Unassigned	Unassigned
OTU1016	k__Bacteria; p__Cyanobacteria; c__Oscillatoriophycideae; o__Chroococcales; f__Xenococcaceae; g__; s__	k__Bacteria;p__Cyanobacteria;c__Cyanobacteria;o__SubsectionII;f__FamilyII;g__Chroococcidiopsis;s__uncultured bacterium
OTU1017	k__Bacteria; p__Proteobacteria; c__Gammaproteobacteria; o__Enterobacteriales; f__Enterobacteriaceae; g__; s__	k__Bacteria;p__Proteobacteria;c__Gammaproteobacteria;o__Enterobacteriales;f__Enterobacteriaceae;g__Raoultella
OTU1018	Unassigned	Unassigned
OTU1019	Unassigned	Unassigned
OTU102	k__Bacteria; p__Bacteroidetes; c__Cytophagia; o__Cytophagales; f__Cytophagaceae; g__Spirosoma; s__	k__Bacteria;p__Bacteroidetes;c__Cytophagia;o__Cytophagales;f__Cytophagaceae;g__uncultured;s__uncultured bacterium
OTU1020	k__Bacteria; p__Proteobacteria; c__Alphaproteobacteria; o__Rhodospirillales; f__Acetobacteraceae; g__; s__	k__Bacteria;p__Proteobacteria;c__Alphaproteobacteria;o__Rhodospirillales;f__Acetobacteraceae;g__Acidiphilium
OTU1021	Unassigned	k__Bacteria;p__Actinobacteria;c__Actinobacteria;o__Corynebacteriales;f__nbr16a11;g__uncultured bacterium;s__uncultured bacterium
OTU1022	k__Bacteria; p__Verrucomicrobia; c__[Spartobacteria]; o__[Chthoniobacterales]; f__[Chthoniobacteraceae]; g__; s__	k__Bacteria;p__Verrucomicrobia;c__Spartobacteria;o__Chthoniobacterales;f__LD29;g__uncultured bacterium;s__uncultured bacterium
OTU1023	k__Bacteria; p__Cyanobacteria; c__Synechococcophycideae; o__Pseudanabaenales; f__Pseudanabaenaceae	k__Bacteria;p__Cyanobacteria;c__Cyanobacteria;o__SubsectionIII;f__FamilyI;g__Leptolyngbya;Ambiguous_taxa
OTU1024	k__Bacteria; p__Proteobacteria; c__Alphaproteobacteria; o__Rhodospirillales; f__Acetobacteraceae	k__Bacteria;p__Proteobacteria;c__Alphaproteobacteria;o__Rhodospirillales;f__Acetobacteraceae;g__uncultured;s__uncultured bacterium
OTU1025	k__Bacteria; p__Acidobacteria; c__Solibacteres; o__Solibacterales; f__Solibacteraceae; g__Candidatus Solibacter; s__	k__Bacteria;p__Acidobacteria;c__Solibacteres;o__Solibacterales;f__Solibacteraceae (Subgroup 3);g__Candidatus Solibacter
OTU1026	Unassigned	Unassigned
OTU1027	k__Bacteria; p__Proteobacteria; c__Alphaproteobacteria; o__Sphingomonadales; f__Sphingomonadaceae; g__Sphingomonas; s__	k__Bacteria;p__Proteobacteria;c__Alphaproteobacteria;o__Sphingomonadales;f__Sphingomonadaceae;g__Sphingomonas;s__uncultured bacterium
OTU1028	k__Bacteria; p__Proteobacteria; c__Gammaproteobacteria; o__Pseudomonadales; f__Moraxellaceae; g__Acinetobacter; s__	k__Bacteria;p__Proteobacteria;c__Gammaproteobacteria;o__Pseudomonadales;f__Moraxellaceae;g__Acinetobacter;s__Acinetobacter baumannii
OTU1029	k__Bacteria; p__Firmicutes; c__Bacilli; o__Bacillales; f__Bacillaceae; g__; s__	k__Bacteria;p__Firmicutes;c__Bacilli;o__Bacillales;f__Bacillaceae
OTU103	k__Bacteria; p__Proteobacteria; c__Alphaproteobacteria; o__Rhizobiales; f__; g__; s__	k__Bacteria;p__Proteobacteria;c__Alphaproteobacteria;o__Rhizobiales;f__Bradyrhizobiaceae;g__Bosea;Ambiguous_taxa
OTU1030	k__Bacteria; p__Proteobacteria; c__Alphaproteobacteria; o__Rhizobiales; f__Methylocystaceae; g__; s__	k__Bacteria;p__Proteobacteria;c__Alphaproteobacteria;o__Rhizobiales;f__Rhizobiales Incertae Sedis;g__Agaricicola;Ambiguous_taxa
OTU1031	k__Bacteria; p__Actinobacteria; c__Thermoleophilia; o__Solirubrobacterales; f__Patulibacteraceae; g__; s__	k__Bacteria;p__Actinobacteria;c__Thermoleophilia;o__Solirubrobacterales;f__Gsoil-1167;g__uncultured bacterium;s__uncultured bacterium
OTU1032	k__Bacteria; p__Proteobacteria; c__Alphaproteobacteria; o__Sphingomonadales; f__Sphingomonadaceae; g__Sphingomonas; s__	k__Bacteria;p__Proteobacteria;c__Alphaproteobacteria;o__Sphingomonadales;f__Sphingomonadaceae;g__Sphingomonas;Ambiguous_taxa
OTU1033	Unassigned	Unassigned
OTU1034	k__Bacteria; p__FBP; c__; o__; f__; g__; s__	k__Bacteria;p__FBP;c__uncultured bacterium;o__uncultured bacterium;f__uncultured bacterium;g__uncultured bacterium;s__uncultured bacterium

OTU1035	k__Bacteria; p__Proteobacteria; c__Alphaproteobacteria; o__Sphingomonadales; f__Sphingomonadaceae; g__Sphingobium; s__	k__Bacteria;p__Proteobacteria;c__Alphaproteobacteria;o__Sphingomonadales;f__Sphingomonadaceae;g__Sphingobium;Ambiguous_taxa
OTU1036	k__Bacteria; p__Planctomycetes; c__Planctomycetia; o__Pirellulales; f__Pirellulaceae; g__; s__	k__Bacteria;p__Planctomycetes;c__Planctomycetacia;o__Planctomycetales;f__Planctomycetaceae;g__uncultured;s__uncultured bacterium
OTU1037	Unassigned	Unassigned
OTU1038	Unassigned	k__Bacteria;p__Proteobacteria;c__Alphaproteobacteria;o__Rhodospirillales;f__Acetobacteraceae;g__uncultured;s__uncultured bacterium
OTU1039	Unassigned	Unassigned
OTU104	k__Bacteria; p__Proteobacteria; c__Gammaproteobacteria; o__Enterobacteriales; f__Enterobacteriaceae	k__Bacteria;p__Proteobacteria;c__Gammaproteobacteria;o__Enterobacteriales;f__Enterobacteriaceae;g__Arsenophonus
OTU1040	Unassigned	Unassigned
OTU1041	k__Bacteria; p__Cyanobacteria; c__Oscillatoriophycideae; o__Oscillatoriales; f__Phormidiaceae; g__Phormidium; s__	k__Bacteria;p__Cyanobacteria;c__Cyanobacteria;o__SubsectionIII;f__FamilyI;g__Microcoleus
OTU1042	k__Bacteria; p__Bacteroidetes; c__Sphingobacteriia; o__Sphingobacteriales; f__Sphingobacteriaceae; g__; s__	k__Bacteria;p__Bacteroidetes;c__Sphingobacteriia;o__Sphingobacteriales;f__Sphingobacteriaceae;g__Mucilaginibacter;s__uncultured bacterium
OTU1043	k__Bacteria; p__Gemmatimonadetes; c__Gemm-3; o__; f__; g__; s__	k__Bacteria;p__Gemmatimonadetes;c__Longimicrobia;o__Longimicrobiales;f__Longimicrobiaceae;g__uncultured bacterium;s__uncultured bacterium
OTU1044	k__Bacteria; p__Proteobacteria; c__Alphaproteobacteria; o__Rhizobiales; f__Methylocystaceae; g__; s__	k__Bacteria;p__Proteobacteria;c__Alphaproteobacteria;o__Rhizobiales;f__1174-901-12;g__uncultured bacterium;s__uncultured bacterium
OTU1045	Unassigned	Unassigned
OTU1046	k__Bacteria; p__Bacteroidetes; c__Sphingobacteriia; o__Sphingobacteriales; f__Sphingobacteriaceae; g__; s__	k__Bacteria;p__Bacteroidetes;c__Sphingobacteriia;o__Sphingobacteriales;f__Sphingobacteriaceae;g__Mucilaginibacter;s__uncultured bacterium
OTU1047	k__Bacteria; p__Proteobacteria; c__Gammaproteobacteria; o__Xanthomonadales; f__Xanthomonadaceae; g__Dokdonella; s__	k__Bacteria;p__Proteobacteria;c__Gammaproteobacteria;o__Xanthomonadales;f__Xanthomonadaceae;g__Dokdonella;Ambiguous_taxa
OTU1048	k__Bacteria; p__Chloroflexi; c__Chloroflexi; o__Chloroflexales; f__Chloroflexaceae; g__; s__	k__Bacteria;p__Chloroflexi;c__Chloroflexia;o__Chloroflexales;f__Chloroflexaceae;g__Chloroflexus;s__uncultured bacterium
OTU1049	k__Bacteria; p__Actinobacteria; c__Actinobacteria; o__Actinomycetales; f__Nocardioidaceae; g__Propionicimonas; s__	k__Bacteria;p__Actinobacteria;c__Actinobacteria;o__Propionibacteriales;f__Propionibacteriaceae
OTU105	k__Bacteria; p__Proteobacteria; c__Gammaproteobacteria; o__Pseudomonadales; f__Moraxellaceae; g__Acinetobacter; s__	k__Bacteria;p__Proteobacteria;c__Gammaproteobacteria;o__Pseudomonadales;f__Moraxellaceae;g__Acinetobacter;s__uncultured bacterium
OTU1050	k__Bacteria; p__Proteobacteria; c__Alphaproteobacteria; o__Sphingomonadales; f__Sphingomonadaceae; g__; s__	k__Bacteria;p__Proteobacteria;c__Alphaproteobacteria;o__Sphingomonadales
OTU1051	k__Bacteria; p__Cyanobacteria; c__Oscillatoriophycideae; o__Oscillatoriales; f__Phormidiaceae; g__Phormidium	k__Bacteria;p__Cyanobacteria;c__Cyanobacteria;o__SubsectionIII;f__FamilyI;g__Phormidium;s__uncultured bacterium
OTU1052	k__Bacteria; p__Actinobacteria; c__Actinobacteria; o__Actinomycetales; f__Mycobacteriaceae; g__Mycobacterium; s__	k__Bacteria;p__Actinobacteria;c__Actinobacteria;o__Corynebacteriales;f__nbr16a11;g__uncultured bacterium;s__uncultured bacterium
OTU1053	k__Bacteria; p__Proteobacteria; c__Alphaproteobacteria; o__Rhodospirillales; f__Acetobacteraceae; g__; s__	k__Bacteria;p__Proteobacteria;c__Alphaproteobacteria;o__Rhodospirillales;f__Acetobacteraceae
OTU1054	k__Bacteria; p__Actinobacteria; c__Actinobacteria; o__Actinomycetales; f__Corynebacteriaceae; g__Corynebacterium; s__	k__Bacteria;p__Actinobacteria;c__Actinobacteria;o__Corynebacteriales;f__Corynebacteriaceae;g__Corynebacterium 1;s__uncultured bacterium
OTU1055	k__Bacteria; p__Acidobacteria; c__Solibacteres; o__Solibacterales; f__Solibacteraceae; g__; s__	k__Bacteria;p__Acidobacteria;c__Solibacteres;o__Solibacterales;f__Solibacteraceae (Subgroup 3);g__Bryobacter;s__uncultured bacterium

OTU1056	k__Bacteria; p__Proteobacteria; c__Deltaproteobacteria; o__Myxococcales; f__Haliangiaceae; g__; s__	k__Bacteria;p__Proteobacteria;c__Deltaproteobacteria;o__Myxococcales;f__Haliangiaceae;g__Haliangium;s__uncultured bacterium
OTU1057	k__Bacteria; p__Actinobacteria; c__Actinobacteria; o__Actinomycetales; f__Mycobacteriaceae; g__Mycobacterium; s__	k__Bacteria;p__Actinobacteria;c__Actinobacteria;o__Corynebacteriales;f__Mycobacteriaceae;g__Mycobacterium;Ambiguous_taxa
OTU1058	Unassigned	Unassigned
OTU1059	Unassigned	Unassigned
OTU106	Unassigned	Unassigned
OTU1060	k__Bacteria; p__Proteobacteria; c__Alphaproteobacteria; o__Rhizobiales; f__Hyphomicrobiaceae; g__Devosia; s__	k__Bacteria;p__Proteobacteria;c__Alphaproteobacteria;o__Rhizobiales;f__Hyphomicrobiaceae;g__Devosia;Ambiguous_taxa
OTU1061	k__Bacteria; p__Actinobacteria; c__Actinobacteria; o__Actinomycetales; f__Nocardioidaceae; g__; s__	k__Bacteria;p__Actinobacteria;c__Actinobacteria;o__Propionibacteriales;f__Nocardioidaceae;g__Nocardioides;s__uncultured bacterium
OTU1062	Unassigned	Unassigned
OTU1063	k__Bacteria; p__Cyanobacteria; c__Oscillatoriophycideae; o__Oscillatoriales; f__Phormidiaceae; g__Phormidium; s__	k__Bacteria;p__Cyanobacteria;c__Cyanobacteria;o__SubsectionIII;f__FamilyI;g__uncultured
OTU1064	k__Bacteria; p__TM7; c__TM7-3; o__; f__; g__; s__	k__Bacteria;p__Saccharibacteria;c__uncultured bacterium;o__uncultured bacterium;f__uncultured bacterium;g__uncultured bacterium;s__uncultured bacterium
OTU1065	k__Bacteria; p__TM7; c__TM7-3; o__; f__; g__; s__	k__Bacteria;p__Saccharibacteria;c__uncultured bacterium;o__uncultured bacterium;f__uncultured bacterium;g__uncultured bacterium;s__uncultured bacterium
OTU1066	k__Bacteria; p__Planctomycetes; c__Phycisphaerae; o__WD2101; f__; g__; s__	k__Bacteria;p__Planctomycetes;c__Phycisphaerae;o__Tepidisphaerales;f__Tepidisphaeraceae
OTU1067	Unassigned	k__Bacteria;p__Bacteroidetes;c__Cytophagia;o__Cytophagales;f__Cytophagaceae;g__Hymenobacter;s__uncultured bacterium
OTU1068	k__Bacteria; p__Proteobacteria; c__Gammaproteobacteria; o__Legionellales; f__Legionellaceae	k__Bacteria;p__Proteobacteria;c__Gammaproteobacteria;o__Legionellales;f__Legionellaceae;g__Legionella
OTU1069	k__Bacteria; p__Actinobacteria; c__Acidimicrobiia; o__Acidimicrobiales; f__; g__; s__	k__Bacteria;p__Actinobacteria;c__Acidimicrobiia;o__Acidimicrobiales;f__uncultured;g__uncultured bacterium;s__uncultured bacterium
OTU107	k__Bacteria; p__Proteobacteria; c__Alphaproteobacteria; o__Sphingomonadales; f__Sphingomonadaceae; g__; s__	k__Bacteria;p__Proteobacteria;c__Alphaproteobacteria;o__Sphingomonadales;f__Sphingomonadaceae
OTU1070	k__Bacteria; p__Proteobacteria; c__Alphaproteobacteria; o__Sphingomonadales; f__Sphingomonadaceae; g__Sphingomonas; s__	k__Bacteria;p__Proteobacteria;c__Alphaproteobacteria;o__Sphingomonadales;f__Sphingomonadaceae;g__Sphingomonas
OTU1071	k__Bacteria; p__Cyanobacteria; c__Synechococcophycideae; o__Pseudanabaenales; f__Pseudanabaenaceae; g__Leptolyngbya; s__	k__Bacteria;p__Cyanobacteria;c__Cyanobacteria;o__SubsectionIII;f__FamilyI;g__Leptolyngbya;s__uncultured bacterium
OTU1072	k__Bacteria; p__Proteobacteria; c__Alphaproteobacteria; o__Rhodobacterales; f__Rhodobacteraceae; g__Rubellimicrobium; s__	k__Bacteria;p__Proteobacteria;c__Alphaproteobacteria;o__Rhodobacterales;f__Rhodobacteraceae;g__Rubellimicrobium;s__uncultured bacterium
OTU1073	Unassigned	Unassigned
OTU1074	k__Bacteria; p__Proteobacteria; c__Alphaproteobacteria; o__Caulobacterales; f__Caulobacteraceae; g__; s__	k__Bacteria;p__Proteobacteria;c__Alphaproteobacteria;o__Caulobacterales;f__Caulobacteraceae;g__uncultured;Ambiguous_taxa
OTU1075	k__Bacteria; p__Proteobacteria; c__Alphaproteobacteria; o__Rhodospirillales; f__Acetobacteraceae; g__Gluconobacter; s__	k__Bacteria;p__Proteobacteria;c__Alphaproteobacteria;o__Rhodospirillales;f__Acetobacteraceae

OTU1076	k__Bacteria; p__Actinobacteria; c__Actinobacteria; o__Actinomycetales; f__Cellulomonadaceae; g__Cellulomonas; s__	k__Bacteria;p__Actinobacteria;c__Actinobacteria;o__Micrococcales;f__Cellulomonadaceae;g__Cellulomonas;Ambiguous_taxa
OTU1077	Unassigned	k__Bacteria;p__Chloroflexi;c__Chloroflexia;o__Chloroflexales;f__Roseiflexaceae;g__Roseiflexus;s__uncultured bacterium
OTU1078	k__Bacteria; p__Acidobacteria; c__S035; o__; f__; g__; s__	k__Bacteria;p__Acidobacteria;c__Subgroup 25;o__uncultured bacterium;f__uncultured bacterium;g__uncultured bacterium;s__uncultured bacterium
OTU1079	Unassigned	Unassigned
OTU108	k__Bacteria; p__Proteobacteria; c__Alphaproteobacteria; o__Sphingomonadales; f__Sphingomonadaceae; g__Sphingomonas; s__	k__Bacteria;p__Proteobacteria;c__Alphaproteobacteria;o__Sphingomonadales;f__Sphingomonadaceae;g__Sphingomonas
OTU1080	k__Bacteria; p__Gemmatimonadetes; c__Gemmatimonadetes; o__Gemmatimonadales; f__; g__; s__	k__Bacteria;p__Gemmatimonadetes;c__Gemmatimonadetes;o__Gemmatimonadales;f__Gemmatimonadaceae;g__Gemmatirosa;s__uncultured bacterium
OTU1081	k__Bacteria; p__Acidobacteria; c__[Chloracidobacteria]; o__PK29; f__; g__; s__	k__Bacteria;p__Acidobacteria;c__Blastocatellia;o__Blastocatellales;f__Blastocatellaceae (Subgroup 4);g__11-24;s__uncultured bacterium
OTU1082	k__Bacteria; p__Proteobacteria; c__Alphaproteobacteria; o__Sphingomonadales; f__Sphingomonadaceae; g__Sphingomonas; s__wittichii	k__Bacteria;p__Proteobacteria;c__Alphaproteobacteria;o__Sphingomonadales;f__Sphingomonadaceae;g__Sphingomonas;Ambiguous_taxa
OTU1083	k__Bacteria; p__Actinobacteria; c__Actinobacteria; o__Bifidobacteriales; f__Bifidobacteriaceae; g__Bifidobacterium; s__longum	k__Bacteria;p__Actinobacteria;c__Actinobacteria;o__Bifidobacteriales;f__Bifidobacteriaceae;g__Bifidobacterium
OTU1084	Unassigned	Unassigned
OTU1085	k__Bacteria; p__Acidobacteria; c__Acidobacteria-6; o__iii1-15; f__; g__; s__	k__Bacteria;p__Acidobacteria;c__Subgroup 6;o__uncultured Acidobacteria bacterium;f__uncultured Acidobacteria bacterium;g__uncultured Acidobacteria bacterium;s__uncultured Acidobacteria bacterium
OTU1086	k__Bacteria; p__Actinobacteria; c__Actinobacteria; o__Actinomycetales; f__Williamsiaceae; g__Williamsia; s__	k__Bacteria;p__Actinobacteria;c__Actinobacteria;o__Corynebacteriales;f__Nocardiaceae;g__Williamsia;Ambiguous_taxa
OTU1087	Unassigned	Unassigned
OTU1088	k__Bacteria; p__Chloroflexi; c__Chloroflexi; o__Chloroflexales; f__Chloroflexaceae; g__Chloronema; s__	k__Bacteria;p__Chloroflexi;c__Chloroflexia;o__Chloroflexales;f__Chloroflexaceae
OTU1089	k__Bacteria; p__Bacteroidetes; c__Flavobacteriia; o__Flavobacteriales; f__[Weeksellaceae]; g__Chryseobacterium; s__	k__Bacteria;p__Bacteroidetes;c__Flavobacteriia;o__Flavobacteriales;f__Flavobacteriaceae;g__Chryseobacterium;Ambiguous_taxa
OTU109	Unassigned	k__Bacteria;p__Proteobacteria;c__Alphaproteobacteria;o__Rhodospirillales;f__Acetobacteraceae;g__uncultured;s__uncultured bacterium
OTU1090	k__Bacteria; p__Proteobacteria; c__Alphaproteobacteria; o__Rhizobiales; f__Bradyrhizobiaceae; g__; s__	k__Bacteria;p__Proteobacteria;c__Alphaproteobacteria;o__Rhizobiales;f__Methylobacteriaceae;g__uncultured;s__uncultured bacterium
OTU1091	k__Bacteria; p__Proteobacteria; c__Betaproteobacteria; o__A21b; f__UD5; g__; s__	k__Bacteria;p__Proteobacteria;c__Betaproteobacteria;o__SC-I-84
OTU1092	k__Bacteria; p__Proteobacteria; c__Betaproteobacteria; o__SC-I-84; f__; g__; s__	k__Bacteria;p__Proteobacteria;c__Betaproteobacteria;o__SC-I-84;f__uncultured bacterium;g__uncultured bacterium;s__uncultured bacterium
OTU1093	k__Bacteria; p__Planctomycetes; c__Phycisphaerae; o__WD2101; f__; g__; s__	k__Bacteria;p__Planctomycetes;c__Phycisphaerae;o__Tepidisphaerales;f__Tepidisphaeraceae;g__uncultured bacterium;s__uncultured bacterium
OTU1094	k__Bacteria; p__Actinobacteria; c__Actinobacteria; o__Actinomycetales; f__Nocardioidaceae; g__Nocardioides; s__	k__Bacteria;p__Actinobacteria;c__Actinobacteria;o__Propionibacteriales;f__Nocardioidaceae;g__Nocardioides;s__uncultured bacterium

OTU1095	k__Bacteria; p__Actinobacteria; c__Actinobacteria; o__Actinomycetales; f__Mycobacteriaceae; g__Mycobacterium; s__	k__Bacteria;p__Actinobacteria;c__Actinobacteria;o__Corynebacteriales;f__Mycobacteriaceae;g__Mycobacterium
OTU1096	Unassigned	Unassigned
OTU1097	k__Bacteria; p__Proteobacteria; c__Deltaproteobacteria; o__Spirobacillales; f__; g__; s__	k__Bacteria;p__Proteobacteria;c__Deltaproteobacteria;o__Oligoflexales;f__Oligoflexaceae;g__uncultured bacterium;s__uncultured bacterium
OTU1098	k__Bacteria; p__Proteobacteria; c__Alphaproteobacteria; o__Caulobacterales; f__Caulobacteraceae; g__Phenylobacterium; s__	k__Bacteria;p__Proteobacteria;c__Alphaproteobacteria;o__Caulobacterales;f__Caulobacteraceae;g__Phenylobacterium;s__uncultured bacterium
OTU1099	k__Bacteria; p__Proteobacteria; c__Gammaproteobacteria; o__Pseudomonadales; f__Moraxellaceae; g__Acinetobacter; s__	k__Bacteria;p__Proteobacteria;c__Gammaproteobacteria;o__Pseudomonadales;f__Moraxellaceae;g__Acinetobacter;Ambiguous_taxa
OTU11	k__Bacteria; p__Verrucomicrobia; c__[Spartobacteria]; o__[Chthoniobacterales]; f__[Chthoniobacteraceae]; g__; s__	k__Bacteria;p__Verrucomicrobia;c__Spartobacteria;o__Chthoniobacterales;f__LD29;g__uncultured bacterium;s__uncultured bacterium
OTU110	k__Bacteria; p__Acidobacteria; c__Acidobacteria-6; o__iii1-15; f__; g__; s__	k__Bacteria;p__Acidobacteria;c__Subgroup 6;o__uncultured bacterium;f__uncultured bacterium;g__uncultured bacterium;s__uncultured bacterium
OTU1100	k__Bacteria; p__Proteobacteria; c__Alphaproteobacteria; o__Rhizobiales; f__Bradyrhizobiaceae; g__Bosea; s__genosp.	k__Bacteria;p__Proteobacteria;c__Alphaproteobacteria;o__Rhizobiales;f__Bradyrhizobiaceae;g__Tardiphaga;Ambiguous_taxa
OTU1101	Unassigned	Unassigned
OTU1102	Unassigned	Unassigned
OTU1103	k__Bacteria; p__Proteobacteria; c__Gammaproteobacteria; o__Legionellales; f__Coxiellaceae; g__; s__	k__Bacteria;p__Proteobacteria;c__Gammaproteobacteria;o__Legionellales;f__Coxiellaceae;g__Aquicella;s__uncultured bacterium
OTU1104	k__Bacteria; p__Proteobacteria; c__Betaproteobacteria; o__; f__; g__; s__	k__Bacteria;p__Proteobacteria;c__Betaproteobacteria;o__TRA3-20;f__uncultured bacterium;g__uncultured bacterium;s__uncultured bacterium
OTU1105	Unassigned	Unassigned
OTU1106	k__Bacteria; p__Proteobacteria; c__Alphaproteobacteria; o__Sphingomonadales; f__Sphingomonadaceae	k__Bacteria;p__Proteobacteria;c__Alphaproteobacteria;o__Sphingomonadales;f__Sphingomonadaceae
OTU1107	k__Bacteria; p__Firmicutes; c__Bacilli; o__Bacillales; f__Bacillaceae; g__Bacillus; s__	k__Bacteria;p__Firmicutes;c__Bacilli;o__Bacillales;f__Bacillaceae;g__Bacillus;Ambiguous_taxa
OTU1108	k__Bacteria; p__Acidobacteria; c__Solibacteres; o__Solibacterales; f__Solibacteraceae; g__; s__	k__Bacteria;p__Acidobacteria;c__Solibacteres;o__Solibacterales;f__Solibacteraceae (Subgroup 3);g__Bryobacter;s__uncultured bacterium
OTU1109	k__Bacteria; p__TM7; c__TM7-1; o__; f__; g__; s__	k__Bacteria;p__Saccharibacteria;c__uncultured bacterium;o__uncultured bacterium;f__uncultured bacterium;g__uncultured bacterium;s__uncultured bacterium
OTU111	Unassigned	Unassigned
OTU1110	k__Bacteria; p__Proteobacteria; c__Gammaproteobacteria; o__Xanthomonadales; f__Xanthomonadaceae; g__; s__	k__Bacteria;p__Proteobacteria;c__Gammaproteobacteria;o__Xanthomonadales;f__Xanthomonadaceae;g__uncultured;s__uncultured bacterium
OTU1111	k__Bacteria; p__Proteobacteria; c__Gammaproteobacteria; o__Xanthomonadales; f__Sinobacteraceae; g__; s__	k__Bacteria;p__Proteobacteria;c__Gammaproteobacteria;o__Xanthomonadales;f__Xanthomonadales Incertae Sedis;g__Acidibacter;s__uncultured bacterium
OTU1112	k__Bacteria; p__Proteobacteria; c__Alphaproteobacteria; o__Rhizobiales; f__Methylobacteriaceae; g__Methylobacterium; s__	k__Bacteria;p__Proteobacteria;c__Alphaproteobacteria;o__Rhizobiales;f__Methylobacteriaceae;g__Methylobacterium;s__uncultured bacterium
OTU1113	k__Bacteria; p__Cyanobacteria; c__; o__; f__; g__; s__	k__Bacteria;p__Cyanobacteria;c__Cyanobacteria;o__SubsectionIII;f__FamilyI;g__Lyngbya;Ambiguous_taxa

OTU1114	k__Bacteria; p__[Thermi]; c__Deinococci; o__Deinococcales; f__Deinococcaceae; g__Deinococcus; s__	k__Bacteria;p__Deinococcus-Thermus;c__Deinococci;o__Deinococcales;f__Deinococcaceae;g__Deinococcus;Ambiguous_taxa
OTU1115	k__Bacteria; p__Bacteroidetes; c__[Saprospirae]; o__[Saprospirales]; f__Chitinophagaceae; g__Flavisolibacter; s__	k__Bacteria;p__Bacteroidetes;c__Sphingobacteriia;o__Sphingobacteriales;f__Chitinophagaceae;g__uncultured
OTU1116	k__Bacteria; p__Proteobacteria; c__Gammaproteobacteria; o__Legionellales; f__Legionellaceae; g__; s__	k__Bacteria;p__Proteobacteria;c__Gammaproteobacteria;o__Legionellales;f__Legionellaceae;g__Legionella
OTU1117	k__Bacteria; p__Actinobacteria; c__Actinobacteria; o__Actinomycetales; f__Intrasporangiaceae	k__Bacteria;p__Actinobacteria;c__Actinobacteria;o__Micrococcales;f__Intrasporangiaceae;g__Lapillicoccus
OTU1118	k__Bacteria; p__Proteobacteria; c__Alphaproteobacteria; o__Sphingomonadales; f__Sphingomonadaceae; g__Sphingomonas; s__wittichii	k__Bacteria;p__Proteobacteria;c__Alphaproteobacteria;o__Sphingomonadales;f__Sphingomonadaceae;g__Sphingomonas;Ambiguous_taxa
OTU1119	Unassigned	Unassigned
OTU112	k__Bacteria; p__Proteobacteria; c__Alphaproteobacteria; o__Sphingomonadales; f__Sphingomonadaceae; g__Sphingomonas; s__wittichii	k__Bacteria;p__Proteobacteria;c__Alphaproteobacteria;o__Sphingomonadales;f__Sphingomonadaceae;g__Sphingomonas;Ambiguous_taxa
OTU1120	k__Bacteria; p__Proteobacteria; c__Alphaproteobacteria; o__Rhodospirillales; f__Acetobacteraceae	k__Bacteria;p__Proteobacteria;c__Alphaproteobacteria;o__Rhodospirillales;f__Acetobacteraceae
OTU1121	k__Bacteria; p__Verrucomicrobia; c__[Spartobacteria]; o__[Chthoniobacterales]; f__[Chthoniobacteraceae]; g__Ellin506; s__	k__Bacteria;p__Verrucomicrobia;c__Spartobacteria;o__Chthoniobacterales;f__Chthoniobacteraceae;g__Chthoniobacter
OTU1122	k__Bacteria; p__Cyanobacteria; c__; o__; f__; g__; s__	k__Bacteria;p__Cyanobacteria;c__Cyanobacteria;o__uncultured;f__uncultured bacterium;g__uncultured bacterium;s__uncultured bacterium
OTU1123	k__Bacteria; p__Proteobacteria; c__Betaproteobacteria; o__Burkholderiales; f__Comamonadaceae; g__; s__	k__Bacteria;p__Proteobacteria;c__Betaproteobacteria;o__Burkholderiales;f__Comamonadaceae;Ambiguous_taxa;Ambiguous_taxa
OTU1124	k__Bacteria; p__Chloroflexi; c__C0119; o__; f__; g__; s__	k__Bacteria;p__Chloroflexi;c__Ktedonobacteria;o__C0119;f__uncultured Chloroflexi bacterium;g__uncultured Chloroflexi bacterium;s__uncultured Chloroflexi bacterium
OTU1125	k__Bacteria; p__Armatimonadetes; c__0319-6E2; o__; f__; g__; s__	k__Bacteria;p__Armatimonadetes;c__uncultured;o__uncultured bacterium;f__uncultured bacterium;g__uncultured bacterium;s__uncultured bacterium
OTU1126	k__Bacteria; p__Proteobacteria; c__Alphaproteobacteria; o__Rhizobiales; f__Methylobacteriaceae; g__; s__	k__Bacteria;p__Proteobacteria;c__Alphaproteobacteria;o__Rhizobiales;f__Methylobacteriaceae;g__Methylobacterium
OTU1127	k__Bacteria; p__Proteobacteria; c__Deltaproteobacteria; o__[Entotheonellales]; f__[Entotheonellaceae]; g__; s__	k__Bacteria;p__Tectomicrobia;c__uncultured bacterium;o__uncultured bacterium;f__uncultured bacterium;g__uncultured bacterium;s__uncultured bacterium
OTU1128	k__Bacteria; p__Bacteroidetes; c__[Saprospirae]; o__[Saprospirales]; f__Chitinophagaceae; g__; s__	k__Bacteria;p__Bacteroidetes;c__Sphingobacteriia;o__Sphingobacteriales;f__Chitinophagaceae;g__Terrimonas;Ambiguous_taxa
OTU1129	k__Bacteria; p__TM7; c__TM7-1; o__; f__; g__; s__	k__Bacteria;p__Saccharibacteria;c__uncultured bacterium;o__uncultured bacterium;f__uncultured bacterium;g__uncultured bacterium;s__uncultured bacterium
OTU113	k__Bacteria; p__Proteobacteria; c__Betaproteobacteria; o__Procabacteriales; f__Procabacteriaceae; g__; s__	k__Bacteria;p__Proteobacteria;c__Betaproteobacteria;o__Neisseriales;f__Neisseriaceae;g__uncultured

OTU1130	k__Bacteria; p__TM7; c__TM7-3; o__; f__; g__; s__	k__Bacteria;p__Saccharibacteria;c__uncultured bacterium;o__uncultured bacterium;f__uncultured bacterium;g__uncultured bacterium;s__uncultured bacterium
OTU1131	k__Bacteria; p__[Thermi]; c__Deinococci; o__Deinococcales; f__Deinococcaceae; g__CM44; s__	k__Bacteria;p__Deinococcus-Thermus;c__Deinococci;o__Deinococcales;f__Deinococcaceae;g__uncultured;s__uncultured bacterium
OTU1132	k__Bacteria; p__Firmicutes; c__Bacilli; o__Lactobacillales; f__Lactobacillaceae; g__Lactobacillus; s__	k__Bacteria;p__Firmicutes;c__Bacilli;o__Lactobacillales;f__Lactobacillaceae;g__Lactobacillus;Ambiguous_taxa
OTU1133	Unassigned	Unassigned
OTU1134	k__Bacteria; p__Chloroflexi; c__Ellin6529; o__; f__; g__; s__	k__Bacteria;p__Chloroflexi;c__KD4-96;o__uncultured bacterium;f__uncultured bacterium;g__uncultured bacterium;s__uncultured bacterium
OTU1135	k__Bacteria; p__Actinobacteria; c__Actinobacteria; o__Actinomycetales; f__Kineosporiaceae; g__Kineosporia; s__	k__Bacteria;p__Actinobacteria;c__Actinobacteria;o__Kineosporiales;f__Kineosporiaceae;g__Kineosporia;Ambiguous_taxa
OTU1136	k__Bacteria; p__Bacteroidetes; c__Cytophagia; o__Cytophagales; f__Cytophagaceae; g__Spirosoma; s__	k__Bacteria;p__Bacteroidetes;c__Cytophagia;o__Cytophagales;f__Cytophagaceae;g__Spirosoma;s__uncultured bacterium
OTU1137	k__Bacteria; p__Actinobacteria; c__MB-A2-108; o__0319-7L14; f__; g__; s__	k__Bacteria;p__Actinobacteria;c__MB-A2-108;o__uncultured bacterium;f__uncultured bacterium;g__uncultured bacterium;s__uncultured bacterium
OTU1138	k__Bacteria; p__Actinobacteria; c__Actinobacteria; o__Actinomycetales; f__Intrasporangiaceae	k__Bacteria;p__Actinobacteria;c__Actinobacteria;o__Micrococcales;f__Dermatophilaceae
OTU1139	k__Bacteria; p__Proteobacteria; c__Gammaproteobacteria; o__Pseudomonadales; f__Moraxellaceae; g__Alkanindiges; s__	k__Bacteria;p__Proteobacteria;c__Gammaproteobacteria;o__Pseudomonadales;f__Moraxellaceae;g__Alkanindiges;Ambiguous_taxa
OTU114	k__Bacteria; p__Planctomycetes; c__Phycisphaerae; o__WD2101; f__; g__; s__	k__Bacteria;p__Planctomycetes;c__Phycisphaerae;o__Tepidisphaerales;f__Tepidisphaeraceae;g__Tepidisphaera;s__uncultured bacterium
OTU1140	k__Bacteria; p__Actinobacteria; c__Actinobacteria; o__Actinomycetales; f__Nocardioidaceae; g__Nocardioides; s__	k__Bacteria;p__Actinobacteria;c__Actinobacteria;o__Propionibacteriales;f__Nocardioidaceae;g__Nocardioides;s__uncultured bacterium
OTU1141	k__Bacteria; p__Proteobacteria; c__Alphaproteobacteria; o__Rhizobiales; f__Beijerinckiaceae; g__; s__	k__Bacteria;p__Proteobacteria;c__Alphaproteobacteria;o__Rhizobiales;f__Methylobacteriaceae
OTU1142	Unassigned	Unassigned
OTU1143	k__Bacteria; p__Actinobacteria; c__Acidimicrobiia; o__Acidimicrobiales; f__; g__; s__	k__Bacteria;p__Actinobacteria;c__Acidimicrobiia;o__Acidimicrobiales;f__uncultured;g__uncultured bacterium;s__uncultured bacterium
OTU1144	k__Bacteria; p__Acidobacteria; c__Acidobacteria-6; o__iii1-15; f__; g__; s__	k__Bacteria;p__Acidobacteria;c__Subgroup 6;o__uncultured bacterium;f__uncultured bacterium;g__uncultured bacterium;s__uncultured bacterium
OTU1145	k__Bacteria; p__Firmicutes; c__Clostridia; o__Clostridiales; f__[Tissierellaceae]; g__Anaerococcus; s__	k__Bacteria;p__Firmicutes;c__Clostridia;o__Clostridiales;f__Family XI;g__Anaerococcus;s__uncultured bacterium
OTU1146	Unassigned	Unassigned
OTU1147	k__Bacteria; p__Proteobacteria; c__Gammaproteobacteria; o__Pseudomonadales; f__Moraxellaceae; g__Acinetobacter; s__	k__Bacteria;p__Proteobacteria;c__Gammaproteobacteria;o__Pseudomonadales;f__Moraxellaceae;g__Acinetobacter
OTU1148	k__Bacteria; p__Proteobacteria; c__Alphaproteobacteria; o__Sphingomonadales; f__Sphingomonadaceae; g__; s__	k__Bacteria;p__Proteobacteria;c__Alphaproteobacteria;o__Sphingomonadales

OTU1149	k__Bacteria; p__Cyanobacteria; c__Chloroplast; o__Streptophyta; f__; g__; s__	k__Bacteria;p__Cyanobacteria;c__Chloroplast;o__Cymbidium faberi;f__Cymbidium faberi;g__Cymbidium faberi;s__Cymbidium faberi
OTU115	k__Bacteria; p__Proteobacteria; c__Alphaproteobacteria; o__Rhodospirillales; f__Acetobacteraceae	k__Bacteria;p__Proteobacteria;c__Alphaproteobacteria;o__Rhodospirillales;f__Acetobacteraceae
OTU1150	k__Bacteria; p__Proteobacteria; c__Alphaproteobacteria; o__Sphingomonadales; f__Sphingomonadaceae; g__Kaistobacter; s__	k__Bacteria;p__Proteobacteria;c__Alphaproteobacteria;o__Sphingomonadales;f__Sphingomonadaceae;g__Sphingomonas
OTU1151	k__Bacteria; p__Verrucomicrobia; c__[Spartobacteria]; o__[Chthoniobacterales]; f__[Chthoniobacteraceae]; g__; s__	k__Bacteria;p__Verrucomicrobia;c__Spartobacteria;o__Chthoniobacterales;f__LD29;g__uncultured bacterium;s__uncultured bacterium
OTU1152	k__Bacteria; p__Planctomycetes; c__Planctomycetia; o__Gemmatales; f__Gemmataceae; g__Gemmata; s__	k__Bacteria;p__Planctomycetes;c__Planctomycetacia;o__Planctomycetales;f__Planctomycetaceae;g__uncultured;s__uncultured bacterium
OTU1153	Unassigned	Unassigned
OTU1154	k__Bacteria; p__Proteobacteria; c__Alphaproteobacteria; o__Sphingomonadales; f__Sphingomonadaceae; g__; s__	k__Bacteria;p__Proteobacteria;c__Alphaproteobacteria;o__Sphingomonadales;f__Sphingomonadaceae;g__Sphingomonas;Ambiguous_taxa
OTU1155	k__Bacteria; p__Armatimonadetes; c__Chthonomonadetes; o__Chthonomonadales; f__Chthonomonadaceae; g__Chthonomonas; s__	k__Bacteria;p__Armatimonadetes;c__Chthonomonadetes;o__Chthonomonadales;f__Chthonomonadaceae;g__Chthonomonas;s__uncultured bacterium
OTU1156	k__Bacteria; p__Proteobacteria; c__Gammaproteobacteria; o__Enterobacteriales; f__Enterobacteriaceae; g__; s__	k__Bacteria;p__Proteobacteria;c__Gammaproteobacteria;o__Enterobacteriales;f__Enterobacteriaceae;g__Arsenophonus
OTU1157	Unassigned	Unassigned
OTU1158	Unassigned	Unassigned
OTU1159	k__Bacteria; p__Proteobacteria; c__Alphaproteobacteria; o__Rhodobacterales; f__Rhodobacteraceae; g__; s__	k__Bacteria;p__Proteobacteria;c__Alphaproteobacteria;o__Rhodobacterales;f__Rhodobacteraceae;g__Oceanicella
OTU116	k__Bacteria; p__Proteobacteria; c__Alphaproteobacteria; o__Sphingomonadales; f__Sphingomonadaceae; g__Sphingomonas; s__	k__Bacteria;p__Proteobacteria;c__Alphaproteobacteria;o__Sphingomonadales;f__AKYG937
OTU1160	k__Bacteria; p__Cyanobacteria; c__Oscillatoriophycideae; o__Chroococcales; f__Xenococcaceae; g__Chroococcidiopsis; s__	k__Bacteria;p__Cyanobacteria;c__Cyanobacteria;o__uncultured;f__uncultured bacterium;g__uncultured bacterium;s__uncultured bacterium
OTU1161	k__Bacteria; p__Acidobacteria; c__[Chloracidobacteria]; o__PK29; f__; g__; s__	k__Bacteria;p__Acidobacteria;c__Blastocatellia;o__Blastocatellales;f__Blastocatellaceae (Subgroup 4);g__11-24
OTU1162	Unassigned	Unassigned
OTU1163	k__Bacteria; p__Bacteroidetes; c__Flavobacteriia; o__Flavobacteriales; f__[Weeksellaceae]	k__Bacteria;p__Bacteroidetes;c__Flavobacteriia;o__Flavobacteriales;f__Flavobacteriaceae
OTU1164	k__Bacteria; p__Armatimonadetes; c__Chthonomonadetes; o__Chthonomonadales; f__Chthonomonadaceae; g__Chthonomonas; s__	k__Bacteria;p__Armatimonadetes;c__Chthonomonadetes;o__Chthonomonadales;f__Chthonomonadaceae;g__Chthonomonas;s__uncultured bacterium
OTU1165	k__Bacteria; p__Proteobacteria; c__Alphaproteobacteria; o__Rhizobiales; f__Methylocystaceae; g__; s__	k__Bacteria;p__Proteobacteria;c__Alphaproteobacteria;o__Rhizobiales;f__1174-901-12;g__uncultured bacterium;s__uncultured bacterium
OTU1166	k__Bacteria; p__Bacteroidetes; c__Flavobacteriia; o__Flavobacteriales; f__[Weeksellaceae]; g__Chryseobacterium; s__	k__Bacteria;p__Bacteroidetes;c__Flavobacteriia;o__Flavobacteriales;f__Flavobacteriaceae;g__Chryseobacterium;Ambiguous_taxa
OTU1167	k__Bacteria; p__Proteobacteria; c__Gammaproteobacteria; o__Enterobacteriales; f__Enterobacteriaceae; g__Klebsiella; s__	k__Bacteria;p__Proteobacteria;c__Gammaproteobacteria;o__Enterobacteriales;f__Enterobacteriaceae;g__Klebsiella
OTU1168	Unassigned	Unassigned
OTU1169	k__Bacteria; p__Proteobacteria; c__Alphaproteobacteria; o__Caulobacterales; f__Caulobacteraceae; g__; s__	k__Bacteria;p__Proteobacteria;c__Alphaproteobacteria;o__Caulobacterales;f__Caulobacteraceae;Ambiguous_taxa;Ambiguous_taxa

OTU117	k__Bacteria; p__Bacteroidetes; c__[Saprospirae]; o__[Saprospirales]; f__Chitinophagaceae; g__; s__	k__Bacteria;p__Bacteroidetes;c__Sphingobacteriia;o__Sphingobacteriales;f__Chitinophagaceae;g__Ferruginibacter
OTU1170	Unassigned	Unassigned
OTU1171	k__Bacteria; p__Armatimonadetes; c__Armatimonadia; o__Armatimonadales; f__Armatimonadaceae; g__; s__	k__Bacteria;p__Armatimonadetes;c__Armatimonadia;o__Armatimonadales;f__uncultured bacterium;g__uncultured bacterium;s__uncultured bacterium
OTU1172	Unassigned	Unassigned
OTU1173	k__Bacteria; p__Cyanobacteria; c__Nostocophycideae; o__Stigonematales; f__Rivulariaceae; g__Calothrix; s__	k__Bacteria;p__Cyanobacteria;c__Cyanobacteria;o__SubsectionIV;f__FamilyII;g__Calothrix;Ambiguous_taxa
OTU1174	Unassigned	Unassigned
OTU1175	Unassigned	Unassigned
OTU1176	Unassigned	Unassigned
OTU1177	k__Bacteria; p__Proteobacteria; c__Alphaproteobacteria; o__Rhizobiales; f__Beijerinckiaceae; g__; s__	k__Bacteria;p__Proteobacteria;c__Alphaproteobacteria;o__Rhizobiales;f__Methylobacteriaceae;g__uncultured;Ambiguous_taxa
OTU1178	k__Bacteria; p__Proteobacteria; c__Betaproteobacteria; o__Burkholderiales; f__Oxalobacteraceae; g__; s__	k__Bacteria;p__Proteobacteria;c__Betaproteobacteria;o__Burkholderiales;f__Oxalobacteraceae;g__Massilia;Ambiguous_taxa
OTU1179	k__Bacteria; p__Proteobacteria; c__Alphaproteobacteria; o__Sphingomonadales; f__Sphingomonadaceae; g__Kaistobacter; s__	k__Bacteria;p__Proteobacteria;c__Alphaproteobacteria;o__Sphingomonadales;f__Sphingomonadaceae;g__Sphingomonas;Ambiguous_taxa
OTU118	k__Bacteria; p__Actinobacteria; c__Actinobacteria; o__Actinomycetales; f__; g__; s__	k__Bacteria;p__Actinobacteria;c__Actinobacteria;o__Kineosporiales;f__Kineosporiaceae;g__Quadrisphaera;s__uncultured bacterium
OTU1180	k__Bacteria; p__Cyanobacteria; c__Oscillatoriophycideae; o__Chroococcales; f__Xenococcaceae; g__; s__	k__Bacteria;p__Cyanobacteria;c__Cyanobacteria;o__SubsectionII;f__FamilyII;g__Chroococcidiopsis;Ambiguous_taxa
OTU1181	k__Bacteria; p__TM7; c__TM7-3; o__; f__; g__; s__	k__Bacteria;p__Saccharibacteria;c__uncultured bacterium;o__uncultured bacterium;f__uncultured bacterium;g__uncultured bacterium;s__uncultured bacterium
OTU1182	k__Bacteria; p__Proteobacteria; c__Alphaproteobacteria; o__Rhizobiales; f__Methylobacteriaceae; g__; s__	k__Bacteria;p__Proteobacteria;c__Alphaproteobacteria;o__Rhizobiales;f__Methylobacteriaceae;g__Methylobacterium
OTU1183	k__Bacteria; p__[Thermi]; c__Deinococci; o__Deinococcales; f__Deinococcaceae; g__Deinococcus; s__	k__Bacteria;p__Deinococcus-Thermus;c__Deinococci;o__Deinococcales;f__Deinococcaceae;g__Deinococcus;Ambiguous_taxa
OTU1184	Unassigned	Unassigned
OTU1185	k__Bacteria; p__TM6; c__SJA-4; o__; f__; g__; s__	k__Bacteria;p__TM6 (Dependentiae)
OTU1186	Unassigned	Unassigned
OTU1187	k__Bacteria; p__Proteobacteria; c__Alphaproteobacteria; o__Rhodospirillales; f__Acetobacteraceae; g__Roseococcus; s__	k__Bacteria;p__Proteobacteria;c__Alphaproteobacteria;o__Rhodospirillales;f__Acetobacteraceae;g__uncultured
OTU1188	k__Bacteria; p__Proteobacteria; c__Alphaproteobacteria; o__Rhizobiales; f__Hyphomicrobiaceae; g__Rhodoplanes; s__	k__Bacteria;p__Proteobacteria;c__Alphaproteobacteria;o__Rhizobiales;f__Xanthobacteraceae
OTU1189	k__Bacteria; p__Proteobacteria; c__Alphaproteobacteria; o__Rhodobacterales; f__Rhodobacteraceae; g__; s__	k__Bacteria;p__Proteobacteria;c__Alphaproteobacteria;o__Rhodobacterales;f__Rhodobacteraceae
OTU119	k__Bacteria; p__Acidobacteria; c__Acidobacteriia; o__Acidobacteriales; f__Acidobacteriaceae; g__; s__	k__Bacteria;p__Acidobacteria;c__Acidobacteria;o__Acidobacteriales;f__Acidobacteriaceae (Subgroup 1);g__Edaphobacter

OTU1190	k__Bacteria; p__Proteobacteria; c__Gammaproteobacteria; o__Oceanospirillales; f__Halomonadaceae; g__Haererehalobacter; s__salaria	k__Bacteria;p__Proteobacteria;c__Gammaproteobacteria;o__Oceanospirillales;f__Halomonadaceae;g__Salinicola;Ambiguous_taxa
OTU1191	k__Bacteria; p__Cyanobacteria; c__Oscillatoriophycideae; o__Chroococcales; f__; g__; s__	k__Bacteria;p__Cyanobacteria;c__Cyanobacteria;o__SubsectionI;f__FamilyI
OTU1192	k__Bacteria; p__Proteobacteria; c__Deltaproteobacteria; o__Myxococcales; f__; g__; s__	k__Bacteria;p__Proteobacteria;c__Deltaproteobacteria;o__Myxococcales;f__Polyangiaceae;g__Sorangium;s__uncultured bacterium
OTU1193	k__Bacteria; p__Proteobacteria; c__Gammaproteobacteria; o__Pseudomonadales; f__Moraxellaceae; g__Acinetobacter; s__	k__Bacteria;p__Proteobacteria;c__Gammaproteobacteria;o__Pseudomonadales;f__Moraxellaceae;g__Acinetobacter
OTU1194	k__Bacteria; p__Proteobacteria; c__Betaproteobacteria; o__Ellin6067; f__; g__; s__	k__Bacteria;p__Proteobacteria;c__Betaproteobacteria;o__Nitrosomonadales;f__Nitrosomonadaceae;g__uncultured;s__uncultured bacterium
OTU1195	k__Bacteria; p__Proteobacteria; c__Betaproteobacteria; o__Burkholderiales; f__Comamonadaceae	k__Bacteria;p__Proteobacteria;c__Betaproteobacteria;o__Burkholderiales;f__Comamonadaceae;Ambiguous_taxa;Ambiguous_taxa
OTU1196	k__Bacteria; p__Actinobacteria; c__Actinobacteria; o__Actinomycetales; f__Nocardiaceae; g__Nocardia; s__	k__Bacteria;p__Actinobacteria;c__Actinobacteria;o__Corynebacteriales;f__nbr16a11;g__uncultured bacterium;s__uncultured bacterium
OTU1197	k__Bacteria; p__Proteobacteria; c__Gammaproteobacteria; o__Pseudomonadales; f__Pseudomonadaceae; g__Pseudomonas; s__	k__Bacteria;p__Proteobacteria;c__Gammaproteobacteria;o__Pseudomonadales;f__Pseudomonadaceae;g__Pseudomonas
OTU1198	k__Bacteria; p__Bacteroidetes; c__Sphingobacteriia; o__Sphingobacteriales; f__Sphingobacteriaceae; g__; s__	k__Bacteria;p__Bacteroidetes;c__Sphingobacteriia;o__Sphingobacteriales;f__Sphingobacteriaceae;g__Mucilaginibacter;Ambiguous_taxa
OTU1199	k__Bacteria; p__Firmicutes; c__Bacilli; o__Lactobacillales; f__Lactobacillaceae; g__Lactobacillus; s__	k__Bacteria;p__Firmicutes;c__Bacilli;o__Lactobacillales;f__Lactobacillaceae;g__Lactobacillus;Ambiguous_taxa
OTU12	k__Bacteria; p__Proteobacteria; c__Alphaproteobacteria; o__Sphingomonadales; f__Sphingomonadaceae; g__; s__	k__Bacteria;p__Proteobacteria;c__Alphaproteobacteria;o__Sphingomonadales;f__Ellin6055;g__uncultured bacterium;s__uncultured bacterium
OTU120	k__Bacteria; p__Firmicutes; c__Clostridia; o__Clostridiales; f__Veillonellaceae; g__Megasphaera; s__	k__Bacteria;p__Firmicutes;c__Negativicutes;o__Selenomonadales;f__Veillonellaceae;g__Megasphaera
OTU1200	k__Bacteria; p__Acidobacteria; c__Acidobacteriia; o__Acidobacteriales; f__Acidobacteriaceae; g__Terriglobus; s__	k__Bacteria;p__Acidobacteria;c__Acidobacteria;o__Acidobacteriales;f__Acidobacteriaceae (Subgroup 1);g__Terriglobus;Ambiguous_taxa
OTU1201	k__Bacteria; p__Proteobacteria; c__Alphaproteobacteria; o__Sphingomonadales; f__Sphingomonadaceae; g__; s__	k__Bacteria;p__Proteobacteria;c__Alphaproteobacteria;o__Sphingomonadales;f__Sphingomonadaceae;g__Sphingomonas
OTU1202	Unassigned	Unassigned
OTU1203	k__Bacteria; p__Actinobacteria; c__Actinobacteria; o__Actinomycetales; f__Williamsiaceae; g__Williamsia; s__	k__Bacteria;p__Actinobacteria;c__Actinobacteria;o__Corynebacteriales;f__Nocardiaceae;g__Gordonia
OTU1204	k__Bacteria; p__TM7; c__TM7-1; o__; f__; g__; s__	k__Bacteria;p__Saccharibacteria;c__uncultured bacterium;o__uncultured bacterium;f__uncultured bacterium;g__uncultured bacterium;s__uncultured bacterium
OTU1205	Unassigned	Unassigned
OTU1206	Unassigned	Unassigned
OTU1207	Unassigned	Unassigned
OTU1208	k__Bacteria; p__Acidobacteria; c__Solibacteres; o__Solibacterales; f__Solibacteraceae; g__; s__	k__Bacteria;p__Acidobacteria;c__Solibacteres;o__Solibacterales;f__Solibacteraceae (Subgroup 3);g__Bryobacter;s__uncultured bacterium
OTU1209	k__Bacteria; p__Actinobacteria; c__Acidimicrobiia; o__Acidimicrobiales; f__; g__; s__	k__Bacteria;p__Actinobacteria;c__Acidimicrobiia;o__Acidimicrobiales;f__uncultured

OTU121	k__Bacteria; p__Proteobacteria; c__Deltaproteobacteria; o__Myxococcales; f__; g__; s__	k__Bacteria;p__Proteobacteria;c__Deltaproteobacteria;o__Myxococcales;f__Polyangiaceae;g__Sorangium
OTU1210	k__Bacteria; p__Proteobacteria; c__Betaproteobacteria; o__Burkholderiales; f__Burkholderiaceae; g__; s__	k__Bacteria;p__Proteobacteria;c__Betaproteobacteria;o__Burkholderiales;f__Burkholderiaceae;g__Lautropia;s__uncultured bacterium
OTU1211	k__Bacteria; p__Actinobacteria; c__Coriobacteriia; o__Coriobacteriales; f__Coriobacteriaceae; g__; s__	k__Bacteria;p__Actinobacteria;c__Coriobacteriia;o__Coriobacteriales;f__Coriobacteriaceae;g__uncultured
OTU1212	Unassigned	Unassigned
OTU1213	Unassigned	Unassigned
OTU1214	Unassigned	Unassigned
OTU1215	k__Bacteria; p__Bacteroidetes; c__Cytophagia; o__Cytophagales; f__Cytophagaceae; g__Spirosoma; s__	k__Bacteria;p__Bacteroidetes;c__Cytophagia;o__Cytophagales;f__Cytophagaceae;g__Spirosoma
OTU1216	k__Bacteria; p__Proteobacteria; c__Deltaproteobacteria; o__Myxococcales; f__Nannocystaceae; g__Nannocystis; s__	k__Bacteria;p__Proteobacteria;c__Deltaproteobacteria;o__Myxococcales;f__Nannocystaceae;g__Nannocystis;Ambiguous_taxa
OTU1217	k__Bacteria; p__Proteobacteria; c__Alphaproteobacteria; o__Rhodospirillales; f__Acetobacteraceae	k__Bacteria;p__Proteobacteria;c__Alphaproteobacteria;o__Rhodospirillales;f__Acetobacteraceae;g__Acetobacter
OTU1218	k__Bacteria; p__Bacteroidetes; c__Flavobacteriia; o__Flavobacteriales; f__[Weeksellaceae]; g__Chryseobacterium; s__	k__Bacteria;p__Bacteroidetes;c__Flavobacteriia;o__Flavobacteriales;f__Flavobacteriaceae;g__Chryseobacterium;Ambiguous_taxa
OTU1219	k__Bacteria; p__Actinobacteria; c__Actinobacteria; o__Actinomycetales; f__Nocardioidaceae; g__; s__	k__Bacteria;p__Actinobacteria;c__Actinobacteria;o__Propionibacteriales;f__Nocardioidaceae;g__Nocardioides
OTU122	k__Bacteria; p__Actinobacteria; c__Actinobacteria; o__Actinomycetales; f__Dermacoccaceae; g__Dermacoccus; s__	k__Bacteria;p__Actinobacteria;c__Actinobacteria;o__Micrococcales;f__Dermacoccaceae;g__Dermacoccus;Ambiguous_taxa
OTU1220	k__Bacteria; p__Cyanobacteria; c__Chloroplast; o__Streptophyta; f__; g__; s__	k__Bacteria;p__Cyanobacteria;c__Chloroplast;Ambiguous_taxa;Ambiguous_taxa;Ambiguous_taxa;Ambiguous_taxa
OTU1221	k__Bacteria; p__Actinobacteria; c__Actinobacteria; o__Actinomycetales; f__Micromonosporaceae; g__; s__	k__Bacteria;p__Actinobacteria;c__Actinobacteria;o__Micromonosporales;f__Micromonosporaceae;g__Catenuloplanes;Ambiguous_taxa
OTU1222	k__Bacteria; p__Actinobacteria; c__Acidimicrobiia; o__Acidimicrobiales; f__C111; g__; s__	k__Bacteria;p__Actinobacteria;c__Acidimicrobiia;o__Acidimicrobiales;f__Acidimicrobiaceae;g__uncultured;s__uncultured bacterium
OTU1223	Unassigned	k__Bacteria;p__Proteobacteria;c__Alphaproteobacteria;o__Rickettsiales;f__SM2D12;g__uncultured bacterium;s__uncultured bacterium
OTU1224	k__Bacteria; p__Bacteroidetes; c__Cytophagia; o__Cytophagales; f__Cytophagaceae; g__Hymenobacter; s__	k__Bacteria;p__Bacteroidetes;c__Cytophagia;o__Cytophagales;f__Cytophagaceae;g__Hymenobacter
OTU1225	k__Bacteria; p__Proteobacteria; c__Alphaproteobacteria; o__Rhizobiales; f__; g__; s__	k__Bacteria;p__Proteobacteria;c__Alphaproteobacteria;o__Rhizobiales;f__Rhizobiales Incertae Sedis;g__Agaricicola
OTU1226	k__Bacteria; p__Acidobacteria; c__Solibacteres; o__Solibacterales; f__Solibacteraceae; g__; s__	k__Bacteria;p__Acidobacteria;c__Solibacteres;o__Solibacterales;f__Solibacteraceae (Subgroup 3);g__Bryobacter;s__uncultured bacterium
OTU1227	k__Bacteria; p__Proteobacteria; c__Alphaproteobacteria; o__Rhizobiales; f__Methylobacteriaceae; g__Methylobacterium; s__	k__Bacteria;p__Proteobacteria;c__Alphaproteobacteria;o__Rhizobiales;f__Methylobacteriaceae;g__Methylobacterium;Ambiguous_taxa
OTU1228	k__Bacteria; p__Actinobacteria; c__Acidimicrobiia; o__Acidimicrobiales; f__C111; g__; s__	k__Bacteria;p__Actinobacteria;c__Acidimicrobiia;o__Acidimicrobiales;f__Acidimicrobiaceae;g__CL500-29 marine group;s__uncultured bacterium
OTU1229	k__Bacteria; p__Actinobacteria; c__Actinobacteria; o__Actinomycetales; f__Streptomycetaceae; g__Streptacidiphilus; s__	k__Bacteria;p__Actinobacteria;c__Actinobacteria;o__Streptomycetales;f__Streptomycetaceae;g__Streptacidiphilus;Ambiguous_taxa

OTU123	k__Bacteria; p__Bacteroidetes; c__[Saprospirae]; o__[Saprospirales]; f__Chitinophagaceae; g__; s__	k__Bacteria;p__Bacteroidetes;c__Sphingobacteriia;o__Sphingobacteriales;f__Chitinophagaceae;g__Taibaiella;s__uncultured bacterium
OTU1230	k__Bacteria; p__Cyanobacteria; c__Chloroplast; o__Streptophyta; f__; g__; s__	k__Bacteria;p__Cyanobacteria;c__Chloroplast;Ambiguous_taxa;Ambiguous_taxa;Ambiguous_taxa;Ambiguous_taxa
OTU1231	k__Bacteria; p__Actinobacteria; c__Actinobacteria; o__Actinomycetales; f__Pseudonocardiaceae; g__Pseudonocardia; s__	k__Bacteria;p__Actinobacteria;c__Actinobacteria;o__Pseudonocardiales;f__Pseudonocardiaceae;g__Pseudonocardia;s__uncultured bacterium
OTU1232	k__Bacteria; p__Cyanobacteria; c__Nostocophycideae; o__Nostocales; f__Nostocaceae	k__Bacteria;p__Cyanobacteria;c__Cyanobacteria;o__SubsectionIII;f__FamilyI;g__Crinalium;s__uncultured bacterium
OTU1233	k__Bacteria; p__Proteobacteria; c__Alphaproteobacteria; o__Rhizobiales; f__Methylocystaceae; g__; s__	k__Bacteria;p__Proteobacteria;c__Alphaproteobacteria;o__Rhizobiales;f__Roseiarcaceae;g__Roseiarcus;Ambiguous_taxa
OTU1234	k__Bacteria; p__Proteobacteria; c__Alphaproteobacteria; o__Rhodospirillales; f__Acetobacteraceae	k__Bacteria;p__Proteobacteria;c__Alphaproteobacteria;o__Rhodospirillales;f__Acetobacteraceae;g__uncultured
OTU1235	k__Bacteria; p__Firmicutes; c__Clostridia; o__Clostridiales; f__Lachnospiraceae; g__; s__	k__Bacteria;p__Firmicutes;c__Clostridia;o__Clostridiales;f__Lachnospiraceae;g__Lachnoclostridium
OTU1236	k__Bacteria; p__Proteobacteria; c__Alphaproteobacteria; o__Rhodospirillales; f__Acetobacteraceae; g__; s__	k__Bacteria;p__Proteobacteria;c__Alphaproteobacteria;o__Rhodospirillales;f__Acetobacteraceae
OTU1237	k__Bacteria; p__Acidobacteria; c__Acidobacteriia; o__Acidobacteriales; f__Acidobacteriaceae; g__; s__	k__Bacteria;p__Acidobacteria;c__Acidobacteria;o__Acidobacteriales;f__Acidobacteriaceae (Subgroup 1)
OTU1238	k__Bacteria; p__Cyanobacteria; c__Oscillatoriophycideae; o__Oscillatoriales; f__Phormidiaceae; g__Phormidium; s__	k__Bacteria;p__Cyanobacteria;c__Cyanobacteria;o__SubsectionIII;f__FamilyI;g__uncultured
OTU1239	k__Bacteria; p__Proteobacteria; c__Alphaproteobacteria; o__Rhizobiales; f__Rhizobiaceae; g__Agrobacterium; s__	k__Bacteria;p__Proteobacteria;c__Alphaproteobacteria;o__Rhizobiales;f__Rhizobiaceae;g__Rhizobium;Ambiguous_taxa
OTU124	Unassigned	Unassigned
OTU1240	Unassigned	k__Bacteria;p__Proteobacteria;c__Alphaproteobacteria;o__Rhodospirillales;f__Acetobacteraceae;g__uncultured;s__uncultured bacterium
OTU1241	k__Bacteria; p__Verrucomicrobia; c__[Spartobacteria]; o__[Chthoniobacterales]; f__[Chthoniobacteraceae]; g__DA101; s__	k__Bacteria;p__Verrucomicrobia;c__Spartobacteria;o__Chthoniobacterales;f__DA101 soil group;Ambiguous_taxa;Ambiguous_taxa
OTU1242	k__Bacteria; p__Planctomycetes; c__Planctomycetia; o__Gemmatales; f__Gemmataceae; g__Gemmata; s__	k__Bacteria;p__Planctomycetes;c__Planctomycetacia;o__Planctomycetales;f__Planctomycetaceae;g__Gemmata;s__uncultured bacterium
OTU1243	Unassigned	Unassigned
OTU1244	k__Bacteria; p__[Thermi]; c__Deinococci; o__Thermales; f__Thermaceae; g__Thermus; s__	k__Bacteria;p__Deinococcus-Thermus;c__Deinococci;o__Thermales;f__Thermaceae;g__Thermus
OTU1245	k__Bacteria; p__Proteobacteria; c__Alphaproteobacteria; o__Rhodobacterales; f__Rhodobacteraceae; g__Rubellimicrobium; s__	k__Bacteria;p__Proteobacteria;c__Alphaproteobacteria;o__Rhodobacterales;f__Rhodobacteraceae;g__Rubellimicrobium;s__uncultured bacterium
OTU1246	Unassigned	Unassigned
OTU1247	k__Bacteria; p__Chloroflexi; c__Anaerolineae; o__Ardenscatenales; f__Ardenscatenaceae; g__Ardenscatena; s__	k__Bacteria;p__Chloroflexi;c__Ardenticatenia;o__Ardenticatenales;f__uncultured bacterium;g__uncultured bacterium;s__uncultured bacterium
OTU1248	Unassigned	Unassigned
OTU1249	Unassigned	Unassigned
OTU125	k__Bacteria; p__Proteobacteria; c__Alphaproteobacteria; o__Rhodospirillales; f__Rhodospirillaceae; g__; s__	k__Bacteria;p__Proteobacteria;c__Alphaproteobacteria;o__Rhodospirillales;f__DA111

OTU1250	k__Bacteria; p__Firmicutes; c__Bacilli; o__Bacillales; f__Staphylococcaceae; g__Staphylococcus; s__sciuri	k__Bacteria;p__Firmicutes;c__Bacilli;o__Bacillales;f__Staphylococcaceae;g__Staphylococcus
OTU1251	k__Bacteria; p__Proteobacteria; c__Alphaproteobacteria; o__Sphingomonadales; f__Sphingomonadaceae; g__Sphingomonas; s__wittichii	k__Bacteria;p__Proteobacteria;c__Alphaproteobacteria;o__Sphingomonadales;f__MN 122.2a;g__uncultured bacterium;s__uncultured bacterium
OTU1252	k__Bacteria; p__Actinobacteria; c__Actinobacteria; o__Actinomycetales; f__Geodermatophilaceae; g__Geodermatophilus	k__Bacteria;p__Actinobacteria;c__Actinobacteria;o__Frankiales;f__Geodermatophilaceae;g__Geodermatophilus
OTU1253	k__Bacteria; p__Actinobacteria; c__Acidimicrobiia; o__Acidimicrobiales; f__; g__; s__	k__Bacteria;p__Actinobacteria;c__Acidimicrobiia;o__Acidimicrobiales;f__Acidimicrobiaceae;g__uncultured;s__uncultured bacterium
OTU1254	k__Bacteria; p__Proteobacteria; c__Alphaproteobacteria; o__Sphingomonadales; f__Sphingomonadaceae; g__Kaistobacter; s__	k__Bacteria;p__Proteobacteria;c__Alphaproteobacteria;o__Sphingomonadales;f__Sphingomonadaceae;g__Sphingomonas;Ambiguous_taxa
OTU1255	k__Bacteria; p__Proteobacteria; c__Alphaproteobacteria; o__Rickettsiales; f__Rickettsiaceae; g__Wolbachia; s__	k__Bacteria;p__Proteobacteria;c__Alphaproteobacteria;o__Rickettsiales;f__Anaplasmataceae;g__Wolbachia
OTU1256	k__Bacteria; p__Proteobacteria; c__Betaproteobacteria; o__Burkholderiales; f__Alcaligenaceae; g__; s__	k__Bacteria;p__Proteobacteria;c__Betaproteobacteria;o__Burkholderiales;f__Alcaligenaceae;g__uncultured;s__uncultured bacterium
OTU1257	k__Bacteria; p__Bacteroidetes; c__Cytophagia; o__Cytophagales; f__Cytophagaceae; g__; s__	k__Bacteria;p__Bacteroidetes;c__Cytophagia;o__Cytophagales;f__Cytophagaceae;g__Ohtaekwangia;Ambiguous_taxa
OTU1258	k__Bacteria; p__Armatimonadetes; c__[Fimbriimonadia]; o__[Fimbriimonadales]; f__[Fimbriimonadaceae]; g__Fimbriimonas; s__	k__Bacteria;p__Armatimonadetes;c__Fimbriimonadia;o__Fimbriimonadales;f__Fimbriimonadaceae;g__uncultured bacterium;s__uncultured bacterium
OTU1259	k__Bacteria; p__Actinobacteria; c__Actinobacteria; o__Actinomycetales; f__Kineosporiaceae	k__Bacteria;p__Actinobacteria;c__Actinobacteria;o__Kineosporiales;f__Kineosporiaceae;g__Angustibacter
OTU126	k__Bacteria; p__Proteobacteria; c__Alphaproteobacteria; o__Rhizobiales; f__Beijerinckiaceae; g__; s__	k__Bacteria;p__Proteobacteria;c__Alphaproteobacteria;o__Rhizobiales;f__Methylobacteriaceae
OTU1260	k__Bacteria; p__Acidobacteria; c__Acidobacteria-6; o__iii1-15; f__; g__; s__	k__Bacteria;p__Acidobacteria;c__Subgroup 6
OTU1261	Unassigned	Unassigned
OTU1262	Unassigned	Unassigned
OTU1263	k__Bacteria; p__Proteobacteria; c__Gammaproteobacteria; o__Pseudomonadales; f__Moraxellaceae; g__Acinetobacter; s__	k__Bacteria;p__Proteobacteria;c__Gammaproteobacteria;o__Pseudomonadales;f__Moraxellaceae;g__Acinetobacter
OTU1264	k__Bacteria; p__Armatimonadetes; c__[Fimbriimonadia]; o__[Fimbriimonadales]; f__[Fimbriimonadaceae]; g__Fimbriimonas; s__	k__Bacteria;p__Armatimonadetes;c__Fimbriimonadia;o__Fimbriimonadales;f__Fimbriimonadaceae;g__uncultured bacterium;s__uncultured bacterium
OTU1265	Unassigned	Unassigned
OTU1266	k__Bacteria; p__Proteobacteria; c__Alphaproteobacteria; o__Rhizobiales; f__Hyphomicrobiaceae; g__Rhodoplanes; s__	k__Bacteria;p__Proteobacteria;c__Alphaproteobacteria;o__Rhizobiales;f__Hyphomicrobiaceae;g__Rhodoplanes
OTU1267	k__Bacteria; p__Proteobacteria; c__Alphaproteobacteria; o__Sphingomonadales; f__Sphingomonadaceae; g__; s__	k__Bacteria;p__Proteobacteria;c__Alphaproteobacteria;o__Sphingomonadales;f__Sphingomonadaceae;g__Sphingomonas
OTU1268	k__Bacteria; p__Proteobacteria; c__Gammaproteobacteria; o__Legionellales; f__Coxiellaceae; g__Rickettsiella; s__	k__Bacteria;p__Proteobacteria;c__Gammaproteobacteria;o__Legionellales;f__Coxiellaceae;g__Rickettsiella;s__uncultured bacterium
OTU1269	k__Bacteria; p__Firmicutes; c__Bacilli; o__Lactobacillales; f__Leuconostocaceae; g__Weissella; s__	k__Bacteria;p__Firmicutes;c__Bacilli;o__Lactobacillales;f__Leuconostocaceae;g__Weissella;Ambiguous_taxa
OTU127	k__Bacteria; p__Proteobacteria; c__Alphaproteobacteria; o__Caulobacterales; f__Caulobacteraceae; g__; s__	k__Bacteria;p__Proteobacteria;c__Alphaproteobacteria;o__Caulobacterales;f__Caulobacteraceae;g__uncultured;s__uncultured bacterium

OTU1270	k__Bacteria; p__Proteobacteria; c__Gammaproteobacteria; o__Enterobacteriales; f__Enterobacteriaceae; g__Proteus; s__	k__Bacteria;p__Proteobacteria;c__Gammaproteobacteria;o__Enterobacteriales;f__Enterobacteriaceae;g__Proteus;s__Proteus vulgaris
OTU1271	k__Bacteria; p__Proteobacteria; c__Gammaproteobacteria; o__Pseudomonadales; f__Moraxellaceae; g__Acinetobacter; s__	k__Bacteria;p__Proteobacteria;c__Gammaproteobacteria;o__Pseudomonadales;f__Moraxellaceae;g__Acinetobacter
OTU1272	k__Bacteria; p__Proteobacteria; c__Alphaproteobacteria; o__; f__; g__; s__	k__Bacteria;p__Proteobacteria;c__Alphaproteobacteria
OTU1273	k__Bacteria; p__Firmicutes; c__Bacilli; o__Lactobacillales; f__Lactobacillaceae; g__Lactobacillus; s__	k__Bacteria;p__Firmicutes;c__Bacilli;o__Lactobacillales;f__Lactobacillaceae;g__Lactobacillus;Ambiguous_taxa
OTU1274	Unassigned	Unassigned
OTU1275	k__Bacteria; p__Acidobacteria; c__Acidobacteria-6; o__iii1-15; f__; g__; s__	k__Bacteria;p__Acidobacteria;c__Subgroup 6
OTU1276	k__Bacteria; p__Proteobacteria; c__Betaproteobacteria; o__Burkholderiales; f__Comamonadaceae; g__; s__	k__Bacteria;p__Proteobacteria;c__Betaproteobacteria;o__Burkholderiales;f__Comamonadaceae;g__uncultured
OTU1277	k__Bacteria; p__Proteobacteria; c__Gammaproteobacteria; o__Enterobacteriales; f__Enterobacteriaceae; g__; s__	k__Bacteria;p__Proteobacteria;c__Gammaproteobacteria;o__Enterobacteriales;f__Enterobacteriaceae;g__Enterobacter
OTU1278	k__Bacteria; p__Proteobacteria; c__Alphaproteobacteria; o__Rhodospirillales; f__Acetobacteraceae	k__Bacteria;p__Proteobacteria;c__Alphaproteobacteria;o__Rhodospirillales;f__Acetobacteraceae
OTU1279	Unassigned	Unassigned
OTU128	k__Bacteria; p__[Thermi]; c__Deinococci; o__Deinococcales; f__Deinococcaceae; g__Deinococcus; s__	k__Bacteria;p__Deinococcus-Thermus;c__Deinococci;o__Deinococcales;f__Deinococcaceae;g__Deinococcus;Ambiguous_taxa
OTU1280	k__Bacteria; p__Proteobacteria; c__Alphaproteobacteria; o__Rhizobiales; f__Methylobacteriaceae; g__Methylobacterium; s__organophilum	k__Bacteria;p__Proteobacteria;c__Alphaproteobacteria;o__Rhizobiales;f__Methylobacteriaceae;g__Methylobacterium;s__uncultured bacterium
OTU1281	k__Bacteria; p__Proteobacteria; c__Alphaproteobacteria; o__Sphingomonadales; f__Sphingomonadaceae; g__Sphingomonas; s__	k__Bacteria;p__Proteobacteria;c__Alphaproteobacteria;o__Sphingomonadales;f__Sphingomonadaceae;g__Sphingomonas
OTU1282	k__Bacteria; p__Actinobacteria; c__Actinobacteria; o__Actinomycetales; f__Nocardioidaceae; g__; s__	k__Bacteria;p__Actinobacteria;c__Actinobacteria;o__Propionibacteriales;f__Nocardioidaceae
OTU1283	Unassigned	Unassigned
OTU1284	k__Bacteria; p__Proteobacteria; c__Betaproteobacteria; o__Burkholderiales; f__Oxalobacteraceae; g__; s__	k__Bacteria;p__Proteobacteria;c__Betaproteobacteria;o__Burkholderiales;f__Oxalobacteraceae;g__Massilia
OTU1285	k__Bacteria; p__Cyanobacteria; c__Nostocophycideae; o__Stigonematales; f__Rivulariaceae; g__Calothrix; s__	k__Bacteria;p__Cyanobacteria;c__Cyanobacteria;o__SubsectionIV;f__FamilyII;g__Calothrix;Ambiguous_taxa
OTU1286	k__Bacteria; p__Verrucomicrobia; c__[Spartobacteria]; o__[Chthoniobacterales]; f__[Chthoniobacteraceae]; g__; s__	k__Bacteria;p__Verrucomicrobia;c__Spartobacteria;o__Chthoniobacterales;f__LD29;g__uncultured bacterium;s__uncultured bacterium
OTU1287	Unassigned	Unassigned
OTU1288	k__Bacteria; p__Cyanobacteria; c__Synechococcophycideae; o__Pseudanabaenales; f__Pseudanabaenaceae; g__Leptolyngbya; s__	k__Bacteria;p__Cyanobacteria;c__Cyanobacteria;o__SubsectionIII;f__FamilyI;g__Leptolyngbya;s__uncultured bacterium
OTU1289	k__Bacteria; p__Proteobacteria; c__Alphaproteobacteria; o__Rhizobiales; f__; g__; s__	k__Bacteria;p__Proteobacteria;c__Alphaproteobacteria;o__Rhizobiales;f__Bradyrhizobiaceae
OTU129	k__Bacteria; p__WS3; c__PRR-12; o__Sediment-1; f__PRR-10; g__; s__	k__Bacteria;p__Latescibacteria;c__uncultured bacterium;o__uncultured bacterium;f__uncultured bacterium;g__uncultured bacterium;s__uncultured bacterium
OTU1290	k__Bacteria; p__Proteobacteria; c__Alphaproteobacteria; o__Sphingomonadales; f__Sphingomonadaceae; g__Sphingomonas; s__	k__Bacteria;p__Proteobacteria;c__Alphaproteobacteria;o__Sphingomonadales;f__Sphingomonadaceae;g__Sphingomonas;s__uncultured bacterium

OTU1291	k__Bacteria; p__Verrucomicrobia; c__[Spartobacteria]; o__[Chthoniobacterales]; f__[Chthoniobacteraceae]; g__; s__	k__Bacteria;p__Verrucomicrobia;c__Spartobacteria;o__Chthoniobacterales;f__LD29;g__uncultured bacterium;s__uncultured bacterium
OTU1292	k__Bacteria; p__Actinobacteria; c__Actinobacteria; o__Actinomycetales; f__; g__; s__	k__Bacteria;p__Actinobacteria;c__Actinobacteria;o__Frankiales;f__Acidothermaceae;g__Acidothermus
OTU1293	k__Bacteria; p__Actinobacteria; c__Thermoleophilia; o__Solirubrobacterales; f__; g__; s__	k__Bacteria;p__Actinobacteria;c__Thermoleophilia;o__Solirubrobacterales;f__Elev-16S-1332;g__uncultured bacterium;s__uncultured bacterium
OTU1294	k__Bacteria; p__Firmicutes; c__Bacilli; o__Turicibacterales; f__Turicibacteraceae; g__Turicibacter; s__	k__Bacteria;p__Firmicutes;c__Erysipelotrichia;o__Erysipelotrichales;f__Erysipelotrichaceae;g__Turicibacter;s__uncultured bacterium
OTU1295	k__Bacteria; p__Proteobacteria; c__Alphaproteobacteria; o__Rhodospirillales; f__Acetobacteraceae	k__Bacteria;p__Proteobacteria;c__Alphaproteobacteria;o__Rhodospirillales;f__Acetobacteraceae;g__uncultured
OTU1296	k__Bacteria; p__Actinobacteria; c__Actinobacteria; o__Actinomycetales; f__Nocardiaceae; g__Rhodococcus; s__	k__Bacteria;p__Actinobacteria;c__Actinobacteria;o__Corynebacteriales;f__Nocardiaceae;g__Rhodococcus;Ambiguous_taxa
OTU1297	k__Bacteria; p__Fusobacteria; c__Fusobacteriia; o__Fusobacteriales; f__Fusobacteriaceae; g__Cetobacterium; s__somerae	k__Bacteria;p__Fusobacteria;c__Fusobacteriia;o__Fusobacteriales;f__Hados.Sed.Eubac.3;g__uncultured bacterium;s__uncultured bacterium
OTU1298	Unassigned	Unassigned
OTU1299	k__Bacteria; p__Cyanobacteria; c__Oscillatoriophycideae; o__Oscillatoriales; f__Phormidiaceae; g__Phormidium; s__	k__Bacteria;p__Cyanobacteria;c__Cyanobacteria;o__SubsectionIII;f__FamilyI;g__uncultured;s__uncultured bacterium
OTU13	k__Bacteria; p__Bacteroidetes; c__[Saprospirae]; o__[Saprospirales]; f__Chitinophagaceae; g__; s__	k__Bacteria;p__Bacteroidetes;c__Sphingobacteriia;o__Sphingobacteriales;f__Chitinophagaceae;g__uncultured;s__uncultured bacterium
OTU130	k__Bacteria; p__Gemmatimonadetes; c__Gemmatimonadetes; o__; f__; g__; s__	k__Bacteria;p__Gemmatimonadetes;c__Gemmatimonadetes;o__Gemmatimonadales;f__Gemmatimonadaceae;g__uncultured
OTU1300	Unassigned	Unassigned
OTU1301	k__Bacteria; p__Actinobacteria; c__Thermoleophilia; o__Solirubrobacterales; f__Conexibacteraceae; g__; s__	k__Bacteria;p__Actinobacteria;c__Thermoleophilia;o__Solirubrobacterales;f__YNPFFP1;g__uncultured bacterium;s__uncultured bacterium
OTU1302	k__Bacteria; p__Proteobacteria; c__Gammaproteobacteria; o__HTCC2188; f__HTCC2089; g__; s__	k__Bacteria;p__Proteobacteria;c__Gammaproteobacteria;o__KI89A clade;f__uncultured bacterium;g__uncultured bacterium;s__uncultured bacterium
OTU1303	k__Bacteria; p__Actinobacteria; c__Coriobacteriia; o__Coriobacteriales; f__Coriobacteriaceae; g__Eggerthella; s__lenta	k__Bacteria;p__Actinobacteria;c__Coriobacteriia;o__Coriobacteriales;f__Coriobacteriaceae;g__Eggerthella
OTU1304	k__Bacteria; p__Proteobacteria; c__Alphaproteobacteria; o__Caulobacterales; f__Caulobacteraceae	k__Bacteria;p__Proteobacteria;c__Alphaproteobacteria;o__Caulobacterales;f__Caulobacteraceae
OTU1305	k__Bacteria; p__Actinobacteria; c__Actinobacteria; o__Actinomycetales	k__Bacteria;p__Actinobacteria;c__Actinobacteria;o__Corynebacteriales
OTU1306	k__Bacteria; p__Acidobacteria; c__[Chloracidobacteria]; o__RB41; f__; g__; s__	k__Bacteria;p__Acidobacteria;c__Blastocatellia;o__Blastocatellales;f__Blastocatellaceae (Subgroup 4);g__uncultured;s__uncultured bacterium
OTU1307	k__Bacteria; p__Proteobacteria; c__Alphaproteobacteria; o__Rhizobiales; f__Methylocystaceae; g__; s__	k__Bacteria;p__Proteobacteria;c__Alphaproteobacteria;o__Rhizobiales;f__1174-901-12;g__uncultured bacterium;s__uncultured bacterium
OTU1308	k__Bacteria; p__Cyanobacteria; c__Chloroplast; o__Stramenopiles; f__; g__; s__	k__Bacteria;p__Cyanobacteria;c__Chloroplast;o__uncultured bacterium;f__uncultured bacterium;g__uncultured bacterium;s__uncultured bacterium
OTU1309	k__Bacteria; p__Bacteroidetes; c__Bacteroidia; o__Bacteroidales; f__Bacteroidaceae; g__Bacteroides; s__	k__Bacteria;p__Bacteroidetes;c__Bacteroidia;o__Bacteroidales;f__Bacteroidaceae;g__Bacteroides;s__uncultured bacterium

OTU131	k__Bacteria; p__Acidobacteria; c__[Chloracidobacteria]; o__RB41; f__Ellin6075; g__; s__	k__Bacteria;p__Acidobacteria;c__Blastocatellia;o__Blastocatellales;f__Blastocatellaceae (Subgroup 4);g__Blastocatella;s__uncultured bacterium
OTU1310	k__Bacteria; p__Proteobacteria; c__Betaproteobacteria; o__SC-I-84; f__; g__; s__	k__Bacteria;p__Proteobacteria;c__Betaproteobacteria;o__SC-I-84;f__uncultured bacterium;g__uncultured bacterium;s__uncultured bacterium
OTU1311	k__Bacteria; p__Proteobacteria; c__Alphaproteobacteria; o__Caulobacterales; f__Caulobacteraceae; g__; s__	k__Bacteria;p__Proteobacteria;c__Alphaproteobacteria;o__Caulobacterales;f__Caulobacteraceae;Ambiguous_taxa
OTU1312	k__Bacteria; p__Proteobacteria; c__Alphaproteobacteria; o__Rhodospirillales; f__Acetobacteraceae; g__; s__	k__Bacteria;p__Proteobacteria;c__Alphaproteobacteria;o__Rhodospirillales;f__Acetobacteraceae;g__Rhodovarius;s__uncultured bacterium
OTU1313	k__Bacteria; p__Bacteroidetes; c__[Rhodothermi]; o__[Rhodothermales]; f__Rhodothermaceae; g__; s__	k__Bacteria;p__Bacteroidetes;c__Bacteroidetes Incertae Sedis;o__Order II;f__Rhodothermaceae;g__uncultured;s__uncultured Bacteroidetes bacterium
OTU1314	k__Bacteria; p__Proteobacteria; c__Alphaproteobacteria; o__Rhodospirillales; f__Acetobacteraceae; g__; s__	k__Bacteria;p__Proteobacteria;c__Alphaproteobacteria;o__Rhodospirillales;f__Acetobacteraceae;g__uncultured;s__uncultured Acetobacteraceae bacterium
OTU1315	k__Bacteria; p__Actinobacteria; c__Thermoleophilia; o__Solirubrobacterales; f__Conexibacteraceae; g__; s__	k__Bacteria;p__Actinobacteria;c__Thermoleophilia;o__Solirubrobacterales;f__YNPFFP1
OTU1316	k__Bacteria; p__Firmicutes; c__Bacilli; o__Lactobacillales; f__Lactobacillaceae; g__Lactobacillus; s__	k__Bacteria;p__Firmicutes;c__Bacilli;o__Lactobacillales;f__Lactobacillaceae;g__Lactobacillus;Ambiguous_taxa
OTU1317	k__Bacteria; p__Proteobacteria; c__Betaproteobacteria; o__Burkholderiales; f__Oxalobacteraceae; g__; s__	k__Bacteria;p__Proteobacteria;c__Betaproteobacteria;o__Burkholderiales;f__Oxalobacteraceae;g__Massilia;Ambiguous_taxa
OTU1318	k__Bacteria; p__Cyanobacteria; c__Nostocophycideae; o__Nostocales; f__Scytonemataceae; g__Scytonema; s__	k__Bacteria;p__Cyanobacteria;c__Cyanobacteria
OTU1319	k__Bacteria; p__[Thermi]; c__Deinococci; o__Deinococcales; f__Deinococcaceae; g__Deinococcus; s__	k__Bacteria;p__Deinococcus-Thermus;c__Deinococci;o__Deinococcales;f__Deinococcaceae;g__Deinococcus;s__uncultured bacterium
OTU132	k__Bacteria; p__Bacteroidetes; c__[Saprospirae]; o__[Saprospirales]; f__Chitinophagaceae; g__Flavisolibacter; s__	k__Bacteria;p__Bacteroidetes;c__Sphingobacteriia;o__Sphingobacteriales;f__Chitinophagaceae;g__uncultured
OTU1320	k__Bacteria; p__Chloroflexi; c__Ellin6529; o__; f__; g__; s__	k__Bacteria;p__Chloroflexi;c__KD4-96;o__uncultured bacterium;f__uncultured bacterium;g__uncultured bacterium;s__uncultured bacterium
OTU1321	k__Bacteria; p__Actinobacteria; c__Actinobacteria; o__Actinomycetales; f__Pseudonocardiaceae; g__Pseudonocardia; s__	k__Bacteria;p__Actinobacteria;c__Actinobacteria;o__Pseudonocardiales;f__Pseudonocardiaceae;g__Pseudonocardia;s__uncultured bacterium
OTU1322	Unassigned	Unassigned
OTU1323	Unassigned	Unassigned
OTU1324	k__Bacteria; p__Acidobacteria; c__Acidobacteriia; o__Acidobacteriales; f__Acidobacteriaceae; g__Edaphobacter; s__modestum	k__Bacteria;p__Acidobacteria;c__Acidobacteria;o__Acidobacteriales;f__Acidobacteriaceae (Subgroup 1);g__uncultured;s__uncultured bacterium
OTU1325	k__Bacteria; p__Firmicutes; c__Clostridia; o__Clostridiales; f__Ruminococcaceae; g__; s__	k__Bacteria;p__Firmicutes;c__Clostridia;o__Clostridiales;f__Ruminococcaceae;g__Ruminiclostridium 1;Ambiguous_taxa
OTU1326	k__Bacteria; p__Proteobacteria; c__Alphaproteobacteria; o__Rhizobiales; f__Methylocystaceae; g__; s__	k__Bacteria;p__Proteobacteria;c__Alphaproteobacteria;o__Rhizobiales;f__1174-901-12;g__uncultured bacterium;s__uncultured bacterium
OTU1327	k__Bacteria; p__Proteobacteria; c__Gammaproteobacteria; o__Pseudomonadales; f__Pseudomonadaceae; g__Pseudomonas; s__	k__Bacteria;p__Proteobacteria;c__Gammaproteobacteria;o__Pseudomonadales;f__Pseudomonadaceae;g__Pseudomonas
OTU1328	Unassigned	Unassigned
OTU1329	k__Bacteria; p__Proteobacteria; c__Alphaproteobacteria; o__; f__; g__; s__	k__Bacteria;p__Proteobacteria;c__Alphaproteobacteria;o__Rickettsiales;f__SM2D12;g__uncultured bacterium;s__uncultured bacterium

OTU133	k__Bacteria; p__Planctomycetes; c__Planctomycetia; o__Planctomycetales; f__Planctomycetaceae; g__Planctomyces; s__	k__Bacteria;p__Planctomycetes;c__Planctomycetacia;o__Planctomycetales;f__Planctomycetaceae;g__Planctomyces
OTU1330	k__Bacteria; p__Planctomycetes; c__Planctomycetia; o__Gemmatales; f__Gemmataceae; g__Gemmata; s__	k__Bacteria;p__Planctomycetes;c__Planctomycetacia;o__Planctomycetales;f__Planctomycetaceae;g__Gemmata;s__uncultured bacterium
OTU1331	k__Bacteria; p__Proteobacteria; c__Alphaproteobacteria; o__Rhodospirillales; f__Acetobacteraceae	k__Bacteria;p__Proteobacteria;c__Alphaproteobacteria;o__Rhodospirillales;f__Acetobacteraceae
OTU1332	k__Bacteria; p__Proteobacteria; c__Alphaproteobacteria; o__Rhizobiales; f__Phyllobacteriaceae	k__Bacteria;p__Proteobacteria;c__Alphaproteobacteria;o__Rhizobiales;f__Phyllobacteriaceae
OTU1333	Unassigned	Unassigned
OTU1334	k__Bacteria; p__Actinobacteria; c__Actinobacteria; o__Actinomycetales; f__Nocardioidaceae; g__Nocardioides; s__	k__Bacteria;p__Actinobacteria;c__Actinobacteria;o__Propionibacteriales;f__Nocardioidaceae;g__Nocardioides;Ambiguous_taxa
OTU1335	Unassigned	Unassigned
OTU1336	Unassigned	Unassigned
OTU1337	k__Bacteria; p__Firmicutes; c__Bacilli; o__Bacillales; f__; g__; s__	k__Bacteria;p__Firmicutes;c__Bacilli;o__Bacillales;f__Bacillaceae;g__Bacillus;Ambiguous_taxa
OTU1338	k__Bacteria; p__Proteobacteria; c__Alphaproteobacteria; o__Rhizobiales; f__Methylobacteriaceae; g__Methylobacterium; s__	k__Bacteria;p__Proteobacteria;c__Alphaproteobacteria;o__Rhizobiales;f__Methylobacteriaceae;g__Methylobacterium
OTU1339	k__Bacteria; p__TM7; c__TM7-1; o__; f__; g__; s__	k__Bacteria;p__Saccharibacteria;c__uncultured bacterium;o__uncultured bacterium;f__uncultured bacterium;g__uncultured bacterium;s__uncultured bacterium
OTU134	k__Bacteria; p__Cyanobacteria; c__Oscillatoriophycideae; o__Chroococcales; f__Xenococcaceae; g__; s__	k__Bacteria;p__Cyanobacteria;c__Cyanobacteria;o__uncultured;f__uncultured bacterium;g__uncultured bacterium;s__uncultured bacterium
OTU1340	k__Bacteria; p__Proteobacteria; c__Alphaproteobacteria; o__Rhizobiales; f__Rhizobiaceae; g__Agrobacterium; s__	k__Bacteria;p__Proteobacteria;c__Alphaproteobacteria;o__Rhizobiales;f__Rhizobiaceae;g__Rhizobium;Ambiguous_taxa
OTU1341	k__Bacteria; p__Proteobacteria; c__Alphaproteobacteria; o__Rhodospirillales; f__Acetobacteraceae; g__Gluconobacter; s__	k__Bacteria;p__Proteobacteria;c__Alphaproteobacteria;o__Rhodospirillales;f__Acetobacteraceae;g__Gluconobacter
OTU1342	k__Bacteria; p__Proteobacteria; c__Betaproteobacteria; o__; f__; g__; s__	k__Bacteria;p__Proteobacteria;c__Betaproteobacteria;o__TRA3-20
OTU1343	k__Bacteria; p__Bacteroidetes; c__Sphingobacteriia; o__Sphingobacteriales; f__Sphingobacteriaceae; g__; s__	k__Bacteria;p__Bacteroidetes;c__Sphingobacteriia;o__Sphingobacteriales;f__Sphingobacteriaceae;g__Mucilaginibacter;s__uncultured bacterium
OTU1344	Unassigned	Unassigned
OTU1345	k__Bacteria; p__Proteobacteria; c__Gammaproteobacteria; o__Pseudomonadales; f__Moraxellaceae; g__Acinetobacter; s__	k__Bacteria;p__Proteobacteria;c__Gammaproteobacteria;o__Pseudomonadales;f__Moraxellaceae;g__Acinetobacter
OTU1346	k__Bacteria; p__Actinobacteria; c__Actinobacteria; o__Actinomycetales; f__Microbacteriaceae; g__; s__	k__Bacteria;p__Actinobacteria;c__Actinobacteria;o__Micrococcales;f__Microbacteriaceae
OTU1347	Unassigned	Unassigned
OTU1348	k__Bacteria; p__Proteobacteria; c__Alphaproteobacteria; o__Rhizobiales; f__Methylocystaceae; g__; s__	k__Bacteria;p__Proteobacteria;c__Alphaproteobacteria;o__Rhizobiales;f__1174-901-12
OTU1349	k__Bacteria; p__Actinobacteria; c__Thermoleophilia; o__Gaiellales; f__Gaiellaceae; g__; s__	k__Bacteria;p__Actinobacteria;c__Thermoleophilia;o__Gaiellales;f__uncultured;g__uncultured bacterium;s__uncultured bacterium
OTU135	k__Bacteria; p__Bacteroidetes; c__Bacteroidia; o__Bacteroidales; f__Prevotellaceae; g__Prevotella; s__copri	k__Bacteria;p__Bacteroidetes;c__Bacteroidia;o__Bacteroidales;f__Prevotellaceae;g__Prevotella 9;s__uncultured bacterium

OTU1350	k__Bacteria; p__Proteobacteria; c__Alphaproteobacteria; o__Rhodospirillales; f__Acetobacteraceae; g__Gluconobacter; s__	k__Bacteria;p__Proteobacteria;c__Alphaproteobacteria;o__Rhodospirillales;f__Acetobacteraceae;g__Gluconobacter
OTU1351	k__Bacteria; p__Firmicutes; c__Bacilli; o__Lactobacillales; f__Lactobacillaceae; g__Lactobacillus; s__	k__Bacteria;p__Firmicutes;c__Bacilli;o__Lactobacillales;f__Lactobacillaceae;g__Lactobacillus
OTU1352	Unassigned	Unassigned
OTU1353	k__Bacteria; p__Actinobacteria; c__Actinobacteria; o__Actinomycetales; f__Nocardiaceae	k__Bacteria;p__Actinobacteria;c__Actinobacteria;o__Corynebacteriales;f__Nocardiaceae;g__Rhodococcus
OTU1354	Unassigned	Unassigned
OTU1355	k__Bacteria; p__Proteobacteria; c__Deltaproteobacteria; o__Bdellovibrionales; f__Bdellovibrionaceae; g__Bdellovibrio; s__	k__Bacteria;p__Proteobacteria;c__Deltaproteobacteria;o__Bdellovibrionales;f__Bdellovibrionaceae;g__Bdellovibrio
OTU1356	k__Bacteria; p__Proteobacteria; c__Alphaproteobacteria; o__Rhodospirillales; f__Rhodospirillaceae; g__; s__	k__Bacteria;p__Proteobacteria;c__Alphaproteobacteria;o__Rhodospirillales;f__MND8;g__uncultured bacterium;s__uncultured bacterium
OTU1357	Unassigned	Unassigned
OTU1358	k__Bacteria; p__Proteobacteria; c__Betaproteobacteria; o__; f__; g__; s__	k__Bacteria;p__Proteobacteria;c__Betaproteobacteria;o__Nitrosomonadales;f__Nitrosomonadaceae;g__uncultured
OTU1359	k__Bacteria; p__[Thermi]; c__Deinococci; o__Deinococcales; f__Deinococcaceae; g__Deinococcus; s__	k__Bacteria;p__Deinococcus-Thermus;c__Deinococci;o__Deinococcales;f__Deinococcaceae;g__Deinococcus
OTU136	k__Bacteria; p__Proteobacteria; c__Alphaproteobacteria; o__Sphingomonadales; f__Sphingomonadaceae; g__Sphingomonas; s__yabuuchiae	k__Bacteria;p__Proteobacteria;c__Alphaproteobacteria;o__Sphingomonadales;f__Sphingomonadaceae;g__Sphingomonas;Ambiguous_taxa
OTU1360	k__Bacteria; p__Cyanobacteria; c__Synechococcophycideae; o__Synechococcales; f__Acaryochloridaceae; g__Acaryochloris; s__	k__Bacteria;p__Cyanobacteria;c__Chloroplast;o__uncultured cyanobacterium;f__uncultured cyanobacterium;g__uncultured cyanobacterium;s__uncultured cyanobacterium
OTU1361	k__Bacteria; p__Firmicutes; c__Clostridia; o__Clostridiales; f__Clostridiaceae; g__; s__	k__Bacteria;p__Firmicutes;c__Clostridia;o__Clostridiales;f__Clostridiaceae 1;g__Clostridium sensu stricto 1
OTU1362	k__Bacteria; p__Bacteroidetes; c__[Saprospirae]; o__[Saprospirales]; f__Chitinophagaceae; g__; s__	k__Bacteria;p__Bacteroidetes;c__Sphingobacteriia;o__Sphingobacteriales;f__Chitinophagaceae;g__uncultured
OTU1363	k__Bacteria; p__Acidobacteria; c__Acidobacteriia; o__Acidobacteriales; f__Acidobacteriaceae; g__; s__	k__Bacteria;p__Acidobacteria;c__Acidobacteria;o__Acidobacteriales;f__Acidobacteriaceae (Subgroup 1);g__uncultured
OTU1364	k__Bacteria; p__Firmicutes; c__Bacilli; o__Lactobacillales; f__; g__; s__	k__Bacteria;p__Firmicutes;c__Bacilli;o__Lactobacillales;f__Lactobacillaceae;g__Lactobacillus;s__uncultured bacterium
OTU1365	k__Bacteria; p__Proteobacteria; c__TA18; o__PHOS-HD29; f__; g__; s__	k__Bacteria;p__Proteobacteria;c__Deltaproteobacteria;o__SAR324 clade(Marine group B);f__uncultured bacterium;g__uncultured bacterium;s__uncultured bacterium
OTU1366	Unassigned	Unassigned
OTU1367	k__Bacteria; p__Firmicutes; c__Bacilli; o__Lactobacillales; f__Lactobacillaceae; g__Lactobacillus; s__	k__Bacteria;p__Firmicutes;c__Bacilli;o__Lactobacillales;f__Lactobacillaceae;g__Lactobacillus;Ambiguous_taxa
OTU1368	k__Bacteria; p__Proteobacteria; c__Deltaproteobacteria; o__Desulfovibrionales; f__Desulfovibrionaceae; g__Desulfovibrio; s__	k__Bacteria;p__Proteobacteria;c__Deltaproteobacteria;o__Desulfovibrionales;f__Desulfovibrionaceae;g__Desulfovibrio;Ambiguous_taxa
OTU1369	k__Bacteria; p__Actinobacteria; c__Actinobacteria; o__Actinomycetales; f__Geodermatophilaceae; g__; s__	k__Bacteria;p__Actinobacteria;c__Actinobacteria;o__Frankiales;f__Geodermatophilaceae;g__Geodermatophilus;Ambiguous_taxa

OTU137	k__Bacteria; p__Proteobacteria; c__Alphaproteobacteria; o__Rhodospirillales; f__Acetobacteraceae; g__; s__	k__Bacteria;p__Proteobacteria;c__Alphaproteobacteria;o__Rhodospirillales;f__Acetobacteraceae;g__uncultured
OTU1370	Unassigned	Unassigned
OTU1371	k__Bacteria; p__Proteobacteria; c__Alphaproteobacteria; o__Sphingomonadales; f__Sphingomonadaceae; g__Sphingomonas; s__	k__Bacteria;p__Proteobacteria;c__Alphaproteobacteria;o__Sphingomonadales;f__Sphingomonadaceae;g__Sphingomonas;Ambiguous_taxa
OTU1372	Unassigned	Unassigned
OTU1373	k__Bacteria; p__Firmicutes; c__Clostridia; o__Clostridiales; f__Clostridiaceae; g__; s__	k__Bacteria;p__Firmicutes;c__Clostridia;o__Clostridiales;f__Peptostreptococcaceae
OTU1374	k__Bacteria; p__Proteobacteria; c__Alphaproteobacteria; o__Rhodospirillales; f__Acetobacteraceae; g__Roseomonas; s__mucosa	k__Bacteria;p__Proteobacteria;c__Alphaproteobacteria;o__Rhodospirillales;f__Acetobacteraceae;g__Roseomonas
OTU1375	Unassigned	k__Bacteria;p__Chloroflexi;c__Chloroflexia;o__Chloroflexales;f__Roseiflexaceae;g__Roseiflexus;s__uncultured bacterium
OTU1376	k__Bacteria; p__Acidobacteria; c__Acidobacteriia; o__Acidobacteriales; f__Acidobacteriaceae; g__; s__	k__Bacteria;p__Acidobacteria;c__Acidobacteria;o__Acidobacteriales;f__Acidobacteriaceae (Subgroup 1);g__Granulicella
OTU1377	k__Bacteria; p__Proteobacteria; c__Alphaproteobacteria; o__Rhodospirillales; f__Acetobacteraceae; g__; s__	k__Bacteria;p__Proteobacteria;c__Alphaproteobacteria;o__Rhodospirillales;f__Acetobacteraceae;g__uncultured;s__uncultured Acetobacteraceae bacterium
OTU1378	k__Bacteria; p__Firmicutes; c__Bacilli; o__Lactobacillales; f__Lactobacillaceae; g__; s__	k__Bacteria;p__Firmicutes;c__Bacilli;o__Lactobacillales;f__Lactobacillaceae;g__Lactobacillus;s__Lactobacillus plantarum
OTU1379	k__Bacteria; p__Acidobacteria; c__Solibacteres; o__Solibacterales; f__; g__; s__	k__Bacteria;p__Acidobacteria;c__Solibacteres;o__Solibacterales;f__Solibacteraceae (Subgroup 3);g__Bryobacter
OTU138	k__Bacteria; p__Proteobacteria; c__Betaproteobacteria; o__Burkholderiales; f__Burkholderiaceae; g__; s__	k__Bacteria;p__Proteobacteria;c__Betaproteobacteria;o__Burkholderiales;f__Burkholderiaceae;g__Burkholderia-Paraburkholderia;s__uncultured bacterium
OTU1380	Unassigned	Unassigned
OTU1381	k__Bacteria; p__Proteobacteria; c__Alphaproteobacteria; o__Rhodospirillales; f__Acetobacteraceae; g__; s__	k__Bacteria;p__Proteobacteria;c__Alphaproteobacteria;o__Rhodospirillales;f__Acetobacteraceae;g__uncultured;s__uncultured bacterium
OTU1382	Unassigned	Unassigned
OTU1383	k__Bacteria; p__Proteobacteria; c__Betaproteobacteria; o__Burkholderiales; f__Comamonadaceae	k__Bacteria;p__Proteobacteria;c__Betaproteobacteria;o__Burkholderiales;f__Comamonadaceae;g__uncultured
OTU1384	Unassigned	Unassigned
OTU1385	k__Bacteria; p__Proteobacteria; c__Alphaproteobacteria; o__Rhizobiales; f__Hyphomicrobiaceae; g__Devosia; s__	k__Bacteria;p__Proteobacteria;c__Alphaproteobacteria;o__Rhizobiales;f__Hyphomicrobiaceae;g__Devosia
OTU1386	k__Bacteria; p__Verrucomicrobia; c__[Spartobacteria]; o__[Chthoniobacterales]; f__[Chthoniobacteraceae]; g__; s__	k__Bacteria;p__Verrucomicrobia;c__Spartobacteria;o__Chthoniobacterales;f__LD29;g__uncultured bacterium;s__uncultured bacterium
OTU1387	Unassigned	Unassigned
OTU1388	k__Bacteria; p__Actinobacteria; c__Actinobacteria; o__Actinomycetales; f__Nocardioidaceae; g__Nocardioides; s__	k__Bacteria;p__Actinobacteria;c__Actinobacteria;o__Propionibacteriales;f__Nocardioidaceae;g__Nocardioides;Ambiguous_taxa
OTU1389	k__Bacteria; p__Proteobacteria; c__Gammaproteobacteria; o__Xanthomonadales; f__Xanthomonadaceae; g__Lysobacter; s__	k__Bacteria;p__Proteobacteria;c__Gammaproteobacteria;o__Xanthomonadales;f__Xanthomonadaceae;g__Lysobacter;Ambiguous_taxa
OTU139	k__Bacteria; p__Acidobacteria; c__Acidobacteria-6; o__iii1-15; f__; g__; s__	k__Bacteria;p__Acidobacteria;c__Subgroup 6
OTU1390	k__Bacteria; p__Actinobacteria; c__Actinobacteria; o__Actinomycetales; f__Kineosporiaceae	k__Bacteria;p__Actinobacteria;c__Actinobacteria;o__Kineosporiales;f__Kineosporiaceae;g__Kineococcus
OTU1391	Unassigned	Unassigned

OTU1392	k__Bacteria; p__Verrucomicrobia; c__[Spartobacteria]; o__[Chthoniobacterales]; f__[Chthoniobacteraceae]; g__Ellin506; s__	k__Bacteria;p__Verrucomicrobia;c__Spartobacteria;o__Chthoniobacterales;f__Chthoniobacteraceae;g__Chthoniobacter;s__uncultured bacterium
OTU1393	k__Bacteria; p__Proteobacteria; c__Alphaproteobacteria; o__Sphingomonadales; f__Sphingomonadaceae; g__Sphingobium; s__	k__Bacteria;p__Proteobacteria;c__Alphaproteobacteria;o__Sphingomonadales;f__Sphingomonadaceae;g__Sphingobium
OTU1394	Unassigned	Unassigned
OTU1395	k__Bacteria; p__Bacteroidetes; c__Flavobacteriia; o__Flavobacteriales; f__Flavobacteriaceae; g__Flavobacterium; s__	k__Bacteria;p__Bacteroidetes;c__Flavobacteriia;o__Flavobacteriales;f__Flavobacteriaceae;g__Flavobacterium;s__uncultured bacterium
OTU1396	k__Bacteria; p__Proteobacteria; c__Gammaproteobacteria; o__Pseudomonadales; f__Pseudomonadaceae; g__; s__	k__Bacteria;p__Proteobacteria;c__Gammaproteobacteria;o__Pseudomonadales;f__Pseudomonadaceae;g__Pseudomonas
OTU14	k__Bacteria; p__Actinobacteria; c__Actinobacteria; o__Actinomycetales; f__Microbacteriaceae; g__Curtobacterium; s__	k__Bacteria;p__Actinobacteria;c__Actinobacteria;o__Micrococcales;f__Microbacteriaceae
OTU140	k__Bacteria; p__Proteobacteria; c__Alphaproteobacteria; o__Rhizobiales; f__Hyphomicrobiaceae; g__Rhodoplanes; s__	k__Bacteria;p__Proteobacteria;c__Alphaproteobacteria;o__Rhizobiales;f__Xanthobacteraceae;g__Pseudolabrys;s__uncultured bacterium
OTU141	k__Bacteria; p__Proteobacteria; c__Gammaproteobacteria; o__Pseudomonadales; f__Pseudomonadaceae; g__Pseudomonas; s__	k__Bacteria;p__Proteobacteria;c__Gammaproteobacteria;o__Pseudomonadales;f__Pseudomonadaceae;g__Pseudomonas;Ambiguous_taxa
OTU142	k__Bacteria; p__Firmicutes; c__Bacilli; o__Lactobacillales; f__Lactobacillaceae; g__Lactobacillus; s__	k__Bacteria;p__Firmicutes;c__Bacilli;o__Lactobacillales;f__Lactobacillaceae;g__Lactobacillus
OTU143	k__Bacteria; p__Actinobacteria; c__Actinobacteria; o__Actinomycetales; f__Nocardiaceae; g__Nocardia; s__	k__Bacteria;p__Actinobacteria;c__Actinobacteria;o__Corynebacteriales;f__nbr16a11;g__uncultured bacterium;s__uncultured bacterium
OTU144	Unassigned	Unassigned
OTU145	k__Bacteria; p__Bacteroidetes; c__Cytophagia; o__Cytophagales; f__Cytophagaceae; g__Hymenobacter; s__	k__Bacteria;p__Bacteroidetes;c__Cytophagia;o__Cytophagales;f__Cytophagaceae;g__Hymenobacter
OTU146	k__Bacteria; p__Firmicutes; c__Erysipelotrichi; o__Erysipelotrichales; f__Erysipelotrichaceae; g__cc_115; s__	k__Bacteria;p__Firmicutes;c__Erysipelotrichia;o__Erysipelotrichales;f__Erysipelotrichaceae;g__uncultured;Ambiguous_taxa
OTU147	k__Bacteria; p__Proteobacteria; c__Betaproteobacteria; o__Burkholderiales; f__Comamonadaceae; g__; s__	k__Bacteria;p__Proteobacteria;c__Betaproteobacteria;o__Burkholderiales
OTU148	k__Bacteria; p__Bacteroidetes; c__[Saprospirae]; o__[Saprospirales]; f__Chitinophagaceae; g__; s__	k__Bacteria;p__Bacteroidetes;c__Sphingobacteriia;o__Sphingobacteriales;f__Chitinophagaceae;g__Ferruginibacter;s__uncultured bacterium
OTU149	Unassigned	Unassigned
OTU15	k__Bacteria; p__Acidobacteria; c__Acidobacteria-6; o__iii1-15; f__; g__; s__	k__Bacteria;p__Acidobacteria;c__Subgroup 6;o__uncultured bacterium;f__uncultured bacterium;g__uncultured bacterium;s__uncultured bacterium
OTU150	Unassigned	Unassigned
OTU151	k__Bacteria; p__Proteobacteria; c__Gammaproteobacteria; o__Enterobacteriales; f__Enterobacteriaceae; g__; s__	k__Bacteria;p__Proteobacteria;c__Gammaproteobacteria;o__Enterobacteriales;f__Enterobacteriaceae;g__Enterobacter
OTU152	k__Bacteria; p__Tenericutes; c__Mollicutes; o__Mycoplasmatales; f__Mycoplasmataceae; g__Mycoplasma	k__Bacteria;p__Tenericutes;c__Mollicutes;o__Mycoplasmatales;f__Mycoplasmataceae;g__Mycoplasma;Ambiguous_taxa
OTU153	k__Bacteria; p__Actinobacteria; c__Actinobacteria; o__Actinomycetales; f__Nocardiaceae; g__Nocardia; s__	k__Bacteria;p__Actinobacteria;c__Actinobacteria;o__Corynebacteriales;f__nbr16a11;g__uncultured bacterium;s__uncultured bacterium
OTU154	k__Bacteria; p__Proteobacteria; c__Alphaproteobacteria; o__Sphingomonadales; f__Sphingomonadaceae	k__Bacteria;p__Proteobacteria;c__Alphaproteobacteria;o__Sphingomonadales;f__Sphingomonadaceae;g__Sphingomonas

OTU155	k__Bacteria; p__Tenericutes; c__Mollicutes; o__Entomoplasmatales; f__; g__; s__	k__Bacteria;p__Tenericutes;c__Mollicutes;o__Entomoplasmatales;f__Spiroplasmataceae;g__Spiroplasma
OTU156	k__Bacteria; p__Proteobacteria; c__Alphaproteobacteria; o__Rhizobiales	k__Bacteria;p__Proteobacteria;c__Alphaproteobacteria;o__Rhizobiales;f__Hyphomicrobiaceae;g__Dichotomicrobium
OTU157	Unassigned	Unassigned
OTU158	Unassigned	Unassigned
OTU159	k__Bacteria; p__Proteobacteria; c__Alphaproteobacteria; o__Sphingomonadales; f__Sphingomonadaceae	k__Bacteria;p__Proteobacteria;c__Alphaproteobacteria;o__Sphingomonadales;f__Sphingomonadaceae;g__Sphingomonas;s__uncultured bacterium
OTU16	k__Bacteria; p__TM7; c__TM7-1; o__; f__; g__; s__	k__Bacteria;p__Saccharibacteria;c__uncultured bacterium;o__uncultured bacterium;f__uncultured bacterium;g__uncultured bacterium;s__uncultured bacterium
OTU160	k__Bacteria; p__Chloroflexi; c__Gitt-GS-136; o__; f__; g__; s__	k__Bacteria;p__Chloroflexi;c__Gitt-GS-136;o__uncultured bacterium;f__uncultured bacterium;g__uncultured bacterium;s__uncultured bacterium
OTU161	k__Bacteria; p__Verrucomicrobia; c__[Spartobacteria]; o__[Chthoniobacterales]; f__[Chthoniobacteraceae]; g__DA101; s__	k__Bacteria;p__Verrucomicrobia;c__Spartobacteria;o__Chthoniobacterales;f__DA101 soil group;g__uncultured bacterium;s__uncultured bacterium
OTU162	k__Bacteria; p__TM7; c__TM7-1; o__; f__; g__; s__	k__Bacteria;p__Saccharibacteria;c__uncultured bacterium;o__uncultured bacterium;f__uncultured bacterium;g__uncultured bacterium;s__uncultured bacterium
OTU163	k__Bacteria; p__Proteobacteria; c__Alphaproteobacteria; o__Rhizobiales; f__Bradyrhizobiaceae; g__; s__	k__Bacteria;p__Proteobacteria;c__Alphaproteobacteria;o__Rhizobiales;f__Methylobacteriaceae;g__uncultured;s__uncultured bacterium
OTU164	k__Bacteria; p__Bacteroidetes; c__Sphingobacteriia; o__Sphingobacteriales; f__Sphingobacteriaceae; g__; s__	k__Bacteria;p__Bacteroidetes;c__Sphingobacteriia;o__Sphingobacteriales;f__Sphingobacteriaceae;g__Mucilaginibacter;Ambiguous_taxa
OTU165	k__Bacteria; p__Proteobacteria; c__Betaproteobacteria; o__Burkholderiales; f__Oxalobacteraceae; g__; s__	k__Bacteria;p__Proteobacteria;c__Betaproteobacteria;o__Burkholderiales;f__Oxalobacteraceae;g__Massilia
OTU166	k__Bacteria; p__Proteobacteria; c__Alphaproteobacteria; o__Rhodospirillales; f__Acetobacteraceae; g__; s__	k__Bacteria;p__Proteobacteria;c__Alphaproteobacteria;o__Rhodospirillales;f__Acetobacteraceae
OTU167	k__Bacteria; p__Firmicutes; c__Bacilli; o__Lactobacillales; f__Lactobacillaceae; g__Lactobacillus; s__	k__Bacteria;p__Firmicutes;c__Bacilli;o__Lactobacillales;f__Lactobacillaceae;g__Lactobacillus
OTU168	k__Bacteria; p__Armatimonadetes; c__Armatimonadia; o__Armatimonadales; f__Armatimonadaceae; g__; s__	k__Bacteria;p__Armatimonadetes;c__Armatimonadia;o__Armatimonadales;f__uncultured bacterium;g__uncultured bacterium;s__uncultured bacterium
OTU169	k__Bacteria; p__Bacteroidetes; c__[Saprospirae]; o__[Saprospirales]; f__Chitinophagaceae; g__Flavisolibacter; s__	k__Bacteria;p__Bacteroidetes;c__Sphingobacteriia;o__Sphingobacteriales;f__Chitinophagaceae;g__Flavisolibacter;s__uncultured bacterium
OTU17	k__Bacteria; p__Gemmatimonadetes; c__Gemm-1; o__; f__; g__; s__	k__Bacteria;p__Gemmatimonadetes;c__Gemmatimonadetes;o__Gemmatimonadales;f__Gemmatimonadaceae;g__uncultured
OTU170	k__Bacteria; p__Proteobacteria; c__Alphaproteobacteria; o__Rhizobiales	k__Bacteria;p__Proteobacteria;c__Alphaproteobacteria;o__Rhizobiales;f__Bartonellaceae;g__Bartonella;Ambiguous_taxa
OTU171	k__Bacteria; p__Proteobacteria; c__Betaproteobacteria; o__Burkholderiales; f__Comamonadaceae; g__Tepidimonas; s__	k__Bacteria;p__Proteobacteria;c__Betaproteobacteria;o__Burkholderiales;f__Comamonadaceae;g__Tepidimonas
OTU172	k__Bacteria; p__Proteobacteria; c__Alphaproteobacteria; o__Rhodobacterales; f__Rhodobacteraceae; g__Rubellimicrobium; s__	k__Bacteria;p__Proteobacteria;c__Alphaproteobacteria;o__Rhodobacterales;f__Rhodobacteraceae;g__Rubellimicrobium;s__uncultured bacterium

OTU173	k__Bacteria; p__Acidobacteria; c__Acidobacteria-6; o__iii1-15; f__RB40; g__; s__	k__Bacteria;p__Acidobacteria;c__Subgroup 6;o__uncultured bacterium;f__uncultured bacterium;g__uncultured bacterium;s__uncultured bacterium
OTU174	k__Bacteria; p__Proteobacteria; c__Alphaproteobacteria; o__Sphingomonadales; f__Sphingomonadaceae; g__Sphingomonas; s__wittichii	k__Bacteria;p__Proteobacteria;c__Alphaproteobacteria;o__Sphingomonadales;f__Sphingomonadaceae;g__Sphingomonas;Ambiguous_taxa
OTU175	k__Bacteria; p__Firmicutes; c__Bacilli; o__Lactobacillales; f__Lactobacillaceae; g__Lactobacillus; s__	k__Bacteria;p__Firmicutes;c__Bacilli;o__Lactobacillales;f__Lactobacillaceae;g__Lactobacillus
OTU176	k__Bacteria; p__Proteobacteria; c__Alphaproteobacteria; o__Rhizobiales; f__Hyphomicrobiaceae; g__Rhodoplanes; s__	k__Bacteria;p__Proteobacteria;c__Alphaproteobacteria;o__Rhizobiales
OTU177	k__Bacteria; p__Cyanobacteria; c__Synechococcophycideae; o__Pseudanabaenales; f__Pseudanabaenaceae; g__Leptolyngbya; s__	k__Bacteria;p__Cyanobacteria;c__Cyanobacteria;o__SubsectionIII;f__FamilyI;g__Leptolyngbya
OTU178	k__Bacteria; p__Cyanobacteria; c__ML635J-21; o__; f__; g__; s__	k__Bacteria;p__Cyanobacteria;c__ML635J-21
OTU179	k__Bacteria; p__Firmicutes; c__Bacilli; o__Lactobacillales; f__Lactobacillaceae; g__Lactobacillus; s__	k__Bacteria;p__Firmicutes;c__Bacilli;o__Lactobacillales;f__Lactobacillaceae;g__Lactobacillus;Ambiguous_taxa
OTU18	k__Bacteria; p__Proteobacteria; c__Alphaproteobacteria; o__Rhizobiales; f__Methylobacteriaceae; g__; s__	k__Bacteria;p__Proteobacteria;c__Alphaproteobacteria;o__Rhizobiales;f__Methylobacteriaceae;g__Methylobacterium;Ambiguous_taxa
OTU180	k__Bacteria; p__Cyanobacteria; c__Synechococcophycideae; o__Pseudanabaenales; f__Pseudanabaenaceae; g__Leptolyngbya; s__	k__Bacteria;p__Cyanobacteria;c__Cyanobacteria;o__SubsectionIII;f__FamilyI;g__Leptolyngbya
OTU181	k__Bacteria; p__Planctomycetes; c__Phycisphaerae; o__WD2101; f__; g__; s__	k__Bacteria;p__Planctomycetes;c__Phycisphaerae;o__Tepidisphaerales;f__Tepidisphaeraceae;g__uncultured bacterium;s__uncultured bacterium
OTU182	k__Bacteria; p__Proteobacteria; c__Alphaproteobacteria; o__Rhodobacterales; f__Rhodobacteraceae; g__Paracoccus; s__	k__Bacteria;p__Proteobacteria;c__Alphaproteobacteria;o__Rhodobacterales;f__Rhodobacteraceae;g__Paracoccus;Ambiguous_taxa
OTU183	k__Bacteria; p__Proteobacteria; c__Epsilonproteobacteria; o__Campylobacterales; f__Helicobacteraceae; g__Flexispira	k__Bacteria;p__Proteobacteria;c__Epsilonproteobacteria;o__Campylobacterales;f__Helicobacteraceae;g__Helicobacter;Ambiguous_taxa
OTU184	Unassigned	Unassigned
OTU185	k__Bacteria; p__Cyanobacteria; c__Oscillatoriophycideae; o__Chroococcales; f__Xenococcaceae; g__; s__	k__Bacteria;p__Cyanobacteria;c__Cyanobacteria;o__SubsectionII;f__FamilyII;g__Chroococcidiopsis
OTU186	k__Bacteria; p__Acidobacteria; c__Acidobacteriia; o__Acidobacteriales; f__Acidobacteriaceae; g__; s__	k__Bacteria;p__Acidobacteria;c__Acidobacteria;o__Acidobacteriales;f__Acidobacteriaceae (Subgroup 1);g__Edaphobacter;Ambiguous_taxa
OTU187	k__Bacteria; p__Actinobacteria; c__Actinobacteria; o__Actinomycetales; f__Streptomycetaceae; g__Streptomyces; s__	k__Bacteria;p__Actinobacteria;c__Actinobacteria;o__Streptomycetales;f__Streptomycetaceae;g__Streptomyces
OTU188	k__Bacteria; p__Firmicutes; c__Bacilli; o__Bacillales; f__Bacillaceae; g__Bacillus; s__	k__Bacteria;p__Firmicutes;c__Bacilli;o__Bacillales;f__Bacillaceae;g__Bacillus
OTU189	k__Bacteria; p__Proteobacteria; c__Alphaproteobacteria; o__Sphingomonadales; f__Sphingomonadaceae; g__; s__	k__Bacteria;p__Proteobacteria;c__Alphaproteobacteria;o__Sphingomonadales;f__Erythrobacteraceae;g__uncultured
OTU19	k__Bacteria; p__Actinobacteria; c__Rubrobacteria; o__Rubrobacterales; f__Rubrobacteraceae; g__Rubrobacter; s__	k__Bacteria;p__Actinobacteria;c__Rubrobacteria;o__Rubrobacterales;f__Rubrobacteriaceae;g__Rubrobacter;s__uncultured bacterium
OTU190	k__Bacteria; p__Proteobacteria; c__Alphaproteobacteria; o__Rhizobiales; f__Rhodobiaceae; g__Afifella; s__	k__Bacteria;p__Proteobacteria;c__Alphaproteobacteria;o__Rhizobiales;f__JG34-KF-361;g__uncultured bacterium;s__uncultured bacterium
OTU191	k__Bacteria; p__Actinobacteria; c__Actinobacteria; o__Actinomycetales; f__Intrasporangiaceae; g__Phycicoccus; s__	k__Bacteria;p__Actinobacteria;c__Actinobacteria;o__Micrococcales;f__Intrasporangiaceae;g__Phycicoccus

OTU192	k__Bacteria; p__Proteobacteria; c__Alphaproteobacteria; o__Rhizobiales	k__Bacteria;p__Proteobacteria;c__Alphaproteobacteria;o__Rhizobiales;f__Rhizobiaceae
OTU193	k__Bacteria; p__Proteobacteria; c__Alphaproteobacteria; o__Rhodospirillales; f__Acetobacteraceae; g__; s__	k__Bacteria;p__Proteobacteria;c__Alphaproteobacteria;o__Rhodospirillales;f__Acetobacteraceae;g__Roseomonas
OTU194	k__Bacteria; p__Proteobacteria; c__Gammaproteobacteria; o__Enterobacteriales; f__Enterobacteriaceae	k__Bacteria;p__Proteobacteria;c__Gammaproteobacteria;o__Enterobacteriales;f__Enterobacteriaceae
OTU195	k__Bacteria; p__Proteobacteria; c__Alphaproteobacteria; o__Rhodobacterales; f__Rhodobacteraceae; g__Rubellimicrobium; s__	k__Bacteria;p__Proteobacteria;c__Alphaproteobacteria;o__Rhodobacterales;f__Rhodobacteraceae;g__Rubellimicrobium;s__uncultured bacterium
OTU196	k__Bacteria; p__Proteobacteria; c__Alphaproteobacteria; o__Rhizobiales; f__Bradyrhizobiaceae; g__; s__	k__Bacteria;p__Proteobacteria;c__Alphaproteobacteria;o__Rhizobiales;f__Methylobacteriaceae
OTU197	k__Bacteria; p__Proteobacteria; c__Gammaproteobacteria; o__Xanthomonadales; f__Xanthomonadaceae; g__Lysobacter; s__	k__Bacteria;p__Proteobacteria;c__Gammaproteobacteria;o__Xanthomonadales;f__Xanthomonadaceae;g__Lysobacter
OTU198	k__Bacteria; p__Proteobacteria; c__Alphaproteobacteria; o__Rhodobacterales; f__Rhodobacteraceae; g__Rhodobacter; s__	k__Bacteria;p__Proteobacteria;c__Alphaproteobacteria;o__Rhodobacterales;f__Rhodobacteraceae;g__Falsirhodobacter;Ambiguous_taxa
OTU199	k__Bacteria; p__Proteobacteria; c__Gammaproteobacteria; o__Legionellales; f__; g__; s__	k__Bacteria;p__Proteobacteria;c__Gammaproteobacteria;o__HTA4;Ambiguous_taxa;Ambiguous_taxa;Ambiguous_taxa
OTU2	k__Bacteria; p__Actinobacteria; c__Thermoleophilia; o__Solirubrobacterales; f__Patulibacteraceae; g__; s__	k__Bacteria;p__Actinobacteria;c__Thermoleophilia;o__Solirubrobacterales;f__Solirubrobacteraceae;g__Solirubrobacter
OTU20	k__Bacteria; p__Bacteroidetes; c__Cytophagia; o__Cytophagales; f__Cytophagaceae; g__Adhaeribacter; s__	k__Bacteria;p__Bacteroidetes;c__Cytophagia;o__Cytophagales;f__Cytophagaceae;g__Adhaeribacter;s__uncultured bacterium
OTU200	k__Bacteria; p__Bacteroidetes; c__[Saprospirae]; o__[Saprospirales]; f__Chitinophagaceae; g__; s__	k__Bacteria;p__Bacteroidetes;c__Sphingobacteriia;o__Sphingobacteriales;f__Chitinophagaceae
OTU201	k__Bacteria; p__Proteobacteria; c__Gammaproteobacteria; o__Xanthomonadales; f__Xanthomonadaceae; g__Lysobacter; s__	k__Bacteria;p__Proteobacteria;c__Gammaproteobacteria;o__Xanthomonadales;f__Xanthomonadaceae;g__Lysobacter;Ambiguous_taxa
OTU202	Unassigned	Unassigned
OTU203	k__Bacteria; p__Planctomycetes; c__Phycisphaerae; o__WD2101; f__; g__; s__	k__Bacteria;p__Planctomycetes;c__Phycisphaerae;o__Tepidisphaerales;f__Tepidisphaeraceae;g__uncultured bacterium;s__uncultured bacterium
OTU204	k__Bacteria; p__Firmicutes; c__Clostridia; o__Clostridiales; f__[Tissierellaceae]; g__Anaerococcus; s__	k__Bacteria;p__Firmicutes;c__Clostridia;o__Clostridiales;f__Family XI;g__Anaerococcus;s__uncultured bacterium
OTU205	k__Bacteria; p__TM7; c__TM7-3; o__; f__; g__; s__	k__Bacteria;p__Saccharibacteria;c__uncultured bacterium;o__uncultured bacterium;f__uncultured bacterium;g__uncultured bacterium;s__uncultured bacterium
OTU206	Unassigned	Unassigned
OTU207	k__Bacteria; p__Armatimonadetes; c__Armatimonadia; o__Armatimonadales; f__Armatimonadaceae; g__; s__	k__Bacteria;p__Armatimonadetes;c__Armatimonadia;o__Armatimonadales;Ambiguous_taxa;Ambiguous_taxa;Ambiguous_taxa
OTU208	k__Bacteria; p__Proteobacteria; c__Alphaproteobacteria; o__Rhodospirillales; f__Acetobacteraceae; g__; s__	k__Bacteria;p__Proteobacteria;c__Alphaproteobacteria;o__Rhodospirillales;f__Acetobacteraceae
OTU209	k__Bacteria; p__Proteobacteria; c__Gammaproteobacteria; o__Legionellales; f__Coxiellaceae; g__; s__	k__Bacteria;p__Proteobacteria;c__Gammaproteobacteria;o__Legionellales;f__Coxiellaceae;g__uncultured
OTU21	k__Bacteria; p__Proteobacteria; c__Alphaproteobacteria; o__Rhizobiales; f__Rhizobiaceae; g__Rhizobium; s__	k__Bacteria;p__Proteobacteria;c__Alphaproteobacteria;o__Rhizobiales;f__Rhizobiaceae;g__Rhizobium;s__Rhizobium etli

OTU210	k__Bacteria; p__Acidobacteria; c__Acidobacteria-6; o__iii1-15; f__; g__; s__	k__Bacteria;p__Acidobacteria;c__Subgroup 6;o__uncultured bacterium;f__uncultured bacterium;g__uncultured bacterium;s__uncultured bacterium
OTU211	Unassigned	Unassigned
OTU212	k__Bacteria; p__Proteobacteria; c__Alphaproteobacteria; o__Sphingomonadales; f__Sphingomonadaceae; g__Kaistobacter; s__	k__Bacteria;p__Proteobacteria;c__Alphaproteobacteria;o__Sphingomonadales;f__Sphingomonadaceae;g__Sphingomonas
OTU213	k__Bacteria; p__Planctomycetes; c__Planctomycetia; o__Pirellulales; f__Pirellulaceae; g__; s__	k__Bacteria;p__Planctomycetes;c__Planctomycetacia;o__Planctomycetales;f__Planctomycetaceae;g__Pir4 lineage;s__uncultured bacterium
OTU214	k__Bacteria; p__Armatimonadetes; c__Armatimonadia; o__Armatimonadales; f__Armatimonadaceae; g__; s__	k__Bacteria;p__Armatimonadetes;c__Armatimonadia;o__Armatimonadales;f__uncultured bacterium;g__uncultured bacterium;s__uncultured bacterium
OTU215	k__Bacteria; p__Acidobacteria; c__Solibacteres; o__Solibacterales; f__Solibacteraceae; g__Candidatus Solibacter; s__	k__Bacteria;p__Acidobacteria;c__Solibacteres;o__Solibacterales;f__Solibacteraceae (Subgroup 3);g__Candidatus Solibacter
OTU216	Unassigned	Unassigned
OTU217	k__Bacteria; p__Proteobacteria; c__Alphaproteobacteria; o__Rhodospirillales; f__Acetobacteraceae; g__; s__	k__Bacteria;p__Proteobacteria;c__Alphaproteobacteria;o__Rhodospirillales;f__Acetobacteraceae;g__uncultured
OTU218	k__Bacteria; p__Proteobacteria; c__Alphaproteobacteria; o__Sphingomonadales; f__Sphingomonadaceae; g__Sphingomonas; s__wittichii	k__Bacteria;p__Proteobacteria;c__Alphaproteobacteria;o__Sphingomonadales;f__Sphingomonadaceae;g__Sphingomonas
OTU219	k__Bacteria; p__Firmicutes; c__Bacilli; o__Lactobacillales; f__Leuconostocaceae; g__; s__	k__Bacteria;p__Firmicutes;c__Bacilli;o__Lactobacillales;f__Leuconostocaceae;g__Weissella;s__Weissella confusa
OTU22	k__Bacteria; p__Verrucomicrobia; c__[Spartobacteria]; o__[Chthoniobacterales]; f__[Chthoniobacteraceae]; g__Ellin506; s__	k__Bacteria;p__Verrucomicrobia;c__Spartobacteria;o__Chthoniobacterales;f__Chthoniobacteraceae;g__Chthoniobacter;s__uncultured Verrucomicrobia bacterium
OTU220	k__Bacteria; p__[Thermi]; c__Deinococci; o__Deinococcales; f__Deinococcaceae; g__Deinococcus; s__	k__Bacteria;p__Deinococcus-Thermus;c__Deinococci;o__Deinococcales;f__Deinococcaceae;g__Deinococcus;s__uncultured bacterium
OTU221	Unassigned	Unassigned
OTU222	k__Bacteria; p__Proteobacteria; c__Alphaproteobacteria; o__Rhizobiales; f__Methylobacteriaceae; g__Methylobacterium; s__	k__Bacteria;p__Proteobacteria;c__Alphaproteobacteria;o__Rhizobiales;f__Methylobacteriaceae;g__Methylobacterium;s__uncultured bacterium
OTU223	k__Bacteria; p__Proteobacteria; c__Alphaproteobacteria; o__Rhizobiales; f__Aurantimonadaceae; g__; s__	k__Bacteria;p__Proteobacteria;c__Alphaproteobacteria;o__Rhizobiales;f__Aurantimonadaceae;g__Aureimonas;Ambiguous_taxa
OTU224	Unassigned	Unassigned
OTU225	k__Bacteria; p__Bacteroidetes; c__Cytophagia; o__Cytophagales; f__Cytophagaceae; g__; s__	k__Bacteria;p__Bacteroidetes;c__Cytophagia;o__Cytophagales;f__Cytophagaceae;g__Chryseolinea
OTU226	k__Bacteria; p__Proteobacteria; c__Deltaproteobacteria; o__Myxococcales; f__; g__; s__	k__Bacteria;p__Proteobacteria;c__Deltaproteobacteria;o__Myxococcales;f__Haliangiaceae;g__Haliangium;s__uncultured bacterium
OTU227	k__Bacteria; p__Proteobacteria; c__Gammaproteobacteria; o__Pseudomonadales; f__Pseudomonadaceae; g__Pseudomonas; s__	k__Bacteria;p__Proteobacteria;c__Gammaproteobacteria;o__Pseudomonadales;f__Pseudomonadaceae;g__Pseudomonas
OTU228	Unassigned	Unassigned
OTU229	k__Bacteria; p__TM7; c__TM7-1; o__; f__; g__; s__	k__Bacteria;p__Saccharibacteria;c__uncultured bacterium;o__uncultured bacterium;f__uncultured bacterium;g__uncultured bacterium;s__uncultured bacterium

OTU23	k__Bacteria; p__Acidobacteria; c__Acidobacteriia; o__Acidobacteriales; f__Koribacteraceae; g__; s__	k__Bacteria;p__Acidobacteria;c__Acidobacteria;o__Acidobacteriales;f__Acidobacteriaceae (Subgroup 1);g__uncultured
OTU230	k__Bacteria; p__Cyanobacteria; c__Synechococcophycideae; o__Pseudanabaenales; f__Pseudanabaenaceae; g__Leptolyngbya; s__	k__Bacteria;p__Cyanobacteria;c__Cyanobacteria;o__SubsectionIII;f__FamilyI;g__uncultured;s__uncultured bacterium
OTU231	k__Bacteria; p__Proteobacteria; c__Alphaproteobacteria; o__Rhizobiales; f__Hyphomicrobiaceae; g__; s__	k__Bacteria;p__Proteobacteria;c__Alphaproteobacteria;o__Rhizobiales;f__Hyphomicrobiaceae
OTU232	k__Bacteria; p__Proteobacteria; c__Epsilonproteobacteria; o__Campylobacterales; f__Campylobacteraceae; g__Arcobacter; s__	k__Bacteria;p__Proteobacteria;c__Epsilonproteobacteria;o__Campylobacterales;f__Campylobacteraceae;g__Arcobacter;s__uncultured bacterium
OTU233	k__Bacteria; p__Proteobacteria; c__Alphaproteobacteria; o__Rhodospirillales; f__Rhodospirillaceae; g__; s__	k__Bacteria;p__Proteobacteria;c__Alphaproteobacteria;o__Rhodospirillales;f__Rhodospirillaceae;g__Niveispirillum
OTU234	k__Bacteria; p__Actinobacteria; c__Actinobacteria; o__Actinomycetales; f__Corynebacteriaceae; g__Corynebacterium; s__	k__Bacteria;p__Actinobacteria;c__Actinobacteria;o__Corynebacteriales;f__Corynebacteriaceae;g__Corynebacterium 1;s__uncultured bacterium
OTU235	Unassigned	Unassigned
OTU236	k__Bacteria; p__Cyanobacteria; c__Oscillatoriophycideae; o__Chroococcales; f__Xenococcaceae; g__; s__	k__Bacteria;p__Cyanobacteria;c__Cyanobacteria;o__uncultured;f__uncultured bacterium;g__uncultured bacterium;s__uncultured bacterium
OTU237	Unassigned	Unassigned
OTU238	k__Bacteria; p__Proteobacteria; c__Alphaproteobacteria; o__Rhodospirillales; f__Acetobacteraceae; g__Gluconobacter; s__	k__Bacteria;p__Proteobacteria;c__Alphaproteobacteria;o__Rhodospirillales;f__Acetobacteraceae
OTU239	k__Bacteria; p__Bacteroidetes; c__Sphingobacteriia; o__Sphingobacteriales; f__Sphingobacteriaceae; g__; s__	k__Bacteria;p__Bacteroidetes;c__Sphingobacteriia;o__Sphingobacteriales;f__Sphingobacteriaceae;g__Mucilaginibacter;Ambiguous_taxa
OTU24	k__Bacteria; p__Actinobacteria; c__Actinobacteria; o__Actinomycetales; f__Gordoniaceae; g__Gordonia; s__	k__Bacteria;p__Actinobacteria;c__Actinobacteria;o__Corynebacteriales;f__Nocardiaceae;g__Gordonia;Ambiguous_taxa
OTU240	k__Bacteria; p__Gemmatimonadetes; c__Gemmatimonadetes; o__Gemmatimonadales; f__; g__; s__	k__Bacteria;p__Gemmatimonadetes;c__Gemmatimonadetes;o__Gemmatimonadales;f__Gemmatimonadaceae;g__Gemmatirosa;s__uncultured bacterium
OTU241	Unassigned	Unassigned
OTU242	k__Bacteria; p__Proteobacteria; c__Betaproteobacteria; o__Methylophilales; f__Methylophilaceae	k__Bacteria;p__Proteobacteria;c__Betaproteobacteria;o__Methylophilales;f__Methylophilaceae;g__Methylotenera;s__uncultured bacterium
OTU243	k__Bacteria; p__Proteobacteria; c__TA18; o__PHOS-HD29; f__; g__; s__	k__Bacteria;p__Proteobacteria;c__Deltaproteobacteria;o__SAR324 clade(Marine group B);f__uncultured bacterium;g__uncultured bacterium;s__uncultured bacterium
OTU244	Unassigned	Unassigned
OTU245	k__Bacteria; p__Actinobacteria; c__Thermoleophilia; o__Solirubrobacterales; f__; g__; s__	k__Bacteria;p__Actinobacteria;c__Thermoleophilia;o__Solirubrobacterales;f__Elev-16S-1332;g__uncultured bacterium;s__uncultured bacterium
OTU246	Unassigned	Unassigned
OTU247	k__Bacteria; p__Actinobacteria; c__Actinobacteria; o__Actinomycetales; f__Mycobacteriaceae; g__Mycobacterium; s__	k__Bacteria;p__Actinobacteria;c__Actinobacteria;o__Corynebacteriales;f__Mycobacteriaceae;g__Mycobacterium
OTU248	k__Bacteria; p__Proteobacteria; c__Betaproteobacteria; o__Burkholderiales; f__Comamonadaceae; g__Rubrivivax; s__	k__Bacteria;p__Proteobacteria;c__Betaproteobacteria;o__Burkholderiales;f__Comamonadaceae;g__Comamonas
OTU249	k__Bacteria; p__Firmicutes; c__Bacilli; o__Bacillales; f__Bacillaceae; g__Anoxybacillus; s__kestanbolensis	k__Bacteria;p__Firmicutes;c__Bacilli;o__Bacillales;f__Bacillaceae;g__Anoxybacillus

OTU25	k__Bacteria; p__Verrucomicrobia; c__[Spartobacteria]; o__[Chthoniobacterales]; f__[Chthoniobacteraceae]; g__Candidatus Xiphinematobacter; s__	k__Bacteria;p__Verrucomicrobia;c__Spartobacteria;o__Chthoniobacterales;f__Chthoniobacterales Incertae Sedis;g__Terrimicrobium;Ambiguous_taxa
OTU250	Unassigned	Unassigned
OTU251	Unassigned	Unassigned
OTU252	Unassigned	Unassigned
OTU253	Unassigned	Unassigned
OTU254	k__Bacteria; p__Proteobacteria; c__Alphaproteobacteria; o__Rhodospirillales; f__Acetobacteraceae; g__; s__	k__Bacteria;p__Proteobacteria;c__Alphaproteobacteria;o__Rhodospirillales;f__Acetobacteraceae;g__Acidiphilium
OTU255	k__Bacteria; p__Proteobacteria; c__Alphaproteobacteria; o__Rhizobiales; f__Methylocystaceae; g__; s__	k__Bacteria;p__Proteobacteria;c__Alphaproteobacteria;o__Rhizobiales;f__1174-901-12
OTU256	k__Bacteria; p__Proteobacteria; c__Alphaproteobacteria; o__Rhizobiales; f__Rhizobiaceae; g__Agrobacterium; s__	k__Bacteria;p__Proteobacteria;c__Alphaproteobacteria;o__Rhizobiales;f__Rhizobiaceae;g__Rhizobium;Ambiguous_taxa
OTU257	k__Bacteria; p__TM7; c__TM7-1; o__; f__; g__; s__	k__Bacteria;p__Saccharibacteria;c__uncultured bacterium;o__uncultured bacterium;f__uncultured bacterium;g__uncultured bacterium;s__uncultured bacterium
OTU258	k__Bacteria; p__Proteobacteria; c__Gammaproteobacteria; o__Enterobacteriales; f__Enterobacteriaceae; g__; s__	k__Bacteria;p__Proteobacteria;c__Gammaproteobacteria;o__Enterobacteriales;f__Enterobacteriaceae;g__Enterobacter
OTU259	k__Bacteria; p__Proteobacteria; c__Alphaproteobacteria; o__Sphingomonadales; f__Erythrobacteraceae; g__; s__	k__Bacteria;p__Proteobacteria;c__Alphaproteobacteria;o__Sphingomonadales;f__Erythrobacteraceae;g__Altererythrobacter
OTU26	k__Bacteria; p__Actinobacteria; c__Acidimicrobiia; o__Acidimicrobiales; f__; g__; s__	k__Bacteria;p__Actinobacteria;c__Acidimicrobiia;o__Acidimicrobiales;f__uncultured;g__uncultured bacterium;s__uncultured bacterium
OTU260	Unassigned	Unassigned
OTU261	k__Bacteria; p__Acidobacteria; c__Sva0725; o__Sva0725; f__; g__; s__	k__Bacteria;p__Acidobacteria;c__Holophagae;o__Subgroup 10;f__ABS-19;g__uncultured bacterium;s__uncultured bacterium
OTU262	k__Bacteria; p__Bacteroidetes; c__[Saprospirae]; o__[Saprospirales]; f__Chitinophagaceae; g__Flavisolibacter; s__	k__Bacteria;p__Bacteroidetes;c__Sphingobacteriia;o__Sphingobacteriales;f__Chitinophagaceae;g__Flavisolibacter
OTU263	k__Bacteria; p__Tenericutes; c__Mollicutes; o__Entomoplasmatales; f__Entomoplasmataceae; g__Mesoplasma; s__	k__Bacteria;p__Tenericutes;c__Mollicutes;o__Entomoplasmatales;f__Entomoplasmataceae;g__Mesoplasma;Ambiguous_taxa
OTU264	k__Bacteria; p__Cyanobacteria; c__Synechococcophycideae; o__Pseudanabaenales; f__Pseudanabaenaceae; g__Arthronema; s__	k__Bacteria;p__Cyanobacteria;c__Cyanobacteria;o__SubsectionIII;f__FamilyI;g__Arthronema
OTU265	k__Bacteria; p__Proteobacteria; c__Gammaproteobacteria; o__Pasteurellales; f__Pasteurellaceae; g__Haemophilus; s__	k__Bacteria;p__Proteobacteria;c__Gammaproteobacteria;o__Pasteurellales;f__Pasteurellaceae;g__Haemophilus;s__uncultured bacterium
OTU266	k__Bacteria; p__Proteobacteria; c__Gammaproteobacteria; o__Enterobacteriales; f__Enterobacteriaceae; g__Serratia; s__	k__Bacteria;p__Proteobacteria;c__Gammaproteobacteria;o__Enterobacteriales;f__Enterobacteriaceae
OTU267	Unassigned	Unassigned
OTU268	k__Bacteria; p__Actinobacteria; c__Actinobacteria; o__Actinomycetales; f__Gordoniaceae; g__Gordonia; s__	k__Bacteria;p__Actinobacteria;c__Actinobacteria;o__Corynebacteriales;f__Nocardiaceae;g__Gordonia;Ambiguous_taxa
OTU269	Unassigned	Unassigned
OTU27	k__Bacteria; p__Proteobacteria; c__Alphaproteobacteria; o__Sphingomonadales; f__Sphingomonadaceae; g__Sphingomonas; s__	k__Bacteria;p__Proteobacteria;c__Alphaproteobacteria;o__Sphingomonadales;f__Sphingomonadaceae;g__Sphingomonas;s__uncultured bacterium

OTU270	k__Bacteria; p__Acidobacteria; c__Acidobacteriia; o__Acidobacteriales; f__Acidobacteriaceae; g__; s__	k__Bacteria;p__Acidobacteria;c__Acidobacteria;o__Acidobacteriales;f__Acidobacteriaceae (Subgroup 1);Ambiguous_taxa
OTU271	k__Bacteria; p__Actinobacteria; c__Actinobacteria; o__Actinomycetales; f__Nocardioidaceae; g__; s__	k__Bacteria;p__Actinobacteria;c__Actinobacteria;o__Propionibacteriales;f__Nocardioidaceae;g__Nocardioides;s__uncultured bacterium
OTU272	k__Bacteria; p__Bacteroidetes; c__Cytophagia; o__Cytophagales; f__Cytophagaceae; g__Hymenobacter; s__	k__Bacteria;p__Bacteroidetes;c__Cytophagia;o__Cytophagales;f__Cytophagaceae;g__Hymenobacter;Ambiguous_taxa
OTU273	k__Bacteria; p__Armatimonadetes; c__Armatimonadia; o__Armatimonadales; f__Armatimonadaceae; g__; s__	k__Bacteria;p__Armatimonadetes;c__Armatimonadia;o__Armatimonadales;f__uncultured bacterium;g__uncultured bacterium;s__uncultured bacterium
OTU274	Unassigned	Unassigned
OTU275	k__Bacteria; p__Cyanobacteria; c__Oscillatoriophycideae; o__Chroococcales; f__; g__; s__	k__Bacteria;p__Cyanobacteria;c__Cyanobacteria;o__SubsectionI;f__FamilyI
OTU276	k__Bacteria; p__Acidobacteria; c__PAUC37f; o__; f__; g__; s__	k__Bacteria;p__Acidobacteria;c__Subgroup 11;o__uncultured Acidobacteria bacterium;f__uncultured Acidobacteria bacterium;g__uncultured Acidobacteria bacterium;s__uncultured Acidobacteria bacterium
OTU277	k__Bacteria; p__Proteobacteria; c__Betaproteobacteria; o__Burkholderiales; f__Comamonadaceae; g__Delftia; s__	k__Bacteria;p__Proteobacteria;c__Betaproteobacteria;o__Burkholderiales;f__Comamonadaceae;g__Delftia;s__uncultured bacterium
OTU278	k__Bacteria; p__Bacteroidetes; c__Bacteroidia; o__Bacteroidales; f__Prevotellaceae; g__Prevotella; s__melaninogenica	k__Bacteria;p__Bacteroidetes;c__Bacteroidia;o__Bacteroidales;f__Prevotellaceae;g__Prevotella 7;s__uncultured bacterium
OTU279	k__Bacteria; p__Bacteroidetes; c__Sphingobacteriia; o__Sphingobacteriales; f__Sphingobacteriaceae; g__; s__	k__Bacteria;p__Bacteroidetes;c__Sphingobacteriia;o__Sphingobacteriales;f__Sphingobacteriaceae;g__Mucilaginibacter;s__uncultured bacterium
OTU28	Unassigned	Unassigned
OTU280	k__Bacteria; p__Proteobacteria; c__Alphaproteobacteria; o__Caulobacterales; f__Caulobacteraceae; g__Phenylobacterium; s__	k__Bacteria;p__Proteobacteria;c__Alphaproteobacteria;o__Caulobacterales;f__Caulobacteraceae;g__Phenylobacterium;Ambiguous_taxa
OTU281	k__Bacteria; p__Proteobacteria; c__Alphaproteobacteria; o__Rhodospirillales; f__Acetobacteraceae	k__Bacteria;p__Proteobacteria;c__Alphaproteobacteria;o__Rhodospirillales;f__Acetobacteraceae;g__Gluconobacter
OTU282	k__Bacteria; p__Cyanobacteria; c__Nostocophycideae; o__Stigonematales; f__Rivulariaceae; g__Calothrix; s__	k__Bacteria;p__Cyanobacteria;c__Cyanobacteria;o__SubsectionIV;f__FamilyII;g__Calothrix;Ambiguous_taxa
OTU283	k__Bacteria; p__Bacteroidetes; c__Sphingobacteriia; o__Sphingobacteriales; f__Sphingobacteriaceae; g__; s__	k__Bacteria;p__Bacteroidetes;c__Sphingobacteriia;o__Sphingobacteriales;f__Sphingobacteriaceae;g__Sphingobacterium;Ambiguous_taxa
OTU284	k__Bacteria; p__Proteobacteria; c__Alphaproteobacteria; o__Rhizobiales; f__; g__; s__	k__Bacteria;p__Proteobacteria;c__Alphaproteobacteria;o__Rhizobiales;f__Rhizobiales Incertae Sedis;g__Bauldia;s__uncultured bacterium
OTU285	k__Bacteria; p__Proteobacteria; c__Betaproteobacteria; o__Burkholderiales; f__Burkholderiaceae; g__Burkholderia; s__	k__Bacteria;p__Proteobacteria;c__Betaproteobacteria;o__Burkholderiales;f__Burkholderiaceae;g__Burkholderia-Paraburkholderia
OTU286	k__Bacteria; p__Proteobacteria; c__Betaproteobacteria; o__Burkholderiales; f__Burkholderiaceae; g__Burkholderia; s__	k__Bacteria;p__Proteobacteria;c__Betaproteobacteria;o__Burkholderiales;f__Burkholderiaceae;g__Burkholderia-Paraburkholderia
OTU287	k__Bacteria; p__Actinobacteria; c__Actinobacteria; o__Actinomycetales; f__Dietziaceae; g__Dietzia; s__	k__Bacteria;p__Actinobacteria;c__Actinobacteria;o__Corynebacteriales;f__Dietziaceae;g__Dietzia;s__uncultured bacterium
OTU288	k__Bacteria; p__Proteobacteria; c__Gammaproteobacteria; o__Pseudomonadales; f__Moraxellaceae; g__; s__	k__Bacteria;p__Proteobacteria;c__Gammaproteobacteria;o__Pseudomonadales;f__Moraxellaceae;g__Psychrobacter
OTU289	k__Bacteria; p__Firmicutes; c__Clostridia; o__Clostridiales; f__Veillonellaceae; g__Veillonella; s__dispar	k__Bacteria;p__Firmicutes;c__Negativicutes;o__Selenomonadales;f__Veillonellaceae;g__Veillonella;s__uncultured bacterium

OTU29	Unassigned	k__Bacteria;p__Proteobacteria;c__Alphaproteobacteria;o__Rhodospirillales;f__Acetobacteraceae;g__uncultured;s__uncultured bacterium
OTU290	k__Bacteria; p__Cyanobacteria; c__Nostocophycideae; o__Nostocales; f__Scytonemataceae; g__; s__	k__Bacteria;p__Cyanobacteria;c__Cyanobacteria;o__SubsectionI;f__FamilyI;g__Brasilonema;Ambiguous_taxa
OTU291	k__Bacteria; p__TM7; c__TM7-3; o__EW055; f__; g__; s__	k__Bacteria;p__Saccharibacteria;c__uncultured bacterium;o__uncultured bacterium;f__uncultured bacterium;g__uncultured bacterium;s__uncultured bacterium
OTU292	Unassigned	Unassigned
OTU293	k__Bacteria; p__Acidobacteria; c__Acidobacteriia; o__Acidobacteriales; f__Acidobacteriaceae; g__Terriglobus; s__	k__Bacteria;p__Acidobacteria;c__Acidobacteria;o__Acidobacteriales;f__Acidobacteriaceae (Subgroup 1);g__Terriglobus;Ambiguous_taxa
OTU294	k__Bacteria; p__Planctomycetes; c__Planctomycetia; o__Pirellulales; f__Pirellulaceae; g__Pirellula; s__	k__Bacteria;p__Planctomycetes;c__Planctomycetacia;o__Planctomycetales;f__Planctomycetaceae;g__Pirellula;s__uncultured bacterium
OTU295	k__Bacteria; p__Acidobacteria; c__[Chloracidobacteria]; o__RB41; f__Ellin6075; g__; s__	k__Bacteria;p__Acidobacteria;c__Blastocatellia;o__Blastocatellales;f__Blastocatellaceae (Subgroup 4);g__Blastocatella;s__uncultured bacterium
OTU296	k__Bacteria; p__Actinobacteria; c__Actinobacteria; o__Actinomycetales; f__; g__; s__	k__Bacteria;p__Actinobacteria;c__Actinobacteria;o__Frankiales;f__Acidothermaceae;g__Acidothermus;Ambiguous_taxa
OTU297	k__Bacteria; p__Proteobacteria; c__Gammaproteobacteria; o__Pasteurellales; f__Pasteurellaceae	k__Bacteria;p__Proteobacteria;c__Gammaproteobacteria;o__Pasteurellales;f__Pasteurellaceae;g__Aggregatibacter;s__uncultured bacterium
OTU298	k__Bacteria; p__Proteobacteria; c__Alphaproteobacteria; o__Rhodospirillales; f__Acetobacteraceae; g__; s__	k__Bacteria;p__Proteobacteria;c__Alphaproteobacteria;o__Rhodospirillales;f__Acetobacteraceae
OTU299	k__Bacteria; p__Gemmatimonadetes; c__Gemm-5; o__; f__; g__; s__	k__Bacteria;p__Gemmatimonadetes;c__S0134 terrestrial group;o__uncultured bacterium;f__uncultured bacterium;g__uncultured bacterium;s__uncultured bacterium
OTU3	Unassigned	Unassigned
OTU30	Unassigned	Unassigned
OTU300	k__Bacteria; p__Bacteroidetes; c__Cytophagia; o__Cytophagales; f__Cytophagaceae; g__Spirosoma; s__	k__Bacteria;p__Bacteroidetes;c__Cytophagia;o__Cytophagales;f__Cytophagaceae;g__Spirosoma
OTU301	k__Bacteria; p__Actinobacteria; c__Actinobacteria; o__Actinomycetales; f__Pseudonocardiaceae; g__Amycolatopsis; s__	k__Bacteria;p__Actinobacteria;c__Actinobacteria;o__Pseudonocardiales;f__Pseudonocardiaceae;g__Amycolatopsis
OTU302	k__Bacteria; p__Proteobacteria; c__Alphaproteobacteria; o__Rhizobiales; f__Phyllobacteriaceae	k__Bacteria;p__Proteobacteria;c__Alphaproteobacteria;o__Rhizobiales;f__Phyllobacteriaceae;g__Aquamicrobium
OTU303	k__Bacteria; p__Bacteroidetes; c__[Saprospirae]; o__[Saprospirales]; f__Chitinophagaceae; g__; s__	k__Bacteria;p__Bacteroidetes;c__Sphingobacteriia;o__Sphingobacteriales;f__Chitinophagaceae;g__Ferruginibacter;Ambiguous_taxa
OTU304	k__Bacteria; p__Proteobacteria; c__Alphaproteobacteria; o__Rhizobiales; f__Hyphomicrobiaceae; g__Rhodoplanes; s__	k__Bacteria;p__Proteobacteria;c__Alphaproteobacteria;o__Rhizobiales;f__Xanthobacteraceae;g__Variibacter
OTU305	k__Bacteria; p__Proteobacteria; c__Alphaproteobacteria; o__Sphingomonadales; f__Sphingomonadaceae; g__; s__	k__Bacteria;p__Proteobacteria;c__Alphaproteobacteria;o__Sphingomonadales;f__Sphingomonadaceae;g__Sphingomonas
OTU306	k__Bacteria; p__Proteobacteria; c__Gammaproteobacteria; o__Enterobacteriales; f__Enterobacteriaceae; g__Erwinia; s__	k__Bacteria;p__Proteobacteria;c__Gammaproteobacteria;o__Enterobacteriales;f__Enterobacteriaceae;g__Pantoea
OTU307	k__Bacteria; p__Proteobacteria; c__Alphaproteobacteria; o__Caulobacterales; f__Caulobacteraceae; g__; s__	k__Bacteria;p__Proteobacteria;c__Alphaproteobacteria;o__Caulobacterales;f__Caulobacteraceae;g__Brevundimonas

OTU308	k__Bacteria; p__Proteobacteria; c__Alphaproteobacteria; o__Rhizobiales; f__Beijerinckiaceae; g__; s__	k__Bacteria;p__Proteobacteria;c__Alphaproteobacteria;o__Rhizobiales
OTU309	Unassigned	Unassigned
OTU31	k__Bacteria; p__Proteobacteria; c__Alphaproteobacteria; o__Rhodospirillales; f__Acetobacteraceae	k__Bacteria;p__Proteobacteria;c__Alphaproteobacteria;o__Rhodospirillales;f__Acetobacteraceae;g__uncultured;Ambiguous_taxa
OTU310	k__Bacteria; p__Proteobacteria; c__Alphaproteobacteria; o__Rhizobiales; f__Bradyrhizobiaceae; g__; s__	k__Bacteria;p__Proteobacteria;c__Alphaproteobacteria;o__Rhizobiales;f__Methylobacteriaceae;g__uncultured;s__uncultured bacterium
OTU311	k__Bacteria; p__Bacteroidetes; c__Cytophagia; o__Cytophagales; f__Cytophagaceae; g__Spirosoma; s__	k__Bacteria;p__Bacteroidetes;c__Cytophagia;o__Cytophagales;f__Cytophagaceae;g__Spirosoma
OTU312	Unassigned	Unassigned
OTU313	k__Bacteria; p__Proteobacteria; c__Betaproteobacteria; o__Burkholderiales; f__Comamonadaceae; g__; s__	k__Bacteria;p__Proteobacteria;c__Betaproteobacteria;o__Burkholderiales;f__Comamonadaceae;Ambiguous_taxa;Ambiguous_taxa
OTU314	k__Bacteria; p__Actinobacteria; c__Actinobacteria; o__Actinomycetales; f__Pseudonocardiaceae; g__Pseudonocardia; s__	k__Bacteria;p__Actinobacteria;c__Actinobacteria;o__Pseudonocardiales;f__Pseudonocardiaceae;g__Pseudonocardia
OTU315	k__Bacteria; p__Verrucomicrobia; c__[Pedosphaerae]; o__[Pedosphaerales]; f__Ellin517; g__; s__	k__Bacteria;p__Verrucomicrobia;c__OPB35 soil group;o__uncultured bacterium;f__uncultured bacterium;g__uncultured bacterium;s__uncultured bacterium
OTU316	k__Bacteria; p__Cyanobacteria; c__Oscillatoriophycideae; o__Chroococcales; f__; g__; s__	k__Bacteria;p__Cyanobacteria;c__Cyanobacteria;o__SubsectionI;f__FamilyI
OTU317	k__Bacteria; p__Cyanobacteria; c__Synechococcophycideae; o__Pseudanabaenales; f__Pseudanabaenaceae; g__Leptolyngbya; s__	k__Bacteria;p__Cyanobacteria;c__Cyanobacteria;o__SubsectionIII;f__FamilyI;g__Leptolyngbya;s__uncultured bacterium
OTU318	k__Bacteria; p__Acidobacteria; c__[Chloracidobacteria]; o__RB41; f__Ellin6075; g__; s__	k__Bacteria;p__Acidobacteria;c__Blastocatellia;o__Blastocatellales;f__Blastocatellaceae (Subgroup 4);g__Blastocatella
OTU319	Unassigned	Unassigned
OTU32	k__Bacteria; p__Cyanobacteria; c__Nostocophycideae; o__Nostocales; f__Scytonemataceae; g__; s__	k__Bacteria;p__Cyanobacteria;c__Cyanobacteria;o__SubsectionV;f__FamilyI;g__Stigonema;Ambiguous_taxa
OTU320	k__Bacteria; p__Proteobacteria; c__Alphaproteobacteria; o__Rhodospirillales; f__Acetobacteraceae; g__; s__	k__Bacteria;p__Proteobacteria;c__Alphaproteobacteria;o__Rhodospirillales;f__Acetobacteraceae
OTU321	k__Bacteria; p__Proteobacteria; c__Alphaproteobacteria; o__Rhizobiales; f__Beijerinckiaceae; g__Beijerinckia; s__	k__Bacteria;p__Proteobacteria;c__Alphaproteobacteria;o__Rhizobiales;f__Beijerinckiaceae
OTU322	k__Bacteria; p__Proteobacteria; c__Alphaproteobacteria; o__Rhodospirillales; f__Acetobacteraceae; g__; s__	k__Bacteria;p__Proteobacteria;c__Alphaproteobacteria;o__Rhodospirillales;f__Acetobacteraceae;g__Acidiphilium
OTU323	k__Bacteria; p__OD1; c__ZB2; o__; f__; g__; s__	k__Bacteria;p__Parcubacteria
OTU324	k__Bacteria; p__Proteobacteria; c__Alphaproteobacteria; o__Rhodospirillales; f__Acetobacteraceae; g__Gluconobacter; s__	k__Bacteria;p__Proteobacteria;c__Alphaproteobacteria;o__Rhodospirillales;f__Acetobacteraceae;g__Gluconobacter
OTU325	Unassigned	Unassigned
OTU326	k__Bacteria; p__Planctomycetes; c__Phycisphaerae; o__WD2101; f__; g__; s__	k__Bacteria;p__Planctomycetes;c__Phycisphaerae;o__Tepidisphaerales;f__Tepidisphaeraceae;g__uncultured bacterium;s__uncultured bacterium
OTU327	k__Bacteria; p__Proteobacteria; c__Alphaproteobacteria; o__Sphingomonadales; f__Sphingomonadaceae; g__; s__	k__Bacteria;p__Proteobacteria;c__Alphaproteobacteria;o__Sphingomonadales;f__7B-8;g__uncultured bacterium;s__uncultured bacterium
OTU328	k__Bacteria; p__Proteobacteria; c__Betaproteobacteria; o__Burkholderiales; f__Oxalobacteraceae; g__Janthinobacterium; s__	k__Bacteria;p__Proteobacteria;c__Betaproteobacteria;o__Burkholderiales;f__Oxalobacteraceae;g__Duganella

OTU329	k__Bacteria; p__Proteobacteria; c__Alphaproteobacteria; o__Rhodospirillales; f__Acetobacteraceae; g__; s__	k__Bacteria;p__Proteobacteria;c__Alphaproteobacteria;o__Rhodospirillales;f__Acetobacteraceae
OTU33	k__Bacteria; p__Proteobacteria; c__Gammaproteobacteria; o__Enterobacteriales; f__Enterobacteriaceae; g__; s__	k__Bacteria;p__Proteobacteria;c__Gammaproteobacteria;o__Enterobacteriales;f__Enterobacteriaceae;g__Escherichia-Shigella;Ambiguous_taxa
OTU330	k__Bacteria; p__TM7; c__TM7-3; o__; f__; g__; s__	k__Bacteria;p__Saccharibacteria;c__uncultured bacterium;o__uncultured bacterium;f__uncultured bacterium;g__uncultured bacterium;s__uncultured bacterium
OTU331	k__Bacteria; p__Proteobacteria; c__Gammaproteobacteria; o__Pseudomonadales; f__Pseudomonadaceae; g__Pseudomonas; s__	k__Bacteria;p__Proteobacteria;c__Gammaproteobacteria;o__Pseudomonadales;f__Pseudomonadaceae;g__Pseudomonas
OTU332	k__Bacteria; p__Actinobacteria; c__Actinobacteria; o__Actinomycetales; f__Geodermatophilaceae	k__Bacteria;p__Actinobacteria;c__Actinobacteria;o__Frankiales;f__Geodermatophilaceae
OTU333	k__Bacteria; p__Proteobacteria; c__Gammaproteobacteria; o__Pseudomonadales; f__Moraxellaceae; g__Acinetobacter; s__	k__Bacteria;p__Proteobacteria;c__Gammaproteobacteria;o__Pseudomonadales;f__Moraxellaceae;g__Acinetobacter
OTU334	k__Bacteria; p__Proteobacteria; c__Gammaproteobacteria; o__Xanthomonadales; f__Xanthomonadaceae; g__Stenotrophomonas; s__	k__Bacteria;p__Proteobacteria;c__Gammaproteobacteria;o__Xanthomonadales;f__Xanthomonadaceae;g__Stenotrophomonas
OTU335	k__Bacteria; p__Proteobacteria; c__Alphaproteobacteria; o__Rhodospirillales; f__Acetobacteraceae; g__Acidocella; s__	k__Bacteria;p__Proteobacteria;c__Alphaproteobacteria;o__Rhodospirillales;f__Acetobacteraceae
OTU336	k__Bacteria; p__Proteobacteria; c__Alphaproteobacteria; o__Rhodospirillales; f__Acetobacteraceae; g__; s__	k__Bacteria;p__Proteobacteria;c__Alphaproteobacteria;o__Rhodospirillales;f__Acetobacteraceae;g__uncultured;s__uncultured bacterium
OTU337	k__Bacteria; p__Actinobacteria; c__Actinobacteria; o__Actinomycetales; f__Propionibacteriaceae; g__; s__	k__Bacteria;p__Actinobacteria;c__Actinobacteria;o__Propionibacteriales;f__Propionibacteriaceae;g__Microlunatus
OTU338	k__Bacteria; p__Actinobacteria; c__Actinobacteria; o__Actinomycetales; f__Geodermatophilaceae; g__Modestobacter; s__	k__Bacteria;p__Actinobacteria;c__Actinobacteria;o__Frankiales;f__Geodermatophilaceae
OTU339	k__Bacteria; p__Acidobacteria; c__Acidobacteria-6; o__iii1-15; f__; g__; s__	k__Bacteria;p__Acidobacteria;c__Subgroup 6
OTU34	k__Bacteria; p__Proteobacteria; c__Betaproteobacteria; o__Burkholderiales; f__Comamonadaceae; g__Methylibium; s__	k__Bacteria;p__Proteobacteria;c__Betaproteobacteria;o__Burkholderiales;f__Comamonadaceae
OTU340	k__Bacteria; p__Proteobacteria; c__Alphaproteobacteria; o__Rhizobiales; f__Bradyrhizobiaceae; g__; s__	k__Bacteria;p__Proteobacteria;c__Alphaproteobacteria;o__Rhizobiales;f__Bradyrhizobiaceae;g__Bradyrhizobium;s__uncultured bacterium
OTU341	k__Bacteria; p__Proteobacteria; c__Gammaproteobacteria; o__Pseudomonadales; f__Pseudomonadaceae; g__; s__	k__Bacteria;p__Proteobacteria;c__Gammaproteobacteria;o__Pseudomonadales;f__Pseudomonadaceae;g__Pseudomonas;Ambiguous_taxa
OTU342	k__Bacteria; p__TM7; c__TM7-1; o__; f__; g__; s__	k__Bacteria;p__Saccharibacteria;c__uncultured bacterium;o__uncultured bacterium;f__uncultured bacterium;g__uncultured bacterium;s__uncultured bacterium
OTU343	Unassigned	Unassigned
OTU344	k__Bacteria; p__Actinobacteria; c__Thermoleophilia; o__Gaiellales; f__Gaiellaceae; g__; s__	k__Bacteria;p__Actinobacteria;c__Thermoleophilia;o__Gaiellales;f__uncultured;Ambiguous_taxa;Ambiguous_taxa
OTU345	k__Bacteria; p__Cyanobacteria; c__Oscillatoriophycideae; o__Oscillatoriales; f__Phormidiaceae; g__Phormidium; s__animale	k__Bacteria;p__Cyanobacteria;c__Cyanobacteria;o__SubsectionIII;f__FamilyI
OTU346	k__Bacteria; p__TM7; c__TM7-1; o__; f__; g__; s__	k__Bacteria;p__Saccharibacteria;c__uncultured bacterium;o__uncultured bacterium;f__uncultured bacterium;g__uncultured bacterium;s__uncultured bacterium
OTU347	Unassigned	Unassigned

OTU348	Unassigned	Unassigned
OTU349	k__Bacteria; p__Bacteroidetes; c__Sphingobacteriia; o__Sphingobacteriales; f__Sphingobacteriaceae; g__Pedobacter; s__	k__Bacteria;p__Bacteroidetes;c__Sphingobacteriia;o__Sphingobacteriales;f__Sphingobacteriaceae;g__Pedobacter;Ambiguous_taxa
OTU35	k__Bacteria; p__TM7; c__TM7-1; o__; f__; g__; s__	k__Bacteria;p__Saccharibacteria;c__uncultured bacterium;o__uncultured bacterium;f__uncultured bacterium;g__uncultured bacterium;s__uncultured bacterium
OTU350	Unassigned	Unassigned
OTU351	Unassigned	Unassigned
OTU352	Unassigned	Unassigned
OTU353	Unassigned	Unassigned
OTU354	k__Bacteria; p__Acidobacteria; c__Solibacteres; o__Solibacterales; f__Solibacteraceae; g__; s__	k__Bacteria;p__Acidobacteria;c__Solibacteres;o__Solibacterales;f__Solibacteraceae (Subgroup 3);g__Bryobacter;s__uncultured bacterium
OTU355	k__Bacteria; p__Proteobacteria; c__Alphaproteobacteria; o__Rhizobiales; f__Methylocystaceae; g__; s__	k__Bacteria;p__Proteobacteria;c__Alphaproteobacteria;o__Rhizobiales;f__Roseiarcaceae;g__Roseiarcus
OTU356	k__Bacteria; p__Actinobacteria; c__Actinobacteria; o__Actinomycetales; f__Propionibacteriaceae; g__Propionibacterium; s__acnes	k__Bacteria;p__Actinobacteria;c__Actinobacteria;o__Propionibacteriales;f__Propionibacteriaceae;g__Propionibacterium;s__uncultured bacterium
OTU357	Unassigned	Unassigned
OTU358	k__Bacteria; p__Firmicutes; c__Bacilli; o__Lactobacillales; f__Lactobacillaceae; g__Lactobacillus; s__	k__Bacteria;p__Firmicutes;c__Bacilli;o__Lactobacillales;f__Lactobacillaceae;g__Lactobacillus
OTU359	k__Bacteria; p__Proteobacteria; c__Alphaproteobacteria; o__Rhizobiales; f__Methylocystaceae; g__; s__	k__Bacteria;p__Proteobacteria;c__Alphaproteobacteria;o__Rhizobiales;f__1174-901-12;g__uncultured bacterium;s__uncultured bacterium
OTU36	k__Bacteria; p__[Thermi]; c__Deinococci; o__Deinococcales; f__Deinococcaceae; g__Deinococcus; s__	k__Bacteria;p__Deinococcus-Thermus;c__Deinococci;o__Deinococcales;f__Deinococcaceae;g__Deinococcus;s__uncultured bacterium
OTU360	k__Bacteria; p__Proteobacteria; c__Alphaproteobacteria; o__Rhodospirillales; f__Acetobacteraceae; g__Gluconobacter; s__	k__Bacteria;p__Proteobacteria;c__Alphaproteobacteria;o__Rhodospirillales;f__Acetobacteraceae;g__uncultured
OTU361	k__Bacteria; p__Planctomycetes; c__Planctomycetia; o__Gemmatales; f__Gemmataceae; g__; s__	k__Bacteria;p__Planctomycetes;c__Planctomycetacia;o__Planctomycetales;f__Planctomycetaceae;g__uncultured;s__uncultured bacterium
OTU362	k__Bacteria; p__Proteobacteria; c__Betaproteobacteria; o__; f__; g__; s__	k__Bacteria;p__Proteobacteria;c__Betaproteobacteria;o__TRA3-20
OTU363	k__Bacteria; p__Cyanobacteria; c__Synechococcophycideae; o__Pseudanabaenales; f__Pseudanabaenaceae; g__Leptolyngbya; s__	k__Bacteria;p__Cyanobacteria;c__Cyanobacteria;o__SubsectionIII;f__FamilyI;g__Leptolyngbya;s__uncultured bacterium
OTU364	Unassigned	Unassigned
OTU365	k__Bacteria; p__Proteobacteria; c__Alphaproteobacteria; o__Sphingomonadales; f__Erythrobacteraceae; g__; s__	k__Bacteria;p__Proteobacteria;c__Alphaproteobacteria;o__Sphingomonadales;f__Erythrobacteraceae;g__Altererythrobacter;Ambiguous_taxa
OTU366	k__Bacteria; p__Proteobacteria; c__Alphaproteobacteria; o__Rhodospirillales; f__Acetobacteraceae; g__; s__	k__Bacteria;p__Proteobacteria;c__Alphaproteobacteria;o__Rhodospirillales;f__Acetobacteraceae;g__Endobacter;Ambiguous_taxa
OTU367	Unassigned	k__Bacteria;p__Planctomycetes;c__Planctomycetacia;o__Planctomycetales;f__Planctomycetaceae;g__uncultured;s__uncultured bacterium
OTU368	k__Bacteria; p__Proteobacteria; c__Alphaproteobacteria; o__Sphingomonadales; f__Sphingomonadaceae; g__; s__	k__Bacteria;p__Proteobacteria;c__Alphaproteobacteria;o__Sphingomonadales;f__uncultured;g__uncultured bacterium;s__uncultured bacterium

OTU369	k__Bacteria; p__Acidobacteria; c__Acidobacteria-6; o__iii1-15; f__; g__; s__	k__Bacteria;p__Acidobacteria;c__Subgroup 6;o__uncultured bacterium;f__uncultured bacterium;g__uncultured bacterium;s__uncultured bacterium
OTU37	k__Bacteria; p__Bacteroidetes; c__[Saprospirae]; o__[Saprospirales]; f__Chitinophagaceae; g__; s__	k__Bacteria;p__Bacteroidetes;c__Sphingobacteriia;o__Sphingobacteriales;f__Chitinophagaceae;g__uncultured;s__uncultured bacterium
OTU370	Unassigned	Unassigned
OTU371	Unassigned	Unassigned
OTU372	k__Bacteria; p__Acidobacteria; c__[Chloracidobacteria]; o__RB41; f__Ellin6075; g__; s__	k__Bacteria;p__Acidobacteria;c__Blastocatellia;o__Blastocatellales;f__Blastocatellaceae (Subgroup 4);g__uncultured;s__uncultured bacterium
OTU373	k__Bacteria; p__Proteobacteria; c__Alphaproteobacteria; o__Rhodospirillales; f__Acetobacteraceae; g__; s__	k__Bacteria;p__Proteobacteria;c__Alphaproteobacteria;o__Rhodospirillales;f__Acetobacteraceae;g__uncultured
OTU374	k__Bacteria; p__Proteobacteria; c__Betaproteobacteria; o__Burkholderiales; f__Burkholderiaceae; g__Burkholderia; s__	k__Bacteria;p__Proteobacteria;c__Betaproteobacteria;o__Burkholderiales;f__Burkholderiaceae;g__Burkholderia-Paraburkholderia
OTU375	k__Bacteria; p__Proteobacteria; c__Gammaproteobacteria; o__Oceanospirillales; f__Halomonadaceae; g__Candidatus Portiera; s__	k__Bacteria;p__Proteobacteria;c__Gammaproteobacteria;o__Oceanospirillales;f__Halomonadaceae;g__Carnimonas
OTU376	k__Bacteria; p__Acidobacteria; c__Acidobacteriia; o__Acidobacteriales; f__Acidobacteriaceae; g__; s__	k__Bacteria;p__Acidobacteria;c__Acidobacteria;o__Acidobacteriales;f__Acidobacteriaceae (Subgroup 1);g__uncultured
OTU377	k__Bacteria; p__Proteobacteria; c__Alphaproteobacteria; o__Rhodospirillales; f__Acetobacteraceae	k__Bacteria;p__Proteobacteria;c__Alphaproteobacteria;o__Rhodospirillales;f__Acetobacteraceae;g__Gluconobacter
OTU378	k__Bacteria; p__Firmicutes; c__Bacilli; o__Lactobacillales; f__Lactobacillaceae; g__Lactobacillus; s__	k__Bacteria;p__Firmicutes;c__Bacilli;o__Lactobacillales;f__Lactobacillaceae;g__Lactobacillus
OTU379	k__Bacteria; p__Actinobacteria; c__Acidimicrobiia; o__Acidimicrobiales; f__EB1017; g__; s__	k__Bacteria;p__Actinobacteria;c__Acidimicrobiia;o__Acidimicrobiales;f__uncultured;g__uncultured bacterium;s__uncultured bacterium
OTU38	k__Bacteria; p__Proteobacteria; c__Betaproteobacteria; o__Neisseriales; f__Neisseriaceae; g__Neisseria; s__cinerea	k__Bacteria;p__Proteobacteria;c__Betaproteobacteria;o__Neisseriales;f__Neisseriaceae;g__Neisseria;s__uncultured bacterium
OTU380	Unassigned	Unassigned
OTU381	k__Bacteria; p__Firmicutes; c__Bacilli; o__Bacillales; f__Paenibacillaceae; g__Paenibacillus; s__	k__Bacteria;p__Firmicutes;c__Bacilli;o__Bacillales;f__Paenibacillaceae;g__Paenibacillus
OTU382	k__Bacteria; p__Planctomycetes; c__Planctomycetia; o__Gemmatales; f__Gemmataceae; g__; s__	k__Bacteria;p__Planctomycetes;c__Planctomycetacia;o__Planctomycetales;f__Planctomycetaceae;g__uncultured
OTU383	k__Bacteria; p__Proteobacteria; c__Alphaproteobacteria; o__Sphingomonadales; f__Sphingomonadaceae; g__Kaistobacter; s__	k__Bacteria;p__Proteobacteria;c__Alphaproteobacteria;o__Sphingomonadales;f__Sphingomonadaceae;g__Sphingomonas;s__uncultured bacterium
OTU384	k__Bacteria; p__[Thermi]; c__Deinococci; o__Deinococcales; f__Deinococcaceae; g__Deinococcus; s__	k__Bacteria;p__Deinococcus-Thermus;c__Deinococci;o__Deinococcales;f__Deinococcaceae;g__Deinococcus;Ambiguous_taxa
OTU385	k__Bacteria; p__Firmicutes; c__Bacilli; o__Lactobacillales; f__; g__; s__	k__Bacteria;p__Firmicutes;c__Bacilli;o__Lactobacillales;f__Lactobacillaceae;g__Lactobacillus
OTU386	k__Bacteria; p__Proteobacteria; c__Alphaproteobacteria; o__Rhizobiales; f__Aurantimonadaceae; g__; s__	k__Bacteria;p__Proteobacteria;c__Alphaproteobacteria;o__Rhizobiales;f__Aurantimonadaceae
OTU387	Unassigned	Unassigned
OTU388	k__Bacteria; p__Actinobacteria; c__Acidimicrobiia; o__Acidimicrobiales; f__; g__; s__	k__Bacteria;p__Actinobacteria;c__Acidimicrobiia;o__Acidimicrobiales;f__uncultured

OTU389	k__Bacteria; p__Proteobacteria; c__Alphaproteobacteria; o__Rhizobiales; f__; g__; s__	k__Bacteria;p__Proteobacteria;c__Alphaproteobacteria;o__Rhizobiales
OTU39	k__Bacteria; p__Bacteroidetes; c__Bacteroidia; o__Bacteroidales; f__Bacteroidaceae; g__Bacteroides; s__	k__Bacteria;p__Bacteroidetes;c__Bacteroidia;o__Bacteroidales;f__Bacteroidaceae;g__Bacteroides;s__uncultured bacterium
OTU390	k__Bacteria; p__Proteobacteria; c__Deltaproteobacteria; o__Spirobacillales; f__; g__; s__	k__Bacteria;p__Proteobacteria;c__Deltaproteobacteria;o__Oligoflexales;f__Oligoflexaceae
OTU391	k__Bacteria; p__Firmicutes; c__Bacilli; o__Lactobacillales; f__Aerococcaceae; g__; s__	k__Bacteria;p__Firmicutes;c__Bacilli;o__Lactobacillales;f__Carnobacteriaceae;g__Marinilactibacillus;s__uncultured bacterium
OTU392	k__Bacteria; p__Firmicutes; c__Clostridia; o__Clostridiales; f__Ruminococcaceae; g__Ruminococcus; s__	k__Bacteria;p__Firmicutes;c__Clostridia;o__Clostridiales;f__Ruminococcaceae;g__Ruminiclostridium 5;s__uncultured bacterium
OTU393	k__Bacteria; p__Proteobacteria; c__Alphaproteobacteria; o__Rhodospirillales; f__Acetobacteraceae	k__Bacteria;p__Proteobacteria;c__Alphaproteobacteria;o__Rhodospirillales;f__Acetobacteraceae;g__Gluconobacter;Ambiguous_taxa
OTU394	k__Bacteria; p__Proteobacteria; c__Gammaproteobacteria; o__Pseudomonadales; f__Moraxellaceae; g__Enhydrobacter; s__	k__Bacteria;p__Proteobacteria;c__Gammaproteobacteria;o__Pseudomonadales;f__Moraxellaceae;g__Enhydrobacter;s__uncultured bacterium
OTU395	Unassigned	Unassigned
OTU396	k__Bacteria; p__Acidobacteria; c__[Chloracidobacteria]; o__RB41; f__; g__; s__	k__Bacteria;p__Acidobacteria;c__Blastocatellia;o__Blastocatellales;f__Blastocatellaceae (Subgroup 4);g__uncultured;s__uncultured bacterium
OTU397	Unassigned	Unassigned
OTU398	k__Bacteria; p__Proteobacteria; c__Gammaproteobacteria; o__Pseudomonadales; f__Moraxellaceae; g__Acinetobacter; s__rhizosphaerae	k__Bacteria;p__Proteobacteria;c__Gammaproteobacteria;o__Pseudomonadales;f__Moraxellaceae;g__Acinetobacter
OTU399	k__Bacteria; p__Bacteroidetes; c__Sphingobacteriia; o__Sphingobacteriales; f__Sphingobacteriaceae; g__; s__	k__Bacteria;p__Bacteroidetes;c__Sphingobacteriia;o__Sphingobacteriales;f__Sphingobacteriaceae;g__Mucilaginibacter;Ambiguous_taxa
OTU4	k__Bacteria; p__Bacteroidetes; c__Cytophagia; o__Cytophagales; f__Cytophagaceae; g__Dyadobacter; s__	k__Bacteria;p__Bacteroidetes;c__Cytophagia;o__Cytophagales;f__Cytophagaceae;g__Dyadobacter;s__uncultured bacterium
OTU40	k__Bacteria; p__Cyanobacteria; c__Oscillatoriophycideae; o__Chroococcales; f__Xenococcaceae; g__; s__	k__Bacteria;p__Cyanobacteria;c__Cyanobacteria;o__SubsectionII;f__FamilyII;g__Chroococcidiopsis;Ambiguous_taxa
OTU400	k__Bacteria; p__Cyanobacteria; c__Nostocophycideae; o__Nostocales; f__Nostocaceae	k__Bacteria;p__Cyanobacteria;c__Cyanobacteria;o__uncultured bacterium;f__uncultured bacterium;g__uncultured bacterium;s__uncultured bacterium
OTU401	Unassigned	Unassigned
OTU402	k__Bacteria; p__Proteobacteria; c__Alphaproteobacteria; o__Rhodobacterales; f__Rhodobacteraceae; g__; s__	k__Bacteria;p__Proteobacteria;c__Alphaproteobacteria;o__Rhodobacterales;f__Rhodobacteraceae;g__Amaricoccus
OTU403	k__Bacteria; p__Proteobacteria; c__Gammaproteobacteria; o__Pasteurellales; f__Pasteurellaceae; g__Haemophilus; s__parainfluenzae	k__Bacteria;p__Proteobacteria;c__Gammaproteobacteria;o__Pasteurellales;f__Pasteurellaceae;g__Haemophilus;s__uncultured bacterium
OTU404	Unassigned	Unassigned
OTU405	k__Bacteria; p__Proteobacteria; c__Gammaproteobacteria; o__Aeromonadales; f__Aeromonadaceae; g__; s__	k__Bacteria;p__Proteobacteria;c__Gammaproteobacteria;o__Aeromonadales;f__Aeromonadaceae;g__Aeromonas
OTU406	Unassigned	Unassigned
OTU407	k__Bacteria; p__Actinobacteria; c__Actinobacteria; o__Actinomycetales; f__Cellulomonadaceae	k__Bacteria;p__Actinobacteria;c__Actinobacteria;o__Micrococcales;f__Cellulomonadaceae
OTU408	Unassigned	Unassigned

OTU409	k__Bacteria; p__Proteobacteria; c__Alphaproteobacteria; o__Rhodospirillales; f__Acetobacteraceae; g__; s__	k__Bacteria;p__Proteobacteria;c__Alphaproteobacteria;o__Rhodospirillales;f__Acetobacteraceae;g__Endobacter;Ambiguous_taxa
OTU41	k__Bacteria; p__Proteobacteria; c__Alphaproteobacteria; o__Rhizobiales; f__Methylocystaceae; g__; s__	k__Bacteria;p__Proteobacteria;c__Alphaproteobacteria;o__Rhizobiales;f__1174-901-12;g__uncultured bacterium;s__uncultured bacterium
OTU410	k__Bacteria; p__Actinobacteria; c__Actinobacteria; o__Actinomycetales; f__Microbacteriaceae; g__; s__	k__Bacteria;p__Actinobacteria;c__Actinobacteria;o__Micrococcales;f__Microbacteriaceae
OTU411	k__Bacteria; p__Firmicutes; c__Bacilli; o__Lactobacillales; f__Lactobacillaceae; g__Lactobacillus; s__	k__Bacteria;p__Firmicutes;c__Bacilli;o__Lactobacillales;f__Lactobacillaceae;g__Lactobacillus;s__uncultured Lactobacillus sp.
OTU412	Unassigned	Unassigned
OTU413	Unassigned	Unassigned
OTU414	k__Bacteria; p__Cyanobacteria; c__Chloroplast; o__Streptophyta; f__; g__; s__	k__Bacteria;p__Cyanobacteria;c__Chloroplast;Ambiguous_taxa;Ambiguous_taxa;Ambiguous_taxa;Ambiguous_taxa
OTU415	k__Bacteria; p__Proteobacteria; c__Alphaproteobacteria; o__Sphingomonadales; f__Sphingomonadaceae; g__Sphingomonas; s__	k__Bacteria;p__Proteobacteria;c__Alphaproteobacteria;o__Sphingomonadales;f__Sphingomonadaceae;g__Sphingomonas;Ambiguous_taxa
OTU416	k__Bacteria; p__Proteobacteria; c__Alphaproteobacteria; o__Sphingomonadales; f__Sphingomonadaceae; g__Sphingomonas; s__	k__Bacteria;p__Proteobacteria;c__Alphaproteobacteria;o__Sphingomonadales;f__Sphingomonadaceae;g__Sphingomonas;s__uncultured bacterium
OTU417	k__Bacteria; p__Bacteroidetes; c__Flavobacteriia; o__Flavobacteriales; f__[Weeksellaceae]; g__Chryseobacterium; s__	k__Bacteria;p__Bacteroidetes;c__Flavobacteriia;o__Flavobacteriales;f__Flavobacteriaceae;g__Chryseobacterium;Ambiguous_taxa
OTU418	k__Bacteria; p__Firmicutes; c__Bacilli; o__Lactobacillales; f__Lactobacillaceae; g__Lactobacillus; s__	k__Bacteria;p__Firmicutes;c__Bacilli;o__Lactobacillales;f__Lactobacillaceae;g__Lactobacillus;Ambiguous_taxa
OTU419	k__Bacteria; p__Proteobacteria; c__Betaproteobacteria; o__Burkholderiales; f__Burkholderiaceae; g__Burkholderia; s__	k__Bacteria;p__Proteobacteria;c__Betaproteobacteria;o__Burkholderiales;f__Burkholderiaceae;g__Burkholderia-Paraburkholderia;Ambiguous_taxa
OTU42	k__Bacteria; p__Actinobacteria; c__Thermoleophilia; o__Gaiellales; f__Gaiellaceae; g__; s__	k__Bacteria;p__Actinobacteria;c__Thermoleophilia;o__Gaiellales;f__Gaiellaceae;g__Gaiella
OTU420	k__Bacteria; p__Proteobacteria; c__Betaproteobacteria; o__Burkholderiales; f__Comamonadaceae	k__Bacteria;p__Proteobacteria;c__Betaproteobacteria;o__Burkholderiales;f__Comamonadaceae
OTU421	k__Bacteria; p__Actinobacteria; c__Actinobacteria; o__Actinomycetales; f__Corynebacteriaceae; g__Corynebacterium; s__durum	k__Bacteria;p__Actinobacteria;c__Actinobacteria;o__Corynebacteriales;f__Corynebacteriaceae;g__Corynebacterium;s__uncultured bacterium
OTU422	k__Bacteria; p__Actinobacteria; c__Acidimicrobiia; o__Acidimicrobiales; f__; g__; s__	k__Bacteria;p__Actinobacteria;c__Acidimicrobiia;o__Acidimicrobiales;f__uncultured;Ambiguous_taxa;Ambiguous_taxa
OTU423	k__Bacteria; p__Proteobacteria; c__Gammaproteobacteria; o__Enterobacteriales; f__Enterobacteriaceae; g__Erwinia	k__Bacteria;p__Proteobacteria;c__Gammaproteobacteria;o__Enterobacteriales;f__Enterobacteriaceae
OTU424	k__Bacteria; p__Proteobacteria; c__Alphaproteobacteria; o__Rhodospirillales; f__Acetobacteraceae	k__Bacteria;p__Proteobacteria;c__Alphaproteobacteria;o__Rhodospirillales;f__Acetobacteraceae
OTU425	k__Bacteria; p__Proteobacteria; c__Gammaproteobacteria; o__Enterobacteriales; f__Enterobacteriaceae; g__; s__	k__Bacteria;p__Proteobacteria;c__Gammaproteobacteria;o__Enterobacteriales;f__Enterobacteriaceae;g__Enterobacter
OTU426	k__Bacteria; p__Actinobacteria; c__Acidimicrobiia; o__Acidimicrobiales; f__; g__; s__	k__Bacteria;p__Actinobacteria;c__Acidimicrobiia;o__Acidimicrobiales;f__uncultured;Ambiguous_taxa;Ambiguous_taxa
OTU427	k__Bacteria; p__Firmicutes; c__Erysipelotrichi; o__Erysipelotrichales; f__Erysipelotrichaceae; g__[Eubacterium]; s__biforme	k__Bacteria;p__Firmicutes;c__Erysipelotrichia;o__Erysipelotrichales;f__Erysipelotrichaceae;g__Holdemanella;s__uncultured bacterium
OTU428	k__Bacteria; p__Actinobacteria; c__Actinobacteria; o__Actinomycetales; f__Nocardioidaceae; g__; s__	k__Bacteria;p__Actinobacteria;c__Actinobacteria;o__Propionibacteriales;f__Nocardioidaceae;g__Nocardioides;s__uncultured bacterium

OTU429	k__Bacteria; p__Proteobacteria; c__Alphaproteobacteria; o__Caulobacterales; f__Caulobacteraceae; g__; s__	k__Bacteria;p__Proteobacteria;c__Alphaproteobacteria;o__Caulobacterales;f__Caulobacteraceae;g__Caulobacter
OTU43	k__Bacteria; p__Proteobacteria; c__Alphaproteobacteria; o__Rhodospirillales; f__Acetobacteraceae; g__; s__	k__Bacteria;p__Proteobacteria;c__Alphaproteobacteria;o__Rhodospirillales;f__Acetobacteraceae;g__Acidiphilium;s__uncultured bacterium
OTU430	k__Bacteria; p__Bacteroidetes; c__Cytophagia; o__Cytophagales; f__Cytophagaceae; g__Spirosoma; s__	k__Bacteria;p__Bacteroidetes;c__Cytophagia;o__Cytophagales;f__Cytophagaceae;g__Spirosoma;s__uncultured bacterium
OTU431	k__Bacteria; p__Proteobacteria; c__Alphaproteobacteria; o__Rhizobiales; f__Methylocystaceae; g__; s__	k__Bacteria;p__Proteobacteria;c__Alphaproteobacteria;o__Rhizobiales;f__1174-901-12;g__uncultured bacterium;s__uncultured bacterium
OTU432	Unassigned	Unassigned
OTU433	k__Bacteria; p__Acidobacteria; c__Sva0725; o__Sva0725; f__; g__; s__	k__Bacteria;p__Acidobacteria;c__Holophagae;o__Subgroup 10;f__ABS-19;g__uncultured bacterium;s__uncultured bacterium
OTU434	k__Bacteria; p__Firmicutes; c__Bacilli; o__Bacillales; f__Bacillaceae; g__Bacillus; s__cereus	k__Bacteria;p__Firmicutes;c__Bacilli;o__Bacillales;f__Bacillaceae;g__Bacillus;s__Bacillus thuringiensis
OTU435	k__Bacteria; p__Armatimonadetes; c__Armatimonadia; o__Armatimonadales; f__Armatimonadaceae; g__; s__	k__Bacteria;p__Armatimonadetes;c__Armatimonadia;o__Armatimonadales;f__uncultured bacterium;g__uncultured bacterium;s__uncultured bacterium
OTU436	k__Bacteria; p__Proteobacteria; c__Alphaproteobacteria; o__Rhizobiales; f__Methylocystaceae; g__; s__	k__Bacteria;p__Proteobacteria;c__Alphaproteobacteria;o__Rhizobiales;f__1174-901-12
OTU437	Unassigned	Unassigned
OTU438	k__Bacteria; p__Proteobacteria; c__Alphaproteobacteria; o__Sphingomonadales; f__Sphingomonadaceae; g__Sphingomonas; s__	k__Bacteria;p__Proteobacteria;c__Alphaproteobacteria;o__Sphingomonadales;f__Sphingomonadaceae;g__Sphingomonas
OTU439	k__Bacteria; p__Cyanobacteria; c__Oscillatoriophycideae; o__Oscillatoriales; f__Phormidiaceae; g__Microcoleus; s__	k__Bacteria;p__Cyanobacteria;c__Cyanobacteria;o__SubsectionIII;f__FamilyI;g__Coleofasciculus;s__uncultured organism
OTU44	k__Bacteria; p__Proteobacteria; c__Alphaproteobacteria; o__Rhizobiales; f__Methylocystaceae; g__; s__	k__Bacteria;p__Proteobacteria;c__Alphaproteobacteria;o__Rhizobiales;f__1174-901-12;g__uncultured bacterium;s__uncultured bacterium
OTU440	k__Bacteria; p__Cyanobacteria; c__Chloroplast; o__Streptophyta; f__; g__; s__	k__Bacteria;p__Cyanobacteria;c__Chloroplast;o__Cercis gigantea;f__Cercis gigantea;g__Cercis gigantea;s__Cercis gigantea
OTU441	k__Bacteria; p__Bacteroidetes; c__Flavobacteriia; o__Flavobacteriales; f__[Weeksellaceae]; g__Chryseobacterium; s__	k__Bacteria;p__Bacteroidetes;c__Flavobacteriia;o__Flavobacteriales;f__Flavobacteriaceae;g__Chryseobacterium;Ambiguous_taxa
OTU442	k__Bacteria; p__Bacteroidetes; c__Cytophagia; o__Cytophagales; f__Cytophagaceae; g__Spirosoma; s__	k__Bacteria;p__Bacteroidetes;c__Cytophagia;o__Cytophagales;f__Cytophagaceae;g__Spirosoma;s__uncultured bacterium
OTU443	k__Bacteria; p__Proteobacteria; c__Gammaproteobacteria; o__Xanthomonadales; f__Xanthomonadaceae; g__Pseudoxanthomonas	k__Bacteria;p__Proteobacteria;c__Gammaproteobacteria;o__Xanthomonadales;f__Xanthomonadaceae;g__Pseudoxanthomonas;Ambiguous_taxa
OTU444	Unassigned	Unassigned
OTU445	Unassigned	Unassigned
OTU446	k__Bacteria; p__Verrucomicrobia; c__[Pedosphaerae]; o__[Pedosphaerales]; f__Ellin517; g__; s__	k__Bacteria;p__Verrucomicrobia;c__OPB35 soil group;o__uncultured bacterium;f__uncultured bacterium;g__uncultured bacterium;s__uncultured bacterium
OTU447	Unassigned	Unassigned
OTU448	k__Bacteria; p__Proteobacteria; c__Alphaproteobacteria; o__Rhizobiales; f__Beijerinckiaceae; g__Beijerinckia; s__	k__Bacteria;p__Proteobacteria;c__Alphaproteobacteria;o__Rhizobiales;f__1174-901-12
OTU449	k__Bacteria; p__Planctomycetes; c__Planctomycetia; o__Gemmatales; f__Gemmataceae; g__Gemmata; s__	k__Bacteria;p__Planctomycetes;c__Planctomycetacia;o__Planctomycetales;f__Planctomycetaceae;g__Gemmata;s__uncultured bacterium

OTU45	k__Bacteria; p__Acidobacteria; c__[Chloracidobacteria]; o__PK29; f__; g__; s__	k__Bacteria;p__Acidobacteria;c__Blastocatellia;o__Blastocatellales;f__Blastocatellaceae (Subgroup 4);g__11-24;s__uncultured bacterium
OTU450	k__Bacteria; p__Proteobacteria; c__Alphaproteobacteria; o__Rhizobiales; f__Methylocystaceae; g__; s__	k__Bacteria;p__Proteobacteria;c__Alphaproteobacteria;o__Rhizobiales;f__1174-901-12;g__uncultured bacterium;s__uncultured bacterium
OTU451	k__Bacteria; p__Actinobacteria; c__Actinobacteria; o__Actinomycetales; f__; g__; s__	k__Bacteria;p__Actinobacteria;c__Actinobacteria;o__Frankiales;f__Sporichthyaceae
OTU452	k__Bacteria; p__Actinobacteria; c__Acidimicrobiia; o__Acidimicrobiales; f__C111; g__; s__	k__Bacteria;p__Actinobacteria;c__Acidimicrobiia;o__Acidimicrobiales;f__Acidimicrobiaceae;g__CL500-29 marine group
OTU453	k__Bacteria; p__Proteobacteria; c__Gammaproteobacteria; o__Enterobacteriales; f__Enterobacteriaceae; g__Erwinia	k__Bacteria;p__Proteobacteria;c__Gammaproteobacteria;o__Enterobacteriales;f__Enterobacteriaceae;g__Pantoea;Ambiguous_taxa
OTU454	k__Bacteria; p__Proteobacteria; c__Alphaproteobacteria; o__Sphingomonadales; f__Sphingomonadaceae; g__Sphingomonas; s__wittichii	k__Bacteria;p__Proteobacteria;c__Alphaproteobacteria;o__Sphingomonadales;f__Sphingomonadaceae;g__Sphingomonas;Ambiguous_taxa
OTU455	k__Bacteria; p__Acidobacteria; c__Acidobacteria-6; o__iii1-15; f__; g__; s__	k__Bacteria;p__Acidobacteria;c__Subgroup 6
OTU456	k__Bacteria; p__Actinobacteria; c__Thermoleophilia; o__Gaiellales; f__Gaiellaceae; g__; s__	k__Bacteria;p__Actinobacteria;c__Thermoleophilia;o__Gaiellales;f__uncultured;g__uncultured bacterium;s__uncultured bacterium
OTU457	k__Bacteria; p__Proteobacteria; c__Gammaproteobacteria; o__Enterobacteriales; f__Enterobacteriaceae; g__; s__	k__Bacteria;p__Proteobacteria;c__Gammaproteobacteria;o__Enterobacteriales;f__Enterobacteriaceae;g__Klebsiella;s__uncultured bacterium
OTU458	k__Bacteria; p__Firmicutes; c__Clostridia; o__Clostridiales; f__Ruminococcaceae; g__Oscillospira; s__	k__Bacteria;p__Firmicutes;c__Clostridia;o__Clostridiales;f__Ruminococcaceae;g__uncultured
OTU459	k__Bacteria; p__Armatimonadetes; c__[Fimbriimonadia]; o__[Fimbriimonadales]; f__[Fimbriimonadaceae]; g__Fimbriimonas; s__	k__Bacteria;p__Armatimonadetes;c__Fimbriimonadia;o__Fimbriimonadales;f__Fimbriimonadaceae;g__uncultured bacterium;s__uncultured bacterium
OTU46	k__Bacteria; p__Proteobacteria; c__Alphaproteobacteria; o__Rhizobiales; f__Bradyrhizobiaceae; g__; s__	k__Bacteria;p__Proteobacteria;c__Alphaproteobacteria;o__Rhizobiales;f__Bradyrhizobiaceae;g__Bradyrhizobium
OTU460	k__Bacteria; p__[Thermi]; c__Deinococci; o__Deinococcales; f__Deinococcaceae; g__Deinococcus; s__	k__Bacteria;p__Deinococcus-Thermus;c__Deinococci;o__Deinococcales;f__Deinococcaceae;g__Deinococcus
OTU461	k__Bacteria; p__Bacteroidetes; c__Bacteroidia; o__Bacteroidales; f__Bacteroidaceae; g__Bacteroides; s__	k__Bacteria;p__Bacteroidetes;c__Bacteroidia;o__Bacteroidales;f__Bacteroidaceae;g__Bacteroides
OTU462	k__Bacteria; p__Bacteroidetes; c__[Saprospirae]; o__[Saprospirales]; f__Chitinophagaceae; g__; s__	k__Bacteria;p__Bacteroidetes;c__Sphingobacteriia;o__Sphingobacteriales;f__Chitinophagaceae;g__uncultured;s__uncultured bacterium
OTU463	Unassigned	k__Bacteria;p__Planctomycetes;c__Planctomycetacia;o__Planctomycetales;f__Planctomycetaceae;g__uncultured;Ambiguous_taxa
OTU464	k__Bacteria; p__Proteobacteria; c__Gammaproteobacteria; o__Enterobacteriales; f__Enterobacteriaceae; g__Erwinia; s__	k__Bacteria;p__Proteobacteria;c__Gammaproteobacteria;o__Enterobacteriales;f__Enterobacteriaceae;g__Pantoea
OTU465	Unassigned	Unassigned
OTU466	k__Bacteria; p__TM7; c__TM7-1; o__; f__; g__; s__	k__Bacteria;p__Saccharibacteria;c__uncultured bacterium;o__uncultured bacterium;f__uncultured bacterium;g__uncultured bacterium;s__uncultured bacterium
OTU467	k__Bacteria; p__Proteobacteria; c__Alphaproteobacteria; o__Rhizobiales; f__Methylocystaceae; g__; s__	k__Bacteria;p__Proteobacteria;c__Alphaproteobacteria;o__Rhizobiales;f__1174-901-12;g__uncultured bacterium;s__uncultured bacterium
OTU468	Unassigned	Unassigned

OTU469	k__Bacteria; p__Proteobacteria; c__Alphaproteobacteria; o__Rhizobiales; f__Rhizobiaceae; g__Agrobacterium; s__	k__Bacteria;p__Proteobacteria;c__Alphaproteobacteria;o__Rhizobiales
OTU47	k__Bacteria; p__Acidobacteria; c__Solibacteres; o__Solibacterales; f__; g__; s__	k__Bacteria;p__Acidobacteria;c__Solibacteres;o__Solibacterales;f__Solibacteraceae (Subgroup 3);g__Bryobacter;s__uncultured Acidobacteria bacterium
OTU470	k__Bacteria; p__Proteobacteria; c__Alphaproteobacteria; o__Caulobacterales; f__Caulobacteraceae; g__; s__	k__Bacteria;p__Proteobacteria;c__Alphaproteobacteria;o__Caulobacterales;f__Caulobacteraceae;g__uncultured;s__uncultured bacterium
OTU471	k__Bacteria; p__Acidobacteria; c__[Chloracidobacteria]; o__RB41; f__Ellin6075; g__; s__	k__Bacteria;p__Acidobacteria;c__Blastocatellia;o__Blastocatellales;f__Blastocatellaceae (Subgroup 4);g__Stenotrophobacter;s__uncultured bacterium
OTU472	k__Bacteria; p__Proteobacteria; c__Gammaproteobacteria; o__Pseudomonadales; f__Moraxellaceae; g__Acinetobacter; s__	k__Bacteria;p__Proteobacteria;c__Gammaproteobacteria;o__Pseudomonadales;f__Moraxellaceae;g__Acinetobacter
OTU473	k__Bacteria; p__Actinobacteria; c__Actinobacteria; o__Actinomycetales; f__Kineosporiaceae; g__; s__	k__Bacteria;p__Actinobacteria;c__Actinobacteria;o__Kineosporiales;f__Kineosporiaceae;g__Quadrisphaera;Ambiguous_taxa
OTU474	k__Bacteria; p__Firmicutes; c__Bacilli; o__Lactobacillales; f__Lactobacillaceae; g__Lactobacillus; s__	k__Bacteria;p__Firmicutes;c__Bacilli;o__Lactobacillales;f__Lactobacillaceae;g__Lactobacillus;Ambiguous_taxa
OTU475	k__Bacteria; p__Proteobacteria; c__Alphaproteobacteria; o__Rickettsiales; f__mitochondria	k__Bacteria;p__Proteobacteria;c__Alphaproteobacteria;o__Rickettsiales;f__Mitochondria;g__Arachis ipaensis;s__Arachis ipaensis
OTU476	k__Bacteria; p__TM7; c__TM7-3; o__; f__; g__; s__	k__Bacteria;p__Saccharibacteria;c__uncultured bacterium;o__uncultured bacterium;f__uncultured bacterium;g__uncultured bacterium;s__uncultured bacterium
OTU477	k__Bacteria; p__Planctomycetes; c__Planctomycetia; o__Gemmatales; f__Gemmataceae; g__Gemmata; s__	k__Bacteria;p__Planctomycetes;c__Planctomycetacia;o__Planctomycetales;f__Planctomycetaceae;g__Gemmata;Ambiguous_taxa
OTU478	k__Bacteria; p__Proteobacteria; c__Betaproteobacteria; o__MND1; f__; g__; s__	k__Bacteria;p__Proteobacteria;c__Betaproteobacteria;o__Nitrosomonadales;f__Nitrosomonadaceae;g__uncultured;s__uncultured bacterium
OTU479	k__Bacteria; p__Proteobacteria; c__Alphaproteobacteria; o__Rickettsiales; f__; g__; s__	k__Bacteria;p__Proteobacteria;c__Alphaproteobacteria;o__Rickettsiales;f__uncultured;g__uncultured bacterium;s__uncultured bacterium
OTU48	k__Bacteria; p__Actinobacteria; c__Actinobacteria; o__Actinomycetales; f__Pseudonocardiaceae; g__Pseudonocardia; s__	k__Bacteria;p__Actinobacteria;c__Actinobacteria;o__Pseudonocardiales;f__Pseudonocardiaceae;g__Pseudonocardia
OTU480	k__Bacteria; p__Proteobacteria; c__Alphaproteobacteria; o__Rhodospirillales; f__Acetobacteraceae	k__Bacteria;p__Proteobacteria;c__Alphaproteobacteria;o__Rhodospirillales;f__Acetobacteraceae;g__Gluconobacter
OTU481	Unassigned	Unassigned
OTU482	k__Bacteria; p__Proteobacteria; c__Alphaproteobacteria; o__Sphingomonadales; f__Sphingomonadaceae; g__; s__	k__Bacteria;p__Proteobacteria;c__Alphaproteobacteria;o__Sphingomonadales;f__Sphingomonadaceae
OTU483	k__Bacteria; p__Acidobacteria; c__[Chloracidobacteria]; o__RB41; f__Ellin6075; g__; s__	k__Bacteria;p__Acidobacteria;c__Blastocatellia;o__Blastocatellales;f__Blastocatellaceae (Subgroup 4);g__Blastocatella
OTU484	k__Bacteria; p__Proteobacteria; c__Betaproteobacteria; o__Burkholderiales; f__Comamonadaceae; g__; s__	k__Bacteria;p__Proteobacteria;c__Betaproteobacteria;o__Burkholderiales;f__Comamonadaceae;g__uncultured
OTU485	k__Bacteria; p__Firmicutes; c__Bacilli; o__Bacillales; f__Bacillaceae; g__Bacillus; s__	k__Bacteria;p__Firmicutes;c__Bacilli;o__Bacillales;f__Bacillaceae;g__Bacillus;Ambiguous_taxa
OTU486	k__Bacteria; p__Proteobacteria; c__Alphaproteobacteria; o__Rhodospirillales; f__Acetobacteraceae; g__; s__	k__Bacteria;p__Proteobacteria;c__Alphaproteobacteria;o__Rhodospirillales;f__Acetobacteraceae;g__uncultured;s__uncultured bacterium
OTU487	Unassigned	Unassigned

OTU488	k__Bacteria; p__Planctomycetes; c__Phycisphaerae; o__WD2101; f__; g__; s__	k__Bacteria;p__Planctomycetes;c__Phycisphaerae;o__Tepidisphaerales;f__Tepidisphaeraceae;g__uncultured bacterium;s__uncultured bacterium
OTU489	k__Bacteria; p__Proteobacteria; c__Alphaproteobacteria; o__Caulobacterales; f__Caulobacteraceae; g__Mycoplana; s__	k__Bacteria;p__Proteobacteria;c__Alphaproteobacteria;o__Caulobacterales;f__Caulobacteraceae;g__Brevundimonas
OTU49	k__Bacteria; p__Verrucomicrobia; c__[Spartobacteria]; o__[Chthoniobacterales]; f__[Chthoniobacteraceae]; g__OR-59; s__	k__Bacteria;p__Verrucomicrobia;c__Spartobacteria;o__Chthoniobacterales;f__Chthoniobacteraceae;g__Chthoniobacter
OTU490	k__Bacteria; p__Bacteroidetes; c__[Saprospirae]; o__[Saprospirales]; f__Chitinophagaceae; g__; s__	k__Bacteria;p__Bacteroidetes;c__Sphingobacteriia;o__Sphingobacteriales;f__Chitinophagaceae;g__uncultured;s__uncultured bacterium
OTU491	k__Bacteria; p__Actinobacteria; c__Thermoleophilia; o__Solirubrobacterales; f__; g__; s__	k__Bacteria;p__Actinobacteria;c__Thermoleophilia;o__Solirubrobacterales;f__0319-6M6;g__uncultured bacterium;s__uncultured bacterium
OTU492	Unassigned	Unassigned
OTU493	k__Bacteria; p__Actinobacteria; c__Actinobacteria; o__Actinomycetales; f__Brevibacteriaceae; g__Brevibacterium; s__	k__Bacteria;p__Actinobacteria;c__Actinobacteria;o__Micrococcales;f__Brevibacteriaceae;g__Brevibacterium;Ambiguous_taxa
OTU494	k__Bacteria; p__Proteobacteria; c__Alphaproteobacteria; o__Rhizobiales; f__; g__; s__	k__Bacteria;p__Proteobacteria;c__Alphaproteobacteria;o__Rhizobiales;f__Xanthobacteraceae;g__Labrys;s__uncultured bacterium
OTU495	k__Bacteria; p__Firmicutes; c__Bacilli; o__Lactobacillales; f__Lactobacillaceae; g__Lactobacillus; s__	k__Bacteria;p__Firmicutes;c__Bacilli;o__Lactobacillales;f__Lactobacillaceae;g__Lactobacillus;s__Lactobacillus acidophilus
OTU496	Unassigned	Unassigned
OTU497	k__Bacteria; p__Proteobacteria; c__Gammaproteobacteria; o__Legionellales; f__Legionellaceae	k__Bacteria;p__Proteobacteria;c__Gammaproteobacteria;o__Legionellales;f__Legionellaceae;g__Legionella
OTU498	k__Bacteria; p__Proteobacteria; c__Alphaproteobacteria; o__Rhizobiales; f__; g__; s__	k__Bacteria;p__Proteobacteria;c__Alphaproteobacteria;o__Rhizobiales;f__Rhizobiaceae;g__Ensifer;Ambiguous_taxa
OTU499	k__Bacteria; p__Bacteroidetes; c__Cytophagia; o__Cytophagales; f__Cytophagaceae; g__Hymenobacter; s__	k__Bacteria;p__Bacteroidetes;c__Cytophagia;o__Cytophagales;f__Cytophagaceae;g__Hymenobacter;Ambiguous_taxa
OTU5	k__Bacteria; p__Proteobacteria; c__Betaproteobacteria; o__Burkholderiales; f__Comamonadaceae; g__Leptothrix; s__	k__Bacteria;p__Proteobacteria;c__Betaproteobacteria;o__Burkholderiales;f__Comamonadaceae
OTU50	k__Bacteria; p__Proteobacteria; c__Alphaproteobacteria; o__Sphingomonadales; f__Sphingomonadaceae; g__Sphingomonas; s__changbaiensis	k__Bacteria;p__Proteobacteria;c__Alphaproteobacteria;o__Sphingomonadales;f__Sphingomonadaceae;g__Sphingomonas
OTU500	Unassigned	Unassigned
OTU501	k__Bacteria; p__Proteobacteria; c__Alphaproteobacteria; o__Sphingomonadales; f__Sphingomonadaceae; g__Sphingomonas; s__	k__Bacteria;p__Proteobacteria;c__Alphaproteobacteria;o__Sphingomonadales;f__Sphingomonadaceae;g__Sphingomonas;s__uncultured bacterium
OTU502	k__Bacteria; p__Proteobacteria; c__Alphaproteobacteria; o__Rhodospirillales; f__Acetobacteraceae; g__Acidocella; s__	k__Bacteria;p__Proteobacteria;c__Alphaproteobacteria;o__Rhodospirillales;f__Acetobacteraceae
OTU503	k__Bacteria; p__Proteobacteria; c__Alphaproteobacteria; o__Caulobacterales; f__Caulobacteraceae; g__; s__	k__Bacteria;p__Proteobacteria;c__Alphaproteobacteria;o__Caulobacterales;f__Caulobacteraceae;g__uncultured
OTU504	k__Bacteria; p__Actinobacteria; c__Actinobacteria; o__Actinomycetales; f__Microbacteriaceae; g__Microbacterium	k__Bacteria;p__Actinobacteria;c__Actinobacteria;o__Micrococcales;f__Microbacteriaceae;g__Microbacterium;Ambiguous_taxa
OTU505	k__Bacteria; p__Proteobacteria; c__Gammaproteobacteria; o__Enterobacteriales; f__Enterobacteriaceae; g__; s__	k__Bacteria;p__Proteobacteria;c__Gammaproteobacteria;o__Enterobacteriales;f__Enterobacteriaceae;g__Enterobacter
OTU506	k__Bacteria; p__Bacteroidetes; c__Cytophagia; o__Cytophagales; f__Cytophagaceae; g__; s__	k__Bacteria;p__Bacteroidetes;c__Cytophagia;o__Cytophagales;f__Cytophagaceae;g__Flexibacter

OTU507	Unassigned	Unassigned
OTU508	k__Bacteria; p__Proteobacteria; c__Alphaproteobacteria; o__Rhodospirillales; f__Acetobacteraceae; g__Gluconobacter; s__	k__Bacteria;p__Proteobacteria;c__Alphaproteobacteria;o__Rhodospirillales;f__Acetobacteraceae;g__Gluconobacter
OTU509	k__Bacteria; p__Proteobacteria; c__Gammaproteobacteria; o__Enterobacteriales; f__Enterobacteriaceae; g__; s__	k__Bacteria;p__Proteobacteria;c__Gammaproteobacteria;o__Enterobacteriales;f__Enterobacteriaceae;g__uncultured
OTU51	k__Bacteria; p__Planctomycetes; c__Phycisphaerae; o__Phycisphaerales; f__; g__; s__	k__Bacteria;p__Planctomycetes;c__Phycisphaerae;o__Phycisphaerales;f__Phycisphaeraceae;g__CL500-3;s__uncultured bacterium
OTU510	k__Bacteria; p__Actinobacteria; c__Actinobacteria; o__Actinomycetales; f__Nocardioidaceae; g__Nocardioides	k__Bacteria;p__Actinobacteria;c__Actinobacteria;o__Propionibacteriales;f__Nocardioidaceae;g__Nocardioides;Ambiguous_taxa
OTU511	k__Bacteria; p__Proteobacteria; c__Alphaproteobacteria; o__Rhodobacterales; f__Rhodobacteraceae; g__; s__	k__Bacteria;p__Proteobacteria;c__Alphaproteobacteria;o__Rhodobacterales;f__Rhodobacteraceae
OTU512	k__Bacteria; p__Proteobacteria; c__Alphaproteobacteria; o__Caulobacterales; f__Caulobacteraceae; g__Mycoplana; s__	k__Bacteria;p__Proteobacteria;c__Alphaproteobacteria;o__Caulobacterales;f__Caulobacteraceae;g__Brevundimonas
OTU513	k__Bacteria; p__Proteobacteria; c__Gammaproteobacteria; o__Xanthomonadales; f__Sinobacteraceae; g__; s__	k__Bacteria;p__Proteobacteria;c__Gammaproteobacteria;o__Xanthomonadales;f__Xanthomonadales Incertae Sedis;g__Acidibacter;s__uncultured bacterium
OTU514	k__Bacteria; p__Actinobacteria; c__Actinobacteria; o__Actinomycetales; f__Nocardioidaceae; g__; s__	k__Bacteria;p__Actinobacteria;c__Actinobacteria;o__Propionibacteriales;f__Nocardioidaceae;g__Nocardioides;s__uncultured bacterium
OTU515	Unassigned	Unassigned
OTU516	k__Bacteria; p__Nitrospirae; c__Nitrospira; o__Nitrospirales; f__Nitrospiraceae; g__Nitrospira; s__	k__Bacteria;p__Nitrospirae;c__Nitrospira;o__Nitrospirales;f__Nitrospiraceae;g__Nitrospira;s__uncultured bacterium
OTU517	k__Bacteria; p__Bacteroidetes; c__[Saprospirae]; o__[Saprospirales]; f__Chitinophagaceae; g__Flavisolibacter; s__	k__Bacteria;p__Bacteroidetes;c__Sphingobacteriia;o__Sphingobacteriales;f__Chitinophagaceae;g__Flavisolibacter;s__uncultured bacterium
OTU518	k__Bacteria; p__Firmicutes; c__Bacilli; o__Bacillales; f__Bacillaceae; g__Bacillus; s__	k__Bacteria;p__Firmicutes;c__Bacilli;o__Bacillales;f__Bacillaceae;g__Bacillus;Ambiguous_taxa
OTU519	k__Bacteria; p__Actinobacteria; c__Actinobacteria; o__Actinomycetales; f__Corynebacteriaceae; g__Corynebacterium; s__	k__Bacteria;p__Actinobacteria;c__Actinobacteria;o__Corynebacteriales;f__Corynebacteriaceae;g__Corynebacterium 1
OTU52	k__Bacteria; p__Proteobacteria; c__Alphaproteobacteria; o__Rhodospirillales; f__Acetobacteraceae; g__; s__	k__Bacteria;p__Proteobacteria;c__Alphaproteobacteria;o__Rhodospirillales;f__Acetobacteraceae;g__uncultured;s__uncultured bacterium
OTU520	k__Bacteria; p__Firmicutes; c__Clostridia; o__Clostridiales; f__Clostridiaceae; g__Thermoanaerobacterium; s__	k__Bacteria;p__Firmicutes;c__Clostridia;o__Thermoanaerobacterales;f__Family III;g__Thermoanaerobacterium;Ambiguous_taxa
OTU521	k__Bacteria; p__Cyanobacteria; c__Chloroplast; o__Streptophyta; f__; g__; s__	k__Bacteria;p__Cyanobacteria;c__Chloroplast;o__uncultured bacterium;f__uncultured bacterium;g__uncultured bacterium;s__uncultured bacterium
OTU522	k__Bacteria; p__Proteobacteria; c__Alphaproteobacteria; o__Rhodospirillales; f__Acetobacteraceae	k__Bacteria;p__Proteobacteria;c__Alphaproteobacteria;o__Rhodospirillales;f__Acetobacteraceae;g__uncultured;s__uncultured Acetobacteraceae bacterium
OTU523	k__Bacteria; p__Actinobacteria; c__Actinobacteria; o__Actinomycetales; f__Nocardiaceae; g__Nocardia; s__	k__Bacteria;p__Actinobacteria;c__Actinobacteria;o__Corynebacteriales;f__nbr16a11;g__uncultured bacterium;s__uncultured bacterium
OTU524	k__Bacteria; p__Verrucomicrobia; c__[Spartobacteria]; o__[Chthoniobacterales]; f__[Chthoniobacteraceae]; g__DA101; s__	k__Bacteria;p__Verrucomicrobia;c__Spartobacteria;o__Chthoniobacterales;f__DA101 soil group;g__uncultured bacterium;s__uncultured bacterium
OTU525	Unassigned	Unassigned

OTU526	k__Bacteria; p__Proteobacteria; c__Alphaproteobacteria; o__; f__; g__; s__	k__Bacteria;p__Proteobacteria;c__Alphaproteobacteria;o__Rickettsiales;f__SM2D12;g__uncultured bacterium;s__uncultured bacterium
OTU527	k__Bacteria; p__Proteobacteria; c__Gammaproteobacteria; o__Aeromonadales; f__Aeromonadaceae; g__; s__	k__Bacteria;p__Proteobacteria;c__Gammaproteobacteria;o__Aeromonadales;f__Aeromonadaceae;g__Aeromonas;Ambiguous_taxa
OTU528	k__Bacteria; p__Proteobacteria; c__Alphaproteobacteria; o__Sphingomonadales; f__Sphingomonadaceae; g__; s__	k__Bacteria;p__Proteobacteria;c__Alphaproteobacteria;o__Sphingomonadales;f__Ellin6055;g__uncultured bacterium;s__uncultured bacterium
OTU529	k__Bacteria; p__Bacteroidetes; c__Cytophagia; o__Cytophagales; f__Cytophagaceae; g__; s__	k__Bacteria;p__Bacteroidetes;c__Cytophagia;o__Cytophagales;f__Cytophagaceae;g__uncultured;s__uncultured bacterium
OTU53	k__Bacteria; p__Proteobacteria; c__Betaproteobacteria; o__Burkholderiales; f__Comamonadaceae; g__; s__	k__Bacteria;p__Proteobacteria;c__Betaproteobacteria;o__Burkholderiales;f__Comamonadaceae;g__Leptothrix;Ambiguous_taxa
OTU530	Unassigned	Unassigned
OTU531	k__Bacteria; p__Proteobacteria; c__Alphaproteobacteria; o__Rhodospirillales; f__; g__; s__	k__Bacteria;p__Proteobacteria;c__Alphaproteobacteria;o__Rhodospirillales;f__Rhodospirillales Incertae Sedis;g__Candidatus Alysiosphaera;s__uncultured bacterium
OTU532	k__Bacteria; p__Actinobacteria; c__Actinobacteria; o__Actinomycetales; f__Brevibacteriaceae; g__Brevibacterium; s__	k__Bacteria;p__Actinobacteria;c__Actinobacteria;o__Micrococcales;f__Brevibacteriaceae;g__Brevibacterium;s__uncultured bacterium
OTU533	k__Bacteria; p__Acidobacteria; c__Acidobacteria-6; o__iii1-15; f__; g__; s__	k__Bacteria;p__Acidobacteria;c__Subgroup 6
OTU534	k__Bacteria; p__Proteobacteria; c__Alphaproteobacteria; o__Rhodospirillales; f__Rhodospirillaceae; g__; s__	k__Bacteria;p__Proteobacteria;c__Alphaproteobacteria;o__Rhodospirillales;f__Rhodospirillales Incertae Sedis;g__Reyranella;Ambiguous_taxa
OTU535	Unassigned	Unassigned
OTU536	k__Bacteria; p__Cyanobacteria; c__Nostocophycideae; o__Nostocales; f__Scytonemataceae; g__Brasilonema	k__Bacteria;p__Cyanobacteria;c__Cyanobacteria;o__SubsectionI;f__FamilyI;g__Brasilonema;Ambiguous_taxa
OTU537	k__Bacteria; p__Proteobacteria; c__Alphaproteobacteria; o__Rickettsiales; f__mitochondria	k__Bacteria;p__Proteobacteria;c__Alphaproteobacteria;o__Rickettsiales;f__Mitochondria
OTU538	k__Bacteria; p__Cyanobacteria; c__Chloroplast; o__Chlorophyta; f__; g__; s__	k__Bacteria;p__Cyanobacteria;c__Chloroplast
OTU539	k__Bacteria; p__Proteobacteria; c__Alphaproteobacteria; o__Sphingomonadales; f__Sphingomonadaceae; g__Sphingomonas; s__	k__Bacteria;p__Proteobacteria;c__Alphaproteobacteria;o__Sphingomonadales;f__Sphingomonadaceae;g__Sphingomonas;Ambiguous_taxa
OTU54	k__Bacteria; p__Bacteroidetes; c__Sphingobacteriia; o__Sphingobacteriales; f__Sphingobacteriaceae; g__; s__	k__Bacteria;p__Bacteroidetes;c__Sphingobacteriia;o__Sphingobacteriales;f__Sphingobacteriaceae;g__Mucilaginibacter
OTU540	k__Bacteria; p__Actinobacteria; c__Actinobacteria; o__Actinomycetales; f__Actinosynnemataceae; g__Lentzea	k__Bacteria;p__Actinobacteria;c__Actinobacteria;o__Pseudonocardiales;f__Pseudonocardiaceae;Ambiguous_taxa;Ambiguous_taxa
OTU541	k__Bacteria; p__Proteobacteria; c__Alphaproteobacteria; o__; f__; g__; s__	k__Bacteria;p__Proteobacteria;c__Alphaproteobacteria;o__Rickettsiales;f__SM2D12;g__uncultured bacterium;s__uncultured bacterium
OTU542	k__Bacteria; p__Proteobacteria; c__Deltaproteobacteria; o__Bdellovibrionales; f__Bdellovibrionaceae; g__Bdellovibrio; s__	k__Bacteria;p__Proteobacteria;c__Deltaproteobacteria;o__Bdellovibrionales;f__Bdellovibrionaceae;g__Bdellovibrio;s__uncultured bacterium
OTU543	k__Bacteria; p__Proteobacteria; c__Betaproteobacteria; o__Burkholderiales; f__Comamonadaceae	k__Bacteria;p__Proteobacteria;c__Betaproteobacteria;o__Burkholderiales;f__Comamonadaceae;Ambiguous_taxa;Ambiguous_taxa
OTU544	k__Bacteria; p__Actinobacteria; c__Actinobacteria; o__Actinomycetales; f__Sporichthyaceae; g__; s__	k__Bacteria;p__Actinobacteria;c__Actinobacteria;o__Frankiales;f__uncultured
OTU545	Unassigned	Unassigned

OTU546	k__Bacteria; p__Actinobacteria; c__Actinobacteria; o__Actinomycetales; f__Geodermatophilaceae	k__Bacteria;p__Actinobacteria;c__Actinobacteria;o__Frankiales;f__Geodermatophilaceae;g__Blastococcus;s__uncultured bacterium
OTU547	k__Bacteria; p__Proteobacteria; c__Betaproteobacteria; o__Burkholderiales; f__Burkholderiaceae; g__Burkholderia; s__	k__Bacteria;p__Proteobacteria;c__Betaproteobacteria;o__Burkholderiales;f__Burkholderiaceae;g__Burkholderia-Paraburkholderia;Ambiguous_taxa
OTU548	k__Bacteria; p__FBP; c__; o__; f__; g__; s__	k__Bacteria;p__FBP;c__uncultured bacterium;o__uncultured bacterium;f__uncultured bacterium;g__uncultured bacterium;s__uncultured bacterium
OTU549	k__Bacteria; p__Proteobacteria; c__Alphaproteobacteria; o__Rhizobiales; f__; g__; s__	k__Bacteria;p__Proteobacteria;c__Alphaproteobacteria;o__Rhizobiales
OTU55	k__Bacteria; p__Firmicutes; c__Bacilli; o__Lactobacillales; f__Lactobacillaceae; g__Lactobacillus; s__	k__Bacteria;p__Firmicutes;c__Bacilli;o__Lactobacillales;f__Lactobacillaceae;g__Lactobacillus
OTU550	k__Bacteria; p__[Thermi]; c__Deinococci; o__Deinococcales; f__Deinococcaceae; g__Deinococcus; s__	k__Bacteria;p__Deinococcus-Thermus;c__Deinococci;o__Deinococcales;f__Deinococcaceae;g__Deinococcus;s__uncultured bacterium
OTU551	k__Bacteria; p__TM7; c__TM7-3; o__EW055; f__; g__; s__	k__Bacteria;p__Saccharibacteria;c__uncultured bacterium;o__uncultured bacterium;f__uncultured bacterium;g__uncultured bacterium;s__uncultured bacterium
OTU552	k__Bacteria; p__Cyanobacteria; c__Synechococcophycideae; o__Pseudanabaenales; f__Pseudanabaenaceae; g__Leptolyngbya; s__	k__Bacteria;p__Cyanobacteria;c__Cyanobacteria;o__SubsectionIII;f__FamilyI;g__Leptolyngbya
OTU553	k__Bacteria; p__Actinobacteria; c__Actinobacteria; o__Actinomycetales; f__Dermacoccaceae; g__Dermacoccus; s__	k__Bacteria;p__Actinobacteria;c__Actinobacteria;o__Micrococcales;f__Dermacoccaceae
OTU554	Unassigned	Unassigned
OTU555	k__Bacteria; p__Proteobacteria; c__Alphaproteobacteria; o__Rhizobiales; f__Beijerinckiaceae; g__; s__	k__Bacteria;p__Proteobacteria;c__Alphaproteobacteria;o__Rhizobiales;f__Methylobacteriaceae;g__uncultured;s__uncultured bacterium
OTU556	k__Bacteria; p__Firmicutes; c__Bacilli; o__Lactobacillales; f__Lactobacillaceae; g__Lactobacillus; s__	k__Bacteria;p__Firmicutes;c__Bacilli;o__Lactobacillales;f__Lactobacillaceae;g__Lactobacillus;Ambiguous_taxa
OTU557	k__Bacteria; p__Actinobacteria; c__Thermoleophilia; o__Solirubrobacterales; f__Solirubrobacteraceae; g__; s__	k__Bacteria;p__Actinobacteria;c__Thermoleophilia;o__Solirubrobacterales;f__Solirubrobacteraceae;g__Solirubrobacter;s__uncultured bacterium
OTU558	k__Bacteria; p__Cyanobacteria; c__; o__; f__; g__; s__	k__Bacteria;p__Cyanobacteria;c__Cyanobacteria;o__SubsectionIII;f__FamilyI
OTU559	k__Bacteria; p__Acidobacteria; c__[Chloracidobacteria]; o__RB41; f__Ellin6075; g__; s__	k__Bacteria;p__Acidobacteria;c__Blastocatellia;o__Blastocatellales;f__Blastocatellaceae (Subgroup 4)
OTU56	k__Bacteria; p__TM7; c__TM7-1; o__; f__; g__; s__	k__Bacteria;p__Saccharibacteria;c__uncultured bacterium;o__uncultured bacterium;f__uncultured bacterium;g__uncultured bacterium;s__uncultured bacterium
OTU560	k__Bacteria; p__Actinobacteria; c__Actinobacteria; o__Actinomycetales; f__Frankiaceae; g__; s__	k__Bacteria;p__Actinobacteria;c__Actinobacteria;o__Frankiales;f__Frankiaceae;g__Jatrophihabitans;Ambiguous_taxa
OTU561	k__Bacteria; p__Verrucomicrobia; c__[Spartobacteria]; o__[Chthoniobacterales]; f__[Chthoniobacteraceae]; g__Candidatus Xiphinematobacter; s__	k__Bacteria;p__Verrucomicrobia;c__Spartobacteria;o__Chthoniobacterales;f__Xiphinematobacteraceae;g__Candidatus Xiphinematobacter
OTU562	k__Bacteria; p__Actinobacteria; c__Thermoleophilia; o__Solirubrobacterales; f__; g__; s__	k__Bacteria;p__Actinobacteria;c__Thermoleophilia;o__Solirubrobacterales;f__Elev-16S-1332
OTU563	Unassigned	Unassigned

OTU564	Unassigned	Unassigned
OTU565	k__Bacteria; p__Proteobacteria; c__Alphaproteobacteria; o__Rhodospirillales; f__Acetobacteraceae; g__; s__	k__Bacteria;p__Proteobacteria;c__Alphaproteobacteria;o__Rhodospirillales;f__Acetobacteraceae;g__Granulibacter
OTU566	k__Bacteria; p__Planctomycetes; c__Planctomycetia; o__Pirellulales; f__Pirellulaceae; g__; s__	k__Bacteria;p__Planctomycetes;c__Planctomycetacia;o__Planctomycetales;f__Planctomycetaceae;g__Pir4 lineage;s__uncultured bacterium
OTU567	k__Bacteria; p__Acidobacteria; c__[Chloracidobacteria]; o__DS-100; f__; g__; s__	k__Bacteria;p__Acidobacteria;c__Blastocatellia;o__Blastocatellales;f__Blastocatellaceae (Subgroup 4);g__DS-100;s__uncultured bacterium
OTU568	k__Bacteria; p__Firmicutes; c__Clostridia; o__Clostridiales; f__Lachnospiraceae; g__[Ruminococcus]; s__gnavus	k__Bacteria;p__Firmicutes;c__Clostridia;o__Clostridiales;f__Lachnospiraceae;g__[Ruminococcus] gnavus group;s__uncultured organism
OTU569	Unassigned	Unassigned
OTU57	Unassigned	Unassigned
OTU570	k__Bacteria; p__Proteobacteria; c__Betaproteobacteria; o__Burkholderiales; f__Oxalobacteraceae; g__; s__	k__Bacteria;p__Proteobacteria;c__Betaproteobacteria;o__Burkholderiales;f__Oxalobacteraceae;g__Noviherbaspirillum;Ambiguous_taxa
OTU571	k__Bacteria; p__Proteobacteria; c__Alphaproteobacteria; o__Rhodobacterales; f__Rhodobacteraceae; g__Rubellimicrobium; s__	k__Bacteria;p__Proteobacteria;c__Alphaproteobacteria;o__Rhodobacterales;f__Rhodobacteraceae;g__Rubellimicrobium;Ambiguous_taxa
OTU572	k__Bacteria; p__Firmicutes; c__Bacilli; o__Lactobacillales; f__Lactobacillaceae; g__Lactobacillus; s__	k__Bacteria;p__Firmicutes;c__Bacilli;o__Lactobacillales;f__Lactobacillaceae;g__Lactobacillus;Ambiguous_taxa
OTU573	k__Bacteria; p__Proteobacteria; c__Betaproteobacteria; o__; f__; g__; s__	k__Bacteria;p__Proteobacteria;c__Betaproteobacteria;o__TRA3-20
OTU574	k__Bacteria; p__Proteobacteria; c__Alphaproteobacteria; o__Sphingomonadales; f__Sphingomonadaceae; g__; s__	k__Bacteria;p__Proteobacteria;c__Alphaproteobacteria;o__Sphingomonadales;Ambiguous_taxa;Ambiguous_taxa;Ambiguous_taxa
OTU575	Unassigned	Unassigned
OTU576	Unassigned	Unassigned
OTU577	k__Bacteria; p__Proteobacteria; c__Alphaproteobacteria; o__Rhodospirillales; f__Acetobacteraceae; g__; s__	k__Bacteria;p__Proteobacteria;c__Alphaproteobacteria;o__Rhodospirillales;f__Acetobacteraceae;g__uncultured;s__uncultured bacterium
OTU578	k__Bacteria; p__Proteobacteria; c__Alphaproteobacteria; o__Rhodospirillales; f__Acetobacteraceae	k__Bacteria;p__Proteobacteria;c__Alphaproteobacteria;o__Rhodospirillales;f__Acetobacteraceae
OTU579	k__Bacteria; p__Armatimonadetes; c__Armatimonadia; o__Armatimonadales; f__Armatimonadaceae; g__; s__	k__Bacteria;p__Armatimonadetes;c__Armatimonadia;o__Armatimonadales;f__uncultured Armatimonadetes bacterium;g__uncultured Armatimonadetes bacterium;s__uncultured Armatimonadetes bacterium
OTU58	k__Bacteria; p__Cyanobacteria; c__Nostocophycideae; o__Nostocales; f__Nostocaceae; g__Nostoc; s__	k__Bacteria;p__Cyanobacteria;c__Cyanobacteria;o__SubsectionIV;f__FamilyI;Ambiguous_taxa;Ambiguous_taxa
OTU580	k__Bacteria; p__Acidobacteria; c__[Chloracidobacteria]; o__PK29; f__; g__; s__	k__Bacteria;p__Acidobacteria;c__Blastocatellia;o__Blastocatellales;f__Blastocatellaceae (Subgroup 4);g__11-24;s__uncultured bacterium
OTU581	k__Bacteria; p__Firmicutes; c__Bacilli; o__Lactobacillales; f__Lactobacillaceae; g__Lactobacillus; s__agilis	k__Bacteria;p__Firmicutes;c__Bacilli;o__Lactobacillales;f__Lactobacillaceae;g__Lactobacillus;s__uncultured bacterium
OTU582	k__Bacteria; p__Proteobacteria; c__Alphaproteobacteria; o__Sphingomonadales; f__Sphingomonadaceae; g__Sphingomonas; s__	k__Bacteria;p__Proteobacteria;c__Alphaproteobacteria;o__Sphingomonadales;f__Sphingomonadaceae;g__Sphingomonas;Ambiguous_taxa
OTU583	k__Bacteria; p__Actinobacteria; c__Actinobacteria; o__Actinomycetales; f__Corynebacteriaceae; g__Corynebacterium; s__variabile	k__Bacteria;p__Actinobacteria;c__Actinobacteria;o__Corynebacteriales;f__Corynebacteriaceae;g__Corynebacterium 1
OTU584	k__Bacteria; p__Acidobacteria; c__Acidobacteria-6; o__iii1-15; f__; g__; s__	k__Bacteria;p__Acidobacteria;c__Subgroup 6;o__uncultured bacterium;f__uncultured bacterium;g__uncultured bacterium;s__uncultured bacterium

OTU585	k__Bacteria; p__Actinobacteria; c__Actinobacteria; o__Actinomycetales; f__Nocardioidaceae; g__Aeromicrobium; s__	k__Bacteria;p__Actinobacteria;c__Actinobacteria;o__Propionibacteriales;f__Nocardioidaceae;g__Aeromicrobium;Ambiguous_taxa
OTU586	Unassigned	Unassigned
OTU587	k__Bacteria; p__Armatimonadetes; c__Armatimonadia; o__Armatimonadales; f__Armatimonadaceae; g__; s__	k__Bacteria;p__Armatimonadetes;c__Armatimonadia;o__Armatimonadales;f__uncultured bacterium;g__uncultured bacterium;s__uncultured bacterium
OTU588	k__Bacteria; p__Proteobacteria; c__Alphaproteobacteria; o__Rhodobacterales; f__Rhodobacteraceae; g__; s__	k__Bacteria;p__Proteobacteria;c__Alphaproteobacteria;o__Rhodobacterales;f__Rhodobacteraceae
OTU589	k__Bacteria; p__Proteobacteria; c__Alphaproteobacteria; o__Sphingomonadales; f__Erythrobacteraceae; g__; s__	k__Bacteria;p__Proteobacteria;c__Alphaproteobacteria;o__Sphingomonadales;f__Erythrobacteraceae;g__Altererythrobacter;s__uncultured bacterium
OTU59	k__Bacteria; p__Cyanobacteria; c__Nostocophycideae; o__Nostocales; f__Nostocaceae; g__; s__	k__Bacteria;p__Cyanobacteria;c__Cyanobacteria;o__SubsectionIV;f__FamilyI;Ambiguous_taxa;Ambiguous_taxa
OTU590	k__Bacteria; p__Actinobacteria; c__Actinobacteria; o__Actinomycetales; f__Nocardioidaceae; g__Nocardioides; s__	k__Bacteria;p__Actinobacteria;c__Actinobacteria;o__Propionibacteriales;f__Nocardioidaceae;g__Nocardioides;s__uncultured bacterium
OTU591	Unassigned	Unassigned
OTU592	k__Bacteria; p__Cyanobacteria; c__Nostocophycideae; o__Nostocales; f__Nostocaceae; g__; s__	k__Bacteria;p__Cyanobacteria;c__Cyanobacteria;o__SubsectionIII;f__FamilyI;g__Crinalium;s__uncultured bacterium
OTU593	k__Bacteria; p__Bacteroidetes; c__Cytophagia; o__Cytophagales; f__Cytophagaceae; g__Spirosoma; s__	k__Bacteria;p__Bacteroidetes;c__Cytophagia;o__Cytophagales;f__Cytophagaceae;g__Spirosoma;s__uncultured bacterium
OTU594	Unassigned	Unassigned
OTU595	k__Bacteria; p__Proteobacteria; c__Betaproteobacteria; o__Burkholderiales; f__Comamonadaceae; g__; s__	k__Bacteria;p__Proteobacteria;c__Betaproteobacteria;o__Burkholderiales;f__Comamonadaceae
OTU596	Unassigned	Unassigned
OTU597	k__Bacteria; p__TM7; c__TM7-1; o__; f__; g__; s__	k__Bacteria;p__Saccharibacteria;c__uncultured bacterium;o__uncultured bacterium;f__uncultured bacterium;g__uncultured bacterium;s__uncultured bacterium
OTU598	k__Bacteria; p__TM7; c__TM7-3; o__; f__; g__; s__	k__Bacteria;p__Saccharibacteria;c__uncultured bacterium;o__uncultured bacterium;f__uncultured bacterium;g__uncultured bacterium;s__uncultured bacterium
OTU599	k__Bacteria; p__Verrucomicrobia; c__[Pedosphaerae]; o__[Pedosphaerales]; f__; g__; s__	k__Bacteria;p__Verrucomicrobia;c__OPB35 soil group;o__uncultured bacterium;f__uncultured bacterium;g__uncultured bacterium;s__uncultured bacterium
OTU6	k__Bacteria; p__Proteobacteria; c__Alphaproteobacteria; o__Sphingomonadales; f__Sphingomonadaceae; g__; s__	k__Bacteria;p__Proteobacteria;c__Alphaproteobacteria;o__Sphingomonadales
OTU60	k__Bacteria; p__Proteobacteria; c__Alphaproteobacteria; o__Sphingomonadales; f__Sphingomonadaceae; g__Sphingomonas; s__	k__Bacteria;p__Proteobacteria;c__Alphaproteobacteria;o__Sphingomonadales;f__Sphingomonadaceae;g__Sphingomonas;Ambiguous_taxa
OTU600	k__Bacteria; p__Bacteroidetes; c__Cytophagia; o__Cytophagales; f__Cytophagaceae; g__Spirosoma; s__	k__Bacteria;p__Bacteroidetes;c__Cytophagia;o__Cytophagales;f__Cytophagaceae;g__Fibrisoma;s__uncultured bacterium
OTU601	Unassigned	Unassigned
OTU602	k__Bacteria; p__Verrucomicrobia; c__[Spartobacteria]; o__[Chthoniobacterales]; f__[Chthoniobacteraceae]; g__DA101; s__	k__Bacteria;p__Verrucomicrobia;c__Spartobacteria;o__Chthoniobacterales;f__DA101 soil group;Ambiguous_taxa;Ambiguous_taxa
OTU603	k__Bacteria; p__Actinobacteria; c__Thermoleophilia; o__Gaiellales; f__Gaiellaceae; g__; s__	k__Bacteria;p__Actinobacteria;c__Thermoleophilia;o__Gaiellales;f__uncultured;g__uncultured bacterium;s__uncultured bacterium

OTU604	Unassigned	Unassigned
OTU605	Unassigned	Unassigned
OTU606	k__Bacteria; p__Proteobacteria; c__Deltaproteobacteria; o__Myxococcales; f__; g__; s__	k__Bacteria;p__Proteobacteria;c__Deltaproteobacteria;o__Myxococcales;f__Polyangiaceae;g__Sorangium;Ambiguous_taxa
OTU607	k__Bacteria; p__Proteobacteria; c__Alphaproteobacteria; o__Rhodospirillales; f__Acetobacteraceae; g__; s__	k__Bacteria;p__Proteobacteria;c__Alphaproteobacteria;o__Rhodospirillales;f__Acetobacteraceae;g__Roseomonas;Ambiguous_taxa
OTU608	k__Bacteria; p__Proteobacteria; c__Alphaproteobacteria; o__Sphingomonadales; f__Sphingomonadaceae; g__Sphingomonas; s__wittichii	k__Bacteria;p__Proteobacteria;c__Alphaproteobacteria;o__Sphingomonadales;f__Sphingomonadaceae;g__Sphingomonas
OTU609	k__Bacteria; p__Bacteroidetes; c__Cytophagia; o__Cytophagales; f__Cytophagaceae; g__Spirosoma; s__	k__Bacteria;p__Bacteroidetes;c__Cytophagia;o__Cytophagales;f__Cytophagaceae;g__Spirosoma
OTU61	k__Bacteria; p__Cyanobacteria; c__Oscillatoriophycideae; o__Chroococcales; f__Xenococcaceae; g__; s__	k__Bacteria;p__Cyanobacteria;c__Cyanobacteria;o__SubsectionII;f__FamilyII;g__Chroococcidiopsis;s__uncultured bacterium
OTU610	Unassigned	Unassigned
OTU611	k__Bacteria; p__Cyanobacteria; c__Chloroplast; o__Streptophyta; f__; g__; s__	k__Bacteria;p__Cyanobacteria;c__Chloroplast
OTU612	Unassigned	Unassigned
OTU613	k__Bacteria; p__Proteobacteria; c__Alphaproteobacteria; o__Rhodobacterales; f__Rhodobacteraceae; g__Paracoccus	k__Bacteria;p__Proteobacteria;c__Alphaproteobacteria;o__Rhodobacterales;f__Rhodobacteraceae;g__Paracoccus;s__uncultured bacterium
OTU614	k__Bacteria; p__Proteobacteria; c__Alphaproteobacteria; o__Sphingomonadales; f__Sphingomonadaceae; g__; s__	k__Bacteria;p__Proteobacteria;c__Alphaproteobacteria;o__Sphingomonadales;f__Sphingomonadaceae;g__Rhizorhapis;Ambiguous_taxa
OTU615	Unassigned	Unassigned
OTU616	k__Bacteria; p__Acidobacteria; c__Acidobacteriia; o__Acidobacteriales; f__Acidobacteriaceae; g__; s__	k__Bacteria;p__Acidobacteria;c__Acidobacteria;o__Acidobacteriales;f__Acidobacteriaceae (Subgroup 1);g__Bryocella
OTU617	k__Bacteria; p__Proteobacteria; c__Alphaproteobacteria; o__Rhizobiales; f__Rhizobiaceae; g__Agrobacterium; s__	k__Bacteria;p__Proteobacteria;c__Alphaproteobacteria;o__Rhizobiales;f__Phyllobacteriaceae;g__Mesorhizobium
OTU618	k__Bacteria; p__Actinobacteria; c__Thermoleophilia; o__Solirubrobacterales; f__; g__; s__	k__Bacteria;p__Actinobacteria;c__Thermoleophilia;o__Solirubrobacterales;f__Elev-16S-1332;g__uncultured bacterium;s__uncultured bacterium
OTU619	k__Bacteria; p__Actinobacteria; c__Actinobacteria; o__Actinomycetales; f__Micrococcaceae; g__Kocuria; s__palustris	k__Bacteria;p__Actinobacteria;c__Actinobacteria;o__Micrococcales;f__Micrococcaceae;g__Kocuria
OTU62	k__Bacteria; p__Actinobacteria; c__Actinobacteria; o__Actinomycetales; f__Nocardioidaceae; g__Propionicimonas; s__	k__Bacteria;p__Actinobacteria;c__Actinobacteria;o__Propionibacteriales;f__Propionibacteriaceae;g__Propioniciclava;s__uncultured bacterium
OTU620	k__Bacteria; p__Acidobacteria; c__Acidobacteria-6; o__iii1-15; f__; g__; s__	k__Bacteria;p__Acidobacteria;c__Subgroup 6;o__uncultured bacterium;f__uncultured bacterium;g__uncultured bacterium;s__uncultured bacterium
OTU621	k__Bacteria; p__Bacteroidetes; c__Flavobacteriia; o__Flavobacteriales; f__Flavobacteriaceae; g__Capnocytophaga; s__	k__Bacteria;p__Bacteroidetes;c__Flavobacteriia;o__Flavobacteriales;f__Flavobacteriaceae;g__Capnocytophaga;s__uncultured bacterium
OTU622	k__Bacteria; p__Proteobacteria; c__Alphaproteobacteria; o__Rhodospirillales; f__Acetobacteraceae; g__Gluconobacter; s__	k__Bacteria;p__Proteobacteria;c__Alphaproteobacteria;o__Rhodospirillales;f__Acetobacteraceae;g__Gluconobacter
OTU623	k__Bacteria; p__Proteobacteria; c__Alphaproteobacteria; o__Rhodospirillales; f__Acetobacteraceae	k__Bacteria;p__Proteobacteria;c__Alphaproteobacteria;o__Rhodospirillales;f__Acetobacteraceae
OTU624	Unassigned	Unassigned

OTU625	k__Bacteria; p__Cyanobacteria; c__Nostocophycideae; o__Nostocales; f__Scytonemataceae; g__Scytonema; s__	k__Bacteria;p__Cyanobacteria;c__Cyanobacteria
OTU626	k__Bacteria; p__TM7; c__; o__; f__; g__; s__	k__Bacteria;p__Saccharibacteria;c__uncultured bacterium;o__uncultured bacterium;f__uncultured bacterium;g__uncultured bacterium;s__uncultured bacterium
OTU627	Unassigned	Unassigned
OTU628	k__Bacteria; p__Firmicutes; c__Bacilli; o__Bacillales; f__Bacillaceae; g__Bacillus; s__flexus	k__Bacteria;p__Firmicutes;c__Bacilli;o__Bacillales;f__Bacillaceae;g__Bacillus
OTU629	k__Bacteria; p__Proteobacteria; c__Gammaproteobacteria; o__Xanthomonadales; f__Xanthomonadaceae; g__; s__	k__Bacteria;p__Proteobacteria;c__Gammaproteobacteria;o__Xanthomonadales;f__Xanthomonadaceae
OTU63	k__Bacteria; p__Proteobacteria; c__Gammaproteobacteria; o__Xanthomonadales; f__Xanthomonadaceae	k__Bacteria;p__Proteobacteria;c__Gammaproteobacteria;o__Xanthomonadales;f__Xanthomonadaceae;g__Ignatzschineria;Ambiguous_taxa
OTU630	Unassigned	Unassigned
OTU631	k__Bacteria; p__Firmicutes; c__Clostridia; o__Clostridiales; f__Veillonellaceae; g__Megasphaera; s__	k__Bacteria;p__Firmicutes;c__Negativicutes;o__Selenomonadales;f__Veillonellaceae;g__Megasphaera
OTU632	Unassigned	Unassigned
OTU633	k__Bacteria; p__Proteobacteria; c__Gammaproteobacteria; o__Enterobacteriales; f__Enterobacteriaceae	k__Bacteria;p__Proteobacteria;c__Gammaproteobacteria;o__Enterobacteriales;f__Enterobacteriaceae;g__Arsenophonus
OTU634	k__Bacteria; p__Proteobacteria; c__Alphaproteobacteria; o__Sphingomonadales; f__Sphingomonadaceae; g__Sphingomonas; s__	k__Bacteria;p__Proteobacteria;c__Alphaproteobacteria;o__Sphingomonadales;f__Sphingomonadaceae;g__Sphingomonas
OTU635	k__Bacteria; p__Proteobacteria; c__Alphaproteobacteria; o__BD7-3; f__; g__; s__	k__Bacteria;p__Proteobacteria;c__Alphaproteobacteria;o__Alphaproteobacteria Incertae Sedis;f__Unknown Family;g__uncultured;s__uncultured bacterium
OTU636	k__Bacteria; p__Proteobacteria; c__Alphaproteobacteria; o__Rhodospirillales; f__Acetobacteraceae	k__Bacteria;p__Proteobacteria;c__Alphaproteobacteria;o__Rhodospirillales;f__Acetobacteraceae
OTU637	k__Bacteria; p__Proteobacteria; c__Alphaproteobacteria; o__Rhodospirillales; f__Acetobacteraceae	k__Bacteria;p__Proteobacteria;c__Alphaproteobacteria;o__Rhodospirillales;f__Acetobacteraceae;g__Gluconobacter;Ambiguous_taxa
OTU638	k__Bacteria; p__Firmicutes; c__Bacilli; o__Lactobacillales; f__Lactobacillaceae; g__Lactobacillus; s__	k__Bacteria;p__Firmicutes;c__Bacilli;o__Lactobacillales;f__Lactobacillaceae;g__Lactobacillus;s__uncultured Lactobacillus sp.
OTU639	k__Bacteria; p__Chloroflexi; c__Chloroflexi; o__[Roseiflexales]; f__; g__; s__	k__Bacteria;p__Chloroflexi;c__Chloroflexia;o__Chloroflexales;f__Roseiflexaceae;g__Roseiflexus;s__uncultured bacterium
OTU64	k__Bacteria; p__Nitrospirae; c__Nitrospira; o__Nitrospirales; f__0319-6A21; g__; s__	k__Bacteria;p__Nitrospirae;c__Nitrospira;o__Nitrospirales;f__0319-6A21;g__uncultured bacterium;s__uncultured bacterium
OTU640	k__Bacteria; p__Proteobacteria; c__Alphaproteobacteria; o__Rhodobacterales; f__Rhodobacteraceae; g__Rubellimicrobium; s__	k__Bacteria;p__Proteobacteria;c__Alphaproteobacteria;o__Rhodobacterales;f__Rhodobacteraceae;g__Rubellimicrobium;s__uncultured bacterium
OTU641	k__Bacteria; p__Proteobacteria; c__Alphaproteobacteria; o__Rhodospirillales; f__Acetobacteraceae; g__; s__	k__Bacteria;p__Proteobacteria;c__Alphaproteobacteria;o__Rhodospirillales;f__Acetobacteraceae
OTU642	k__Bacteria; p__Proteobacteria; c__Gammaproteobacteria; o__Oceanospirillales; f__Halomonadaceae; g__Candidatus Portiera; s__	k__Bacteria;p__Proteobacteria;c__Gammaproteobacteria;o__Oceanospirillales;f__Halomonadaceae
OTU643	Unassigned	Unassigned
OTU644	k__Bacteria; p__Proteobacteria; c__Alphaproteobacteria; o__Rhizobiales; f__Hyphomicrobiaceae; g__Rhodoplanes; s__	k__Bacteria;p__Proteobacteria;c__Alphaproteobacteria;o__Rhizobiales;f__Xanthobacteraceae;g__Variibacter

OTU645	k__Bacteria; p__Bacteroidetes; c__Cytophagia; o__Cytophagales; f__Cytophagaceae; g__Rudanella; s__	k__Bacteria;p__Bacteroidetes;c__Cytophagia;o__Cytophagales;f__Cytophagaceae;g__Rudanella
OTU646	k__Bacteria; p__Proteobacteria; c__Alphaproteobacteria; o__Rhizobiales; f__Methylobacteriaceae; g__; s__	k__Bacteria;p__Proteobacteria;c__Alphaproteobacteria;o__Rhizobiales;f__Methylobacteriaceae;g__Methylobacterium;Ambiguous_taxa
OTU647	Unassigned	Unassigned
OTU648	k__Bacteria; p__Actinobacteria; c__Thermoleophilia; o__Gaiellales; f__Gaiellaceae; g__; s__	k__Bacteria;p__Actinobacteria;c__Thermoleophilia;o__Gaiellales;f__Gaiellaceae;g__Gaiella;Ambiguous_taxa
OTU649	k__Bacteria; p__Bacteroidetes; c__[Saprospirae]; o__[Saprospirales]; f__Chitinophagaceae; g__Segetibacter; s__	k__Bacteria;p__Bacteroidetes;c__Sphingobacteriia;o__Sphingobacteriales;f__Chitinophagaceae;g__Ferruginibacter
OTU65	Unassigned	Unassigned
OTU650	Unassigned	Unassigned
OTU651	k__Bacteria; p__Planctomycetes; c__Planctomycetia; o__Gemmatales; f__Gemmataceae; g__Gemmata; s__	k__Bacteria;p__Planctomycetes;c__Planctomycetacia;o__Planctomycetales;f__Planctomycetaceae;g__Gemmata;Ambiguous_taxa
OTU652	k__Bacteria; p__Bacteroidetes; c__Cytophagia; o__Cytophagales; f__Cytophagaceae; g__Larkinella; s__	k__Bacteria;p__Bacteroidetes;c__Cytophagia;o__Cytophagales;f__Cytophagaceae;g__Larkinella
OTU653	k__Bacteria; p__Actinobacteria; c__Actinobacteria; o__Actinomycetales; f__; g__; s__	k__Bacteria;p__Actinobacteria;c__Actinobacteria;o__Kineosporiales;f__Kineosporiaceae;g__Quadrisphaera
OTU654	k__Bacteria; p__Proteobacteria; c__Deltaproteobacteria; o__Myxococcales; f__Haliangiaceae; g__; s__	k__Bacteria;p__Proteobacteria;c__Deltaproteobacteria;o__Myxococcales;f__Haliangiaceae;g__Haliangium
OTU655	Unassigned	Unassigned
OTU656	k__Bacteria; p__Firmicutes; c__Bacilli; o__Lactobacillales; f__Lactobacillaceae; g__Lactobacillus; s__	k__Bacteria;p__Firmicutes;c__Bacilli;o__Lactobacillales;f__Lactobacillaceae;g__Lactobacillus;Ambiguous_taxa
OTU657	k__Bacteria; p__Proteobacteria; c__Betaproteobacteria; o__Hydrogenophilales; f__Hydrogenophilaceae; g__Thiobacillus; s__	k__Bacteria;p__Proteobacteria;c__Betaproteobacteria;o__Hydrogenophilales;f__Hydrogenophilaceae;g__Thiobacillus;s__uncultured bacterium
OTU658	k__Bacteria; p__Acidobacteria; c__[Chloracidobacteria]; o__RB41; f__Ellin6075; g__; s__	k__Bacteria;p__Acidobacteria;c__Blastocatellia;o__Blastocatellales;f__Blastocatellaceae (Subgroup 4);g__uncultured;s__uncultured bacterium
OTU659	k__Bacteria; p__Actinobacteria; c__Actinobacteria; o__Actinomycetales; f__Nocardioidaceae; g__Nocardioides; s__	k__Bacteria;p__Actinobacteria;c__Actinobacteria;o__Propionibacteriales;f__Nocardioidaceae;g__Nocardioides;Ambiguous_taxa
OTU66	k__Bacteria; p__Planctomycetes; c__Planctomycetia; o__Gemmatales; f__Gemmataceae; g__; s__	k__Bacteria;p__Planctomycetes;c__Planctomycetacia;o__Planctomycetales;f__Planctomycetaceae;g__uncultured;s__uncultured bacterium
OTU660	k__Bacteria; p__Actinobacteria; c__Actinobacteria; o__Actinomycetales; f__Nocardioidaceae; g__; s__	k__Bacteria;p__Actinobacteria;c__Actinobacteria;o__Propionibacteriales;f__Nocardioidaceae;g__Nocardioides;Ambiguous_taxa
OTU661	k__Bacteria; p__Firmicutes; c__Clostridia; o__Clostridiales; f__Ruminococcaceae; g__; s__	k__Bacteria;p__Firmicutes;c__Clostridia;o__Clostridiales;f__Ruminococcaceae;g__Candidatus Soleaferrea
OTU662	k__Bacteria; p__Cyanobacteria; c__Chloroplast; o__Stramenopiles; f__; g__; s__	k__Bacteria;p__Cyanobacteria;c__Chloroplast
OTU663	Unassigned	Unassigned
OTU664	k__Bacteria; p__Chloroflexi; c__Gitt-GS-136; o__; f__; g__; s__	k__Bacteria;p__Chloroflexi;c__Gitt-GS-136;Ambiguous_taxa;Ambiguous_taxa;Ambiguous_taxa;Ambiguous_taxa
OTU665	k__Bacteria; p__Proteobacteria; c__Alphaproteobacteria; o__Rhizobiales; f__Methylobacteriaceae; g__; s__	k__Bacteria;p__Proteobacteria;c__Alphaproteobacteria;o__Rhizobiales;f__Methylobacteriaceae;g__Methylobacterium;Ambiguous_taxa

OTU666	k__Bacteria; p__Actinobacteria; c__Actinobacteria; o__Actinomycetales; f__Microbacteriaceae; g__Microbacterium; s__	k__Bacteria;p__Actinobacteria;c__Actinobacteria;o__Micrococcales;f__Microbacteriaceae;g__Microbacterium
OTU667	k__Bacteria; p__Armatimonadetes; c__Armatimonadia; o__Armatimonadales; f__Armatimonadaceae; g__; s__	k__Bacteria;p__Armatimonadetes;c__Armatimonadia;o__Armatimonadales;f__uncultured bacterium;g__uncultured bacterium;s__uncultured bacterium
OTU668	Unassigned	Unassigned
OTU669	k__Bacteria; p__Bacteroidetes; c__Bacteroidia; o__Bacteroidales; f__Bacteroidaceae; g__Bacteroides; s__	k__Bacteria;p__Bacteroidetes;c__Bacteroidia;o__Bacteroidales;f__Bacteroidaceae;g__Bacteroides
OTU67	k__Bacteria; p__Proteobacteria; c__Alphaproteobacteria; o__Rhizobiales; f__; g__; s__	k__Bacteria;p__Proteobacteria;c__Alphaproteobacteria;o__Rhizobiales;f__Rhizobiales Incertae Sedis;g__Phreatobacter;s__uncultured bacterium
OTU670	k__Bacteria; p__Proteobacteria; c__Alphaproteobacteria; o__Caulobacterales; f__Caulobacteraceae; g__; s__	k__Bacteria;p__Proteobacteria;c__Alphaproteobacteria;o__Caulobacterales;f__Caulobacteraceae;g__uncultured
OTU671	k__Bacteria; p__Proteobacteria; c__Betaproteobacteria; o__Burkholderiales; f__Alcaligenaceae; g__Achromobacter; s__	k__Bacteria;p__Proteobacteria;c__Betaproteobacteria;o__Burkholderiales;f__Alcaligenaceae;g__Achromobacter
OTU672	k__Bacteria; p__Firmicutes; c__Clostridia; o__Clostridiales; f__Christensenellaceae; g__; s__	k__Bacteria;p__Firmicutes;c__Clostridia;o__Clostridiales;f__Christensenellaceae;g__Christensenellaceae R-7 group;s__uncultured bacterium
OTU673	Unassigned	Unassigned
OTU674	Unassigned	Unassigned
OTU675	k__Bacteria; p__Actinobacteria; c__Acidimicrobiia; o__Acidimicrobiales; f__C111; g__; s__	k__Bacteria;p__Actinobacteria;c__Acidimicrobiia;o__Acidimicrobiales;f__Acidimicrobiaceae;g__uncultured;s__uncultured bacterium
OTU676	k__Bacteria; p__Firmicutes; c__Bacilli; o__Lactobacillales; f__Enterococcaceae; g__Enterococcus; s__	k__Bacteria;p__Firmicutes;c__Bacilli;o__Lactobacillales;f__Enterococcaceae;g__Enterococcus
OTU677	Unassigned	Unassigned
OTU678	k__Bacteria; p__Bacteroidetes; c__Flavobacteriia; o__Flavobacteriales; f__[Weeksellaceae]; g__Cloacibacterium; s__	k__Bacteria;p__Bacteroidetes;c__Flavobacteriia;o__Flavobacteriales;f__Flavobacteriaceae;g__Cloacibacterium;s__uncultured bacterium
OTU679	k__Bacteria; p__Proteobacteria; c__Gammaproteobacteria; o__Xanthomonadales; f__Xanthomonadaceae; g__Dokdonella; s__	k__Bacteria;p__Proteobacteria;c__Gammaproteobacteria;o__Xanthomonadales;f__Xanthomonadaceae
OTU68	k__Bacteria; p__Proteobacteria; c__Gammaproteobacteria; o__Pseudomonadales; f__Moraxellaceae; g__Acinetobacter; s__	k__Bacteria;p__Proteobacteria;c__Gammaproteobacteria;o__Pseudomonadales;f__Moraxellaceae;g__Acinetobacter
OTU680	k__Bacteria; p__Proteobacteria; c__Alphaproteobacteria; o__Sphingomonadales; f__Sphingomonadaceae; g__Sphingomonas; s__	k__Bacteria;p__Proteobacteria;c__Alphaproteobacteria;o__Sphingomonadales;f__Sphingomonadaceae;g__Sphingomonas
OTU681	k__Bacteria; p__Proteobacteria; c__Gammaproteobacteria; o__Enterobacteriales; f__Enterobacteriaceae	k__Bacteria;p__Proteobacteria;c__Gammaproteobacteria;o__Enterobacteriales;f__Enterobacteriaceae
OTU682	k__Bacteria; p__TM7; c__TM7-1; o__; f__; g__; s__	k__Bacteria;p__Saccharibacteria;c__uncultured bacterium;o__uncultured bacterium;f__uncultured bacterium;g__uncultured bacterium;s__uncultured bacterium
OTU683	k__Bacteria; p__Proteobacteria; c__Alphaproteobacteria; o__Sphingomonadales; f__Sphingomonadaceae; g__; s__	k__Bacteria;p__Proteobacteria;c__Alphaproteobacteria;o__Sphingomonadales;f__Sphingomonadaceae;g__Sphingomonas;s__uncultured bacterium
OTU684	k__Bacteria; p__Proteobacteria; c__Alphaproteobacteria; o__Rhodospirillales; f__Acetobacteraceae; g__; s__	k__Bacteria;p__Proteobacteria;c__Alphaproteobacteria;o__Rhodospirillales;f__Acetobacteraceae;g__Acidisphaera
OTU685	k__Bacteria; p__Verrucomicrobia; c__[Spartobacteria]; o__[Chthoniobacterales]; f__[Chthoniobacteraceae]; g__; s__	k__Bacteria;p__Verrucomicrobia;c__Spartobacteria;o__Chthoniobacterales;f__LD29;g__uncultured bacterium;s__uncultured bacterium

OTU686	k__Bacteria; p__Proteobacteria; c__Alphaproteobacteria; o__Rhizobiales; f__Methylobacteriaceae; g__Methylobacterium; s__	k__Bacteria;p__Proteobacteria;c__Alphaproteobacteria;o__Rhizobiales;f__Methylobacteriaceae;g__Methylobacterium;Ambiguous_taxa
OTU687	k__Bacteria; p__Firmicutes; c__Bacilli; o__Lactobacillales; f__Streptococcaceae; g__Streptococcus; s__	k__Bacteria;p__Firmicutes;c__Bacilli;o__Lactobacillales;f__Streptococcaceae;g__Streptococcus;s__uncultured bacterium
OTU688	k__Bacteria; p__Proteobacteria; c__Alphaproteobacteria; o__Rhizobiales; f__Methylocystaceae; g__; s__	k__Bacteria;p__Proteobacteria;c__Alphaproteobacteria;o__Rhizobiales;f__Beijerinckiaceae
OTU689	k__Bacteria; p__Proteobacteria; c__Alphaproteobacteria; o__Rhizobiales; f__Aurantimonadaceae; g__; s__	k__Bacteria;p__Proteobacteria;c__Alphaproteobacteria;o__Rhizobiales;f__Aurantimonadaceae;g__Aureimonas;s__uncultured bacterium
OTU69	Unassigned	Unassigned
OTU690	k__Bacteria; p__Actinobacteria; c__Actinobacteria; o__Actinomycetales; f__Nocardioidaceae; g__; s__	k__Bacteria;p__Actinobacteria;c__Actinobacteria;o__Propionibacteriales;f__Nocardioidaceae;g__Nocardioides
OTU691	k__Bacteria; p__Proteobacteria; c__Alphaproteobacteria; o__Rhodospirillales; f__Acetobacteraceae; g__; s__	k__Bacteria;p__Proteobacteria;c__Alphaproteobacteria;o__Rhodospirillales;f__Acetobacteraceae;g__uncultured;s__uncultured bacterium
OTU692	k__Bacteria; p__Actinobacteria; c__Actinobacteria; o__Actinomycetales; f__Nocardioidaceae; g__Nocardioides; s__	k__Bacteria;p__Actinobacteria;c__Actinobacteria;o__Propionibacteriales;f__Nocardioidaceae;g__Nocardioides;Ambiguous_taxa
OTU693	k__Bacteria; p__Proteobacteria; c__Alphaproteobacteria; o__Sphingomonadales; f__Sphingomonadaceae; g__Kaistobacter; s__	k__Bacteria;p__Proteobacteria;c__Alphaproteobacteria;o__Sphingomonadales;f__Sphingomonadaceae;g__Sphingomonas
OTU694	Unassigned	Unassigned
OTU695	k__Bacteria; p__Bacteroidetes; c__Cytophagia; o__Cytophagales; f__Cytophagaceae; g__; s__	k__Bacteria;p__Bacteroidetes;c__Cytophagia;o__Cytophagales;f__Cytophagaceae;g__Siphonobacter;Ambiguous_taxa
OTU696	k__Bacteria; p__Cyanobacteria; c__Nostocophycideae; o__Nostocales; f__Scytonemataceae; g__; s__	k__Bacteria;p__Cyanobacteria;c__Cyanobacteria;o__SubsectionI;f__FamilyI;g__Brasilonema;Ambiguous_taxa
OTU697	k__Bacteria; p__Planctomycetes; c__Planctomycetia; o__Planctomycetales; f__Planctomycetaceae; g__Planctomyces; s__	k__Bacteria;p__Planctomycetes;c__Planctomycetacia;o__Planctomycetales;f__Planctomycetaceae;g__Planctomyces;s__uncultured bacterium
OTU698	k__Bacteria; p__Actinobacteria; c__Actinobacteria; o__Actinomycetales; f__Pseudonocardiaceae; g__Saccharopolyspora; s__	k__Bacteria;p__Actinobacteria;c__Actinobacteria;o__Pseudonocardiales;f__Pseudonocardiaceae;g__Saccharopolyspora;Ambiguous_taxa
OTU699	k__Bacteria; p__Proteobacteria; c__Deltaproteobacteria; o__Myxococcales; f__Polyangiaceae; g__; s__	k__Bacteria;p__Proteobacteria;c__Deltaproteobacteria;o__Myxococcales;f__Polyangiaceae;g__uncultured;s__uncultured bacterium
OTU7	k__Bacteria; p__Bacteroidetes; c__Cytophagia; o__Cytophagales; f__Cytophagaceae; g__; s__	k__Bacteria;p__Bacteroidetes;c__Cytophagia;o__Cytophagales;f__Cytophagaceae;g__uncultured;s__uncultured bacterium
OTU70	k__Bacteria; p__Acidobacteria; c__Acidobacteriia; o__Acidobacteriales; f__Acidobacteriaceae; g__; s__	k__Bacteria;p__Acidobacteria;c__Acidobacteria;o__Acidobacteriales;f__Acidobacteriaceae (Subgroup 1);g__uncultured;s__uncultured bacterium
OTU700	k__Bacteria; p__Proteobacteria; c__Alphaproteobacteria; o__Rhodospirillales; f__Acetobacteraceae; g__; s__	k__Bacteria;p__Proteobacteria;c__Alphaproteobacteria;o__Rhodospirillales;f__Acetobacteraceae;g__uncultured;s__uncultured Acetobacteraceae bacterium
OTU701	k__Bacteria; p__Proteobacteria; c__Alphaproteobacteria; o__Sphingomonadales; f__Sphingomonadaceae; g__Sphingomonas; s__	k__Bacteria;p__Proteobacteria;c__Alphaproteobacteria;o__Sphingomonadales;f__Sphingomonadaceae;g__Sphingomonas;Ambiguous_taxa
OTU702	k__Bacteria; p__Actinobacteria; c__Actinobacteria; o__Actinomycetales; f__Nocardioidaceae; g__Propionicimonas; s__	k__Bacteria;p__Actinobacteria;c__Actinobacteria;o__Propionibacteriales;f__Propionibacteriaceae;g__Naumannella
OTU703	k__Bacteria; p__Cyanobacteria; c__Synechococcophycideae; o__Pseudanabaenales; f__Pseudanabaenaceae; g__Leptolyngbya; s__	k__Bacteria;p__Cyanobacteria;c__Cyanobacteria;o__SubsectionIII;f__FamilyI;g__Leptolyngbya;s__uncultured bacterium

OTU704	Unassigned	k__Bacteria;p__WS6;c__uncultured bacterium;o__uncultured bacterium;f__uncultured bacterium;g__uncultured bacterium;s__uncultured bacterium
OTU705	Unassigned	Unassigned
OTU706	k__Bacteria; p__Proteobacteria; c__Alphaproteobacteria; o__Sphingomonadales; f__Sphingomonadaceae; g__Kaistobacter; s__	k__Bacteria;p__Proteobacteria;c__Alphaproteobacteria;o__Sphingomonadales;f__Sphingomonadaceae;g__Sphingomonas
OTU707	k__Bacteria; p__Proteobacteria; c__Alphaproteobacteria; o__Sphingomonadales; f__Sphingomonadaceae; g__; s__	k__Bacteria;p__Proteobacteria;c__Alphaproteobacteria;o__Sphingomonadales;f__Ellin6055;g__uncultured bacterium;s__uncultured bacterium
OTU708	k__Bacteria; p__Cyanobacteria; c__Oscillatoriophycideae; o__Chroococcales; f__Xenococcaceae; g__; s__	k__Bacteria;p__Cyanobacteria;c__Cyanobacteria;o__SubsectionII;f__FamilyII;g__Chroococcidiopsis;Ambiguous_taxa
OTU709	k__Bacteria; p__Proteobacteria; c__Gammaproteobacteria; o__Pseudomonadales; f__Moraxellaceae; g__Psychrobacter; s__pacificensis	k__Bacteria;p__Proteobacteria;c__Gammaproteobacteria;o__Pseudomonadales;f__Moraxellaceae;g__Psychrobacter;s__uncultured bacterium
OTU71	k__Bacteria; p__Proteobacteria; c__Gammaproteobacteria; o__Xanthomonadales; f__Xanthomonadaceae; g__Luteimonas; s__	k__Bacteria;p__Proteobacteria;c__Gammaproteobacteria;o__Xanthomonadales;f__Xanthomonadaceae;g__Luteimonas
OTU710	k__Bacteria; p__Bacteroidetes; c__[Saprospirae]; o__[Saprospirales]; f__Chitinophagaceae; g__Flavisolibacter; s__	k__Bacteria;p__Bacteroidetes;c__Sphingobacteriia;o__Sphingobacteriales;f__Chitinophagaceae;g__Flavisolibacter
OTU711	Unassigned	Unassigned
OTU712	k__Bacteria; p__Bacteroidetes; c__Cytophagia; o__Cytophagales; f__Cytophagaceae; g__Hymenobacter; s__	k__Bacteria;p__Bacteroidetes;c__Cytophagia;o__Cytophagales;f__Cytophagaceae;g__Hymenobacter;s__uncultured bacterium
OTU713	Unassigned	Unassigned
OTU714	k__Bacteria; p__TM7; c__TM7-1; o__; f__; g__; s__	k__Bacteria;p__Saccharibacteria;c__uncultured bacterium;o__uncultured bacterium;f__uncultured bacterium;g__uncultured bacterium;s__uncultured bacterium
OTU715	k__Bacteria; p__Bacteroidetes; c__Cytophagia; o__Cytophagales; f__Cytophagaceae; g__Dyadobacter; s__	k__Bacteria;p__Bacteroidetes;c__Cytophagia;o__Cytophagales;f__Cytophagaceae;g__Dyadobacter;Ambiguous_taxa
OTU716	k__Bacteria; p__Planctomycetes; c__Phycisphaerae; o__WD2101; f__; g__; s__	k__Bacteria;p__Planctomycetes;c__Phycisphaerae;o__Tepidisphaerales;f__Tepidisphaeraceae;g__uncultured bacterium;s__uncultured bacterium
OTU717	k__Bacteria; p__Firmicutes; c__Bacilli; o__Lactobacillales; f__Streptococcaceae; g__Streptococcus; s__	k__Bacteria;p__Firmicutes;c__Bacilli;o__Lactobacillales;f__Streptococcaceae;g__Streptococcus;s__uncultured bacterium
OTU718	Unassigned	Unassigned
OTU719	k__Bacteria; p__Verrucomicrobia; c__[Spartobacteria]; o__[Chthoniobacterales]; f__[Chthoniobacteraceae]; g__DA101; s__	k__Bacteria;p__Verrucomicrobia;c__Spartobacteria;o__Chthoniobacterales;f__DA101 soil group;g__uncultured bacterium;s__uncultured bacterium
OTU72	Unassigned	Unassigned
OTU720	k__Bacteria; p__Bacteroidetes; c__Cytophagia; o__Cytophagales; f__Cytophagaceae; g__Spirosoma; s__	k__Bacteria;p__Bacteroidetes;c__Cytophagia;o__Cytophagales;f__Cytophagaceae;g__Spirosoma;s__uncultured bacterium
OTU721	k__Bacteria; p__Proteobacteria; c__Alphaproteobacteria; o__Sphingomonadales; f__Erythrobacteraceae; g__; s__	k__Bacteria;p__Proteobacteria;c__Alphaproteobacteria;o__Sphingomonadales;f__Erythrobacteraceae
OTU722	k__Bacteria; p__Armatimonadetes; c__Armatimonadia; o__Armatimonadales; f__Armatimonadaceae; g__; s__	k__Bacteria;p__Armatimonadetes;c__Armatimonadia;o__Armatimonadales;f__uncultured bacterium;g__uncultured bacterium;s__uncultured bacterium
OTU723	k__Bacteria; p__Cyanobacteria; c__Nostocophycideae; o__Nostocales; f__Nostocaceae; g__; s__	k__Bacteria;p__Cyanobacteria;c__Cyanobacteria;o__SubsectionIV;f__FamilyI

OTU724	Unassigned	Unassigned
OTU725	k__Bacteria; p__Firmicutes; c__Bacilli; o__Lactobacillales; f__Lactobacillaceae; g__Lactobacillus; s__	k__Bacteria;p__Firmicutes;c__Bacilli;o__Lactobacillales;f__Lactobacillaceae;g__Lactobacillus;s__uncultured Lactobacillus sp.
OTU726	k__Bacteria; p__Firmicutes; c__Bacilli; o__Lactobacillales; f__Lactobacillaceae; g__Lactobacillus; s__	k__Bacteria;p__Firmicutes;c__Bacilli;o__Lactobacillales;f__Lactobacillaceae;g__Lactobacillus;Ambiguous_taxa
OTU727	Unassigned	Unassigned
OTU728	k__Bacteria; p__Proteobacteria; c__Alphaproteobacteria; o__Sphingomonadales; f__Sphingomonadaceae; g__; s__	k__Bacteria;p__Proteobacteria;c__Alphaproteobacteria;o__Sphingomonadales;f__Sphingomonadaceae;g__Rhizorhapis;Ambiguous_taxa
OTU729	k__Bacteria; p__Bacteroidetes; c__[Saprospirae]; o__[Saprospirales]; f__Chitinophagaceae; g__; s__	k__Bacteria;p__Bacteroidetes;c__Sphingobacteriia;o__Sphingobacteriales;f__Chitinophagaceae;g__Taibaiella
OTU73	k__Bacteria; p__TM7; c__TM7-3; o__EW055; f__; g__; s__	k__Bacteria;p__Saccharibacteria;c__uncultured bacterium;o__uncultured bacterium;f__uncultured bacterium;g__uncultured bacterium;s__uncultured bacterium
OTU730	k__Bacteria; p__Proteobacteria; c__Alphaproteobacteria; o__Rhizobiales; f__Methylobacteriaceae; g__Methylobacterium; s__	k__Bacteria;p__Proteobacteria;c__Alphaproteobacteria;o__Rhizobiales;f__Methylobacteriaceae;g__Methylobacterium;Ambiguous_taxa
OTU731	k__Bacteria; p__Planctomycetes; c__Planctomycetia; o__Pirellulales; f__Pirellulaceae; g__Pirellula; s__	k__Bacteria;p__Planctomycetes;c__Planctomycetacia;o__Planctomycetales;f__Planctomycetaceae;g__Pirellula
OTU732	Unassigned	Unassigned
OTU733	Unassigned	Unassigned
OTU734	Unassigned	Unassigned
OTU735	k__Bacteria; p__Actinobacteria; c__Actinobacteria; o__Actinomycetales; f__Williamsiaceae; g__Williamsia; s__	k__Bacteria;p__Actinobacteria;c__Actinobacteria;o__Corynebacteriales;f__Nocardiaceae;g__Williamsia;Ambiguous_taxa
OTU736	k__Bacteria; p__Actinobacteria; c__Thermoleophilia; o__Solirubrobacterales; f__Solirubrobacteraceae; g__; s__	k__Bacteria;p__Actinobacteria;c__Thermoleophilia;o__Solirubrobacterales;f__Solirubrobacteraceae;g__Solirubrobacter;s__uncultured bacterium
OTU737	k__Bacteria; p__Cyanobacteria; c__Oscillatoriophycideae; o__Oscillatoriales; f__Phormidiaceae; g__Phormidium; s__	k__Bacteria;p__Cyanobacteria;c__Cyanobacteria;o__SubsectionIII;f__FamilyI;g__Phormidium
OTU738	Unassigned	Unassigned
OTU739	k__Bacteria; p__Proteobacteria; c__Alphaproteobacteria; o__Rhizobiales; f__Beijerinckiaceae; g__Beijerinckia; s__	k__Bacteria;p__Proteobacteria;c__Alphaproteobacteria;o__Rhizobiales;f__Beijerinckiaceae
OTU74	Unassigned	Unassigned
OTU740	k__Bacteria; p__Acidobacteria; c__Acidobacteria-6; o__iii1-15; f__; g__; s__	k__Bacteria;p__Acidobacteria;c__Subgroup 6;o__uncultured bacterium;f__uncultured bacterium;g__uncultured bacterium;s__uncultured bacterium
OTU741	k__Bacteria; p__[Thermi]; c__Deinococci; o__Deinococcales; f__Deinococcaceae; g__Deinococcus; s__	k__Bacteria;p__Deinococcus-Thermus;c__Deinococci;o__Deinococcales;f__Deinococcaceae;g__Deinococcus;s__uncultured bacterium
OTU742	k__Bacteria; p__Chloroflexi; c__TK10; o__B07_WMSP1; f__; g__; s__	k__Bacteria;p__Chloroflexi;c__TK10
OTU743	Unassigned	Unassigned
OTU744	k__Bacteria; p__Chlamydiae; c__Chlamydiia; o__Chlamydiales; f__Rhabdochlamydiaceae; g__Candidatus Rhabdochlamydia; s__	k__Bacteria;p__Chlamydiae;c__Chlamydiae;o__Chlamydiales;f__Simkaniaceae;g__Candidatus Rhabdochlamydia
OTU745	Unassigned	Unassigned

OTU746	k__Bacteria; p__Proteobacteria; c__Alphaproteobacteria; o__Rhodospirillales; f__Acetobacteraceae; g__; s__	k__Bacteria;p__Proteobacteria;c__Alphaproteobacteria;o__Rhodospirillales;f__Acetobacteraceae;g__uncultured;s__uncultured bacterium
OTU747	k__Bacteria; p__Firmicutes; c__Bacilli; o__Lactobacillales; f__Lactobacillaceae; g__Lactobacillus; s__	k__Bacteria;p__Firmicutes;c__Bacilli;o__Lactobacillales;f__Lactobacillaceae;g__Lactobacillus;Ambiguous_taxa
OTU748	Unassigned	Unassigned
OTU749	k__Bacteria; p__Bacteroidetes; c__Cytophagia; o__Cytophagales; f__Cytophagaceae; g__Dyadobacter; s__	k__Bacteria;p__Bacteroidetes;c__Cytophagia;o__Cytophagales;f__Cytophagaceae;g__Dyadobacter;s__uncultured bacterium
OTU75	k__Bacteria; p__Cyanobacteria; c__Oscillatoriophycideae; o__Chroococcales; f__Xenococcaceae; g__; s__	k__Bacteria;p__Cyanobacteria;c__Cyanobacteria;o__SubsectionIII;f__FamilyI;g__Crinalium
OTU750	k__Bacteria; p__Proteobacteria; c__Deltaproteobacteria; o__Myxococcales; f__Cystobacterineae; g__; s__	k__Bacteria;p__Proteobacteria;c__Deltaproteobacteria;o__Myxococcales;f__P3OB-42;g__uncultured bacterium;s__uncultured bacterium
OTU751	k__Bacteria; p__Actinobacteria; c__Thermoleophilia; o__Gaiellales; f__; g__; s__	k__Bacteria;p__Actinobacteria;c__Thermoleophilia;o__Gaiellales;f__uncultured;Ambiguous_taxa;Ambiguous_taxa
OTU752	k__Bacteria; p__Proteobacteria; c__Alphaproteobacteria; o__Sphingomonadales; f__Sphingomonadaceae; g__Sphingomonas; s__	k__Bacteria;p__Proteobacteria;c__Alphaproteobacteria;o__Sphingomonadales;f__Sphingomonadaceae;g__Sphingomonas
OTU753	k__Bacteria; p__Firmicutes; c__Bacilli; o__Lactobacillales; f__Lactobacillaceae; g__Lactobacillus; s__	k__Bacteria;p__Firmicutes;c__Bacilli;o__Lactobacillales;f__Lactobacillaceae;g__Lactobacillus
OTU754	k__Bacteria; p__Cyanobacteria; c__Oscillatoriophycideae; o__Chroococcales; f__Xenococcaceae; g__; s__	k__Bacteria;p__Cyanobacteria;c__Cyanobacteria;o__uncultured;f__uncultured bacterium;g__uncultured bacterium;s__uncultured bacterium
OTU755	k__Bacteria; p__Proteobacteria; c__Alphaproteobacteria; o__Rhodospirillales; f__Acetobacteraceae	k__Bacteria;p__Proteobacteria;c__Alphaproteobacteria;o__Rhodospirillales;f__Acetobacteraceae;g__uncultured;s__uncultured Acetobacteraceae bacterium
OTU756	k__Bacteria; p__Proteobacteria; c__Deltaproteobacteria; o__Myxococcales; f__; g__; s__	k__Bacteria;p__Proteobacteria;c__Deltaproteobacteria;o__Myxococcales;f__Haliangiaceae;g__Haliangium;s__uncultured bacterium
OTU757	Unassigned	Unassigned
OTU758	k__Bacteria; p__Proteobacteria; c__Betaproteobacteria; o__Burkholderiales; f__Alcaligenaceae; g__; s__	k__Bacteria;p__Proteobacteria;c__Betaproteobacteria;o__Burkholderiales;f__Alcaligenaceae;g__Alcaligenes
OTU759	k__Bacteria; p__Proteobacteria; c__Alphaproteobacteria; o__Sphingomonadales; f__Sphingomonadaceae	k__Bacteria;p__Proteobacteria;c__Alphaproteobacteria;o__Sphingomonadales
OTU76	k__Bacteria; p__Proteobacteria; c__Alphaproteobacteria; o__Rickettsiales; f__mitochondria	k__Bacteria;p__Proteobacteria;c__Alphaproteobacteria;o__Rickettsiales;f__Mitochondria
OTU760	k__Bacteria; p__Actinobacteria; c__Actinobacteria; o__Actinomycetales; f__Nocardioidaceae; g__Nocardioides; s__plantarum	k__Bacteria;p__Actinobacteria;c__Actinobacteria;o__Propionibacteriales;f__Nocardioidaceae;g__Nocardioides;Ambiguous_taxa
OTU761	Unassigned	Unassigned
OTU762	Unassigned	Unassigned
OTU763	k__Bacteria; p__Proteobacteria; c__Alphaproteobacteria; o__Sphingomonadales; f__Sphingomonadaceae; g__; s__	k__Bacteria;p__Proteobacteria;c__Alphaproteobacteria;o__Sphingomonadales;f__MN 122.2a;g__uncultured bacterium;s__uncultured bacterium
OTU764	Unassigned	Unassigned
OTU765	k__Bacteria; p__Proteobacteria; c__Alphaproteobacteria; o__Sphingomonadales; f__Sphingomonadaceae; g__Sphingobium; s__	k__Bacteria;p__Proteobacteria;c__Alphaproteobacteria;o__Sphingomonadales;f__Sphingomonadaceae;g__Zymomonas
OTU766	k__Bacteria; p__Proteobacteria; c__Alphaproteobacteria; o__Rickettsiales; f__mitochondria	k__Bacteria;p__Proteobacteria;c__Alphaproteobacteria;o__Rickettsiales;f__Mitochondria;Ambiguous_taxa;Ambiguous_taxa
OTU767	Unassigned	Unassigned

OTU768	k__Bacteria; p__Proteobacteria; c__Alphaproteobacteria; o__Rhizobiales; f__Methylobacteriaceae; g__; s__	k__Bacteria;p__Proteobacteria;c__Alphaproteobacteria;o__Rhizobiales;f__Methylobacteriaceae;g__Methylobacterium;s__uncultured bacterium
OTU769	k__Bacteria; p__Proteobacteria; c__Betaproteobacteria; o__Burkholderiales; f__Oxalobacteraceae; g__; s__	k__Bacteria;p__Proteobacteria;c__Betaproteobacteria;o__Burkholderiales;f__Oxalobacteraceae;g__Massilia;Ambiguous_taxa
OTU77	k__Bacteria; p__Cyanobacteria; c__; o__; f__; g__; s__	k__Bacteria;p__Cyanobacteria;c__Cyanobacteria;o__SubsectionIII;f__FamilyI;g__Lyngbya
OTU770	k__Bacteria; p__Proteobacteria; c__Alphaproteobacteria; o__Rhodospirillales; f__Acetobacteraceae	k__Bacteria;p__Proteobacteria;c__Alphaproteobacteria;o__Rhodospirillales;f__Acetobacteraceae
OTU771	k__Bacteria; p__Proteobacteria; c__Alphaproteobacteria; o__Rhizobiales; f__Methylobacteriaceae; g__Methylobacterium; s__komagatae	k__Bacteria;p__Proteobacteria;c__Alphaproteobacteria;o__Rhizobiales;f__Methylobacteriaceae;g__Methylobacterium;Ambiguous_taxa
OTU772	k__Bacteria; p__Proteobacteria; c__Betaproteobacteria; o__Burkholderiales; f__Comamonadaceae	k__Bacteria;p__Proteobacteria;c__Betaproteobacteria;o__Burkholderiales;f__Comamonadaceae
OTU773	k__Bacteria; p__Firmicutes; c__Bacilli; o__Lactobacillales; f__Lactobacillaceae; g__Lactobacillus; s__	k__Bacteria;p__Firmicutes;c__Bacilli;o__Lactobacillales;f__Lactobacillaceae;g__Lactobacillus;s__uncultured Lactobacillaceae bacterium
OTU774	Unassigned	Unassigned
OTU775	Unassigned	Unassigned
OTU776	k__Bacteria; p__Proteobacteria; c__Gammaproteobacteria; o__Pseudomonadales; f__Moraxellaceae; g__Acinetobacter; s__	k__Bacteria;p__Proteobacteria;c__Gammaproteobacteria;o__Pseudomonadales;f__Moraxellaceae;g__Acinetobacter;Ambiguous_taxa
OTU777	Unassigned	Unassigned
OTU778	k__Bacteria; p__Bacteroidetes; c__Flavobacteriia; o__Flavobacteriales; f__Flavobacteriaceae; g__Flavobacterium; s__	k__Bacteria;p__Bacteroidetes;c__Flavobacteriia;o__Flavobacteriales;f__Flavobacteriaceae;g__Flavobacterium
OTU779	k__Bacteria; p__Proteobacteria; c__Alphaproteobacteria; o__Caulobacterales; f__Caulobacteraceae; g__; s__	k__Bacteria;p__Proteobacteria;c__Alphaproteobacteria;o__Caulobacterales;f__Caulobacteraceae;g__uncultured
OTU78	k__Bacteria; p__Bacteroidetes; c__[Saprospirae]; o__[Saprospirales]; f__Chitinophagaceae; g__; s__	k__Bacteria;p__Bacteroidetes;c__Sphingobacteriia;o__Sphingobacteriales;f__Chitinophagaceae;g__uncultured;s__uncultured bacterium
OTU780	k__Bacteria; p__Proteobacteria; c__Betaproteobacteria; o__Burkholderiales; f__Burkholderiaceae; g__Burkholderia; s__andropogonis	k__Bacteria;p__Proteobacteria;c__Betaproteobacteria;o__Burkholderiales;f__Burkholderiaceae;g__Burkholderia-Paraburkholderia;Ambiguous_taxa
OTU781	k__Bacteria; p__Bacteroidetes; c__Sphingobacteriia; o__Sphingobacteriales; f__Sphingobacteriaceae; g__Pedobacter; s__	k__Bacteria;p__Bacteroidetes;c__Sphingobacteriia;o__Sphingobacteriales;f__Sphingobacteriaceae;g__Pedobacter;Ambiguous_taxa
OTU782	k__Bacteria; p__Proteobacteria; c__Alphaproteobacteria; o__Rhizobiales; f__; g__; s__	k__Bacteria;p__Proteobacteria;c__Alphaproteobacteria;o__Rhizobiales;f__Rhizobiaceae;g__Rhizobium
OTU783	k__Bacteria; p__Firmicutes; c__Clostridia; o__Clostridiales; f__Lachnospiraceae; g__; s__	k__Bacteria;p__Firmicutes;c__Clostridia;o__Clostridiales;f__Lachnospiraceae;g__Hungatella
OTU784	k__Bacteria; p__Proteobacteria; c__Alphaproteobacteria; o__Rhizobiales; f__Phyllobacteriaceae	k__Bacteria;p__Proteobacteria;c__Alphaproteobacteria;o__Rhizobiales;f__Phyllobacteriaceae;g__Mesorhizobium;Ambiguous_taxa
OTU785	k__Bacteria; p__Cyanobacteria; c__4C0d-2; o__YS2; f__; g__; s__	k__Bacteria;p__Cyanobacteria;c__Melainabacteria;o__Gastranaerophilales;f__uncultured bacterium;g__uncultured bacterium;s__uncultured bacterium
OTU786	k__Bacteria; p__Actinobacteria; c__Actinobacteria; o__Actinomycetales; f__Nocardioidaceae; g__Aeromicrobium; s__	k__Bacteria;p__Actinobacteria;c__Actinobacteria;o__Propionibacteriales;f__Nocardioidaceae;g__Aeromicrobium;s__uncultured bacterium
OTU787	k__Bacteria; p__Proteobacteria; c__Alphaproteobacteria; o__Rhizobiales; f__Brucellaceae; g__Ochrobactrum; s__	k__Bacteria;p__Proteobacteria;c__Alphaproteobacteria;o__Rhizobiales;f__Brucellaceae
OTU788	Unassigned	Unassigned

OTU789	k__Bacteria; p__Verrucomicrobia; c__[Spartobacteria]; o__[Chthoniobacterales]; f__[Chthoniobacteraceae]; g__Ellin506; s__	k__Bacteria;p__Verrucomicrobia;c__Spartobacteria;o__Chthoniobacterales;f__Chthoniobacteraceae;g__Chthoniobacter;s__uncultured bacterium
OTU79	Unassigned	Unassigned
OTU790	k__Bacteria; p__Cyanobacteria; c__Oscillatoriophycideae; o__Oscillatoriales; f__Phormidiaceae; g__Microcoleus; s__	k__Bacteria;p__Cyanobacteria;c__Cyanobacteria;o__SubsectionIII;f__FamilyI;g__Microcoleus
OTU791	k__Bacteria; p__Actinobacteria; c__Actinobacteria; o__Actinomycetales; f__Nocardioidaceae; g__; s__	k__Bacteria;p__Actinobacteria;c__Actinobacteria;o__Propionibacteriales;f__Nocardioidaceae;g__Marmoricola
OTU792	k__Bacteria; p__Proteobacteria; c__Betaproteobacteria; o__Burkholderiales; f__Oxalobacteraceae; g__; s__	k__Bacteria;p__Proteobacteria;c__Betaproteobacteria;o__Burkholderiales;f__Oxalobacteraceae;g__Massilia
OTU793	k__Bacteria; p__Firmicutes; c__Bacilli; o__Lactobacillales; f__Lactobacillaceae; g__Lactobacillus; s__	k__Bacteria;p__Firmicutes;c__Bacilli;o__Lactobacillales;f__Lactobacillaceae;g__Lactobacillus
OTU794	k__Bacteria; p__Proteobacteria; c__Alphaproteobacteria; o__Caulobacterales; f__Caulobacteraceae; g__; s__	k__Bacteria;p__Proteobacteria;c__Alphaproteobacteria;o__Caulobacterales;f__Caulobacteraceae;g__uncultured;Ambiguous_taxa
OTU795	Unassigned	Unassigned
OTU796	k__Bacteria; p__Proteobacteria; c__Deltaproteobacteria; o__Myxococcales; f__; g__; s__	k__Bacteria;p__Proteobacteria;c__Deltaproteobacteria;o__Myxococcales;f__Archangiaceae
OTU797	k__Bacteria; p__Proteobacteria; c__Alphaproteobacteria; o__Rhodobacterales; f__Rhodobacteraceae; g__Rubellimicrobium; s__	k__Bacteria;p__Proteobacteria;c__Alphaproteobacteria;o__Rhodobacterales;f__Rhodobacteraceae;g__Rubellimicrobium;s__uncultured bacterium
OTU798	k__Bacteria; p__Proteobacteria; c__Alphaproteobacteria; o__BD7-3; f__; g__; s__	k__Bacteria;p__Proteobacteria;c__Alphaproteobacteria;o__Alphaproteobacteria Incertae Sedis;f__uncultured;g__uncultured bacterium;s__uncultured bacterium
OTU799	k__Bacteria; p__Cyanobacteria; c__Synechococcophycideae; o__Pseudanabaenales; f__Pseudanabaenaceae; g__Leptolyngbya; s__	k__Bacteria;p__Cyanobacteria;c__Cyanobacteria;o__SubsectionIII;f__FamilyI;g__Leptolyngbya;s__uncultured bacterium
OTU8	Unassigned	k__Bacteria;p__Cyanobacteria;c__Chloroplast;o__uncultured bacterium;f__uncultured bacterium;g__uncultured bacterium;s__uncultured bacterium
OTU80	k__Bacteria; p__Proteobacteria; c__Gammaproteobacteria; o__Enterobacteriales; f__Enterobacteriaceae; g__; s__	k__Bacteria;p__Proteobacteria;c__Gammaproteobacteria;o__Enterobacteriales;f__Enterobacteriaceae;g__Pantoea
OTU800	k__Bacteria; p__Acidobacteria; c__Acidobacteria-6; o__iii1-15; f__; g__; s__	k__Bacteria;p__Acidobacteria;c__Subgroup 6;Ambiguous_taxa;Ambiguous_taxa;Ambiguous_taxa;Ambiguous_taxa
OTU801	k__Bacteria; p__Bacteroidetes; c__Cytophagia; o__Cytophagales; f__Cytophagaceae; g__Hymenobacter; s__	k__Bacteria;p__Bacteroidetes;c__Cytophagia;o__Cytophagales;f__Cytophagaceae;g__Hymenobacter
OTU802	k__Bacteria; p__Proteobacteria; c__Betaproteobacteria; o__Burkholderiales; f__Comamonadaceae; g__; s__	k__Bacteria;p__Proteobacteria;c__Betaproteobacteria;o__Burkholderiales;f__Comamonadaceae
OTU803	k__Bacteria; p__Actinobacteria; c__Actinobacteria; o__Actinomycetales; f__Corynebacteriaceae; g__Corynebacterium; s__	k__Bacteria;p__Actinobacteria;c__Actinobacteria;o__Corynebacteriales;f__Corynebacteriaceae;g__Corynebacterium 1
OTU804	Unassigned	Unassigned
OTU805	k__Bacteria; p__Firmicutes; c__Bacilli; o__Lactobacillales; f__Streptococcaceae; g__Streptococcus; s__	k__Bacteria;p__Firmicutes;c__Bacilli;o__Lactobacillales;f__Streptococcaceae;g__Streptococcus;s__uncultured organism
OTU806	k__Bacteria; p__Firmicutes; c__Clostridia; o__Clostridiales; f__Veillonellaceae; g__Dialister; s__	k__Bacteria;p__Firmicutes;c__Negativicutes;o__Selenomonadales;f__Veillonellaceae;g__Dialister;s__uncultured bacterium

OTU807	k__Bacteria; p__Proteobacteria; c__Betaproteobacteria; o__Burkholderiales; f__Comamonadaceae	k__Bacteria;p__Proteobacteria;c__Betaproteobacteria;o__Burkholderiales;f__Comamonadaceae
OTU808	k__Bacteria; p__Armatimonadetes; c__Armatimonadia; o__Armatimonadales; f__Armatimonadaceae; g__; s__	k__Bacteria;p__Armatimonadetes;c__Armatimonadia;o__Armatimonadales;f__uncultured bacterium;g__uncultured bacterium;s__uncultured bacterium
OTU809	k__Bacteria; p__Actinobacteria; c__Acidimicrobiia; o__Acidimicrobiales; f__; g__; s__	k__Bacteria;p__Actinobacteria;c__Acidimicrobiia;o__Acidimicrobiales;f__uncultured
OTU81	k__Bacteria; p__Actinobacteria; c__Thermoleophilia; o__Solirubrobacterales; f__; g__; s__	k__Bacteria;p__Actinobacteria;c__Thermoleophilia;o__Solirubrobacterales;f__Elev-16S-1332;g__uncultured bacterium;s__uncultured bacterium
OTU810	k__Bacteria; p__Bacteroidetes; c__Cytophagia; o__Cytophagales; f__Cytophagaceae; g__Spirosoma; s__	k__Bacteria;p__Bacteroidetes;c__Cytophagia;o__Cytophagales;f__Cytophagaceae;g__Spirosoma;s__uncultured bacterium
OTU811	k__Bacteria; p__Proteobacteria; c__Betaproteobacteria; o__Burkholderiales; f__Oxalobacteraceae; g__Janthinobacterium; s__	k__Bacteria;p__Proteobacteria;c__Betaproteobacteria;o__Burkholderiales;f__Oxalobacteraceae;g__Duganella
OTU812	Unassigned	Unassigned
OTU813	k__Bacteria; p__Acidobacteria; c__Acidobacteriia; o__Acidobacteriales; f__Acidobacteriaceae; g__Edaphobacter; s__modestum	k__Bacteria;p__Acidobacteria;c__Acidobacteria;o__Acidobacteriales;f__Acidobacteriaceae (Subgroup 1);g__Granulicella;s__uncultured bacterium
OTU814	k__Bacteria; p__Bacteroidetes; c__Cytophagia; o__Cytophagales; f__Cytophagaceae; g__; s__	k__Bacteria;p__Bacteroidetes;c__Cytophagia;o__Cytophagales;f__Cytophagaceae;g__Larkinella;s__uncultured bacterium
OTU815	k__Bacteria; p__Firmicutes; c__Clostridia; o__Clostridiales; f__Ruminococcaceae; g__Oscillospira; s__	k__Bacteria;p__Firmicutes;c__Clostridia;o__Clostridiales;f__Ruminococcaceae;g__Flavonifractor;s__uncultured bacterium
OTU816	Unassigned	Unassigned
OTU817	k__Bacteria; p__Bacteroidetes; c__[Saprospirae]; o__[Saprospirales]; f__Chitinophagaceae; g__; s__	k__Bacteria;p__Bacteroidetes;c__Sphingobacteriia;o__Sphingobacteriales;f__Chitinophagaceae;g__Ferruginibacter;s__uncultured bacterium
OTU818	Unassigned	Unassigned
OTU819	k__Bacteria; p__Proteobacteria; c__Gammaproteobacteria; o__Legionellales; f__Coxiellaceae; g__; s__	k__Bacteria;p__Proteobacteria;c__Gammaproteobacteria;o__Legionellales;f__Coxiellaceae;g__uncultured
OTU82	k__Bacteria; p__Proteobacteria; c__Alphaproteobacteria; o__Sphingomonadales; f__Sphingomonadaceae; g__Kaistobacter; s__	k__Bacteria;p__Proteobacteria;c__Alphaproteobacteria;o__Sphingomonadales;f__Sphingomonadaceae;g__Sphingomonas;Ambiguous_taxa
OTU820	k__Bacteria; p__Proteobacteria; c__Betaproteobacteria; o__Burkholderiales; f__Oxalobacteraceae; g__; s__	k__Bacteria;p__Proteobacteria;c__Betaproteobacteria;o__Burkholderiales;f__Oxalobacteraceae;g__Massilia;Ambiguous_taxa
OTU821	k__Bacteria; p__Bacteroidetes; c__[Saprospirae]; o__[Saprospirales]; f__Chitinophagaceae; g__; s__	k__Bacteria;p__Bacteroidetes;c__Sphingobacteriia;o__Sphingobacteriales;f__Chitinophagaceae;g__Cnuella;Ambiguous_taxa
OTU822	Unassigned	Unassigned
OTU823	k__Bacteria; p__Acidobacteria; c__Acidobacteria-6; o__iii1-15; f__; g__; s__	k__Bacteria;p__Acidobacteria;c__Subgroup 6
OTU824	k__Bacteria; p__Verrucomicrobia; c__[Spartobacteria]; o__[Chthoniobacterales]; f__[Chthoniobacteraceae]; g__; s__	k__Bacteria;p__Verrucomicrobia;c__Spartobacteria;o__Chthoniobacterales;f__LD29;g__uncultured bacterium;s__uncultured bacterium
OTU825	k__Bacteria; p__Proteobacteria; c__Gammaproteobacteria; o__Enterobacteriales; f__Enterobacteriaceae; g__Klebsiella; s__	k__Bacteria;p__Proteobacteria;c__Gammaproteobacteria;o__Enterobacteriales;f__Enterobacteriaceae;g__Klebsiella;Ambiguous_taxa
OTU826	k__Bacteria; p__Tenericutes; c__Mollicutes; o__Entomoplasmatales; f__Entomoplasmataceae	k__Bacteria;p__Tenericutes;c__Mollicutes;o__Entomoplasmatales;f__Entomoplasmataceae;g__Mesoplasma
OTU827	Unassigned	k__Bacteria;p__Chloroflexi;c__Chloroflexia;o__Kallotenuales;f__AKIW781;g__uncultured bacterium;s__uncultured bacterium

OTU828	k__Bacteria; p__[Thermi]; c__Deinococci; o__Deinococcales; f__Deinococcaceae; g__Deinococcus; s__	k__Bacteria;p__Deinococcus-Thermus;c__Deinococci;o__Deinococcales;f__Deinococcaceae;g__Deinococcus;s__uncultured bacterium
OTU829	k__Bacteria; p__Proteobacteria; c__Betaproteobacteria; o__Burkholderiales; f__Comamonadaceae	k__Bacteria;p__Proteobacteria;c__Betaproteobacteria;o__Burkholderiales;f__Comamonadaceae;g__Ramlibacter;s__uncultured bacterium
OTU83	k__Bacteria; p__Firmicutes; c__Bacilli; o__Lactobacillales; f__Lactobacillaceae; g__Lactobacillus; s__	k__Bacteria;p__Firmicutes;c__Bacilli;o__Lactobacillales;f__Lactobacillaceae;g__Lactobacillus
OTU830	k__Bacteria; p__Cyanobacteria; c__Oscillatoriophycideae; o__Chroococcales; f__Xenococcaceae; g__; s__	k__Bacteria;p__Cyanobacteria;c__Cyanobacteria;o__SubsectionII;f__FamilyII;g__Chroococcidiopsis;Ambiguous_taxa
OTU831	k__Bacteria; p__Actinobacteria; c__Acidimicrobiia; o__Acidimicrobiales; f__; g__; s__	k__Bacteria;p__Actinobacteria;c__Acidimicrobiia;o__Acidimicrobiales;f__uncultured;g__uncultured bacterium;s__uncultured bacterium
OTU832	k__Bacteria; p__Firmicutes; c__Bacilli; o__Lactobacillales; f__Lactobacillaceae; g__Lactobacillus; s__	k__Bacteria;p__Firmicutes;c__Bacilli;o__Lactobacillales;f__Lactobacillaceae;g__Lactobacillus;Ambiguous_taxa
OTU833	k__Bacteria; p__Actinobacteria; c__Actinobacteria; o__Actinomycetales; f__Frankiaceae; g__; s__	k__Bacteria;p__Actinobacteria;c__Actinobacteria;o__Frankiales;f__Frankiaceae;g__Jatrophihabitans;s__uncultured bacterium
OTU834	k__Bacteria; p__Proteobacteria; c__Alphaproteobacteria; o__Rhizobiales; f__Rhizobiaceae; g__Agrobacterium; s__	k__Bacteria;p__Proteobacteria;c__Alphaproteobacteria;o__Rhizobiales;f__Rhizobiaceae;g__Ensifer
OTU835	k__Bacteria; p__Actinobacteria; c__Actinobacteria; o__Actinomycetales; f__Microbacteriaceae; g__Agromyces; s__	k__Bacteria;p__Actinobacteria;c__Actinobacteria;o__Micrococcales;f__Microbacteriaceae;g__Agromyces;Ambiguous_taxa
OTU836	Unassigned	Unassigned
OTU837	Unassigned	Unassigned
OTU838	k__Bacteria; p__Proteobacteria; c__Alphaproteobacteria; o__Sphingomonadales; f__Sphingomonadaceae; g__; s__	k__Bacteria;p__Proteobacteria;c__Alphaproteobacteria;o__Sphingomonadales;f__Sphingomonadaceae;g__Sphingomonas;Ambiguous_taxa
OTU839	k__Bacteria; p__Firmicutes; c__Bacilli; o__Lactobacillales; f__Lactobacillaceae; g__Lactobacillus; s__brevis	k__Bacteria;p__Firmicutes;c__Bacilli;o__Lactobacillales;f__Lactobacillaceae;g__Lactobacillus;Ambiguous_taxa
OTU84	k__Bacteria; p__Firmicutes; c__Bacilli; o__Bacillales; f__Staphylococcaceae; g__Staphylococcus; s__	k__Bacteria;p__Firmicutes;c__Bacilli;o__Bacillales;f__Staphylococcaceae;g__Staphylococcus;s__uncultured bacterium
OTU840	k__Bacteria; p__Actinobacteria; c__Actinobacteria; o__Actinomycetales; f__Micrococcaceae; g__; s__	k__Bacteria;p__Actinobacteria;c__Actinobacteria;o__Micrococcales;f__Micrococcaceae;g__Paenarthrobacter
OTU841	k__Bacteria; p__Proteobacteria; c__Alphaproteobacteria; o__Rhodospirillales; f__Acetobacteraceae	k__Bacteria;p__Proteobacteria;c__Alphaproteobacteria;o__Rhodospirillales;f__Acetobacteraceae;g__uncultured;s__uncultured bacterium
OTU842	k__Bacteria; p__Actinobacteria; c__Actinobacteria; o__Actinomycetales; f__Pseudonocardiaceae; g__; s__	k__Bacteria;p__Actinobacteria;c__Actinobacteria;o__Propionibacteriales;f__Propionibacteriaceae;g__uncultured
OTU843	k__Bacteria; p__Firmicutes; c__Clostridia; o__Clostridiales; f__Lachnospiraceae; g__Dorea; s__	k__Bacteria;p__Firmicutes;c__Clostridia;o__Clostridiales;f__Lachnospiraceae;g__[Eubacterium] fissicatena group;Ambiguous_taxa
OTU844	k__Bacteria; p__Proteobacteria; c__Alphaproteobacteria; o__Rhodospirillales; f__Acetobacteraceae; g__; s__	k__Bacteria;p__Proteobacteria;c__Alphaproteobacteria;o__Rhodospirillales;f__Acetobacteraceae;g__uncultured;s__uncultured Acetobacteraceae bacterium
OTU845	k__Bacteria; p__Proteobacteria; c__Alphaproteobacteria; o__Rhodospirillales; f__Acetobacteraceae; g__; s__	k__Bacteria;p__Proteobacteria;c__Alphaproteobacteria;o__Rhodospirillales;f__Acetobacteraceae;g__uncultured;s__uncultured Acetobacteraceae bacterium
OTU846	k__Bacteria; p__Bacteroidetes; c__[Saprospirae]; o__[Saprospirales]; f__Chitinophagaceae; g__; s__	k__Bacteria;p__Bacteroidetes;c__Sphingobacteriia;o__Sphingobacteriales;f__Chitinophagaceae;g__uncultured;s__uncultured bacterium

OTU847	k__Bacteria; p__Bacteroidetes; c__[Saprospirae]; o__[Saprospirales]; f__Chitinophagaceae; g__; s__	k__Bacteria;p__Bacteroidetes;c__Sphingobacteriia;o__Sphingobacteriales;f__Chitinophagaceae;g__Taibaiella;s__uncultured bacterium
OTU848	k__Bacteria; p__MVP-21; c__; o__; f__; g__; s__	k__Bacteria;p__BJ-169;c__uncultured bacterium;o__uncultured bacterium;f__uncultured bacterium;g__uncultured bacterium;s__uncultured bacterium
OTU849	k__Bacteria; p__Armatimonadetes; c__Armatimonadia; o__Armatimonadales; f__Armatimonadaceae; g__; s__	k__Bacteria;p__Armatimonadetes;c__Armatimonadia;o__Armatimonadales;f__uncultured bacterium;g__uncultured bacterium;s__uncultured bacterium
OTU85	k__Bacteria; p__TM7; c__TM7-1; o__; f__; g__; s__	k__Bacteria;p__Saccharibacteria;c__uncultured bacterium;o__uncultured bacterium;f__uncultured bacterium;g__uncultured bacterium;s__uncultured bacterium
OTU850	k__Bacteria; p__Bacteroidetes; c__[Saprospirae]; o__[Saprospirales]; f__Chitinophagaceae; g__Chitinophaga; s__	k__Bacteria;p__Bacteroidetes;c__Sphingobacteriia;o__Sphingobacteriales;f__Chitinophagaceae;g__Chitinophaga
OTU851	Unassigned	Unassigned
OTU852	k__Bacteria; p__Bacteroidetes; c__Cytophagia; o__Cytophagales; f__Cytophagaceae; g__Hymenobacter; s__	k__Bacteria;p__Bacteroidetes;c__Cytophagia;o__Cytophagales;f__Cytophagaceae;g__Hymenobacter;Ambiguous_taxa
OTU853	Unassigned	k__Bacteria;p__Proteobacteria;c__Alphaproteobacteria;o__Rhodospirillales;f__Acetobacteraceae;g__uncultured;s__uncultured bacterium
OTU854	k__Bacteria; p__Firmicutes; c__Clostridia; o__Clostridiales; f__Lachnospiraceae; g__; s__	k__Bacteria;p__Firmicutes;c__Clostridia;o__Clostridiales;f__Lachnospiraceae;g__Tyzzerella 3;s__uncultured Eubacteriaceae bacterium
OTU855	k__Bacteria; p__Verrucomicrobia; c__[Spartobacteria]; o__[Chthoniobacterales]; f__[Chthoniobacteraceae]; g__Candidatus Xiphinematobacter; s__	k__Bacteria;p__Verrucomicrobia;c__Spartobacteria;o__Chthoniobacterales;f__Xiphinematobacteraceae;g__Candidatus Xiphinematobacter;s__uncultured bacterium
OTU856	k__Bacteria; p__Firmicutes; c__Bacilli; o__Bacillales; f__Planococcaceae; g__Lysinibacillus; s__boronitolerans	k__Bacteria;p__Firmicutes;c__Bacilli;o__Bacillales;f__Planococcaceae;g__Lysinibacillus
OTU857	k__Bacteria; p__Proteobacteria; c__Gammaproteobacteria; o__Vibrionales; f__Pseudoalteromonadaceae	k__Bacteria;p__Proteobacteria;c__Gammaproteobacteria;o__Vibrionales;f__Vibrionaceae;g__Vibrio
OTU858	k__Bacteria; p__Firmicutes; c__Bacilli; o__Lactobacillales; f__Carnobacteriaceae; g__Granulicatella; s__	k__Bacteria;p__Firmicutes;c__Bacilli;o__Lactobacillales;f__Carnobacteriaceae;g__Granulicatella;s__uncultured bacterium
OTU859	k__Bacteria; p__Cyanobacteria; c__Synechococcophycideae; o__Pseudanabaenales; f__Pseudanabaenaceae; g__Leptolyngbya; s__	k__Bacteria;p__Cyanobacteria;c__Cyanobacteria;o__SubsectionIII;f__FamilyI;g__Leptolyngbya
OTU86	k__Bacteria; p__Bacteroidetes; c__Cytophagia; o__Cytophagales; f__Cytophagaceae; g__Rudanella; s__	k__Bacteria;p__Bacteroidetes;c__Cytophagia;o__Cytophagales;f__Cytophagaceae;g__Rudanella
OTU860	k__Bacteria; p__TM7; c__TM7-3; o__; f__; g__; s__	k__Bacteria;p__Saccharibacteria;c__uncultured bacterium;o__uncultured bacterium;f__uncultured bacterium;g__uncultured bacterium;s__uncultured bacterium
OTU861	k__Bacteria; p__Actinobacteria; c__Actinobacteria; o__Actinomycetales; f__Microbacteriaceae; g__Curtobacterium; s__	k__Bacteria;p__Actinobacteria;c__Actinobacteria;o__Micrococcales;f__Microbacteriaceae
OTU862	k__Bacteria; p__Cyanobacteria; c__Nostocophycideae; o__Stigonematales; f__Rivulariaceae; g__Calothrix; s__	k__Bacteria;p__Cyanobacteria;c__Cyanobacteria;o__SubsectionIV;f__FamilyI;g__Calothrix
OTU863	k__Bacteria; p__[Thermi]; c__Deinococci; o__Deinococcales; f__Deinococcaceae; g__Deinococcus; s__	k__Bacteria;p__Deinococcus-Thermus;c__Deinococci;o__Deinococcales;f__Deinococcaceae;g__Deinococcus;s__uncultured bacterium

OTU864	k__Bacteria; p__Cyanobacteria; c__Chloroplast; o__Chlorophyta; f__; g__; s__	k__Bacteria;p__Cyanobacteria;c__Chloroplast
OTU865	Unassigned	Unassigned
OTU866	k__Bacteria; p__Actinobacteria; c__Acidimicrobiia; o__Acidimicrobiales; f__Iamiaceae; g__Iamia; s__	k__Bacteria;p__Actinobacteria;c__Acidimicrobiia;o__Acidimicrobiales;f__Iamiaceae;g__Iamia
OTU867	k__Bacteria; p__Acidobacteria; c__[Chloracidobacteria]; o__11-24; f__; g__; s__	k__Bacteria;p__Acidobacteria;c__Blastocatellia;o__Blastocatellales;f__Blastocatellaceae (Subgroup 4);g__11-24;Ambiguous_taxa
OTU868	Unassigned	Unassigned
OTU869	k__Bacteria; p__Actinobacteria; c__Actinobacteria; o__Actinomycetales; f__Micrococcaceae; g__Rothia; s__mucilaginosa	k__Bacteria;p__Actinobacteria;c__Actinobacteria;o__Micrococcales;f__Micrococcaceae;g__Rothia;s__uncultured bacterium
OTU87	k__Bacteria; p__Cyanobacteria; c__Synechococcophycideae; o__Pseudanabaenales; f__Pseudanabaenaceae; g__Leptolyngbya; s__	k__Bacteria;p__Cyanobacteria;c__Cyanobacteria;o__SubsectionIII;f__FamilyI;g__Leptolyngbya;s__uncultured bacterium
OTU870	k__Bacteria; p__Actinobacteria; c__Actinobacteria; o__Actinomycetales; f__Micromonosporaceae; g__Pilimelia; s__	k__Bacteria;p__Actinobacteria;c__Actinobacteria;o__Micromonosporales;f__Micromonosporaceae;g__Luedemannella;Ambiguous_taxa
OTU871	k__Bacteria; p__Proteobacteria; c__Alphaproteobacteria; o__Rhizobiales; f__Hyphomicrobiaceae; g__Devosia; s__	k__Bacteria;p__Proteobacteria;c__Alphaproteobacteria;o__Rhizobiales;f__Hyphomicrobiaceae;g__Devosia
OTU872	k__Bacteria; p__Planctomycetes; c__Planctomycetia; o__Gemmatales; f__Gemmataceae; g__; s__	k__Bacteria;p__Planctomycetes;c__Planctomycetacia;o__Planctomycetales;f__Planctomycetaceae;g__uncultured;s__uncultured bacterium
OTU873	k__Bacteria; p__Bacteroidetes; c__[Rhodothermi]; o__[Rhodothermales]; f__Rhodothermaceae; g__Rubricoccus; s__	k__Bacteria;p__Bacteroidetes;c__Bacteroidetes Incertae Sedis;o__Order II;f__Rhodothermaceae;g__Rubrivirga
OTU874	k__Bacteria; p__Actinobacteria; c__Actinobacteria; o__Actinomycetales; f__; g__; s__	k__Bacteria;p__Actinobacteria;c__Actinobacteria;o__Frankiales;f__Sporichthyaceae;g__uncultured
OTU875	k__Bacteria; p__Firmicutes; c__Clostridia; o__Clostridiales; f__Clostridiaceae; g__Clostridium; s__	k__Bacteria;p__Firmicutes;c__Clostridia;o__Clostridiales;f__Clostridiaceae 1;g__Clostridium sensu stricto 10
OTU876	k__Bacteria; p__Cyanobacteria; c__Synechococcophycideae; o__Pseudanabaenales; f__Pseudanabaenaceae; g__Leptolyngbya; s__	k__Bacteria;p__Cyanobacteria;c__Cyanobacteria;o__SubsectionIII;f__FamilyI;g__Leptolyngbya;s__uncultured bacterium
OTU877	k__Bacteria; p__Proteobacteria; c__Alphaproteobacteria; o__Rhodospirillales; f__Rhodospirillaceae; g__; s__	k__Bacteria;p__Proteobacteria;c__Alphaproteobacteria;o__Rhodospirillales;f__DA111;g__uncultured bacterium;s__uncultured bacterium
OTU878	Unassigned	Unassigned
OTU879	k__Bacteria; p__Acidobacteria; c__Acidobacteria-6; o__iii1-15; f__; g__; s__	k__Bacteria;p__Acidobacteria;c__Subgroup 6
OTU88	k__Bacteria; p__Proteobacteria; c__Betaproteobacteria; o__Burkholderiales; f__Comamonadaceae; g__Methylibium; s__	k__Bacteria;p__Proteobacteria;c__Betaproteobacteria;o__Burkholderiales;f__Comamonadaceae;g__uncultured
OTU880	k__Bacteria; p__TM7; c__SC3; o__; f__; g__; s__	k__Bacteria;p__Saccharibacteria;c__uncultured bacterium;o__uncultured bacterium;f__uncultured bacterium;g__uncultured bacterium;s__uncultured bacterium
OTU881	k__Bacteria; p__Proteobacteria; c__Gammaproteobacteria; o__Pseudomonadales; f__Moraxellaceae; g__Acinetobacter; s__	k__Bacteria;p__Proteobacteria;c__Gammaproteobacteria;o__Pseudomonadales;f__Moraxellaceae;g__Acinetobacter;Ambiguous_taxa
OTU882	Unassigned	Unassigned
OTU883	Unassigned	Unassigned
OTU884	k__Bacteria; p__Proteobacteria; c__Gammaproteobacteria; o__Xanthomonadales; f__Xanthomonadaceae; g__; s__	k__Bacteria;p__Proteobacteria;c__Gammaproteobacteria;o__Xanthomonadales;f__Xanthomonadaceae
OTU885	Unassigned	Unassigned

OTU886	k__Bacteria; p__Proteobacteria; c__Gammaproteobacteria; o__Pseudomonadales; f__Moraxellaceae; g__; s__	k__Bacteria;p__Proteobacteria;c__Gammaproteobacteria;o__Pseudomonadales;f__Moraxellaceae;g__Alkanindiges;Ambiguous_taxa
OTU887	k__Bacteria; p__Firmicutes; c__Bacilli; o__Lactobacillales; f__Lactobacillaceae; g__Lactobacillus; s__	k__Bacteria;p__Firmicutes;c__Bacilli;o__Lactobacillales;f__Lactobacillaceae;g__Lactobacillus;s__uncultured Lactobacillus sp.
OTU888	k__Bacteria; p__Actinobacteria; c__Actinobacteria; o__Actinomycetales; f__Nocardioidaceae; g__; s__	k__Bacteria;p__Actinobacteria;c__Actinobacteria;o__Propionibacteriales;f__Nocardioidaceae;g__Nocardioides;s__uncultured bacterium
OTU889	k__Bacteria; p__Proteobacteria; c__Alphaproteobacteria; o__Rhodospirillales; f__Rhodospirillaceae; g__Azospirillum; s__	k__Bacteria;p__Proteobacteria;c__Alphaproteobacteria;o__Rhodospirillales;f__Rhodospirillaceae;g__Azospirillum
OTU89	Unassigned	Unassigned
OTU890	k__Bacteria; p__Actinobacteria; c__Actinobacteria; o__Actinomycetales; f__Pseudonocardiaceae; g__Actinomycetospora; s__	k__Bacteria;p__Actinobacteria;c__Actinobacteria;o__Pseudonocardiales;f__Pseudonocardiaceae;g__Actinomycetospora;Ambiguous_taxa
OTU891	k__Bacteria; p__[Thermi]; c__Deinococci; o__Deinococcales; f__Trueperaceae; g__Truepera; s__	k__Bacteria;p__Deinococcus-Thermus;c__Deinococci;o__Deinococcales;f__Trueperaceae;g__Truepera
OTU892	k__Bacteria; p__Bacteroidetes; c__[Saprospirae]; o__[Saprospirales]; f__Chitinophagaceae; g__; s__	k__Bacteria;p__Bacteroidetes;c__Sphingobacteriia;o__Sphingobacteriales;f__Chitinophagaceae;g__uncultured;s__uncultured bacterium
OTU893	k__Bacteria; p__Actinobacteria; c__Actinobacteria; o__Actinomycetales; f__Corynebacteriaceae; g__Corynebacterium; s__	k__Bacteria;p__Actinobacteria;c__Actinobacteria;o__Corynebacteriales;f__Corynebacteriaceae;g__Corynebacterium 1;s__uncultured bacterium
OTU894	k__Bacteria; p__Proteobacteria; c__Alphaproteobacteria; o__Rhodobacterales; f__Rhodobacteraceae; g__Rubellimicrobium; s__	k__Bacteria;p__Proteobacteria;c__Alphaproteobacteria;o__Rhodobacterales;f__Rhodobacteraceae;g__Rubellimicrobium;s__uncultured bacterium
OTU895	k__Bacteria; p__Proteobacteria; c__Alphaproteobacteria; o__Rhizobiales; f__Beijerinckiaceae; g__; s__	k__Bacteria;p__Proteobacteria;c__Alphaproteobacteria;o__Rhizobiales;f__Methylobacteriaceae
OTU896	k__Bacteria; p__Proteobacteria; c__Gammaproteobacteria; o__Pseudomonadales; f__Moraxellaceae; g__Acinetobacter; s__	k__Bacteria;p__Proteobacteria;c__Gammaproteobacteria;o__Pseudomonadales;f__Moraxellaceae;g__Acinetobacter;Ambiguous_taxa
OTU897	k__Bacteria; p__Cyanobacteria; c__Oscillatoriophycideae; o__Oscillatoriales; f__Phormidiaceae; g__Phormidium; s__	k__Bacteria;p__Cyanobacteria;c__Cyanobacteria;o__SubsectionIII;f__FamilyI;g__uncultured
OTU898	Unassigned	Unassigned
OTU899	k__Bacteria; p__Bacteroidetes; c__Bacteroidia; o__Bacteroidales; f__Porphyromonadaceae; g__Dysgonomonas; s__	k__Bacteria;p__Bacteroidetes;c__Bacteroidia;o__Bacteroidales;f__Porphyromonadaceae;g__Dysgonomonas;s__uncultured bacterium
OTU9	k__Bacteria; p__Acidobacteria; c__Acidobacteria-6; o__iii1-15; f__; g__; s__	k__Bacteria;p__Acidobacteria;c__Subgroup 6;Ambiguous_taxa;Ambiguous_taxa;Ambiguous_taxa;Ambiguous_taxa
OTU90	k__Bacteria; p__Actinobacteria; c__Actinobacteria; o__Actinomycetales; f__Mycobacteriaceae; g__Mycobacterium; s__celatum	k__Bacteria;p__Actinobacteria;c__Actinobacteria;o__Corynebacteriales;f__Mycobacteriaceae;g__Mycobacterium
OTU900	k__Bacteria; p__Cyanobacteria; c__Chloroplast; o__Chlorophyta; f__; g__; s__	k__Bacteria;p__Cyanobacteria;c__Chloroplast
OTU901	Unassigned	Unassigned
OTU902	k__Bacteria; p__Proteobacteria; c__Betaproteobacteria; o__Burkholderiales; f__Oxalobacteraceae; g__; s__	k__Bacteria;p__Proteobacteria;c__Betaproteobacteria;o__Burkholderiales;f__Oxalobacteraceae;g__Massilia;Ambiguous_taxa
OTU903	k__Bacteria; p__Actinobacteria; c__Actinobacteria; o__Actinomycetales; f__Kineosporiaceae; g__Kineococcus; s__	k__Bacteria;p__Actinobacteria;c__Actinobacteria;o__Kineosporiales;f__Kineosporiaceae;g__Kineococcus;s__Kineococcus aurantiacus
OTU904	k__Bacteria; p__Proteobacteria; c__Betaproteobacteria; o__SC-I-84; f__; g__; s__	k__Bacteria;p__Proteobacteria;c__Betaproteobacteria;o__SC-I-84
OTU905	Unassigned	Unassigned

OTU906	k__Bacteria; p__Proteobacteria; c__Gammaproteobacteria; o__Pseudomonadales; f__Pseudomonadaceae; g__; s__	k__Bacteria;p__Proteobacteria;c__Gammaproteobacteria;o__Pseudomonadales;f__Pseudomonadaceae;g__Pseudomonas;s__uncultured bacterium
OTU907	k__Bacteria; p__Chloroflexi; c__C0119; o__; f__; g__; s__	k__Bacteria;p__Chloroflexi;c__Ktedonobacteria;o__C0119;f__uncultured bacterium;g__uncultured bacterium;s__uncultured bacterium
OTU908	Unassigned	k__Bacteria;p__Actinobacteria;c__Actinobacteria;o__Corynebacteriales;f__nbr16a11;g__uncultured bacterium;s__uncultured bacterium
OTU909	Unassigned	Unassigned
OTU91	k__Bacteria; p__Cyanobacteria; c__Chloroplast; o__Stramenopiles; f__; g__; s__	k__Bacteria;p__Cyanobacteria;c__Chloroplast;o__uncultured bacterium;f__uncultured bacterium;g__uncultured bacterium;s__uncultured bacterium
OTU910	k__Bacteria; p__Proteobacteria; c__Alphaproteobacteria; o__Rhizobiales	k__Bacteria;p__Proteobacteria;c__Alphaproteobacteria;o__Rhizobiales;f__D05-2;g__uncultured bacterium;s__uncultured bacterium
OTU911	k__Bacteria; p__Bacteroidetes; c__[Saprospirae]; o__[Saprospirales]; f__Chitinophagaceae; g__; s__	k__Bacteria;p__Bacteroidetes;c__Sphingobacteriia;o__Sphingobacteriales;f__Chitinophagaceae;g__uncultured;s__uncultured bacterium
OTU912	Unassigned	Unassigned
OTU913	k__Bacteria; p__Cyanobacteria; c__4C0d-2; o__SM1D11; f__; g__; s__	k__Bacteria;p__Cyanobacteria;c__Melainabacteria;o__Vampirovibrionales;f__uncultured bacterium;g__uncultured bacterium;s__uncultured bacterium
OTU914	k__Bacteria; p__Proteobacteria; c__Alphaproteobacteria; o__Sphingomonadales; f__Sphingomonadaceae; g__Sphingomonas; s__wittichii	k__Bacteria;p__Proteobacteria;c__Alphaproteobacteria;o__Sphingomonadales;f__Sphingomonadaceae;g__Sphingomonas
OTU915	k__Bacteria; p__TM7; c__TM7-3; o__I025; f__; g__; s__	k__Bacteria;p__Saccharibacteria;c__uncultured bacterium;o__uncultured bacterium;f__uncultured bacterium;g__uncultured bacterium;s__uncultured bacterium
OTU916	k__Bacteria; p__Cyanobacteria; c__Chloroplast; o__Streptophyta; f__; g__; s__	k__Bacteria;p__Cyanobacteria;c__Chloroplast;o__uncultured bacterium;f__uncultured bacterium;g__uncultured bacterium;s__uncultured bacterium
OTU917	k__Bacteria; p__Proteobacteria; c__Alphaproteobacteria; o__Sphingomonadales; f__Sphingomonadaceae; g__; s__	k__Bacteria;p__Proteobacteria;c__Alphaproteobacteria;o__Sphingomonadales
OTU918	k__Bacteria; p__Actinobacteria; c__Actinobacteria; o__Actinomycetales; f__Micrococcaceae; g__Micrococcus	k__Bacteria;p__Actinobacteria;c__Actinobacteria;o__Micrococcales;f__Micrococcaceae;g__Micrococcus;Ambiguous_taxa
OTU919	k__Bacteria; p__Planctomycetes; c__Phycisphaerae; o__WD2101; f__; g__; s__	k__Bacteria;p__Planctomycetes;c__Phycisphaerae;o__Tepidisphaerales;f__Tepidisphaeraceae;g__Tepidisphaera;s__uncultured bacterium
OTU92	k__Bacteria; p__Proteobacteria; c__Betaproteobacteria; o__Burkholderiales; f__Comamonadaceae; g__Ramlibacter; s__	k__Bacteria;p__Proteobacteria;c__Betaproteobacteria;o__Burkholderiales;f__Comamonadaceae;g__Caenimonas
OTU920	k__Bacteria; p__Actinobacteria; c__Actinobacteria; o__Actinomycetales; f__Pseudonocardiaceae; g__Actinomycetospora; s__	k__Bacteria;p__Actinobacteria;c__Actinobacteria;o__Pseudonocardiales;f__Pseudonocardiaceae;g__Actinomycetospora;Ambiguous_taxa
OTU921	k__Bacteria; p__Bacteroidetes; c__Flavobacteriia; o__Flavobacteriales; f__Flavobacteriaceae; g__Flavobacterium; s__	k__Bacteria;p__Bacteroidetes;c__Flavobacteriia;o__Flavobacteriales;f__Flavobacteriaceae;g__Flavobacterium
OTU922	k__Bacteria; p__Proteobacteria; c__Alphaproteobacteria; o__Rhizobiales; f__Methylocystaceae; g__; s__	k__Bacteria;p__Proteobacteria;c__Alphaproteobacteria;o__Rhizobiales;f__1174-901-12;g__uncultured bacterium;s__uncultured bacterium
OTU923	Unassigned	Unassigned

OTU924	k__Bacteria; p__TM7; c__TM7-1; o__; f__; g__; s__	k__Bacteria;p__Saccharibacteria;c__uncultured bacterium;o__uncultured bacterium;f__uncultured bacterium;g__uncultured bacterium;s__uncultured bacterium
OTU925	k__Bacteria; p__Bacteroidetes; c__Cytophagia; o__Cytophagales; f__Cytophagaceae; g__Hymenobacter; s__	k__Bacteria;p__Bacteroidetes;c__Cytophagia;o__Cytophagales;f__Cytophagaceae;g__Hymenobacter;s__uncultured bacterium
OTU926	k__Bacteria; p__Proteobacteria; c__Betaproteobacteria; o__Burkholderiales; f__Alcaligenaceae; g__Sutterella; s__	k__Bacteria;p__Proteobacteria;c__Betaproteobacteria;o__Burkholderiales;f__Alcaligenaceae;g__Sutterella;s__uncultured bacterium
OTU927	k__Bacteria; p__Proteobacteria; c__Alphaproteobacteria; o__Sphingomonadales; f__Sphingomonadaceae; g__; s__	k__Bacteria;p__Proteobacteria;c__Alphaproteobacteria;o__Sphingomonadales;f__Sphingomonadaceae;g__Zymomonas;Ambiguous_taxa
OTU928	Unassigned	Unassigned
OTU929	k__Bacteria; p__Proteobacteria; c__Gammaproteobacteria; o__Xanthomonadales; f__Xanthomonadaceae; g__Lysobacter; s__	k__Bacteria;p__Proteobacteria;c__Gammaproteobacteria;o__Xanthomonadales;f__Xanthomonadaceae;g__Lysobacter;s__uncultured bacterium
OTU93	k__Bacteria; p__Actinobacteria; c__Actinobacteria; o__Actinomycetales; f__Geodermatophilaceae; g__Geodermatophilus; s__	k__Bacteria;p__Actinobacteria;c__Actinobacteria;o__Frankiales;f__Geodermatophilaceae;g__Geodermatophilus
OTU930	k__Bacteria; p__Proteobacteria; c__Gammaproteobacteria; o__Pseudomonadales; f__Pseudomonadaceae; g__Pseudomonas; s__	k__Bacteria;p__Proteobacteria;c__Gammaproteobacteria;o__Pseudomonadales;f__Pseudomonadaceae;g__Pseudomonas;Ambiguous_taxa
OTU931	k__Bacteria; p__TM7; c__TM7-1; o__; f__; g__; s__	k__Bacteria;p__Saccharibacteria;c__uncultured bacterium;o__uncultured bacterium;f__uncultured bacterium;g__uncultured bacterium;s__uncultured bacterium
OTU932	k__Bacteria; p__Proteobacteria; c__Gammaproteobacteria; o__Xanthomonadales; f__Xanthomonadaceae; g__; s__	k__Bacteria;p__Proteobacteria;c__Gammaproteobacteria;o__Xanthomonadales;f__Xanthomonadaceae;g__Silanimonas;s__uncultured bacterium
OTU933	k__Bacteria; p__Proteobacteria; c__Gammaproteobacteria; o__Pseudomonadales; f__Pseudomonadaceae; g__Pseudomonas; s__	k__Bacteria;p__Proteobacteria;c__Gammaproteobacteria;o__Pseudomonadales;f__Pseudomonadaceae;g__Pseudomonas
OTU934	k__Bacteria; p__Bacteroidetes; c__[Rhodothermi]; o__[Rhodothermales]; f__Rhodothermaceae; g__Rubricoccus; s__	k__Bacteria;p__Bacteroidetes;c__Bacteroidetes Incertae Sedis;o__Order II;f__Rhodothermaceae;g__Rubrivirga
OTU935	k__Bacteria; p__Bacteroidetes; c__[Saprospirae]; o__[Saprospirales]; f__Chitinophagaceae; g__; s__	k__Bacteria;p__Bacteroidetes;c__Sphingobacteriia;o__Sphingobacteriales;f__Chitinophagaceae;g__uncultured;s__uncultured bacterium
OTU936	Unassigned	Unassigned
OTU937	k__Bacteria; p__Proteobacteria; c__Alphaproteobacteria; o__Rhodospirillales; f__Acetobacteraceae; g__Gluconobacter; s__	k__Bacteria;p__Proteobacteria;c__Alphaproteobacteria;o__Rhodospirillales;f__Acetobacteraceae
OTU938	k__Bacteria; p__Proteobacteria; c__Alphaproteobacteria; o__Rhodobacterales; f__Rhodobacteraceae; g__Rubellimicrobium; s__	k__Bacteria;p__Proteobacteria;c__Alphaproteobacteria;o__Rhodobacterales;f__Rhodobacteraceae;g__Rubellimicrobium;Ambiguous_taxa
OTU939	k__Bacteria; p__Proteobacteria; c__Alphaproteobacteria; o__Sphingomonadales; f__Sphingomonadaceae; g__Sphingomonas; s__wittichii	k__Bacteria;p__Proteobacteria;c__Alphaproteobacteria;o__Sphingomonadales;f__Sphingomonadaceae;g__Sphingomonas
OTU94	k__Bacteria; p__Gemmatimonadetes; c__Gemm-3; o__; f__; g__; s__	k__Bacteria;p__Gemmatimonadetes;c__Longimicrobia;o__Longimicrobiales;f__Longimicrobiaceae;g__Longimicrobium
OTU940	k__Bacteria; p__Bacteroidetes; c__[Saprospirae]; o__[Saprospirales]; f__Chitinophagaceae; g__Flavisolibacter; s__	k__Bacteria;p__Bacteroidetes;c__Sphingobacteriia;o__Sphingobacteriales;f__Chitinophagaceae;g__Flavisolibacter
OTU941	Unassigned	Unassigned

OTU942	k__Bacteria; p__TM7; c__TM7-1; o__; f__; g__; s__	k__Bacteria;p__Saccharibacteria;c__uncultured bacterium;o__uncultured bacterium;f__uncultured bacterium;g__uncultured bacterium;s__uncultured bacterium
OTU943	k__Bacteria; p__Firmicutes; c__Bacilli; o__Lactobacillales; f__Lactobacillaceae; g__Lactobacillus; s__	k__Bacteria;p__Firmicutes;c__Bacilli;o__Lactobacillales;f__Lactobacillaceae;g__Lactobacillus;Ambiguous_taxa
OTU944	k__Bacteria; p__Proteobacteria; c__Gammaproteobacteria; o__Enterobacteriales; f__Enterobacteriaceae; g__; s__	k__Bacteria;p__Proteobacteria;c__Gammaproteobacteria;o__Enterobacteriales;f__Enterobacteriaceae;g__Enterobacter
OTU945	k__Bacteria; p__Bacteroidetes; c__[Saprospirae]; o__[Saprospirales]; f__Chitinophagaceae; g__; s__	k__Bacteria;p__Bacteroidetes;c__Sphingobacteriia;o__Sphingobacteriales;f__Chitinophagaceae;g__Parasegetibacter;s__uncultured bacterium
OTU946	k__Bacteria; p__Proteobacteria; c__Alphaproteobacteria; o__Rhizobiales; f__Methylobacteriaceae; g__Methylobacterium; s__	k__Bacteria;p__Proteobacteria;c__Alphaproteobacteria;o__Rhizobiales;f__Methylobacteriaceae;g__Methylobacterium;Ambiguous_taxa
OTU947	k__Bacteria; p__Proteobacteria; c__Alphaproteobacteria; o__Rhizobiales; f__Bradyrhizobiaceae; g__Balneimonas; s__	k__Bacteria;p__Proteobacteria;c__Alphaproteobacteria;o__Rhizobiales;f__Methylobacteriaceae;g__Microvirga;Ambiguous_taxa
OTU948	k__Bacteria; p__Acidobacteria; c__Solibacteres; o__Solibacterales; f__Solibacteraceae; g__; s__	k__Bacteria;p__Acidobacteria;c__Solibacteres;o__Solibacterales;f__Solibacteraceae (Subgroup 3);g__Bryobacter;s__uncultured bacterium
OTU949	k__Bacteria; p__Proteobacteria; c__Alphaproteobacteria; o__Rhizobiales; f__Rhizobiaceae; g__Agrobacterium; s__	k__Bacteria;p__Proteobacteria;c__Alphaproteobacteria;o__Rhizobiales;f__Rhizobiaceae;g__Rhizobium;Ambiguous_taxa
OTU95	k__Bacteria; p__Cyanobacteria; c__Oscillatoriophycideae; o__Chroococcales; f__Xenococcaceae; g__; s__	k__Bacteria;p__Cyanobacteria;c__Cyanobacteria
OTU950	Unassigned	k__Bacteria;p__Verrucomicrobia;c__Spartobacteria;o__Chthoniobacterales;f__Chthoniobacteraceae;g__Chthoniobacter;s__uncultured bacterium
OTU951	Unassigned	k__Bacteria;p__Proteobacteria;c__Alphaproteobacteria;o__Rhodospirillales;f__Acetobacteraceae;g__uncultured
OTU952	k__Bacteria; p__Proteobacteria; c__Alphaproteobacteria; o__Rhodobacterales; f__Rhodobacteraceae; g__Rubellimicrobium; s__	k__Bacteria;p__Proteobacteria;c__Alphaproteobacteria;o__Rhodobacterales;f__Rhodobacteraceae;g__Rubellimicrobium;s__uncultured bacterium
OTU953	k__Bacteria; p__Firmicutes; c__Bacilli; o__Gemellales; f__Gemellaceae; g__; s__	k__Bacteria;p__Firmicutes;c__Bacilli;o__Bacillales;f__Family XI;g__Gemella;s__uncultured bacterium
OTU954	k__Bacteria; p__Actinobacteria; c__Rubrobacteria; o__Rubrobacterales; f__Rubrobacteraceae; g__Rubrobacter; s__	k__Bacteria;p__Actinobacteria;c__Rubrobacteria;o__Rubrobacterales;f__Rubrobacteriaceae;g__Rubrobacter;s__uncultured bacterium
OTU955	k__Bacteria; p__Bacteroidetes; c__[Saprospirae]; o__[Saprospirales]; f__Chitinophagaceae; g__; s__	k__Bacteria;p__Bacteroidetes;c__Sphingobacteriia;o__Sphingobacteriales;f__Chitinophagaceae;g__Parafilimonas
OTU956	k__Bacteria; p__Firmicutes; c__Bacilli; o__Lactobacillales; f__Lactobacillaceae; g__Lactobacillus; s__	k__Bacteria;p__Firmicutes;c__Bacilli;o__Lactobacillales;f__Lactobacillaceae;g__Lactobacillus
OTU957	k__Bacteria; p__Bacteroidetes; c__Flavobacteriia; o__Flavobacteriales; f__[Weeksellaceae]; g__Chryseobacterium; s__	k__Bacteria;p__Bacteroidetes;c__Flavobacteriia;o__Flavobacteriales;f__Flavobacteriaceae
OTU958	k__Bacteria; p__Bacteroidetes; c__Cytophagia; o__Cytophagales; f__Cytophagaceae; g__Hymenobacter; s__	k__Bacteria;p__Bacteroidetes;c__Cytophagia;o__Cytophagales;f__Cytophagaceae;g__Hymenobacter;Ambiguous_taxa
OTU959	Unassigned	Unassigned
OTU96	k__Bacteria; p__Proteobacteria; c__Alphaproteobacteria; o__Caulobacterales; f__Caulobacteraceae; g__; s__	k__Bacteria;p__Proteobacteria;c__Alphaproteobacteria;o__Caulobacterales;f__Caulobacteraceae;g__Brevundimonas;Ambiguous_taxa
OTU960	Unassigned	Unassigned

OTU961	k__Bacteria; p__Fusobacteria; c__Fusobacteriia; o__Fusobacteriales; f__Fusobacteriaceae; g__Fusobacterium; s__	k__Bacteria;p__Fusobacteria;c__Fusobacteriia;o__Fusobacteriales;f__Fusobacteriaceae;g__Fusobacterium;s__uncultured bacterium
OTU962	k__Bacteria; p__Actinobacteria; c__Actinobacteria; o__Actinomycetales; f__Micrococcaceae; g__Arthrobacter; s__	k__Bacteria;p__Actinobacteria;c__Actinobacteria;o__Micrococcales;f__Micrococcaceae;g__Glutamicibacter;Ambiguous_taxa
OTU963	k__Bacteria; p__Bacteroidetes; c__Cytophagia; o__Cytophagales; f__Cytophagaceae; g__Dyadobacter; s__	k__Bacteria;p__Bacteroidetes;c__Cytophagia;o__Cytophagales;f__Cytophagaceae;g__Dyadobacter
OTU964	k__Bacteria; p__Actinobacteria; c__Actinobacteria; o__Actinomycetales; f__Dermatophilaceae	k__Bacteria;p__Actinobacteria;c__Actinobacteria;o__Micrococcales;f__Dermatophilaceae
OTU965	k__Bacteria; p__Firmicutes; c__Bacilli; o__Lactobacillales; f__Leuconostocaceae; g__Weissella; s__	k__Bacteria;p__Firmicutes;c__Bacilli;o__Lactobacillales;f__Leuconostocaceae;g__Weissella;Ambiguous_taxa
OTU966	Unassigned	Unassigned
OTU967	k__Bacteria; p__Bacteroidetes; c__Cytophagia; o__Cytophagales; f__Cytophagaceae; g__Hymenobacter; s__	k__Bacteria;p__Bacteroidetes;c__Cytophagia;o__Cytophagales;f__Cytophagaceae;g__Hymenobacter
OTU968	k__Bacteria; p__Bacteroidetes; c__[Saprospirae]; o__[Saprospirales]; f__Chitinophagaceae; g__; s__	k__Bacteria;p__Bacteroidetes;c__Sphingobacteriia;o__Sphingobacteriales;f__Chitinophagaceae;g__Ferruginibacter;s__uncultured bacterium
OTU969	k__Bacteria; p__Armatimonadetes; c__[Fimbriimonadia]; o__[Fimbriimonadales]; f__[Fimbriimonadaceae]; g__Fimbriimonas; s__	k__Bacteria;p__Armatimonadetes;c__Fimbriimonadia;o__Fimbriimonadales;f__Fimbriimonadaceae;g__uncultured bacterium;s__uncultured bacterium
OTU97	k__Bacteria; p__Bacteroidetes; c__Cytophagia; o__Cytophagales; f__Cytophagaceae; g__; s__	k__Bacteria;p__Bacteroidetes;c__Cytophagia;o__Cytophagales;f__Cytophagaceae;g__uncultured;s__uncultured bacterium
OTU970	Unassigned	Unassigned
OTU971	Unassigned	Unassigned
OTU972	Unassigned	Unassigned
OTU973	k__Bacteria; p__Proteobacteria; c__Gammaproteobacteria; o__Pseudomonadales; f__Moraxellaceae; g__Acinetobacter; s__rhizosphaerae	k__Bacteria;p__Proteobacteria;c__Gammaproteobacteria;o__Pseudomonadales;f__Moraxellaceae;g__Acinetobacter
OTU974	k__Bacteria; p__Proteobacteria; c__Deltaproteobacteria; o__Bdellovibrionales; f__Bdellovibrionaceae; g__Bdellovibrio; s__	k__Bacteria;p__Proteobacteria;c__Deltaproteobacteria;o__Bdellovibrionales;f__Bdellovibrionaceae;g__Bdellovibrio;s__uncultured bacterium
OTU975	k__Bacteria; p__Firmicutes; c__Bacilli; o__Lactobacillales; f__Leuconostocaceae; g__Weissella; s__	k__Bacteria;p__Firmicutes;c__Bacilli;o__Lactobacillales;f__Leuconostocaceae;g__Weissella;Ambiguous_taxa
OTU976	k__Bacteria; p__Cyanobacteria; c__ML635J-21; o__; f__; g__; s__	k__Bacteria;p__Cyanobacteria;c__ML635J-21;o__uncultured bacterium;f__uncultured bacterium;g__uncultured bacterium;s__uncultured bacterium
OTU977	k__Bacteria; p__Verrucomicrobia; c__[Spartobacteria]; o__[Chthoniobacterales]; f__[Chthoniobacteraceae]; g__Ellin506; s__	k__Bacteria;p__Verrucomicrobia;c__Spartobacteria;o__Chthoniobacterales;f__Chthoniobacteraceae;g__Chthoniobacter;s__uncultured bacterium
OTU978	Unassigned	Unassigned
OTU979	k__Bacteria; p__Bacteroidetes; c__[Saprospirae]; o__[Saprospirales]; f__Chitinophagaceae; g__; s__	k__Bacteria;p__Bacteroidetes;c__Sphingobacteriia;o__Sphingobacteriales;f__Chitinophagaceae;g__uncultured;s__uncultured bacterium
OTU98	k__Bacteria; p__TM7; c__SC3; o__; f__; g__; s__	k__Bacteria;p__Saccharibacteria;c__uncultured bacterium;o__uncultured bacterium;f__uncultured bacterium;g__uncultured bacterium;s__uncultured bacterium
OTU980	k__Bacteria; p__Proteobacteria; c__Alphaproteobacteria; o__Rhizobiales; f__Xanthobacteraceae; g__Labrys; s__	k__Bacteria;p__Proteobacteria;c__Alphaproteobacteria;o__Rhizobiales;f__Xanthobacteraceae;g__Labrys

OTU981	k__Bacteria; p__Proteobacteria; c__Gammaproteobacteria; o__Legionellales; f__Coxiellaceae; g__; s__	k__Bacteria;p__Proteobacteria;c__Gammaproteobacteria;o__Legionellales;f__Coxiellaceae;g__uncultured;s__uncultured bacterium
OTU982	Unassigned	k__Bacteria;p__Actinobacteria;c__Actinobacteria;o__Corynebacteriales;f__nbr16a11;g__uncultured bacterium;s__uncultured bacterium
OTU983	k__Bacteria; p__Planctomycetes; c__Phycisphaerae; o__WD2101; f__; g__; s__	k__Bacteria;p__Planctomycetes;c__Phycisphaerae;o__Tepidisphaerales;f__Tepidisphaeraceae;g__uncultured bacterium;s__uncultured bacterium
OTU984	k__Bacteria; p__Bacteroidetes; c__Cytophagia; o__Cytophagales; f__Cytophagaceae; g__Rudanella; s__	k__Bacteria;p__Bacteroidetes;c__Cytophagia;o__Cytophagales;f__Cytophagaceae;g__Fibrella
OTU985	k__Bacteria; p__Bacteroidetes; c__Flavobacteriia; o__Flavobacteriales; f__Flavobacteriaceae; g__Flavobacterium; s__	k__Bacteria;p__Bacteroidetes;c__Flavobacteriia;o__Flavobacteriales;f__Flavobacteriaceae;g__Flavobacterium;s__uncultured bacterium
OTU986	k__Bacteria; p__Proteobacteria; c__Betaproteobacteria; o__Burkholderiales; f__Comamonadaceae; g__; s__	k__Bacteria;p__Proteobacteria;c__Betaproteobacteria;o__Burkholderiales;f__Comamonadaceae;g__Pseudorhodoferax;s__uncultured bacterium
OTU987	k__Bacteria; p__Gemmatimonadetes; c__Gemmatimonadetes; o__Gemmatimonadales; f__; g__; s__	k__Bacteria;p__Gemmatimonadetes;c__Gemmatimonadetes;o__Gemmatimonadales;f__Gemmatimonadaceae;g__Gemmatirosa;s__uncultured bacterium
OTU988	k__Bacteria; p__Firmicutes; c__Bacilli; o__Bacillales; f__Paenibacillaceae; g__Paenibacillus; s__	k__Bacteria;p__Firmicutes;c__Bacilli;o__Bacillales;f__Paenibacillaceae;g__Paenibacillus;Ambiguous_taxa
OTU989	k__Bacteria; p__Proteobacteria; c__Alphaproteobacteria; o__Rhodospirillales; f__Acetobacteraceae; g__Gluconobacter; s__	k__Bacteria;p__Proteobacteria;c__Alphaproteobacteria;o__Rhodospirillales;f__Acetobacteraceae;g__Gluconobacter;Ambiguous_taxa
OTU99	k__Bacteria; p__Proteobacteria; c__Gammaproteobacteria; o__Xanthomonadales; f__Xanthomonadaceae; g__; s__	k__Bacteria;p__Proteobacteria;c__Gammaproteobacteria;o__Xanthomonadales;f__Xanthomonadaceae;g__Stenotrophomonas
OTU990	k__Bacteria; p__Actinobacteria; c__Thermoleophilia; o__Gaiellales; f__Gaiellaceae; g__; s__	k__Bacteria;p__Actinobacteria;c__Thermoleophilia;o__Gaiellales;f__uncultured;g__uncultured bacterium;s__uncultured bacterium
OTU991	k__Bacteria; p__Proteobacteria; c__Alphaproteobacteria; o__Rhizobiales; f__Beijerinckiaceae; g__; s__	k__Bacteria;p__Proteobacteria;c__Alphaproteobacteria;o__Rhizobiales;f__Methylobacteriaceae;g__uncultured;s__uncultured bacterium
OTU992	k__Bacteria; p__Proteobacteria; c__Alphaproteobacteria; o__Rhizobiales; f__Xanthobacteraceae; g__Labrys; s__	k__Bacteria;p__Proteobacteria;c__Alphaproteobacteria;o__Rhizobiales;f__Xanthobacteraceae;g__Labrys;s__uncultured bacterium
OTU993	k__Bacteria; p__Proteobacteria; c__Alphaproteobacteria; o__Rhodospirillales; f__Acetobacteraceae; g__; s__	k__Bacteria;p__Proteobacteria;c__Alphaproteobacteria;o__Rhodospirillales;f__Acetobacteraceae;g__Asaia
OTU994	k__Bacteria; p__Proteobacteria; c__Alphaproteobacteria; o__Sphingomonadales; f__Sphingomonadaceae; g__; s__	k__Bacteria;p__Proteobacteria;c__Alphaproteobacteria;o__Sphingomonadales;f__Sphingomonadaceae;g__Rhizorhapis;Ambiguous_taxa
OTU995	k__Bacteria; p__Bacteroidetes; c__Flavobacteriia; o__Flavobacteriales; f__Flavobacteriaceae; g__Flavobacterium; s__	k__Bacteria;p__Bacteroidetes;c__Flavobacteriia;o__Flavobacteriales;f__Flavobacteriaceae;g__Flavobacterium;s__uncultured bacterium
OTU996	Unassigned	Unassigned
OTU997	k__Bacteria; p__Proteobacteria; c__Gammaproteobacteria; o__Enterobacteriales; f__Enterobacteriaceae	k__Bacteria;p__Proteobacteria;c__Gammaproteobacteria;o__Enterobacteriales;f__Enterobacteriaceae;g__Cronobacter
OTU998	k__Bacteria; p__Planctomycetes; c__Planctomycetia; o__Pirellulales; f__Pirellulaceae; g__; s__	k__Bacteria;p__Planctomycetes;c__Planctomycetacia;o__Planctomycetales;f__Planctomycetaceae;g__Pirellula;s__uncultured bacterium
OTU999	k__Bacteria; p__Cyanobacteria; c__Oscillatoriophycideae; o__Chroococcales; f__Xenococcaceae; g__Chroococcidiopsis; s__	k__Bacteria;p__Cyanobacteria;c__Cyanobacteria;o__uncultured;f__uncultured bacterium;g__uncultured bacterium;s__uncultured bacterium
